# MiR‐182‐3p targets TRF2 and impairs tumor growth of triple‐negative breast cancer

**DOI:** 10.15252/emmm.202216033

**Published:** 2022-11-25

**Authors:** Roberto Dinami, Luca Pompili, Eleonora Petti, Manuela Porru, Carmen D'Angelo, Serena Di Vito, Angela Rizzo, Virginia Campani, Giuseppe De Rosa, Alejandra Bruna, Violeta Serra, Miguel Mano, Mauro Giacca, Carlo Leonetti, Gennaro Ciliberto, Madalena Tarsounas, Antonella Stoppacciaro, Stefan Schoeftner, Annamaria Biroccio

**Affiliations:** ^1^ Translational Oncology Research Unit IRCCS—Regina Elena National Cancer Institute Rome Italy; ^2^ Department of Ecological and Biological Sciences (DEB) University of Tuscia Viterbo Italy; ^3^ Department of Pharmacy University Federico II of Naples Naples Italy; ^4^ CRUK Cambridge Institute University of Cambridge Cambridge UK; ^5^ Vall d'Hebron Institute of Oncology Barcelona Spain; ^6^ Functional Genomics and RNA‐based Therapeutics Laboratory, Center for Neuroscience and Cell Biology (CNC) University of Coimbra Coimbra Portugal; ^7^ Department of Life Sciences University of Coimbra Coimbra Portugal; ^8^ King's College London, British Heart Foundation Centre of Research Excellence School of Cardiovascular Medicine & Sciences London UK; ^9^ Scientific Direction IRCCS‐Regina Elena National Cancer Institute Rome Italy; ^10^ Department of Oncology, Genome Stability and Tumourigenesis Group, MRC Oxford Institute for Radiation Oncology University of Oxford Oxford UK; ^11^ Department of Clinical and Molecular Medicine, St. Andrea Hospital Sapienza University of Rome Rome Italy; ^12^ Department of Life Sciences University of Trieste Trieste Italy

**Keywords:** miR‐182‐3p, target therapy, telomeres, TRF2, triple‐negative breast cancer, Autophagy & Cell Death, Cancer, DNA Replication, Recombination & Repair

## Abstract

The telomeric repeat‐binding factor 2 (TRF2) is a telomere‐capping protein that plays a key role in the maintenance of telomere structure and function. It is highly expressed in different cancer types, and it contributes to cancer progression. To date, anti‐cancer strategies to target TRF2 remain a challenge. Here, we developed a miRNA‐based approach to reduce TRF2 expression. By performing a high‐throughput luciferase screening of 54 candidate miRNAs, we identified miR‐182‐3p as a specific and efficient post‐transcriptional regulator of TRF2. Ectopic expression of miR‐182‐3p drastically reduced TRF2 protein levels in a panel of telomerase‐ or alternative lengthening of telomeres (ALT)‐positive cancer cell lines. Moreover, miR‐182‐3p induced DNA damage at telomeric and pericentromeric sites, eventually leading to strong apoptosis activation. We also observed that treatment with lipid nanoparticles (LNPs) containing miR‐182‐3p impaired tumor growth in triple‐negative breast cancer (TNBC) models, including patient‐derived tumor xenografts (PDTXs), without affecting mouse survival or tissue function. Finally, LNPs‐miR‐182‐3p were able to cross the blood–brain barrier and reduce intracranial tumors representing a possible therapeutic option for metastatic brain lesions.

## Graphical abstract

A miRNA‐based strategy to inhibit the telomeric protein TRF2 was developed, which led to efficient decrease of triple negative breast cancer growth.

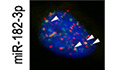

## Introduction

Telomeres are nucleoprotein structures localized at chromosome ends to ensure genomic stability. Human telomeres are composed of TTAGGG DNA tandem repeats bound by six proteins (TRF1, TRF2, POT1, TPP1, TIN2 and RAP1) forming a complex named shelterin (de Lange, 2018). Due to a phenomenon described as “end‐replication problem”, telomeres of somatic cells lose about 200 nucleotides during each cell division leading to replicative senescence (Olovnikov, 1973; Harley *et al*, 1990). In contrast, cancer cells counteract progressive telomere shortening by the activation of mechanisms, defined as telomere length maintenance mechanisms (TMMs), which confer an indefinite proliferative potential. The most common TMM (85–90% of human tumors) consists in the activation of telomerase, a reverse transcriptase formed by a catalytic subunit (TERT) and an RNA component (TERC), which acts as a template for *de novo* synthesis of TTAGGG repeats (Greider & Blackburn, 1985). The remaining 10–15% of human cancers exploit the homologous recombination pathway to elongate telomeres by a mechanism defined “Alternative Lengthening of Telomeres” (ALT) (Bryan *et al*, 1997; Recagni *et al*, 2020).

Over the last decades, telomerase has been deeply investigated as a prime telomeric target for anti‐cancer treatment by testing different classes of inhibitors (GRVAC1, GV1001, GRN163L), some of which are in clinical trials (Harley, 2008; Joseph *et al*, 2010). However, all the strategies adopted to inhibit telomerase activity have shown some limitations. First, telomerase inhibition only becomes efficient when telomeres have reached a critical length in tumors with *wild type* p53 (González‐Suárez *et al*, 2000; Perera *et al*, 2008). Moreover, it has been shown that a long period of treatment with telomerase inhibitors leads cancer cells to develop mechanisms of resistance, or to switch TMM from telomerase activation to ALT (Recagni *et al*, 2020).

Recent work suggests that alternative strategies for telomere targeting in human cancer could rely on the inhibition of shelterin components. In particular, TRF1 chemical inhibitors have been demonstrated to recapitulate the TRF1 knockdown phenotype and reduce tumor growth in xenografts from patient‐derived primary Glioma Stem Cells (GSCs) (Bejarano *et al*, 2019).

Together with TRF1, TRF2 is a main component of shelterin that is essential to block ataxia‐telangiectasia‐mutated (ATM) signaling and non‐homologous end joining (NHEJ) repair pathway at the end of chromosomes, thus preserving telomere stability (van Steensel *et al*, 1998; Celli & de Lange, 2005; Denchi & de Lange, 2007; Doksani *et al*, 2013). However, recent evidence indicates that the functional repertoire of TRF2 extends outside the telomeres. Indeed, TRF2 has been reported to be essential for the replication of pericentromeric sites and other “hard‐to‐replicate” heterochromatic regions, contributing to the maintenance of genome‐wide heterochromatin stability (Mendez‐Bermudez *et al*, 2018; Bauwens *et al*, 2021). In addition, our group demonstrated that TRF2, over‐expressed in various human cancers, promotes immune escape of cancer cells and tumor angiogenesis by controlling gene expression through the binding to a set of interstitial telomeric sequences (ITS) dispersed throughout the human genome (Biroccio *et al*, 2013; Maï *et al*, 2015; Cherfils‐Vicini *et al*, 2019; Zizza *et al*, 2019). The telomeric and extra‐telomeric tumor‐promoting functions of TRF2 make this shelterin component a very interesting target for anti‐cancer treatment.

microRNAs (miRNAs) are a class of small, non‐coding RNAs of 21–24 nucleotides, which play a crucial role in the post‐transcriptional regulation of gene expression by blocking translation or inducing degradation of their mRNA targets (O'Brien *et al*, 2018). Thanks to advancements in nucleic acid delivery systems, miRNAs represent an important tool for next‐generation cancer treatments (Wong & Cheah, 2020).

Here, by using a high‐throughput luciferase screening, we identified miR‐182‐3p as the most efficient human miRNA able to abrogate TRF2 expression, to activate DNA damage at telomeric and pericentromeric sites and to induce apoptosis in human cancer cells. Next, we delivered miR‐182‐3p using lipid nanoparticles (LNPs) in aggressive triple‐negative breast cancer (TNBC) mouse models. We found that treatment with LNPs‐miR‐182‐3p, by reducing TRF2 levels, was able to impair tumor growth in multiple TNBC models, including patient‐derived tumor xenografts (PDTXs), BRCA proficient and deficient, resistant to PARP inhibitors. This study proposes, for the first time, an anticancer miRNA‐based approach to target TRF2 *in vivo*.

## Results

### High‐throughput screening identifies miR‐182‐3p as a novel regulator of TRF2 expression

**Figure 1:**
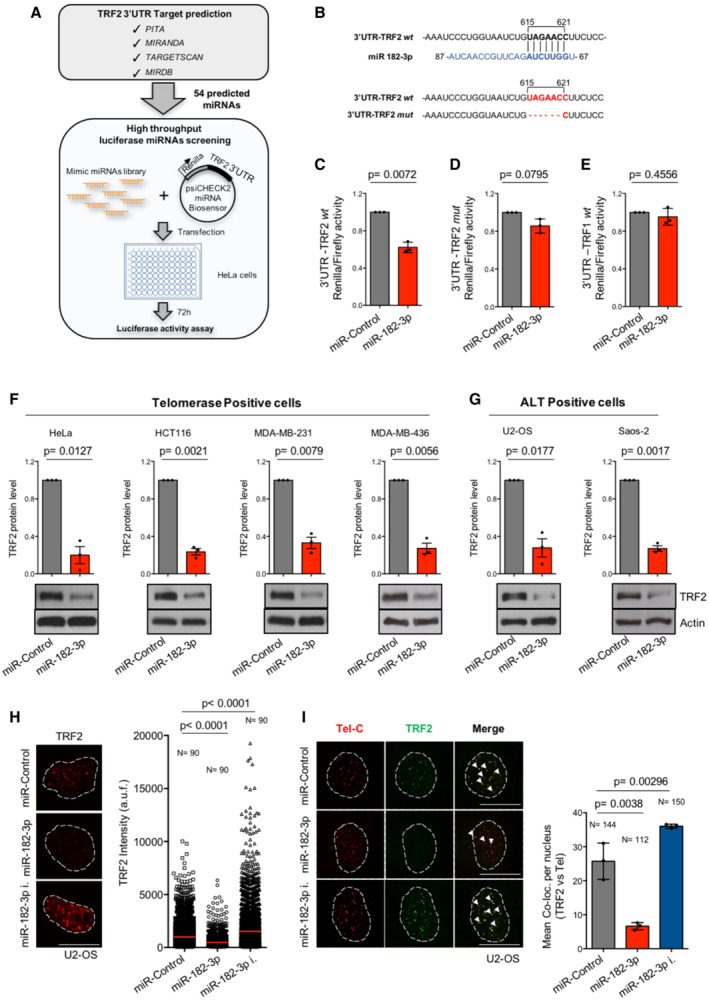
miR‐182‐3p reduces TRF2 expression in different cancer cell lines ASchematic representation of luciferase screening approach. Upper panel shows the four target predictions software used for *in silico* analysis. Bottom panel indicates the main steps performed in the high‐throughput screening.BUpper panel, sequence interaction of miR‐182‐3p with the target site of the *wild type* 3′UTR of TRF2 in human. Bottom panel, generation of mutant 3′UTR of TRF2 luciferase construct containing the deletion of target site for miR‐182‐3p.C–ELuciferase reporter assay in HeLa cells using the synthetic miR‐Control or miR‐182‐3p in combination with the *wild type* (C) or the mutant 3′UTR of TRF2 construct (D) or the *wild type* 3′UTR of TRF1 (E).F, GWestern blotting for TRF2 expression in telomerase‐positive (HeLa, HCT116, MDA‐MB‐231, MDA‐MB‐436) and ALT‐positive (U2‐OS, Saos‐2) cells transiently transfected with miR‐Control or miR‐182‐3p. Upper panel shows the quantification of TRF2 expression. Bottom panel, representative images are shown, actin was used as loading control.HU2‐OS cells transiently transfected with the miR‐Control, miR‐182‐3p or miR‐182‐3p inhibitor were assayed by quantitative immunofluorescence for TRF2 3 days post‐transfection. Left panel, representative images. Scale bar: 10 μm. Right panel, quantification of TRF2 fluorescence intensity. a.f.u. arbitrary fluorescence units. *N* = number of analyzed nuclei. Red bar indicates mean value.IU2‐OS cells transfected as described in (H) were assayed by immunofluorescence combined with telomeric FISH. Left panel, representative images of co‐localizations between TRF2 and telomeres (white arrowheads). Scale bar: 10 μm. Right panel, co‐localizations were analyzed using ImageJ software. *N* = number of analyzed nuclei. Data information: For (C–G and I), data are shown as mean ± SD. Three independent experiments were performed (*n* = 3). *P* values are determined by Student's *t*‐test; for (H), *P* values are determined by Mann–Whitney *t*‐test. Source data are available online for this figure.

**Figure EV1:**
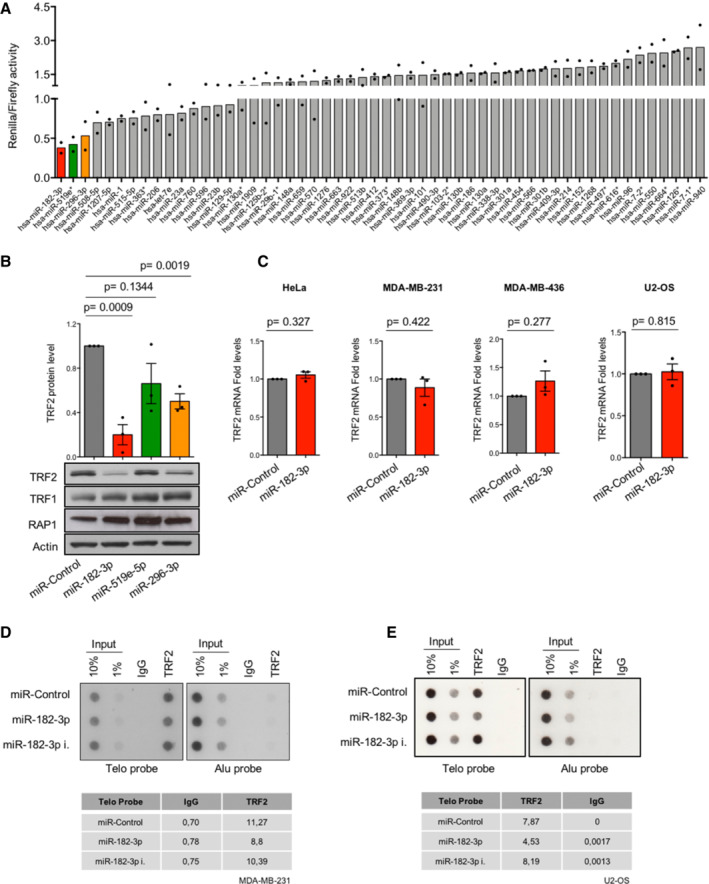
High‐throughput luciferase miRNAs screening identifies miR‐182‐3p as the most efficient miRNA able to target TRF2 AResults of high‐throughput luciferase screening performed in Hela cells using the wild type 3′UTR‐TRF2 vector in combination with each of the 54 miRNAs selected by *in silico* analysis. Three days post‐transfection, luciferase ratio (Renilla:Firefly) of each miRNA was calculated, the control miRNA was set “1.” Renilla:Firefly ratios < 1 indicate target specificity of candidate miRNAs for the 3′UTR of TRF2. miRNAs near to the ratio of 0.5 were considered for further analysis. Two biological replicates were performed.BHeLa cells transiently transfected with the indicated miRNAs (miR‐Control, miR‐182‐3p, miR‐519e‐5p, miR‐296‐3p) were assayed by western blotting. Upper panel, quantification of TRF2 expression. Bottom panel, representative images of TRF2, TRF1 and RAP1 are shown, actin was used as loading control.CAnalysis of TRF2 mRNA expression performed by qPCR in four different cancer cell lines (HeLa, MDA‐MB‐231, MDA‐MB‐436, U2‐OS) 3 days post‐transfection with miR‐Control or miR‐182‐3p. The control miRNA was set “1.” Three independent experiments were performed.D, ETelomeric ChIP assay in MDA‐MB‐231 (D) and U2‐OS cells (E). Quantification of TRF2 enrichment at telomeric repeats, in the different conditions, is shown in the table under the respective figure. Alu probe and Rabbit IgG were used as negative control for the assay. Data information: For (A), data are presented as mean values. For (B, C), data are presented as mean values ± SD and Student *t‐*test was used to calculate statistical significance. Source data are available online for this figure.

To develop a miRNA‐based strategy to target TRF2 in human cancer, we used a multistep approach. First, we matched computational analyses from four target prediction software to draw a list of candidate miRNAs with target specificity for the 3′UTR of TRF2. Next, by applying a cut‐off based on the guideline of each software, we obtained 54 candidate miRNAs that were tested through a high‐throughput luciferase screening (Fig 1A). Briefly, HeLa cells were transiently co‐transfected with a luciferase reporter vector containing the Renilla luciferase cDNA fused to the full length 3′UTR of TRF2 in combination with each synthetic mimic‐miRNA (Figs 1A and EV1A). By measuring luciferase activity, we found that three miRNAs (miR‐182‐3p; miR‐519e*; miR‐296‐3p) reduce by ~ 50% the Renilla/Firefly luminescence ratio (Fig EV1A; Table EV1). In order to select the most efficient one, we compared their efficacy in inhibiting TRF2 expression. Western blotting analysis revealed that miR‐182‐3p was the most effective miRNA able to inhibit TRF2 expression, inducing a reduction of more than 75% (Fig EV1B).

To validate miR‐182‐3p target specificity, wild type (wt) 3′UTR of TRF2 (Fig 1B) and a mutant of TRF2 carrying a deletion of the miR‐182‐3p target site (Fig 1B) were used in luciferase reporter assays. Moreover, the 3′UTR of TRF1, a related telomeric protein lacking miR‐182‐3p target site, was also included in the same assay. Interestingly, miR‐182‐3p significantly reduced the reporter activity of TRF2 3′UTR, while no effect was observed on mutant TRF2 and on the TRF1 (Fig 1C–E). In line with these observations, ectopic expression of miR‐182‐3p did not impact on TRF1 protein expression and did not indirectly reduce RAP1 protein levels (Fig EV1B).

To verify whether the ability of the miRNA to reduce TRF2 expression is not dependent on the cellular context, miR‐182‐3p was expressed in a panel of telomerase‐ (HeLa, HCT116, MDA‐MB‐231, MDA‐MB‐436) and ALT‐positive (U2‐OS, Saos‐2) cancer cells. We found that ectopic expression of miR‐182‐3p strongly decreased TRF2 protein levels in all tested cancer cell lines (Fig 1F and G), without affecting the mRNA expression (Fig EV1C).

In line with these results, immunofluorescence analysis showed that ectopic expression of miR‐182‐3p significantly reduced TRF2 spot intensity, while miR‐182‐3p inhibitor was able to enhance TRF2 signal in U2‐OS cells (Fig 1H). Moreover, we demonstrated that miR‐182‐3p decreased TRF2 abundance at telomeres as shown by immune‐FISH (Fig 1I) and ChIP assays in Telomerase‐ and ALT‐positive cells (Fig EV1D and E).

Altogether, these data identify miR‐182‐3p as a novel regulator of TRF2 protein expression in human cancer cells.

### miR‐182‐3p induces telomeric and pericentromeric DNA damage

**Figure EV2:**
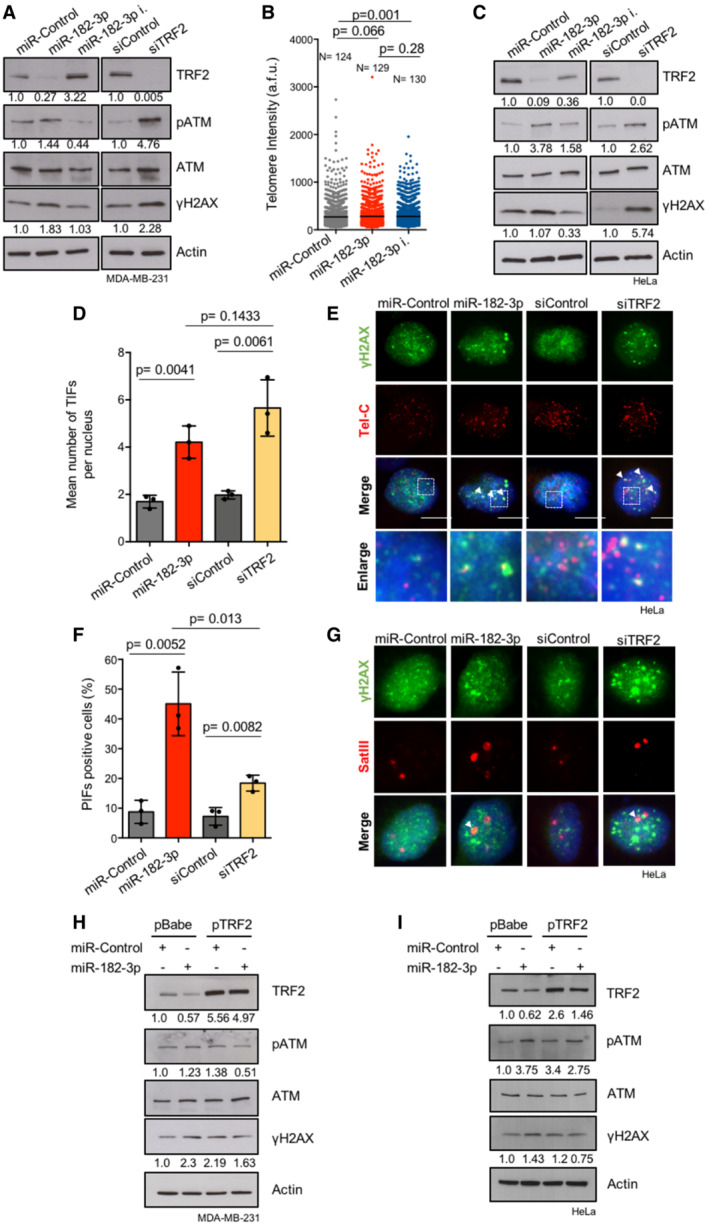
Silencing of TRF2 induces telomeric, pericentromeric and global DNA damage activation AMDA‐MB‐231 cells were transiently transfected with the indicated miRNAs or siRNA. The indicated DNA damage markers were assayed by western blotting. Actin was used as loading control.BTelomeric DNA FISH performed in MDA‐MB‐231 transiently transfected with the indicated miRNAs. Telomere length was measured by TLF software and indicated as arbitrary fluorescence unit (a.f.u). *N* = number of analyzed nuclei. Black bar indicates mean value.CDNA damage markers were assayed by western blotting in HeLa cells. Actin was used as loading control.DImmunofluorescence analysis of γH2AX combined with a telomeric FISH probe (TIFs) was performed in HeLa cells transfected with the indicated miRNAs or siRNAs. Co‐localizations of γH2AX with telomeres are indicated as mean number of TIFs per nucleus.ERepresentative images and enlargements of co‐localizations of experiment described in D.FImmunofluorescence analysis of γH2AX combined with a SatIII FISH probe (PIFs) was performed in HeLa cells transfected with the indicated miRNAs or siRNAs. The γH2AX‐positive cells with ≥ 1 PIFs per nucleus were analyzed.GRepresentative images of co‐localizations relative to the experiment described in (F).H, IMDA‐MB‐231 and HeLa cells over‐expressing TRF2 or an empty vector (pBabe) were transiently transfected with miR‐Control or miR‐182‐3p. TRF2, pATM and γH2AX expression were assayed by western blotting. Actin was used as loading control. Data information: For (D) and (F), data are presented as mean values ± SD. Three independent replicates were performed. Scale bar: 10 μm. At least 60 nuclei were analyzed in (D) and (F). A Student *t‐*test was used to calculate statistical significance. For (B), *P* values are determined by Mann–Whitney *t*‐test. All the experiments were performed 3 days post‐transfection with the indicated miRNAs or siRNAs. Source data are available online for this figure.

Recent evidence highlights a role for TRF2 in the maintenance of genome‐wide heterochromatin stability. Consistently, TRF2 knockdown triggers DNA damage activation at heterochromatic regions such as telomeric and pericentromeric repeats (de Lange, 2018; Mendez‐Bermudez *et al*, 2018). By analyzing DNA damage response markers upon transient miRNA transfection, we found that miR‐182‐3p, similarly to siRNA‐mediated TRF2 knockdown, induces a marked activation of pATM and γH2AX in MDA‐MB‐231 TNBC cells (Fig EV2A). Of note, abrogation of miR‐182‐3p by using a specific inhibitor increases TRF2 levels with consequent inhibition of pATM and γH2AX damage pathway (Fig EV2A).

**Figure 2:**
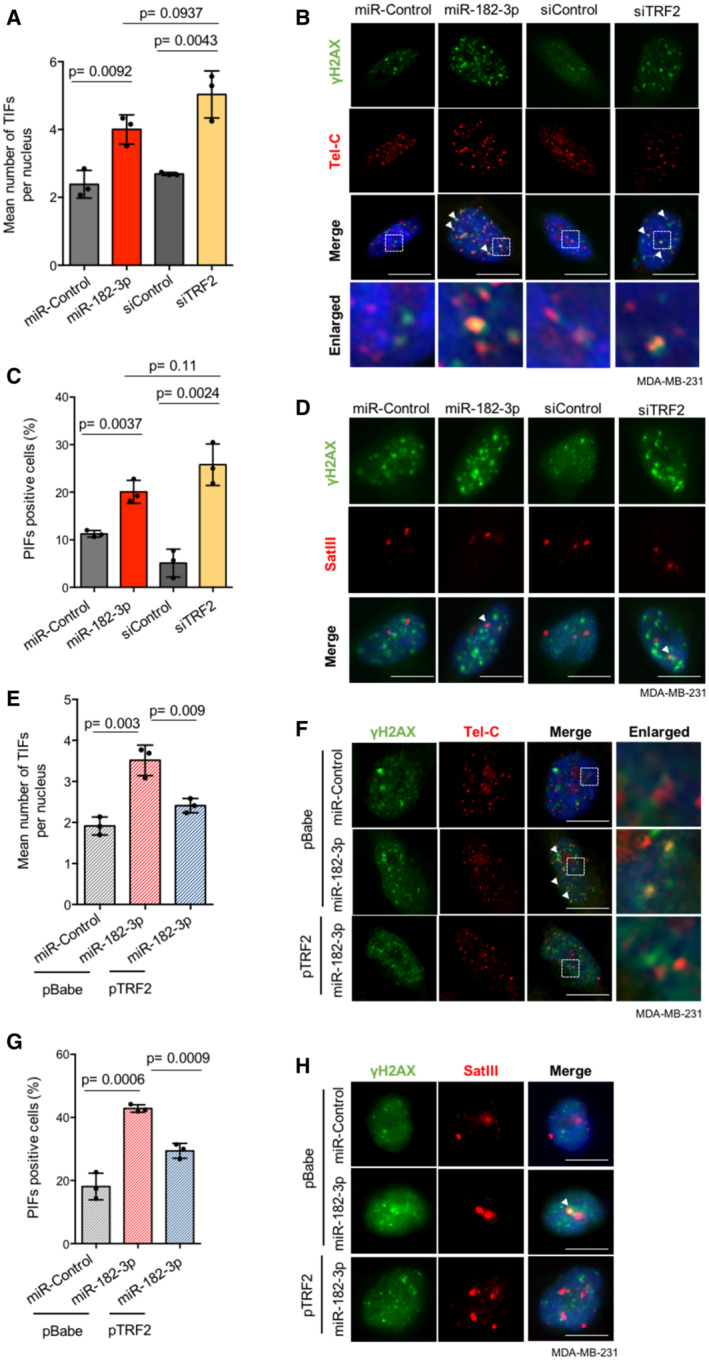
miR‐182‐3p induces telomeric and pericentromeric DNA damage by TRF2 abrogation Immunofluorescence analysis of γH2AX combined with telomeric FISH (TIFs) was performed in MDA‐MB‐231 cells transfected with the indicated miRNAs or siRNAs. The mean number of TIFs per nucleus was analyzed.Representative images and enlargements of co‐localizations (white arrowheads) relative to the experiment described in (A). Scale bar: 10 μm.Immunofluorescence analysis of γH2AX combined with a SatIII FISH probe (PIFs) was performed in MDA‐MB‐231 cells transfected with the indicated miRNAs or siRNAs. The γH2AX‐positive cells with ≥ 1 PIFs per nucleus were analyzed.Representative images of co‐localizations (white arrowheads) relative to the experiment described in (C). Scale bar: 10 μm.Quantification of TIFs in MDA‐MB‐231 cells over‐expressing TRF2 or an empty vector (pBabe), transfected with indicated miRNAs. The mean number of TIFs per nucleus was quantified.Representative images and enlargements relative to the experiment described in (E). White arrowheads indicate co‐localizations events. Scale bar: 10 μm.Quantification of PIFs in MDA‐MB‐231 cells over‐expressing TRF2 or an empty vector (pBabe), transfected with indicated miRNAs. The γH2AX‐positive cells with ≥ 1 PIFs per nucleus were analyzed.Representative images relative to the experiment described in (G). White arrowheads indicate co‐localizations events. Scale bar: 10 μm. Data information: For (A, C, E, G) data are shown as mean ± SD. Three independent experiments were performed (*n* = 3). *P* values are determined by unpaired two‐tailed *t‐*test. At least 60 nuclei were analyzed for each experimental condition. All the experiments were performed 3 days post‐transfection with the indicated miRNAs or siRNAs. Source data are available online for this figure.

To investigate whether DNA damage response caused by ectopic expression of miR‐182‐3p is localized at telomeres or pericentromeres, Telomere dysfunction‐Induced Foci (TIFs) and Pericentromere‐dysfunction Induced Foci (PIFs) were evaluated by combining γH2AX immunofluorescence with DNA FISH, using a specific probe for telomeric (Tel‐C) or pericentromeric (SatIII) repeats. We found that miR‐182‐3p over‐expression was able to strongly increase telomeric and pericentromeric DNA damage, reproducing the effect of TRF2 siRNA, without affecting telomere length in MDA‐MB‐231 cells (Fig 2A–D and EV2B). The ability of miR‐182‐3p to induce DNA damage was also recapitulated in HeLa and U2‐OS cells indicating that this is a general phenomenon occurring in both telomerase‐ and ALT‐positive cells (Fig EV2C–G; Appendix Fig S1A–C).

Finally, to verify that DNA damage response induced by miR‐182‐3p is directly linked to TRF2 inhibition, we transfected miR‐182‐3p in TRF2 over‐expressing cells. Interestingly, we found that DNA damage markers activation (pATM and γH2AX) caused by miR‐182‐3p was attenuated in MDA‐MB‐231 and HeLa cells over‐expressing TRF2 compared to their control (Fig EV2H,I). Moreover, telomeric and pericentromeric DNA damage induced by miR‐182‐3p in control cells (pBabe) was significantly counteracted by TRF2 over‐expression (pTRF2) (Fig 2E–H), thereby demonstrating that DNA damage activation caused by miR‐182‐3p is a consequence of TRF2 modulation.

Overall, these results indicate that miR‐182‐3p, by regulating TRF2 expression, activates the DNA damage response with a specific impact on telomeric and pericentromeric regions.

### Ectopic expression of miR‐182‐3p induces apoptosis in triple‐negative breast cancer cells

**Figure 3:**
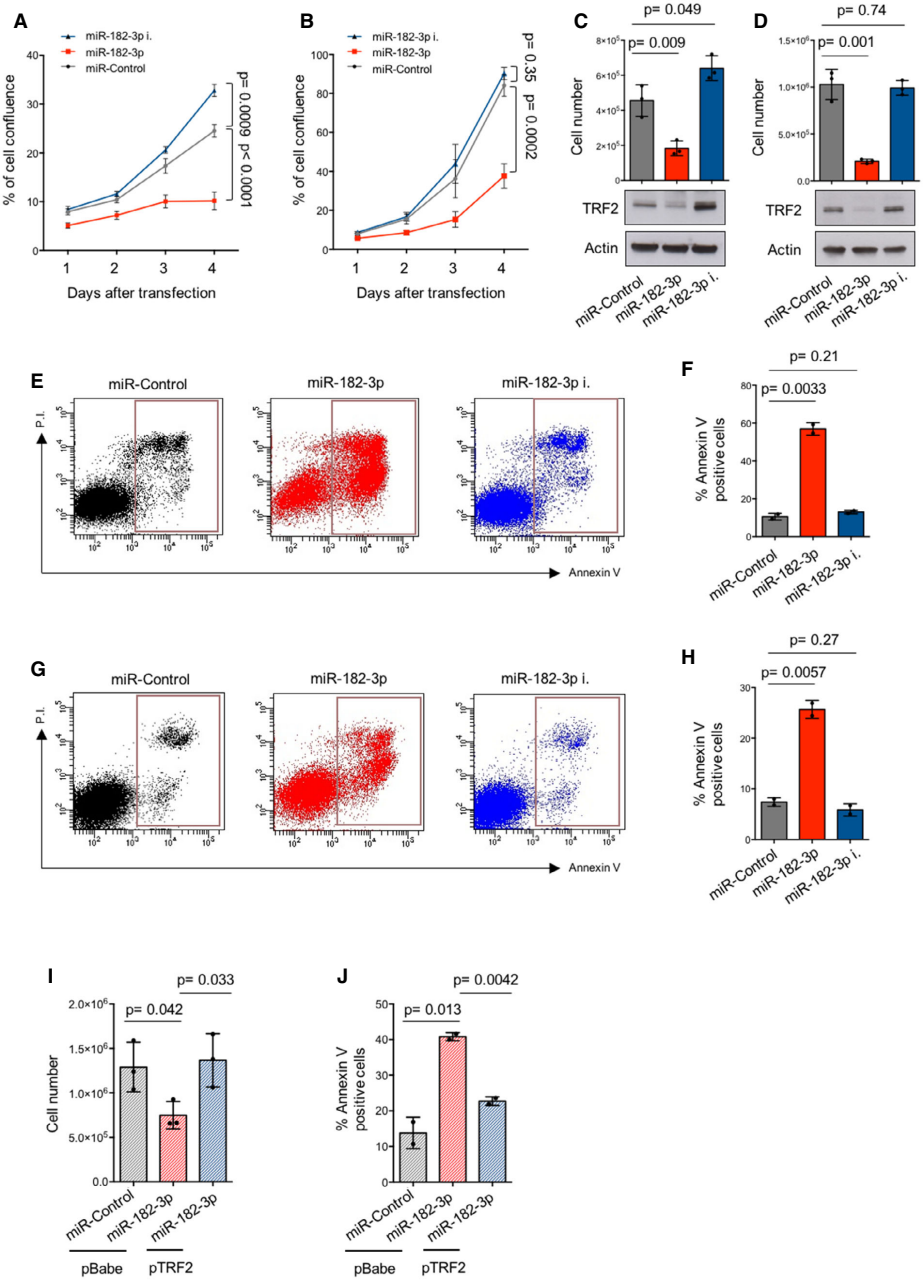
Ectopic expression of miR‐182‐3p reduces cell growth by apoptosis activation A, BMDA‐MB‐436 and MDA‐MB‐231 cells underwent two rounds of transfection with miR‐Control, miR‐182‐3p or miR‐182‐3p inhibitor. Starting from the day of the second transfection, cell confluence was monitored by Incucyte every 24 h up to a maximum of 3 days. The percentage of cell confluence was analyzed.C, DCell number of MDA‐MB‐436 (C) and MDA‐MB‐231 (D) cells and TRF2 expression were analyzed by automatic cell count and by western blotting at the end of the experiment described in (A) and (B). Actin was used as loading control.ETwo‐dimensional scatter plots of Annexin V analysis performed in MDA‐MB‐436 at the end of the second cycle of transfection with miR‐Control, miR‐182‐3p or miR‐182‐3p inhibitor. Red boxes indicate early and late apoptotic cells.FQuantification of Annexin V‐positive cells (%) of experiment described in (E).GTwo‐dimensional scatter plots of Annexin V analysis performed in MDA‐MB‐231 as described in (E).HQuantification of Annexin V‐positive cells (%) of experiment described in (G).I, JMDA‐MB‐436 cells over‐expressing TRF2 or an empty vector (pBabe) were transiently transfected with indicated miRNAs and cell count (I) or apoptosis (J) analysis was performed 72 h post‐transfection. Data information: For (A, B) data are shown as mean ± SEM. For (C, D, F, H, I, J), data are shown as mean ± SD. For (A–D) and (I), three independent experiments were performed (*n* = 3). *P* values are determined by unpaired two‐tailed *t‐*test. For (F), (H) and (J), two different biological replicates were performed. Source data are available online for this figure.

The above results raise the interesting possibility that DNA damage induced by miR‐182‐3p in malignant cells may rapidly promote growth inhibition and/or cell death. As we previously mentioned, TRF2 is up‐regulated in various human malignancies, including breast cancer. Among breast cancer molecular subtypes, triple‐negative is the most aggressive and still lacks a targeted therapy. For this reason, we tested the efficacy of miR‐182‐3p‐mediated targeting of TRF2 in two TNBC cell lines: MDA‐MB‐436, which is BRCA1‐compromised, and MDA‐MB‐231, which instead is characterized by a functional HR pathway. Both cell lines were transfected with miR‐182‐3p, miR‐182‐3p inhibitor and miR‐Control and then monitored by time‐lapse microscopy. Interestingly, in both cell lines, we observed a time‐dependent inhibition of cell growth upon miR‐182‐3p transfection, reaching the maximum effect at day 4 when a cell confluence inhibition of more than 50% was observed (Fig 3A and B). Conversely, ectopic expression of miR‐182‐3p inhibitor increased cell proliferation (Fig 3A and B). The effect of miR‐182‐3p on cell growth was also confirmed by counting the number of cells at the end of the experiment (Fig 3C and D). This effect was associated with a significant reduction of TRF2 levels in miR‐182‐3p transfected cells compared to control and miRNA inhibitor (Fig 3C and D) and, furthermore, with a drastic change of cell morphology towards a more rounded shape, not associated with induction of senescence (Appendix Fig S2A and B).

Next, we evaluated whether the effect on cell growth was due to the inhibition of cell proliferation and/or apoptosis. Propidium Iodide (PI) staining performed in both TNBC cell lines showed that miR‐182‐3p induced an increase of subG1 peak, indicative of apoptosis, paralleled by a reduction of cells in the other phases of the cell cycle (Appendix Fig S2C–F). In line with this observation, annexin V analysis evidenced that miR‐182‐3p strongly triggered apoptosis (Fig 3E–H), that was exacerbated in HR (BRCA1 or BRCA2)‐deficient cells (Fig 3E and F; Appendix Fig S2G and H). On the contrary, only a slight reduction in bromodeoxyuridine (BrdU) incorporation was observed (S phase; Appendix Fig S2I–L), suggesting that apoptosis is the main mechanism by which miR‐182‐3p exerts its anti‐tumoral activity. Interestingly, telomeric FISH on metaphase spreads of MDA‐MB‐436 cells revealed that TRF2 inhibition by miR‐182‐3p induced telomeric aberrations, such as telomere free ends and sister chromatid fusions without impacting on telomere fragility (Appendix Fig S3A–C). Moreover, we also observed an increment of micronuclei at day 3 which undergo to a further increase at day 6 (Appendix Fig S3D and E), suggesting that a progressive accumulation of genomic/telomeric instability may be responsible for apoptosis.

To test whether the pro‐apoptotic effect of miR‐182‐3p was specifically mediated by TRF2 targeting, we transfected miR‐182‐3p in TRF2 over‐expressing (pTRF2) MDA‐MB‐436 cells. We found that cell growth inhibition and apoptosis caused by miR‐182‐3p was significantly counteracted by TRF2 over‐expression (Fig 3I and J), thus demonstrating that TRF2 inhibition plays a key role in the pro‐apoptotic effect of miR‐182‐3p. The effect of miR‐182‐3p, in terms of cell growth and apoptosis, was also recapitulated in ALT‐positive cells (Appendix Fig S4A–D), demonstrating that miR‐182‐3p limits the growth of both telomerase‐ and ALT‐positive cells.

**Figure 4:**
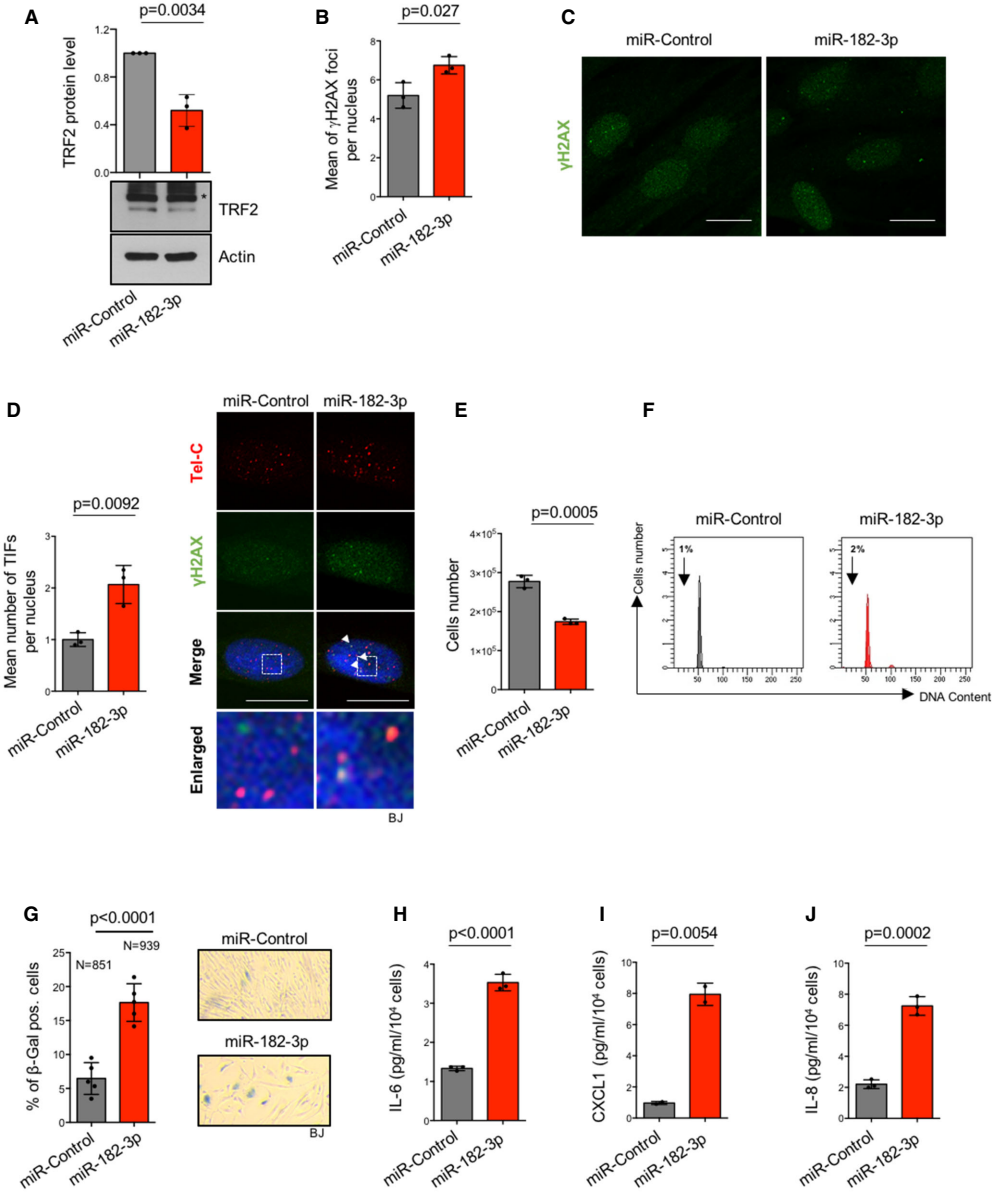
Ectopic expression of miR‐182‐3p reduces cell growth by senescence activation in normal cells AWestern blotting for TRF2 expression in BJ cells transiently transfected with miR‐Control or miR‐182‐3p. The graph represents the quantification of three independent experiments. Representative images are shown, Actin was used as loading control. Unspecific bands are indicated with (*).B, CMean of γH2AX foci per nucleus was analyzed in BJ cells 72 h post‐transfection with the indicated miRNAs. Representative images of γH2AX foci are shown in (C).DImmunofluorescence analysis of γH2AX combined with a telomeric FISH probe (TIFs) was performed in BJ cells 72 h post‐transfection with the indicated miRNAs. Left panel: The mean number of TIFs per nucleus was analyzed. Right panel: Representative images and enlargements of co‐localizations.ECell number of BJ cells was analyzed by automatic cell count at the end of the second round of transfection with miR‐Control or miR‐182‐3p.FFACS analysis to evaluate cell cycle progression by Propidium Iodide (PI) staining in BJ cells treated as indicated in (E).Gβ‐Galactosidase assay in BJ cells after two rounds of transfection with mimic miR‐Control or miR‐182‐3p. Left panel: Analysis of β‐galactosidase‐positive cells. Right panel: Representative images.H–JIL‐6 (H), CXCL1 (I), IL‐8 (J) factors were analyzed by ELISA to evaluate the senescence‐associated secretory phenotype (SASP) in BJ cells treated as indicated in (G). Data information: For (A, B, D, E and G–J), a student *t‐*test was used to calculate statistical significance. Scale bars (10 μm). *P* values are indicated. Source data are available online for this figure.

**Figure EV3:**
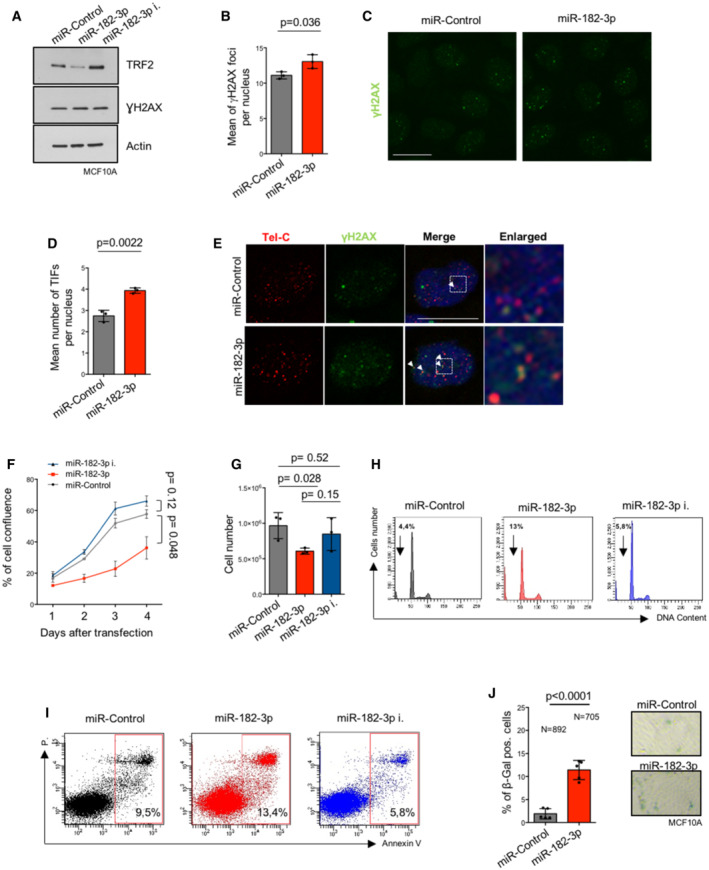
Effects of miR‐182‐3p over‐expression in epithelial breast cancer cells ATRF2 and γH2AX expression after two rounds of transfection with the indicated miRNAs, was analyzed by western blotting in MCF10A cells. Actin was used as loading control.B–EThe mean number of γH2AX foci (B) and TIFs (D) per nucleus were analyzed 72 h post‐transfection with the indicated mimic miRNAs in MCF10A cells. Representative images (C) and (E) are referred to the experiment showed in (B) and (D) respectively.F, GCell confluence (F) of MCF10A was monitored by Incucyte, every 24 h starting from the day of the second transfection, and cell number (G) was counted at the end of experiment (day 4).H–ICell cycle progression analysis by PI staining (H) and cell death analysis by Annexin V assay (I) were performed in MCF10A upon two rounds of transfection with the indicated miRNAs.Jβ‐Galactosidase assay in MCF10A cells after two rounds of transfection with mimic miR‐Control or miR‐182‐3p. Left panel: Analysis of β‐galactosidase‐positive cells. Right panel: Representative images. Data information: Panels (B, D, F, G, J) data are presented as mean values ± SD. A Student *t‐*test was used to calculate statistical significance. *P* values are indicated. Source data are available online for this figure.

Finally, the effects of miR‐182‐3p were also evaluated in normal cells. In primary human skin fibroblasts (BJ), reduction of TRF2 expression mediated by miR‐182‐3p induced DNA damage activation leading to a growth inhibition of about 35% at day 6 post‐transfection (Fig 4A–E). Inhibition of cell growth was not caused by apoptosis, as observed by the absence of subG1 pick in the cell cycle analysis, but was associated with a significant increase of senescent cells, as also confirmed by the induction of Senescent‐Associated Secretory Phenotype (SASP) factors such as IL‐6, CXCL1 and IL‐8 (Fig 4F–J). Telomeric DNA damage, inhibition of proliferation and induction of senescence/apoptosis upon miR‐182‐3p transfection were also observed in immortalized breast epithelial cells (MCF10A) (Fig EV3A–J).

### LNPs‐miR‐182‐3p induces tumor inhibition in triple‐negative breast cancer models

To explore the anti‐tumor potential of miR‐182‐3p *in vivo*, we took advantage of the possibility of delivering efficiently and safely this miRNA in mice using LNPs. Similar to previous studies (de Antonellis *et al*, 2013; di Martino *et al*, 2014; Scognamiglio *et al*, 2014; Fattore *et al*, 2020), LNPs were formulated as described in Materials and Methods; their main biophysical properties are reported in Table 1.

**Table 1:** LNPs‐miRNAs size, polydispersity index (PI), zeta potential (ZP), actual loading and encapsulation efficiency (EE%).

Formulation	miRNA	Diameter (nm ± SD)	PI ± SD	ZP (mV ± SD)	Actual loading ± SD	EE% ± SD
LNP	–	121.7 ± 15.6	0.12 ± 0.06	−8.5 ± 4.8	–	–
LNP1	miR‐Control	140.0 ± 11.3	0.17 ± 0.04	−14.7 ± 6.2	192.4 ± 4.1	96 ± 2.1
LNP2	miR‐182‐3p	152.9 ± 15.3	0.20 ± 0.04	−18.5 ± 5.6	194.1 ± 1.8	97 ± 0.8

**Figure 5:**
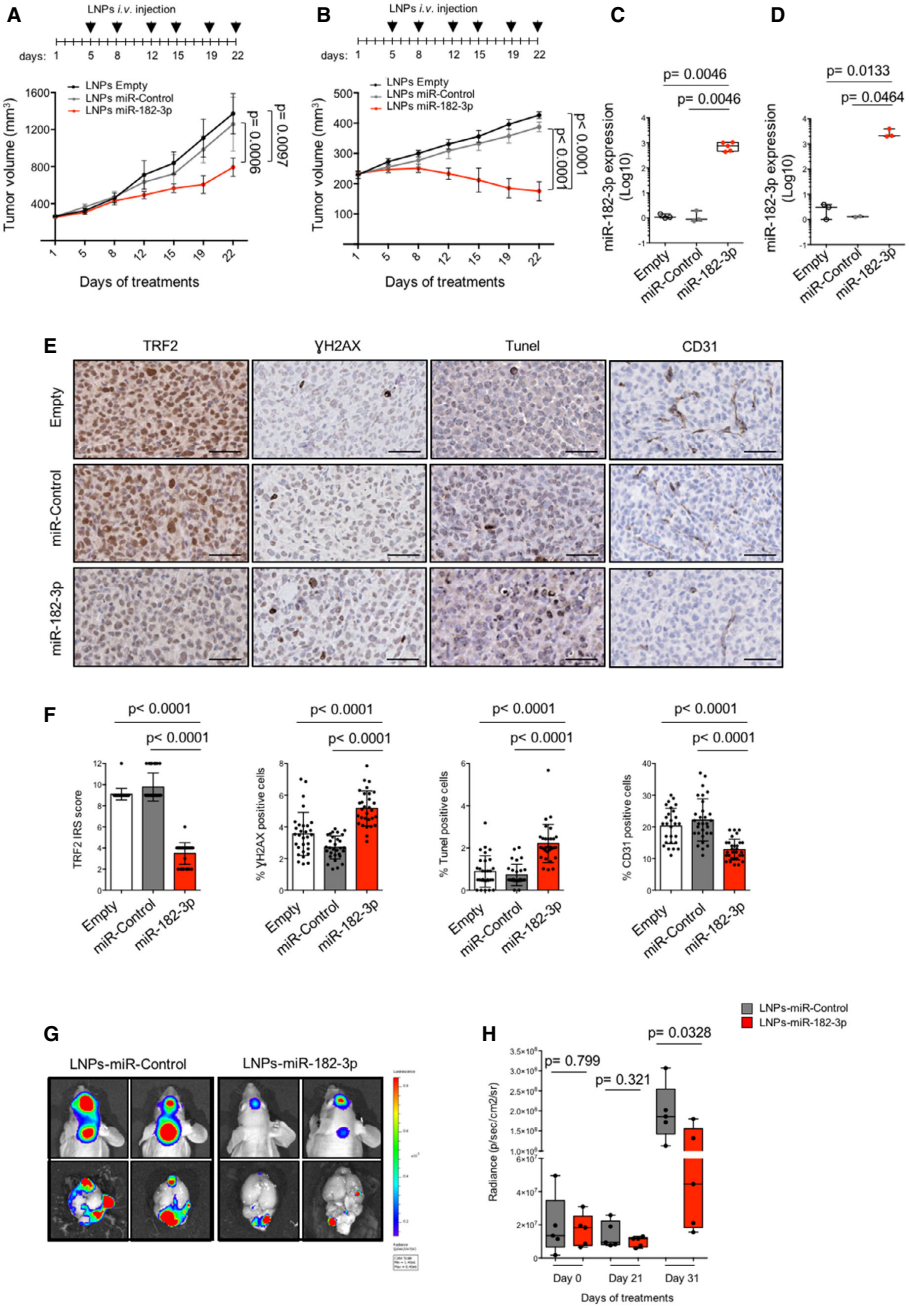
LNPs‐miR‐182‐3p treatment inhibits tumor growth *in vivo* A, BMDA‐MB‐231 (A) and MDA‐MB‐436 (B) tumor xenografts were treated with LNPs‐empty, LNPs‐miR‐Control or by LNPs‐miR‐182‐3p when the tumors became palpable. Mice were treated 6 times by intravenous tail vein injections with 20 μg of LNPs‐miR‐Control, LNPs‐miR‐182‐3p or equivalent volume of LNPs‐empty as indicated in the scheduling. The mean of tumor volumes (*n* = 5 per group) is shown.C, DTumors from mice treated in (A) and (B) were processed to measure miR‐182‐3p expression by TaqMan qPCR.ERepresentative images of IHC analysis of the indicated markers on tumor samples from mice bearing MDA‐MB‐231 human breast cancer xenografts. Scale bar: 50 μm.FThe histograms show the expression of TRF2, calculated as immunoreactivity score (IRS) by IHC, and the count of positive cells to γH2AX, TUNEL or CD31 staining. The analyses were performed on three mice per group, and the points represent the number of field analyzed for each condition.G, HLuminescent MDA‐MB‐436 cells were injected into the brain and monitored by IVIS imaging system. After 1 week from implant, treatment with LNPs‐miR‐Control and LNPs‐miR‐182‐3p was performed as indicated in (A) and (B). Representative images from *in vivo* (upper panel) or *ex‐vivo* (bottom panel) brain tumors are shown in (G). Boxplots (H) show the measurement of photons for each brain tumor (*n* = 5 per group) acquired at the indicated times. Data information: For (A, B, F), data are shown as mean ± SD. For (C, D, H), the line in the middle of the box plot denotes a median value, the limits of box represent the interquartile range (25^th^ to 75^th^ percentiles), while, the whiskers denote the minimum to maximum values. For (A–D) and (H), *P* values are determined by unpaired two‐tailed *t‐*test; for (F), *P* values are determined by Mann–Whitney *t*‐test. Source data are available online for this figure.

Both MDA‐MB‐231 and MDA‐MB‐436, used in the previous experiments, were injected intramuscularly in immunodeficient mice to allow tumor formation. When tumors reached a volume of about 250 mm^3^, mice were randomized, divided into three groups and treated intravenously with empty LNPs (LNPs‐Empty) as negative control, LNPs carrying a non‐targeting miRNA (LNPs‐miR‐Control), or LNPs carrying miR‐182‐3p (LNPs‐miR‐182‐3p) according to a previously described schedule (di Martino *et al*, 2014; Scognamiglio *et al*, 2014), as reported in the upper panel of Fig 5A and B.

While the treatment with LNPs‐miR‐Control did not exert any anti‐tumoral activity, a marked inhibition of tumor growth was observed in mice treated with LNPs‐miR‐182‐3p, reaching about 50% of tumor volume inhibition in MDA‐MB‐231 and even tumor regression in MDA‐MB‐436 (Fig 5A and B). Interestingly, in line with our *in vitro* experiments, BRCA1‐compromised tumors (MDA‐MB‐436) showed an increased sensitivity to LNPs‐miR‐182‐3p treatment compared to HR‐proficient tumors (MDA‐MB‐231).

**Figure EV4:**
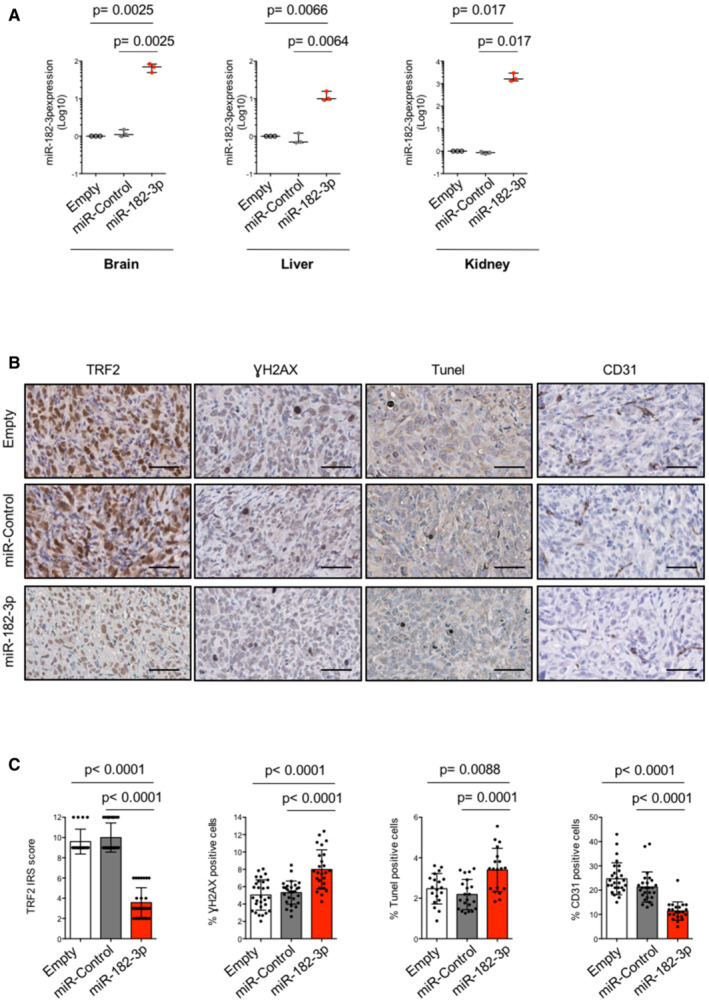
LNPs‐miR‐182‐3p treatment reaches different organs and reduces TRF2 expression in tumor tissue The organs (brain, liver, kidney) taken from mice, previously engrafted with MDA‐MB‐231 cells and treated with LNPs‐empty, LNPs‐miR‐Control or LNPs‐miR‐182‐3p, were assayed for miR‐182‐3p expression by TaqMan qPCR.Representative images show IHC analysis on tumor samples, from mice bearing MDA‐MB‐436 human breast cancer xenografts, with the indicated markers. Scale bar: 50 μm.The histograms show the expression of TRF2 indicated as immunoreactivity score (IRS) and the percentage of positive cells to γH2AX, TIUNEL or CD31 staining in MDA‐MB‐436 xenografts. Three mice per group were analyzed, the points represent the number of field analyzed for each condition. Data information: For (A, C), data are presented as mean values ± SD. Statistical significance using unpaired (A) or Mann–Whitney *t‐*test (C) was calculated. Source data are available online for this figure.

At the end of treatment, mice were sacrificed (day 22) and tumor samples and different organs were excised for *ex‐vivo* analyses. Quantitative PCR analysis revealed the presence of miR‐182‐3p either in the tumors or in the different organs analyzed, including the brain, indicating their ability to pass through the blood–brain barrier (Fig 5C and D and EV4A). Interestingly, pharmacodynamic monitoring of tumor by immunohistochemical analysis (IHC) showed a significant reduction of TRF2 in tumors treated with LNPs‐miR‐182‐3p compared to controls (Figs 5E and F and EV4B and C). In addition, TRF2 modulation by miR‐182‐3p impacted on DNA damage response and apoptosis activation *in vivo*, as shown by a significant increase of γH2AX and TUNEL‐positive cells in tumors treated with LNPs‐miR‐182‐3p compared to controls (Figs 5E and F and EV4B and C). Moreover, according to the pro‐angiogenic role of TRF2 previously demonstrated by our group (Zizza *et al*, 2019), we found that tumors treated with LNPs‐miR‐182‐3p showed a reduced number of vessels (Figs 5E and F and EV4B and C).

Finally, starting from the evidence that LNPs‐miR‐182‐3p cross the blood–brain barrier, we decided to test their efficacy on a model of breast cancer brain metastases by intracranial implantation of luminescent TNBC cells (MDA‐MB‐436) (Soto & Sibson, 2016). One week after cell implantation, mice were treated with LNPs‐miR‐Control or LNPs‐miR‐182‐3p by tail vein injection as previously described (Fig 5A and B). As expected, quantitative bioluminescence imaging revealed that mice treated with LNPs‐miR‐182‐3p show a strong reduction of brain tumor size (day 31) (Fig 5G and H).

Together, these data demonstrate that systemic treatment with miR‐182‐3p embedded in lipid nanoparticles impairs tumor growth in TNBC models by inhibiting TRF2 expression and inducing DNA damage and apoptosis.

### LNPs‐miR‐182‐3p induces tumor inhibition in advanced pre‐clinical models without affecting mouse survival and normal tissue function

Human breast cancers show inter‐ and intra‐tumor heterogeneity that strongly affects response to anticancer therapies (Aparicio & Caldas, 2013). To date, patient‐derived tumor xenografts (PDTXs) and the cell cultures derived from them, named PDTX‐derived tumor cells (PDTCs), maintaining molecular features and heterogeneity of the originating tumor, represent a valid pre‐clinical model to perform anticancer drugs screening (Bruna *et al*, 2016).

**Figure 6:**
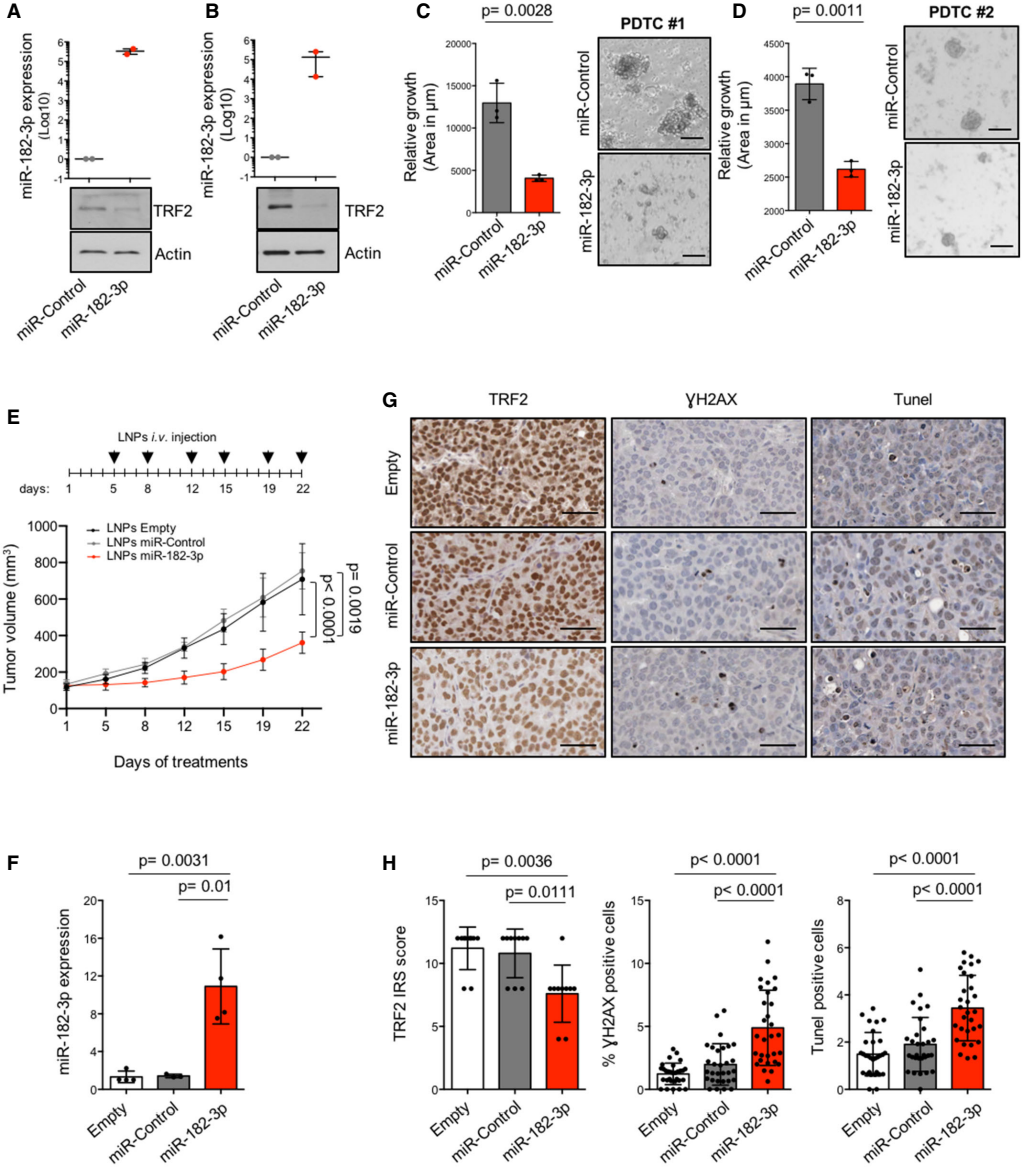
miR‐182‐3p impairs tumor growth by targeting TRF2 in PDTX‐derived tumor cells (PDTCs) and patient‐derived tumor xenografts (PDTXs) A, BPDTCs #1 and #2 underwent two rounds of transfection with miR‐Control or miR‐182‐3p. Three days after the second transfection, miR‐182‐3p and TRF2 expression were analyzed by TaqMan qPCR and western blotting, respectively. Actin was used as loading control.C, DLeft panel, area of each PDTCs was measured by ImageJ. Right panel, representative images are shown. Scale bar: 50 μm. At least 85 3D cells were analyzed for each experimental condition.ENSG mice implanted with breast PDTX (#2) were treated with LNPs‐empty, LNPs‐miR‐Control or LNPs‐miR‐182‐3p as indicated in the scheduling. Caliper measurement of tumors was taken at the indicated days. The mean of tumor volumes (*n* = 5 per group) is shown.FmiR‐182‐3p expression of tumors from mice treated in (E) was assayed by TaqMan qPCR.GRepresentative images of IHC analysis of the indicated markers from tumors of the experiment showed in (E). Scale bar: 50 μm.HThe histograms show the expression levels of TRF2 measured as immunoreactivity score (IRS), the percentage of positive cells to γH2AX and TUNEL. The analysis was performed on three mice per group, the points represent the number of field analyzed for each condition. Data information: For (A–F) and (H), data are shown as mean ± SD. For (A–F), *P* values are determined by unpaired two‐tailed *t‐*test; for (H), *P* values are determined by Mann–Whitney *t*‐test. For the experiments showed in (A, B) and (C, D) two or three biological replicates were performed, respectively. Source data are available online for this figure.

We therefore decided to evaluate the therapeutic potential of miR‐182‐3p in these advanced pre‐clinical models. We first generated short‐term PDTCs (PDTC #1 and #2) from two different triple‐negative breast PDTXs (Bruna *et al*, 2016). One day after cell culture establishment, PDTCs were subjected to transient transfection with miR‐182‐3p and relative miR‐Control. As expected, ectopic expression of miR‐182‐3p, verified by TaqMan qPCR, reduced TRF2 expression and inhibited cell growth not only of PDTC #1 but also of PDTC #2, characterized by BRCA1 germline mutation and resistance to PARP inhibitors (Bruna *et al*, 2016) (Fig 6A–D; Appendix Fig S5A).

Starting from these observations, we tested the efficacy of LNPs‐miR‐182‐3p treatment *in vivo*. For this purpose, PDTXs #2 were subcutaneously implanted in NSG female mice and, when tumors reached a volume of about 150 mm^3^, mice were divided into three groups and treated with LNPs‐Empty, −miR‐Control or –miR‐182‐3p as described above. At the end of the treatments, we found a reduction of ~ 50% of tumor volume in mice treated with LNPs‐miR‐182‐3p, paralleled by high intratumor expression of the miRNA (Fig 6E and F). Moreover, analyses on tumor tissues showed a reduction of TRF2 levels and an increase of γH2AX and TUNEL‐positive cells (Fig 6G and H).

**Figure 7:**
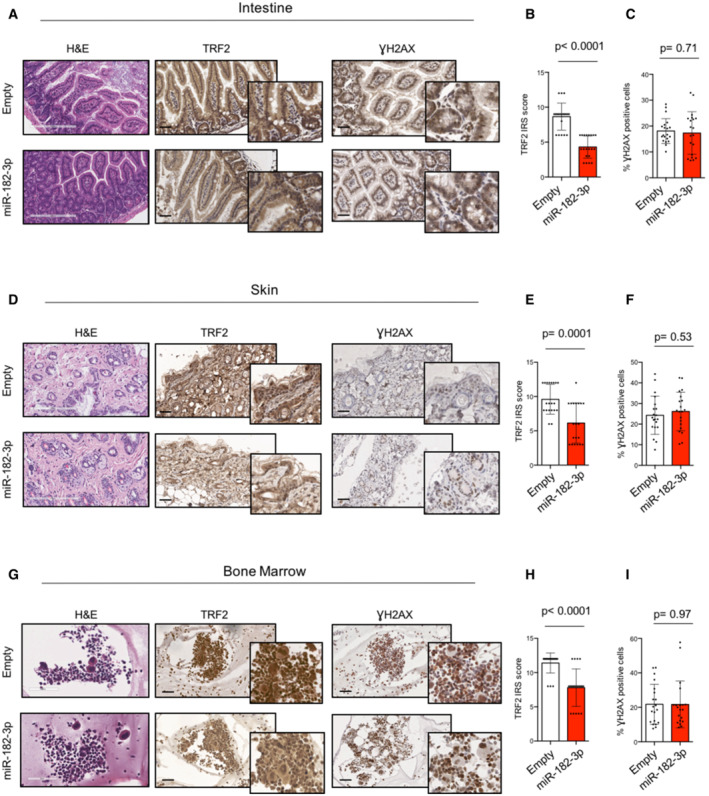
LNPs‐miR‐182‐3p treatment does not cause toxicity or DNA damage in proliferative organs ARepresentative images of intestine sections from mice previously treated with LNPs‐Empty or LNPs‐miR‐182‐3p. H&E staining (scale bar: 200 μm) and IHC analysis with TRF2 or γH2AX antibodies are shown (scale bar: 50 μm).B, CQuantification of TRF2 expression as immunoreactivity score (IRS) (B) and of γH2AX‐positive cells (%) (C) on intestine samples.DRepresentative H&E (scale bar: 200 μm), TRF2 and γH2AX images of skin samples corresponding to LNPs‐Empty or LNPs‐miR‐182‐3p treated animals (scale bar: 50 μm).E, FQuantification of TRF2 expression as immunoreactivity score (IRS) (E) and of γH2AX‐positive cells (%) (F) on skin samples.GRepresentative H&E (scale bar: 200 μm), TRF2 and γH2AX images of bone marrow samples corresponding to LNPs‐Empty or LNPs‐miR‐182‐3p treated animals (scale bar: 50 μm).H, IQuantification of TRF2 expression as immunoreactivity score (IRS) (H) and of γH2AX‐positive cells (%) (I) on bone marrow samples. Data information: For (B, C, E, F, H, I), data are shown as mean ± SD. A Mann–Whitney test *t‐*test was used to calculate statistical significance. Four mice per group were analyzed, the points represent the number of field analyzed for each condition. Source data are available online for this figure.

**Figure EV5:**
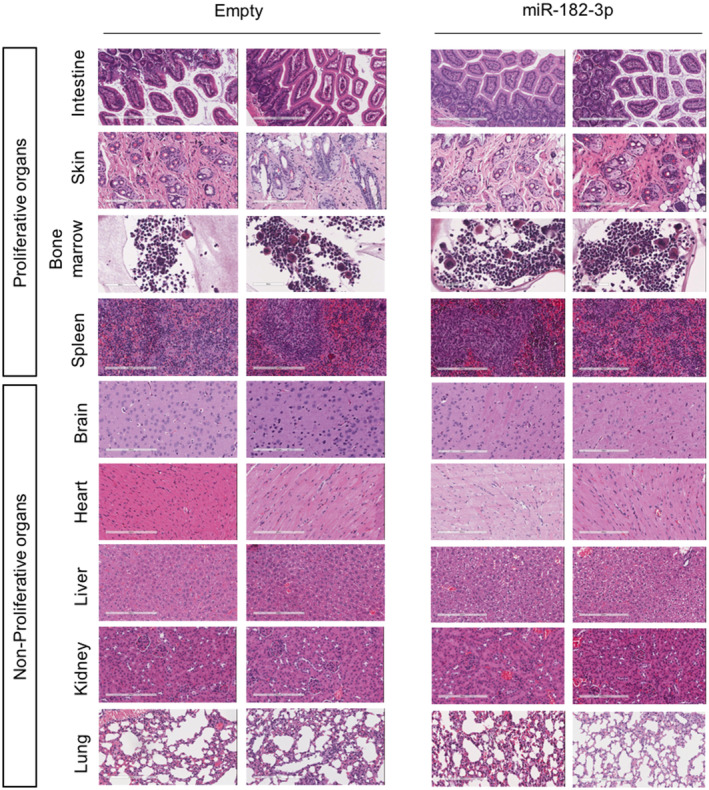
LNPs‐miR‐182‐3p treatment does not compromise tissue viability in proliferative and non‐proliferative organs Proliferative (Intestine, Skin, Bone marrow, Spleen) and non‐proliferative (Brain, Heart, Liver, Kidney, Lung) organs taken from mice, previously treated with LNPs‐empty or LNPs‐miR‐182‐3p, were assayed by hematoxylin and eosin (H&E) staining. Sections from two different mice are shown. Scale bar: 200 μm.

Finally, to validate the therapeutic relevance of TRF2 systemic inhibition by LNPs‐miR‐182‐3p treatment, we evaluated its effects on normal mouse tissues. Histopathological analysis of proliferative and non‐proliferative organs of mice treated with LNPs‐Empty or with LNPs‐miR‐182‐3p did not evidence any lesion neither the presence of aberrant mitotic figures (Figs 7A, D, and G and EV5). In line with this, bone marrow analysis revealed no differences in the morphology of any of the three myeloid lineages (Figs 7G and EV5). Moreover, mice treated with LNPs‐miR‐182‐3p showed a reduced expression of TRF2 in all the proliferative organs analyzed (intestine, bone marrow and skin), not associated with DNA damage (γH2AX) (Fig 7A–I). Finally, IHC of tissues did not show a significant induction of apoptosis (Caspase 3) or senescence (p21, p16 and β‐Gal), neither any relevant change in cell proliferation (Ki67) (Appendix Fig S6A–F). These data indicated that the tumor suppressive activity of miR‐182‐3p is not associated to important adverse effects, supporting the therapeutic potential of TRF2 targeting in cancer.

## Discussion

In the recent years, the advancement in molecular therapy has greatly improved the survival of different types of tumors including breast cancer. However, the targeting of the triple‐negative subtype remains a challenge (Yin *et al*, 2020).

The telomere‐capping factor TRF2 also localizes at non‐telomeric sites and contributes to tumor progression by both telomere‐dependent and independent functions (Blanco *et al*, 2007; Diehl *et al*, 2011; Biroccio *et al*, 2013; Zizza *et al*, 2019). Moreover, TRF2 is highly expressed in various human malignancies, including breast cancer, where its upregulation correlates with reduced recurrent free and overall survival (Cherfils‐Vicini *et al*, 2019; Bhari *et al*, 2021). All these features make TRF2 an interesting therapeutic target in breast cancer.

To date, drug repositioning approaches have led to the identification of two FDA‐approved drugs capable of reducing TRF2 expression (García‐Beccaria *et al*, 2015; Bejarano *et al*, 2017; el Maï *et al*, 2021). However, to the best of our knowledge, strategies to specifically target TRF2 *in vivo* have never been explored so far.

microRNA and small interfering RNA (siRNA) represent today a tool for a next‐generation therapy in various diseases. Besides differences in the mechanism of action between miRNAs and siRNAs, the intrinsic ability of miRNAs to target multiple mRNAs simultaneously allows cooperative anti‐cancer effects compared to siRNAs. To date, four therapeutic siRNAs (Patisiran, Givosiran, Lumasiran, Inclisiran) have been approved by the FDA/EMA and several miRNA‐based approaches have entered Phase II clinical trial (Winkle *et al*, 2021).

miRNAs able to regulate telomere function by targeting TRF1 or hTERT have been previously reported in breast cancer (Dinami *et al*, 2014, 2017). Here, by using the same approach we identified miRNAs able to efficiently modulate TRF2 expression in tumor cells. Of note, miR‐182‐3p turned out to be the most efficient miRNA compared to all the other molecules, including miR‐23a and miR‐490, previously described in the literature (Luo *et al*, 2015; Vinchure *et al*, 2021). Moreover, we found that miR‐182‐3p specifically reduced TRF2 and no other shelterin components such as TRF1 or RAP1. Indeed, targeting of TRF2 by miR‐182‐3p is mediated by a sequence‐specific interplay between the 3′UTR of TRF2 mRNA and the miRNA. Ectopic over‐expression of miR‐182‐3p reduces TRF2 protein levels and its abundance at telomeres in both telomerase and ALT‐positive cancer cells, suggesting that miR‐182‐3p is a general regulator of TRF2 expression.

In line with the role of TRF2 in the maintenance of heterochromatic regions, we showed that miR‐182‐3p triggers DNA damage at telomeric and pericentromeric sites, comparably to TRF2 siRNA. Moreover, accumulation of telomere aberrations and genomic instability was observed upon miR‐182‐3p transfection that may contribute to a strong apoptosis activation in TNBC cells. Of note, the apoptotic effect is exacerbated in cell contexts where HR pathway is impaired (BRCA1 or BRCA2 deficiency), probably due to the accumulation of unresolved DNA damage. Interestingly, as observed in telomerase‐positive cells, miR‐182‐3p was able to induce DNA damage and inhibit cell growth by apoptosis activation also in U2‐OS cells, thus extending the potential use of miR‐182‐3p also for the treatment of ALT cancers. The role of TRF2 on the observed phenotypes was revealed by showing that TRF2 over‐expression significantly attenuates the DNA damage activation, cell growth inhibition and apoptosis, clearly demonstrating that the anti‐tumoral activity of miR‐182‐3p mainly acts through TRF2 targeting.

It is well known that miR‐182 is part of miR‐183/ miR‐182/miR‐96 cluster, which is up‐regulated in most tumors, including breast cancers (Zhang *et al*, 2015; Song *et al*, 2016). Although several studies have demonstrated that miR‐182‐5p over‐expression plays a pro‐tumoral activity in breast cancer, only very few studies take into consideration the role of the specific miR‐182‐3p strand (Cao *et al*, 2018; Zhao *et al*, 2019). In line with our data, Chen *et al* (2019) demonstrated that over‐expression of miR‐182‐3p can inhibit proliferation of osteosarcoma cells by targeting the EBF transcription factor 2. Although our evidence demonstrated a main role of TRF2 targeting in DNA damage and growth inhibition induced by miR‐182‐3p, we cannot completely rule out the eventual contribution of other unknown molecular targets. Indeed, prediction analysis of putative miR‐182‐3p additional targets includes other factors involved in cell proliferation and apoptosis (Table EV2) which are under investigation in a future study.

One of the main challenges in miRNA delivery *in vivo* is to maintain their stability and integrity in the circulation. In this study, we used a formulation of miRNA encapsulated into LNPs, which are considered the most effective and safe miRNA delivery system used in mouse tumor models *in vivo* and also FDA‐approved for the delivery of drugs (Patisiran) or vaccines (anti‐SARS‐CoV2 spike mRNA vaccines; Akinc *et al*, 2019; Vu *et al*, 2021). We found that the intravenous injection of LNPs containing miR‐182‐3p in mice, previously engrafted with two different TNBC cell lines, was capable of significantly reducing tumor growth.

Pharmacodynamic monitoring of the *in vivo* effects induced by administration of LNPs‐miR‐182‐3p revealed that the onco‐suppressive activity of miR‐182‐3p is associated with TRF2 downregulation as well as with an increase of DNA damage/apoptosis. Moreover, miR‐182‐3p appeared to affect also extra‐telomeric functions of TRF2 as demonstrated by reduced angiogenesis at the tumor site. Importantly, we found that synthetic miR‐182‐3p reaches the tumor and various organs including the brain, indicating that LNPs‐miR‐182‐3p cross the blood–brain barrier. Actually, it has been extensively demonstrated that the blood–brain barrier is characterized by an altered permeability in the case of brain tumors (Han & Jiang, 2021), thus allowing the crossing of colloidal particles such as LNPs. This prompted us to test the efficacy of LNPs‐miR‐182‐3p in the treatment of brain metastases, which remain today very difficult to be cured and one of the main causes of death in TNBC patients given the impossibility of most drugs to by‐pass the blood–brain barrier. Interestingly, we observed important anti‐tumoral effects of miR‐182‐3p, suggesting a potential use of this molecule for the treatment of brain metastases.

The therapeutic relevance of LNPs‐miR‐182‐3p was also validated in advanced preclinical models that recapitulate the morphological and molecular characteristics of the originating tumor and represent to date a very useful tool for drugs screening (Bruna *et al*, 2016; Pompili *et al*, 2016). We demonstrated that ectopic introduction of miR‐182‐3p in two different PDTCs can significantly reduce cell growth *in vitro* by targeting TRF2. Moreover, we also showed that LNPs‐miR‐182‐3p treatment is effective *in vivo* on the most aggressive PDTX resistant to PARP inhibitor.

Finally, we investigated the translational relevance of miR‐182‐3p by using primary human fibroblast and immortalized breast epithelial cells. As expected, by reducing TRF2 expression, miR‐182‐3p caused telomeric DNA damage, cell growth inhibition and senescence induction in normal cells. However, the impact of miR‐182‐3p appeared to be less pronounced compared to cancer cells in terms of cell growth inhibition (BJ and MCF10A ~ 35%, cancer cells ~ 55–85%). The increased sensitivity of cancer cells to miR‐182‐3p may be due to different reasons, including the impairment of DNA damage checkpoints, the dependency on TRF2 expression to tolerate genomic instability and/or the possible contribution of other cancer‐relevant miR‐182‐3p targets. The existence of a therapeutic window was observed *in vivo* showing that systemic depletion of TRF2 by LNPs‐miR‐182‐3p did not compromise proliferative and non‐proliferative mouse organs and did not induce a significant activation of DNA damage and apoptosis/senescence in highly proliferative compartments. The lack of a significant DNA damage and/or apoptosis/senescence induction may be due to multiple variables, including the different complexity of the two systems, the doses/scheduling used, and/or the intrinsic selectivity of LNPs for inflamed sites, such as tumor (Zhang *et al*, 2016; Karpuz *et al*, 2021; van Alem *et al*, 2021). However, further studies will be needed to definitely exclude possible side effects *in vivo*.

In conclusion, the data presented here provide proof of concept evidence for the development of a miRNA‐based anti‐cancer therapy against shelterin components, introducing a new specific therapeutic option for TNBC patients.

## Materials and Methods

### Cell lines, culture conditions and transfection

The indicated cancer cell lines were purchased from ATCC: HeLa, HCT116, Saos‐2, U2‐OS, MDA‐MB‐231, MDA‐MB‐436 and BJ. MDA‐MB‐436 luminescent cells (MDA‐MB‐436 LUC) were obtained by infection with the lentiviral vector pRRLSIN.cPPT.RFPL4b.Luciferase.WPRE, that was a gift from Stephen Tapscott (Addgene plasmid # 69252; http://n2t.net/addgene:69252; RRID:Addgene_69252). MDA‐MB‐231 stably over‐expressing TRF2 (pBabe‐puro‐mycTRF2) and the control counterpart (pBabe‐puro‐Empty; Okamoto *et al*, 2013) were obtained by infecting cancer cells with retroviruses generated in amphotropic Phoenix cells previously transfected with the retroviral vectors by JetPEI (Polyplus). MDA‐MB‐231 cells carrying a doxycycline (DOX)‐inducible BRCA2 shRNA were a gift from Tarsounas M. These cells were cultivated in DMEM medium (Sigma) supplemented with 10% tetracycline‐free fetal bovine serum (Clontech). For induction of shBRCA2, 2 μg/ml DOX (D9891, Sigma) was added to growth medium.

All the cell lines (where not indicated) were grown in high‐glucose Dulbecco's Modified Eagle's Medium (DMEM, Invitrogen) supplemented with 10% of fetal bovine serum (Qualified FBS, Gibco), 1% of penicillin–streptomycin and 1% of L‐glutamine at 37°C with 5% CO_2_. All cell lines were regularly tested for mycoplasma contamination.

For transient RNA interference experiments were used miRNA mimics: miR‐182‐3p, miR‐182‐3p inhibitor, miR‐519e‐5p, miR‐296‐3p and miR‐Control (Negative control #1 cat. 4464058) (Ambion); siTRF2 (Dharmacon) and siControl (Santa Cruz Biotechnology). siRNA and miRNA mimics were transfected into cells using INTERFERin (Polyplus) according to the manufacturer's instructions.

### Luciferase reporter screening

Computational target prediction analysis was performed by using three specific softwares (PITA, TargetScan and MicroRNA.org), identifying in‐silico miRNAs with a target specificity for the 3′UTR of TRF2 (Dinami *et al*, 2017). For luciferase reporter assays, the 3′UTR of TRF2 and TRF1 were cloned downstream of Renilla luciferase cassette in the psiCHECK2 vector (Promega). The Firefly reporter gene was used as internal control and to evaluate the transfection efficiency.

High‐throughput luciferase reporter assay was performed in HeLa cells co‐transfected, in a 48 well‐plate, with the 3′UTR‐TRF2 luciferase reporter plasmid (18 ng) and a candidate mimic‐miRNA (final concentration: 50 nM). The screening validation was performed co‐transfecting the mimic miR‐182‐3p (10 nM, Ambion) with the 3′UTR‐TRF2/TRF1 *wild type* or with the mutant of 3′UTR‐TRF2, where the seed sequence for the miR‐182‐3p was deleted (Q5 site‐directed Mutagenesis Kit, NEB). Three days post‐transfection, Renilla/Firefly luciferase reporter activity was assayed using a Dual‐Luciferase Reporter Assay System (Promega) and a GloMax 96 Microplate Luminometer (Promega).

### Protein extracts and Western blotting

Total cell lysates were prepared using a proper buffer (50 mmol/l Tris–HCl pH 7.5, 5 mmol/l EDTA, 250 mmol/l NaCl, 0.1% Triton) containing protease and phosphatase inhibitors (Thermo Fisher Scientific). Samples were sonicated (Diagenode, UCD‐200‐TMEX‐Bioruptor Standard) followed by centrifugation at 16,000 *g* for 2 min. Supernatants were recovered and used for Western blotting analysis. Expression levels of TRF2 were evaluated by using anti‐TRF2 (1:1,000, Millipore, 4A794), anti‐TRF1 (N‐19) (1:1,000, Santa Cruz Biotechnology, Santa Cruz, CA, USA). The DNA damage response was evaluated by using the following antibodies: p‐ATM (Ser1981) (1:1,000, Cell Signaling Technology, Beverly, MA, USA), anti‐ATM (1:1,000, Cell Signaling Technology, Beverly, MA, USA); anti‐γH2AX (Ser139) (1:2,000, Millipore, Bedford, MA). Actin signal was detected by mouse anti‐β‐actin (1:10,000, Sigma Aldrich), and it was used as loading control. The protein band intensity was quantified by densitometry analysis using ImageJ software.

### RNA isolation, qRT‐PCR and TaqMan analysis

Total RNA was isolated from the cell lines using TRIzol reagent (Invitrogen, Carlsbad, CA, USA). The quality and the quantity of RNA extraction was assessed by the *A*_260 nm/_ *A*_280 nm_ and *A*_260 nm/_ *A*_230 nm_ absorbance ratio (Nanodrop 1000, Thermo Fisher Scientific). The mRNA Reverse transcription (RT) was performed using the QuantiTect Reverse Transcription Kit (Qiagen), while miRNA RT was performed by TaqMan™ MicroRNA RT Kit (Applied Biosystems) according to the manufacturer's instructions. mRNA expression was evaluated by SYBR Green (Applied Biosystems) quantitative real‐time PCR (qRT‐PCR) method using a standard protocol. The following primers were used: TRF1 FW‐GCTGTTTGTATGGAAAATGGC; TRF1 RV‐CCGCTGCCTTCATTAGAAAG. TRF2 FW‐CATGCAGGCTTTGCTTGTCA; TRF2 RV‐CTGCATAACCCGCAGCAATC. Actin FW‐AGCACTGTGTTGGCGTACAG; Actin RV‐TCCCTGGAGAAGAGCTACGA. miRNA expression was assayed using TaqMan™ Universal Master Mix II, no UNG with specific TaqMan probes: hsa‐miR‐182*; hsa‐miR‐22; RNU44 (Applied Biosystems). SYBR Green and TaqMan qPCR were run in the QuantStudio 6 Flex Detection system.

### Chromatin immunoprecipitation

ChIP assay was performed as reported in (Petti *et al*, 2019). Briefly, for each condition cells were fixed at 72 h after miRNA transfection with 1% of formaldehyde. Nuclei were isolated by using a Dounce homogenizer and lysed with SDS lysis buffer. Next, the lysates were sonicated to generate fragments averaging 0.5–1 kb (Diagenode Bioruptor Inc., NXT‐Dx Belgium). For each immunoprecipitation condition, 100 μg of chromatin and 4 μg of the indicated antibodies were used. The antibody used for the immunoprecipitation is the rabbit anti‐TRF2 (NB110‐57130, Novus) and IgG Rabbit (Bethyl) were used as negative control. After precipitation, the DNA was extracted from the immuno‐complexes and blotted onto Hybond‐N membrane (Amersham), and telomeric repeat sequences were detected through hybridization with a TTAGGG probe. A nonspecific probe (Alu) was also used. To verify that an equivalent amount of chromatin was used in the immunoprecipitates, samples representing the 1 and 0.1% of the total chromatin (input) were included in the telo‐blot. The filter was exposed to a PhosphorImager screen (Bio‐Rad), and the signals were measured using ImageJ software.

### Immunofluorescence

Cells were fixed in 4% formaldehyde in 1× phosphate‐buffered saline (1× PBS) for 15 min at room temperature (RT) and next permeabilized by treatment with 0.5% Triton X‐100, 0.1% Na‐Citrate (1× PBS) for 5 min at RT. Cells were blocked for 1 h in 3% BSA, 0.1% Tween‐20 (1× PBS) and washed twice with PBS. For immune labeling, cells were incubated with mouse anti‐TRF2 (1:2,000, Millipore, 4A794) antibody in 3% BSA, 0.1% Tween‐20 (1× PBS) for 1 h at RT. Next, cells were washed with 0.3% BSA, 0.1% Tween‐20 (1× PBS) and incubated for 1 h with Anti‐Mouse Alexa Fluor 555 Conjugate (1:500, Cell Signaling) antibody. At least, nuclei were stained with 4′,6‐diamidino‐2‐phenylindole (DAPI, Sigma). Fluorescence signals were acquired by using the Leica DMi8 microscope equipped with the Leica DFC 350FX camera and acquired by Leica application suite X (LasX) software (Leica, Solms, Germany). For telomere fluorescence evaluation, 90 nuclei for each sample in triplicate were captured at 63× magnification, and the telomere fluorescence was quantified by using spot IOD (integrated optical density) analysis using the TFL‐TELO software.

### Immunofluorescence combined with DNA FISH

For immunofluorescence, cells were fixed in 4% formaldehyde (1× PBS) followed by permeabilization with 0.1% Triton X‐100 in (1× PBS) for 7 min at RT. Cells were blocked for 1 h in 3% BSA (1× PBS) and incubated overnight (O.N.) with a mouse antibody anti‐phospho‐Histone H2A.X (Ser139) (1:300, Millipore, Bedford, MA) or with mouse anti‐TRF2 (1:2,000, Millipore, 4A794) antibody. After incubation with secondary antibody, cells were fixed in 4% formaldehyde (1× PBS) and subjected to standard telomere DNA FISH. Fluorescence analysis was performed by confocal laser scanning microscopy using Zeiss LSM 880 Airyscan (Zeiss, Germany). Nuclei showing co‐localization of γH2AX with telomeric probe (Cy3‐labeled TelC PNA Probe, PANAGENE) were analyzed as TIFs positive, while at least one co‐localization with SatIII pericentromeric probe (Cy3‐labeled SatIII PNA probe, PANAGENE) was considered as PIFs positive.

### Telomere DNA FISH

DNA‐FISH was carried out as previously described (Petti *et al*, 2019). Cells were fixed with 4% paraformaldehyde for 10 min at room temperature (RT). The slides were washed three times with 1× PBS for 5 min and next were dehydrated by washing in 70, 90, and 100% ethanol for 5 min each. Slides were allowed to dry for 10 min at RT. The slides were denatured at 80°C for 3 min after adding the Cy3‐labeled (CCCTAA)_3_ probe and next were incubated for 2 h at RT in a humid chamber. Subsequently, the slides were washed under agitation, twice with FISH solution (70% formamide, 10 mM Tris pH 7.2, 0.1% BSA) for 15 min at RT and three times with 0.08% Tween‐20 in 1× TBS at RT. Slides were then dehydrated with washes in 70, 90 and 100% ethanol. Samples where mounted with Vectashield (Vector laboratories). Interphase nuclei were analyzed using spot IOD analysis (TFL‐TELO) software. A Mann–Whitney *t*‐test was used to calculate statistical significance.

For telomere aberrations analysis, metaphases were prepared as previously described (Canela *et al*, 2007) and samples were processed as described for DNA‐FISH.

### Cell proliferation

Cells were seeded 1 × 10^5^ per well and 24 h after were transfected with 10 nM of miR‐Control, miR‐182‐3p or miR‐182‐3p‐inhibitor (miR‐182‐3p‐i). After 72 h, cells were harvested, counted and seeded again to perform the second transfection cycle. Cell growth was monitored by Incucyte® S3 Live‐Cell Analysis System (Essen BioScience, Ann Arbor, MI). Images were automatically captured every 24 h under a phase‐contrast microscope (10× magnification). Viability was assessed by comparing the cell confluence between groups using IncuCyte S3 Image Analysis Software (Essen Bioscience).

### Cell senescence

Senescence was evaluated performing the β‐galactosidase staining‐based assay. Cells were fixed with formaldehyde 2% for 10 min at RT, washed twice with 1× PBS (pH 7.0) and incubated at 37°C O.N. in a dry incubator (without CO_2_) in staining solution: 1 mg/ml X‐gal (5‐bromo‐4‐chloro‐3‐indolyl‐β‐D‐galactoside), 5 mM ferric ferrocyanide (II), 5 mM ferrous ferricyanide (III), 1 mM MgCl_2_ dissolved in 1× PBS pH 6.0. Nuclei were analyzed using a phase‐contrast microscope by 40× magnification. At least 10 fields were analyzed for each plate.

### Enzyme‐linked immunosorbent assay (ELISA)

To evaluate senescence‐associated secretory phenotype (SASP) the amount of IL‐6, IL‐8 and CXCL1 was measured. In detail, BJ cells were incubated with 2 ml of serum‐free medium for 24 h, after the second round of transient transfection with the indicated miRNAs. Next, the medium was collected, centrifuged to remove cellular debris and assayed by ELISA (Quantikine ELISA, R&D systems), according to the manufacturer's instructions. Results were normalized to cell number.

### Flow cytometry

For the analysis of cell progression through the cell cycle phases, cells were harvested, washed with 1× PBS twice and fixed in 70% ethanol at 4°C O.N. Next, cells were washed with 1× PBS and stained with propidium iodide (PI; Sigma Aldrich) at a final concentration of 50 mg/ml and RNase at a final concentration of 75 kU/ml, incubated for 30 min as previously described (Zizza *et al*, 2019). The flow cytometry (Becton‐Dickinson) after staining with PI was analyzed by FACSCelesta (BD Biosciences, San Jose, CA, USA). Simultaneously to cell cycle analysis, the DNA and 5‐bromo‐2′‐deoxyuridine (BrdU) contents of cells was measured as previously described (Biroccio *et al*, 2001). In brief, cells were pulsed with 20 μM of BrdU (Sigma Aldrich) for 15 min, and then the DNA was denatured. Thus, cells were incubated with 20 μl of the mouse Mab‐BrdU (347580 Pure BD) for 1 h at RT, and the detection of BrdU‐labeled cells was permitted by using the anti‐mouse Alexa Fluor 488 (Cell Signaling). Finally, cells were counterstained with PI, acquired and analyzed. As negative control, cells without BrdU incorporation were used.

The apoptosis evaluation was conducted following the annexin V assay versus PI staining, as previously described (Biroccio *et al*, 2002). In brief, cells were harvested and suspended in annexin‐binding buffer (final concentration of 1 × 10^6^ cells/ml). Thus, cells were incubated with fluorescein isothiocyanate‐annexin V (annexin‐FITC) and PI for 15 min at RT away from the light and immediately analyzed by using FACSCelesta (BD Biosciences, San Jose, CA, USA). For each condition, 20,000 events were measured and obtained data were analyzed with FACS Diva Software (BD Biosciences).

### Preparation of LNPs encapsulating miRNAs

Lipid nanoparticle formulations, empty or encapsulating miRNA, were prepared by ethanol injection method as previously described (Fattore *et al*, 2020). Thus, a lipid stock solution of DSPC/CHOL/DODAP/PEG_2000_‐Cer_16_ (25/45/20/10 w/w) was prepared in ethanol (40% v/v of total preparation). Then, an aliquot (0.2 mg) of miR‐Control or miR‐182‐3p was dissolved in citric acid (20 mM, pH 4.0). The two solutions were warmed at 65°C and then the lipid solution was added drop by drop to the miRNA solution under stirring. The preparation was sized through 200 and 100 nm polycarbonate filters using a thermobarrel extruder (Northern Lipids Inc., Vancouver, BC, Canada) at 65°C. Therefore, the preparation was dialyzed (3.5 kDa cutoff) against citrate buffer (20 mM, pH 4.0) for approximately 1 h to remove excess of ethanol and then against HBS (20 mM HEPES, 145 mM NaCl, pH 7.4) for 12–18 h to remove citrate buffer and to neutralize the LNP surface. The amount of miRNA not encapsulated in LNPs was removed by ultracentrifugation at 278,835 ***g*** for 40 min (Optima Max E, Beckman Coulter, USA; rotor TLA 120.2).

1,2‐dioleyl‐3‐dimethylammonium propane (DODAP) and N‐palmitoyl‐sphingosine‐1‐{succinyl[methoxy(polyethylene glycol)2000]} (PEG_2000_‐Cer_16_) were purchased by Avanti Polar Lipids. Distearoylphosphatidylcholine (DSPC) was kindly offered from Lipoid GmbH (Cam, Switzerland). Cholesterol (CHOL), sodium chloride, sodium phosphate, HEPES, citric acid and sodium citrate were purchased by Sigma Aldrich (USA). Ethanol and other solvents were obtained by Exacta Optech (Italy).

### Characterization of lipid nanoparticles (LNPs)

LNP size, particle size distribution (PI) and zeta potential (ZP) were measured by dynamic light scattering with Zetasizer Ultra (Malvern Instruments, Worcestershire, UK). Thus, samples were diluted 1:100 v/v with 0.22 μm filtered water and analyzed. Results were obtained by the average of the measures on three different batches of the same formulation. To measure the amount of miRNA encapsulated, the formulations were dissolved in methanol (1:100 v/v), and samples were centrifuged (for 30 min at 16,250 ***g***; MIKRO 20; Hettich, Tuttlingen, Germany). Supernatants were then analyzed by spectrophotometer at 260 nm. miRNA encapsulation efficiency (EE%) was calculated as % ratio between miRNA actual loading (mg of miRNA/mg of total lipids) and miRNA theorical loading in formulation. Results, calculated as the mean of the measures obtained on three different batches, are summarized in Table 1.

RNA sequences used for LNPs production are the following:

miR‐Control: UCACAACCUCCUAGAAAGAGUAGA;

miR‐182‐3p: UGGUUCUAGACUUGCCAACUA

### *In vivo* xenograft experiments

All animal procedures were in compliance with the national and international directives (D.L. March 4, 2014, no. 26; directive 2010/63/EU of the European Parliament and European Council; Guide for the Care and Use of Laboratory Animals, U.S. National Research Council, 2011; Animal Research guidelines Reporting of *In Vivo* Experiments [ARRIVE] guidelines) and approved by the Italian Ministry of Health (authorization n. 607/2019‐PR, released on 07‐08‐2019). Mice were maintained in a barrier facility on high‐efficiency particulate air HEPA‐filtered racks and received food and water *ad libitum*.

CB17‐SCID (CB17/Icr‐Prkdcscid/IcrIcoCrl, 6 weeks old) female mice (Charles River Laboratories, Calco, Italy) were injected intramuscularly with 4 × 10^6^ of MDA‐MB‐231 or 4 × 10^6^ of MDA‐MB‐436 cells. When tumors reached about 250 mm^3^, animals were randomized to start the treatments. Mice were treated intravenously (*iv*) with LNPs‐Empty, LNPs‐miR‐Control and LNPs‐miR‐182‐3p at 20 μg/mouse at days 5;8;12;15;19;22. Tumor volumes were measured in two dimensions using a caliper and calculated by the formula a × b^2^/2, where “a” and “b” are the long and short sizes of the tumor, respectively. Each experimental group included five mice.

Athymic Nude female mice (Envigo, San Pietro al Natisone (UD), Italy) were anesthetized and injected intracranially with 1.8 × 10^5^ MDA‐MB‐436 LUC cells/mouse, through the middle area of the frontal bone to a 2‐mm depth, using a 0.1‐ml glass micro‐syringe and a 27‐gauge disposable needle. The mice were weighed and medicated with an oral administration of 0.5 mg/kg of Metacam (meloxicam) to control for post‐operative pain and inflammation. The medication was carried out until the end of the experiment. Animals will be closely monitored by visual inspection and weighed daily.

Thus, 6 days post‐injection, mice were randomized, divided into two groups and treated *iv* with LNPs‐miR‐Control and LNPs‐miR‐182‐3p at 20 μg/mouse at days 5;8;12;15;19;22. Tumor growth was monitored by optical IVIS Imaging System 200 series. Briefly, mice were anesthetized with a combination of tiletamine–zolazepam (Telazol, Virbac, Carros, France) and xylazine (xylazine/Rompun BAYER) intramuscularly at 2 mg/kg. Next, mice were injected intraperitoneally with 150 mg/kg D‐luciferin (Caliper Life Sciences) and imaged 10 min after luciferin injection. Imaging was performed at the day of tumor cell injection (baseline) and at days 21 and 31 during the experiment. Data were acquired and analyzed using the living image software version 4.3 (Caliper Life Sciences). Each experimental group included five mice.

To generate patient‐derived tumor xenografts (PDTXs), tumor fragments (15–20 mm^3^) derived from VHI0179, a BRCA1 mutated breast tumor, were coated in Matrigel (Corning) and implanted by a small incision in a subcutaneous pocket made in one side of the lower back into NOD.Cg‐Prkdc^SCID^ IL‐2R null (NSG) female mice (Charles River Laboratories, Calco, Italy). When the tumor reached approximately 200 mm^3^, mice were randomly divided in three groups and treated *iv* with LNPs‐Empty, LNPs‐miR‐Control and LNPs‐miR‐182‐3p at 20 μg per mouse at days 5;8;12;15;19;22. Each experimental group included six mice.

At the end of treatments, mice were sacrificed and tumors and organs were excised for histologic and TaqMan qPCR analysis.

### Histological analysis

Formalin‐fixed and paraffin‐embedded tissue blocks from tumors and major organs were sectioned (2 μm) and stained with hematoxylin and eosin while, for immunostaining, sections were deparaffinized, rehydrated and subjected to antigen retrieval by PT Link (Dako Omnis). Endogenous peroxidase was blocked for 10 min with Peroxidase‐Blocking Solution (Dako Omnis), and then, non‐specific antibody binding was blocked for 20 min with Protein blocking buffer (Dako Omnis). Sections were immunostained with following primary antibodies: anti‐TRF2 mouse monoclonal (Novus Biologicals, 4A794.15, 1:500), anti‐TRF2 rabbit polyclonal (Novus Biologicals, 1:500), anti‐γH2AX rabbit monoclonal (Bethyl Laboratories, BLR059F, 1:500), anti‐γH2AX rabbit monoclonal (AbCam, EP854(2)Y, 1:500), anti‐CD31 rat monoclonal (Dianova, SZ31, 1:10), anti‐Caspase‐3 rabbit polyclonal (Cell Signaling, 1:100), anti‐β‐Gal rabbit polyclonal (Invitrogen, 1:200), anti‐p16INK4a rabbit polyclonal (Invitrogen, 1:200), anti‐p21 rabbit recombinant monoclonal (Invitrogen, NP_000380.1, 1:100), anti‐Ki67 rabbit polyclonal (AbCam, 1:100). Tissue sections were covered for 30 min with Dako EnVision™ FLEX/HRP (EnVision™ FLEX Dako Omnis) or with goat anti‐rabbit Ig/HRP (Agilent, 1:100) for the detection of Ki67 on mouse tissues. Detection of apoptotic cells by terminal deoxytransferase‐mediated deoxy uridine nick end‐labeling (TUNEL) assay was carried out using In Situ Cell Death Detection POD Kit (Roche Molecular Biochemicals). Signal was developed by using DAB (EnVision™ FLEX Dako Omnis). Images were acquired with Aperio SCANSCOPE CS SYSTEM, and immunostaining results were evaluated as percentage of positive cells or immunoreactive score (IRS) (Fedchenko & Reifenrath, 2014).

### PDX‐derived tumor cells (PDTC)

Short‐term PDTC culture was obtained from two patient‐derived tumor xenografts (PDTX #1 STG282; PDTX #2 VHIO179) as described by Bruna *et al* (2016). Briefly, fresh patient‐derived tumor xenografts (PDTXs) were thawed and dissociated, to obtain a single‐cell suspension, by combining mechanical and enzymatic dissociation. The protocol followed for the soft tumor dissociation was performed using GentleMACS Dissociator and the human tumor dissociation kit (Miltenyi Biotec, Cat ID 130‐093‐235) according to the manufacturer's instructions. Single cells were plated at density of 2 × 10^5^ cells (six‐well plates) and treated after 24 h with mimic miRNAs for two transfection cycles. The inhibition of cell growth was measured by ImageJ as the reduction of area.

### Statistics

Statistical analyses were performed using GraphPad Prism 6 software. The statistics test applied for each experiment is reported in the figure legend. The *P* values are indicated in the figures.

## Author contributions

**Roberto Dinami:** Formal analysis; validation; investigation; visualization; methodology; writing—original draft; writing—review and editing. **Luca Pompili:** Formal analysis; validation; investigation; methodology. **Eleonora Petti:** Formal analysis; validation; investigation; visualization; methodology; writing—original draft; writing—review and editing. **Manuela Porru:** Formal analysis; investigation; methodology. **Carmen D'Angelo:** investigation; methodology. **Serena Di Vito:** Investigation; methodology. **Angela Rizzo:** Investigation; methodology. **Virginia Campani:** Investigation; methodology. **Giuseppe De Rosa:** Methodology; supervision. **Alejandra Bruna:** Resources; methodology. **Violeta Serra:** Resources; methodology. **Miguel Mano:** Formal analysis; investigation; methodology. **Mauro Giacca:** Methodology; supervision. **Carlo Leonetti:** Investigation; methodology. **Gennaro Ciliberto:** Supervision. **Madalena Tarsounas:** Resources; supervision. **Antonella Stoppacciaro:** Supervision; methodology. **Stefan Schoeftner:** Conceptualization; resources; investigation. **Annamaria Biroccio:** Conceptualization; supervision; funding acquisition; investigation; methodology; writing—original draft; writing—review and editing.

## Disclosure and competing interests statement

The authors declare that they have no conflict of interest.

## Supporting information



Appendix

Expanded View Figures PDF

Table EV1

Table EV2

Source Data for Expanded View

PDF+

Source Data for Figure 1

Source Data for Figure 2

Source Data for Figure 3

Source Data for Figure 4

Source Data for Figure 5

Source Data for Figure 6

Source Data for Figure 7

## Data Availability

This study includes no data deposited in external repositories.
