# Supplementary material for: MiR‐182‐3p targets TRF2 and impairs tumor growth of triple‐negative breast cancer
Source: EMBO Mol Med. 2022 Nov 25;15(1):e16033. doi: 10.15252/emmm.202216033 (PMC9832842; doi:10.15252/emmm.202216033)
Supplement: Supplementary file 1 — Appendix [file EMMM-15-e16033-s009.pdf]

MiR-182-3p targets TRF2 and impairs tumor growth of triple negative breast cancer

Table of contexts

Appendix Figure S1
Appendix Figure S2
Appendix Figure S3
Appendix Figure S4
Appendix Figure S5
Appendix Figure S6

Appendix Figure S1 - miR-182-3p over-expression induces global and telomeric DNA damage activation in ALT-positive cells

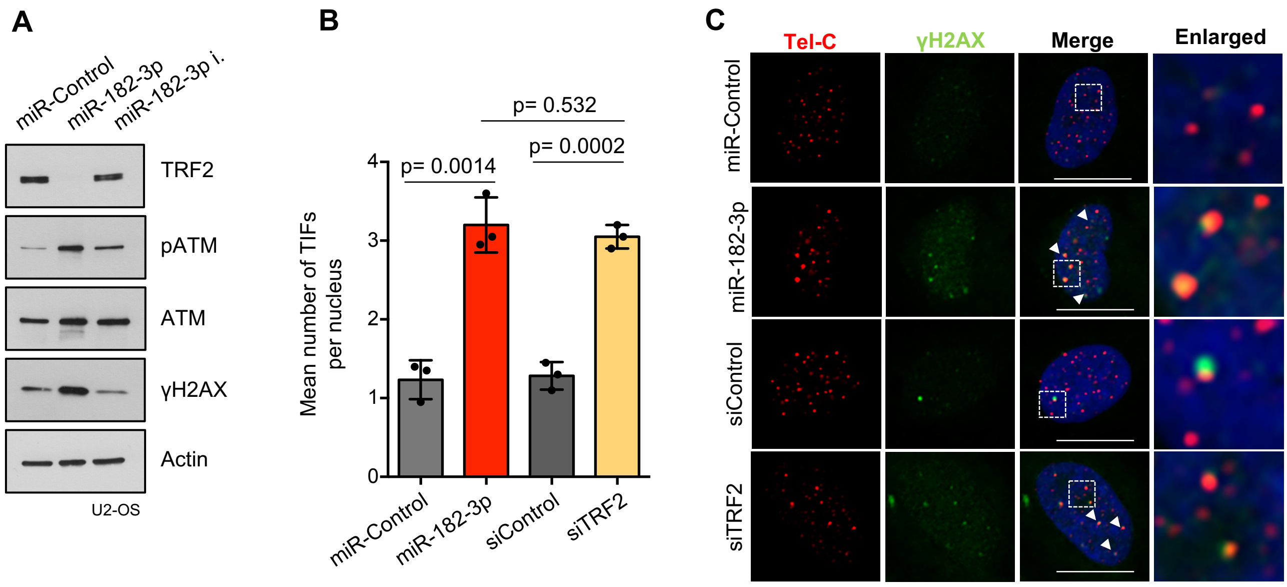

A. U2-OS cells were transiently transfected with the indicated miRNAs and, three days post-transfection the indicated DNA damage markers were assayed by western blotting. Actin was used as loading control.
B. Immunofluorescence analysis of γH2AX combined with a telomeric FISH probe (TIFs) was performed in U2-OS cells 72h post-transfection with the indicated miRNAs and siRNAs. Co-localizations of γH2AX with telomeres are indicated as mean number of TIFs per nucleus.
C. Representative images of co-localizations of the experiment described in B.

Data information: For B, data are presented as mean values ± SD. Three independent replicates were performed. At least 60 nuclei per condition were analysed. Unpaired two-tailed *t* test was used to calculate statistical significance.

Appendix Figure S2 - Synthetic miR-182-3p over-expression does not impact on cell senescence but alters cell cycle progression

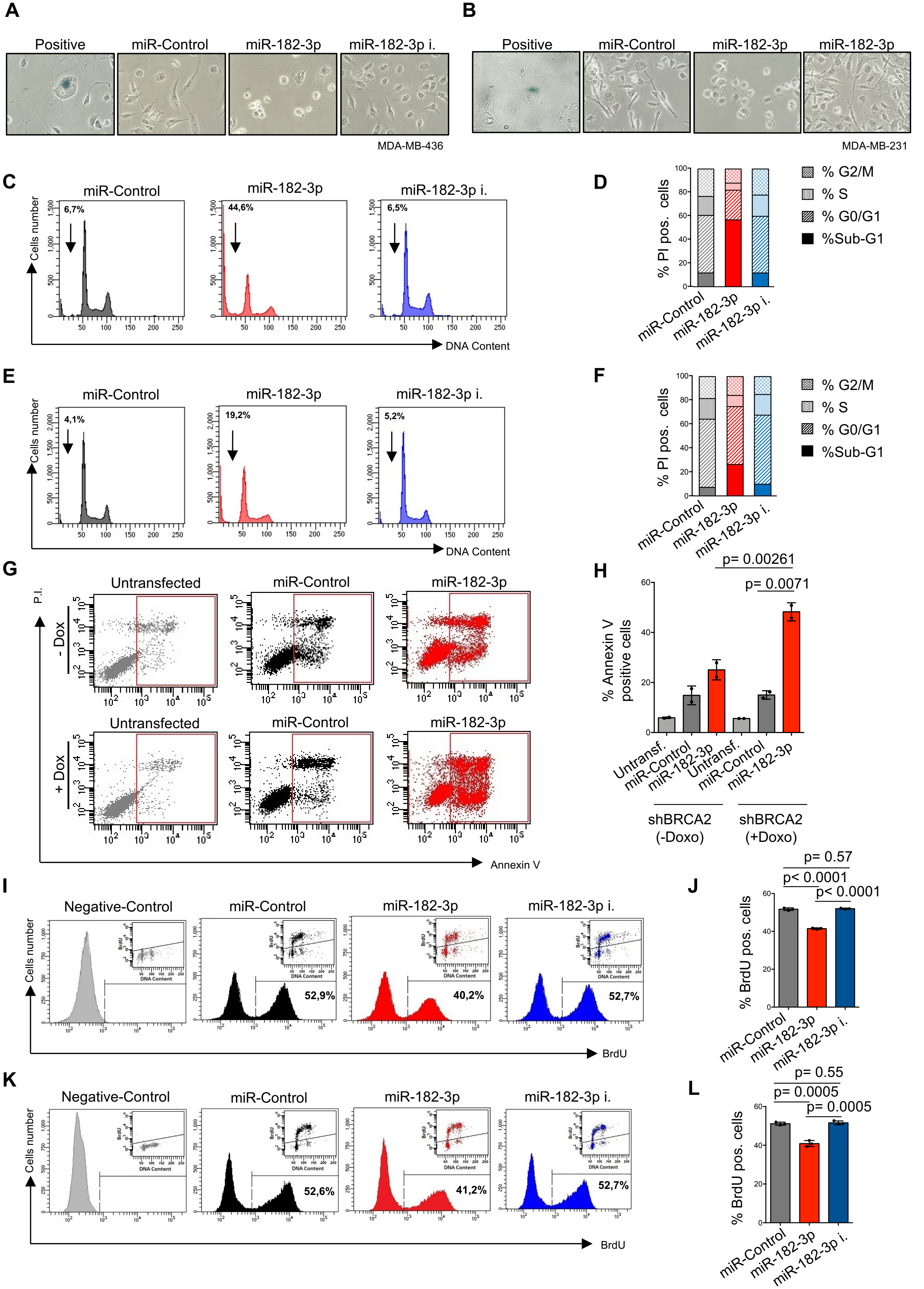

A, B. MDA-MB-436 and MDA-MB-231 cells transiently transfected with the mimic miR-Control, miR- 182-3p or miR-182-3p inhibitor were tested by b-Galactosidase assay. RHPS4 treatment (10μM 72hrs) was used as positive control of the assay.
C-F. FACS analysis to evaluate cell cycle progression by Propidium Iodide (PI) staining was performed in MDA-MB-436 (C) and MDA-MB-231 (E). Quantification of the percentage of cells in the different phases of the cell cycle (D, F).
G. Two-dimensional scatter plots of Annexin V analysis performed in MDA-MB-231 carrying a doxycycline (DOX)-inducible BRCA2 shRNA cassette, at the end of the second cycle of transfection with miR-Control or miR-182-3p. Untransfected cells were used as negative control of assay. Red boxes indicate early and late apoptotic cells.
H. Quantification of Annexin V positive cells (%) of experiment described in G. Two independent experiments were performed.
I-L. Monoparametric analysis of Bromodeoxyuridine (BrdU) incorporation performed by flow cytometry analysis in MDA-MB-436 (G) and MDA-MB-231 (I). Left and right peaks in each graph represent the BrdU negative and positive cells, respectively, selected on the basis of the negative control (grey peaks). The biparametric analysis of BrdU and DNA content is reported inside each graph. Quantification of the percentage of BrdU positive cells (J, L). Three independent experiments were performed.

Data information: For H,J,L, data are presented as mean values ± SD. Unpaired two-tailed *t* test was used to calculate statistical significance.

Appendix Figure S3 - Synthetic miR-182-3p over-expression induces telomere loss, sister chromatid fusions and genomic instability in TNBC cells

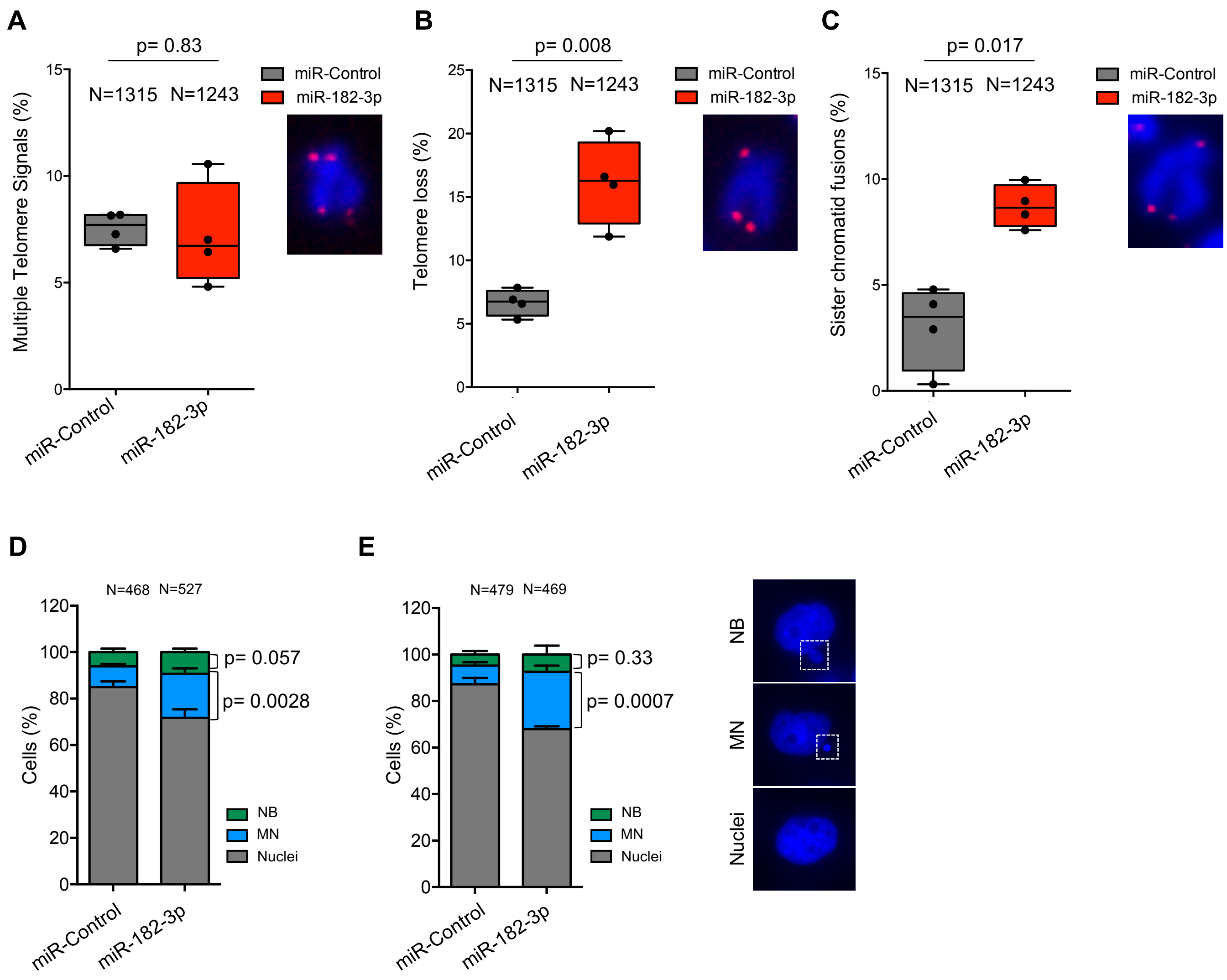

A-C. Telomeric DNA FISH on metaphase spreads was performed in MDA-MB-436 cells transiently transfected with miR-182-3p and miR-Control. Multiple telomere signals (A), telomere loss (B) and sister chromatid fusions (C) were analysed. N= number of analysed chromosomes.
D,E. Micronuclei (MN) and Nuclear Budding (NB) were analysed in MDA-MB-436 cells upon one (D) or two (E) cycles of transfection with the indicated miRNAs. N= number of analysed cells.

Data information: Unpaired two-tailed *t* test was used to calculate statistical significance.

Appendix Figure S4 - Ectopic miR-182-3p over-expression reduces cell growth by apoptosis activation in ALT-positive cells

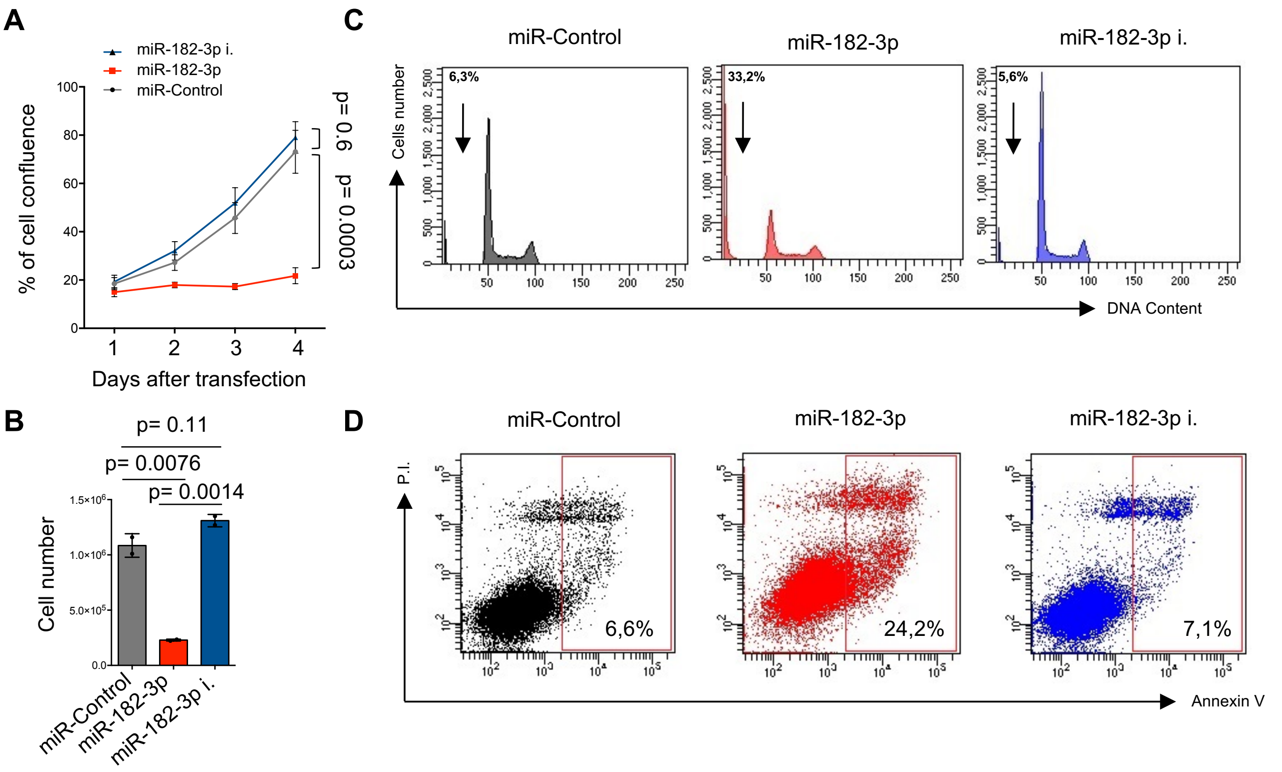

A,B. U2-OS cells underwent two rounds of transfection with miR-Control, miR-182-3p or miR-182-3p inhibitor. Starting from the day of the second transfection, cell confluence was monitored by Incucyte every 24h up to a maximum of 3 days. The percentage of cell confluence was analysed in A. Cells number (B) was analysed at the end of the experiment showed in A.

C. FACS analysis by Propidium Iodide (PI) staining was performed in U2-OS cells transfected with the indicated miRNAs. The percentage of cells in sub-G1 is indicated.

D. U2-OS cells transfected with the indicated miRNAs were assayed by Annexin V. The percentage of apoptotic cells is shown.

Data information: For A and B, data are presented as mean values ± SD. Unpaired two-tailed *t* test was used to calculate statistical significance.

Appendix Figure S5 - PDTCs #2 is resistant to Olaparib

A

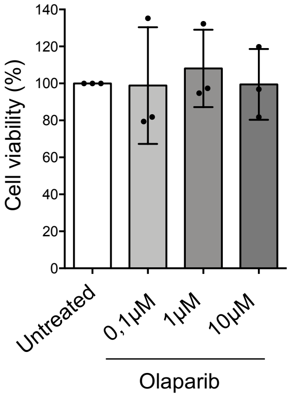

A. PDTCs #2, indicated as resistant to PARP inhibitors (Bruna *et al,* 2016), was treated with different doses of Olaparib. The luminescent cell viability was measured by using CellTiter-Glo. Data are presented as mean values ± SD.

Appendix Figure S6 – LNPs-miR-182-3p does not induce apoptosis and senescence in proliferative organs

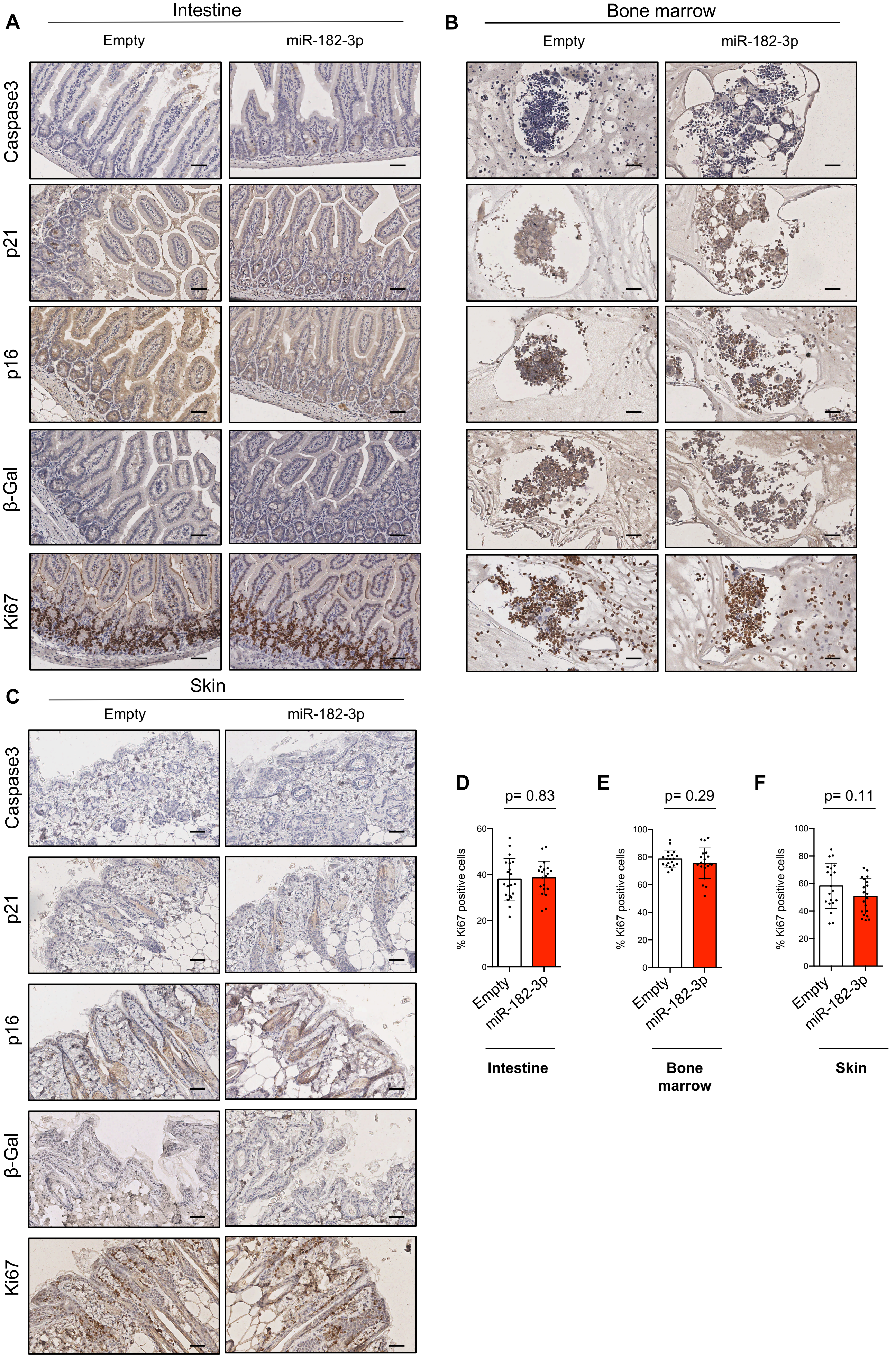

A-C. Representative images of intestine (A), bone marrow (B) and skin (C) samples from mice previously treated with LNPs-empty or LNPs-miR-182-3p. IHC analysis with Caspase 3, p21, p16, β-Gal and Ki67 antibodies are shown. Positive cells, for Caspase 3, p21, p16, β-Gal markers, were <1% per field in both conditions. Scale bar: 50μm.
D-F. Quantification of Ki67 positive cells (%) in intestine (D), bone marrow (E) and skin (F) samples. The analysis was performed on four mice per group. Mann-Whitney *t* test was used to calculate statistical significance.
