# Supplementary material for: MiR‐182‐3p targets TRF2 and impairs tumor growth of triple‐negative breast cancer
Source: EMBO Mol Med. 2022 Nov 25;15(1):e16033. doi: 10.15252/emmm.202216033 (PMC9832842; doi:10.15252/emmm.202216033)
Supplement: Supplementary file 2 — Expanded View Figures PDF [file EMMM-15-e16033-s008.pdf]

Expanded View Figures

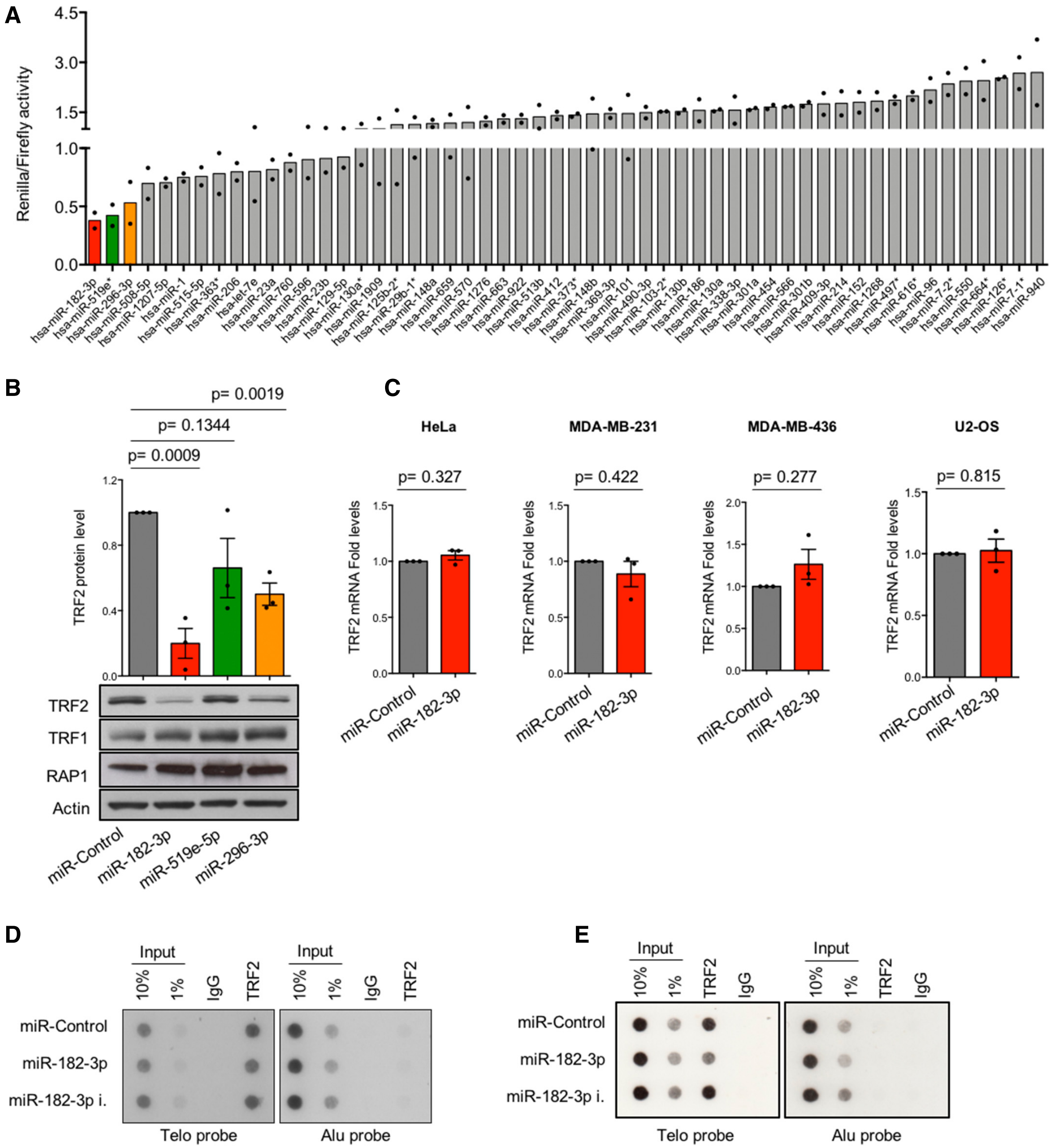

Telo Probe	IgG	TRF2
miR-Control	0,70	11,27
miR-182-3p	0,78	8,8
miR-182-3p i.	0,75	10,39

MDA-MB-231

Telo Probe	TRF2	IgG
miR-Control	7,87	0
miR-182-3p	4,53	0,0017
miR-182-3p i.	8,19	0,0013

U2-OS

Figure EV1.

© 2022 The Authors EMBO Molecular Medicine 15: e16033 | 2023 21 of 20

Figure EV1. High-throughput luciferase miRNAs screening identifies miR-182-3p as the most efficient miRNA able to target TRF2.

A Results of high-throughput luciferase screening performed in Hela cells using the wild type 3′UTR-TRF2 vector in combination with each of the 54 miRNAs selected by *in silico* analysis. Three days post-transfection, luciferase ratio (Renilla:Firefly) of each miRNA was calculated, the control miRNA was set "1." Renilla:Firefly ratios < 1 indicate target specificity of candidate miRNAs for the 3′UTR of TRF2. miRNAs near to the ratio of 0.5 were considered for further analysis. Two biological replicates were performed.

B HeLa cells transiently transfected with the indicated miRNAs (miR-Control, miR-182-3p, miR-519e-5p, miR-296-3p) were assayed by western blotting. Upper panel, quantification of TRF2 expression. Bottom panel, representative images of TRF2, TRF1 and RAP1 are shown, actin was used as loading control.

C Analysis of TRF2 mRNA expression performed by qPCR in four different cancer cell lines (HeLa, MDA-MB-231, MDA-MB-436, U2-OS) 3 days post-transfection with miR-Control or miR-182-3p. The control miRNA was set "1." Three independent experiments were performed.

D, E Telomeric ChIP assay in MDA-MB-231 (D) and U2-OS cells (E). Quantification of TRF2 enrichment at telomeric repeats, in the different conditions, is shown in the table under the respective figure. Alu probe and Rabbit IgG were used as negative control for the assay.

Data information: For (A), data are presented as mean values. For (B, C), data are presented as mean values ± SD and Student *t*-test was used to calculate statistical significance.
Source data are available online for this figure.

Figure EV2. Silencing of TRF2 induces telomeric, pericentromeric and global DNA damage activation.

A MDA-MB-231 cells were transiently transfected with the indicated miRNAs or siRNA. The indicated DNA damage markers were assayed by western blotting. Actin was used as loading control.

B Telomeric DNA FISH performed in MDA-MB-231 transiently transfected with the indicated miRNAs. Telomere length was measured by TLF software and indicated as arbitrary fluorescence unit (a.f.u). *N* = number of analyzed nuclei. Black bar indicates mean value.

C DNA damage markers were assayed by western blotting in HeLa cells. Actin was used as loading control.

D Immunofluorescence analysis of γH2AX combined with a telomeric FISH probe (TIFs) was performed in HeLa cells transfected with the indicated miRNAs or siRNAs. Co-localizations of γH2AX with telomeres are indicated as mean number of TIFs per nucleus.

E Representative images and enlargements of co-localizations of experiment described in D.

F Immunofluorescence analysis of γH2AX combined with a SatIII FISH probe (PIFs) was performed in HeLa cells transfected with the indicated miRNAs or siRNAs. The γH2AX-positive cells with ≥ 1 PIFs per nucleus were analyzed.

G Representative images of co-localizations relative to the experiment described in (F).

H, I MDA-MB-231 and HeLa cells over-expressing TRF2 or an empty vector (pBabe) were transiently transfected with miR-Control or miR-182-3p. TRF2, pATM and γH2AX expression were assayed by western blotting. Actin was used as loading control.

Data information: For (D) and (F), data are presented as mean values ± SD. Three independent replicates were performed. Scale bar: 10 μm. At least 60 nuclei were analyzed in (D) and (F). A Student *t*-test was used to calculate statistical significance. For (B), *P* values are determined by Mann–Whitney *t*-test. All the experiments were performed 3 days post-transfection with the indicated miRNAs or siRNAs.
Source data are available online for this figure.

 © 2022 The Authors

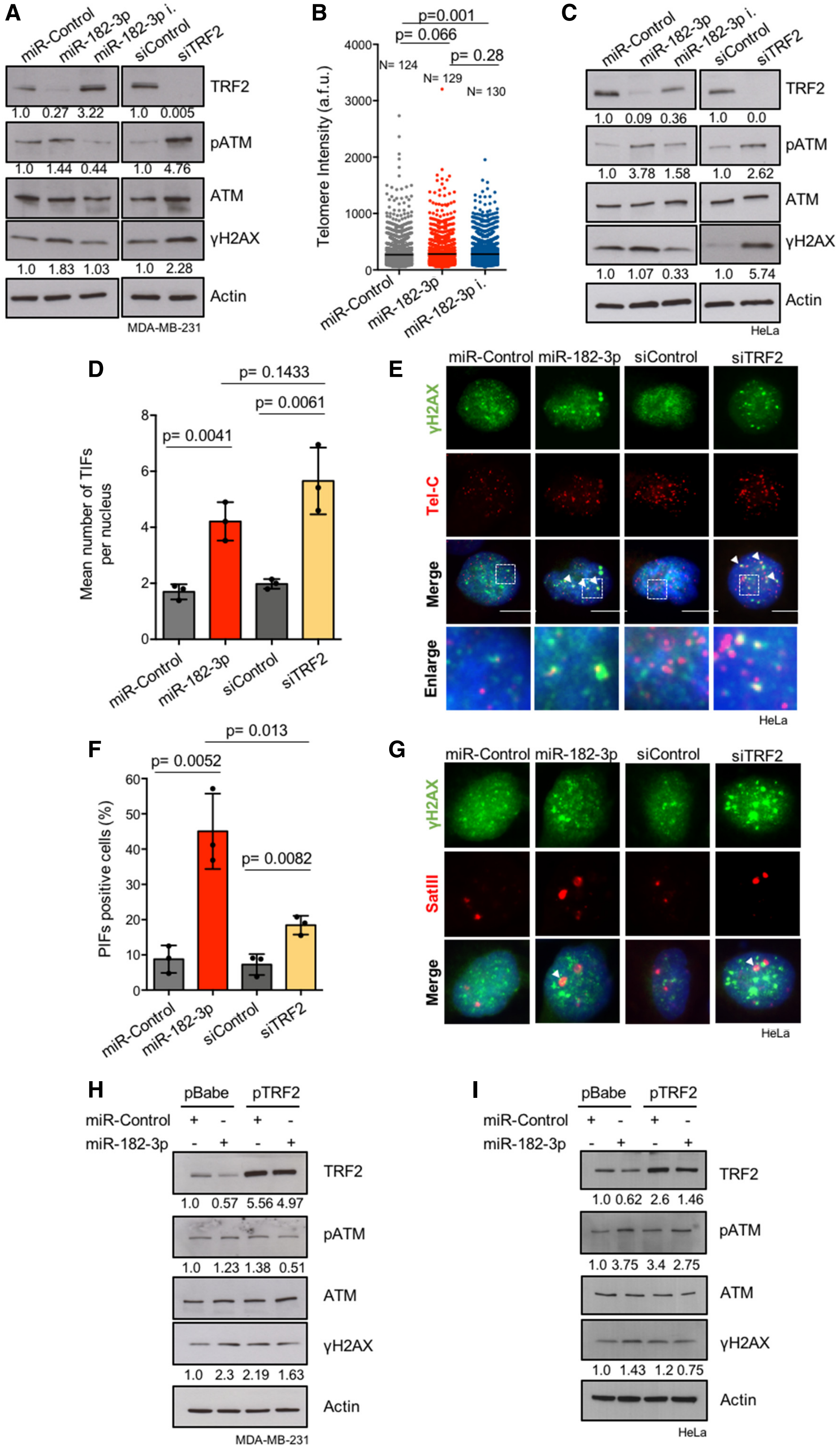
A
miR-Control
miR-182-3p
miR-182-3p i.
siControl
siTRF2
TRF2
1.0 0.27 3.22 1.0 0.005
pATM
1.0 1.44 0.44 1.0 4.76
ATM
γH2AX
1.0 1.83 1.03 1.0 2.28
Actin
MDA-MB-231
B
p=0.001
p= 0.066
p= 0.28
N= 124
N= 129
N= 130
Telomere Intensity (a.f.u.)
C
1.0 0.09 0.36 1.0 0.0
1.0 3.78 1.58 1.0 2.62
1.0 1.07 0.33 1.0 5.74
HeLa
D
p= 0.1433
p= 0.0061
p= 0.0041
Mean number of TIFs per nucleus
E
Tel-C
Merge
Enlarge
F
p= 0.013
p= 0.0052
p= 0.0082
PIFs positive cells (%)
G
SatIII
H
pBabe
pTRF2
1.0 0.57 5.56 4.97
1.0 1.23 1.38 0.51
1.0 2.3 2.19 1.63
I
1.0 0.62 2.6 1.46
1.0 3.75 3.4 2.75
1.0 1.43 1.2 0.75

Figure EV2.

Figure EV3. Effects of miR-182-3p over-expression in epithelial breast cancer cells.

A TRF2 and γH2AX expression after two rounds of transfection with the indicated miRNAs, was analyzed by western blotting in MCF10A cells. Actin was used as loading control.

B–E The mean number of γH2AX foci (B) and TIFs (D) per nucleus were analyzed 72 h post-transfection with the indicated mimic miRNAs in MCF10A cells. Representative images (C) and (E) are referred to the experiment showed in (B) and (D) respectively.

F, G Cell confluence (F) of MCF10A was monitored by Incucyte, every 24 h starting from the day of the second transfection, and cell number (G) was counted at the end of experiment (day 4).

H–I Cell cycle progression analysis by PI staining (H) and cell death analysis by Annexin V assay (I) were performed in MCF10A upon two rounds of transfection with the indicated miRNAs.

J β-Galactosidase assay in MCF10A cells after two rounds of transfection with mimic miR-Control or miR-182-3p. Left panel: Analysis of β-galactosidase-positive cells. Right panel: Representative images.

Data information: Panels (B, D, F, G, J) data are presented as mean values ± SD. A Student *t*-test was used to calculate statistical significance. *P* values are indicated.
Source data are available online for this figure.

© 2022 The Authors

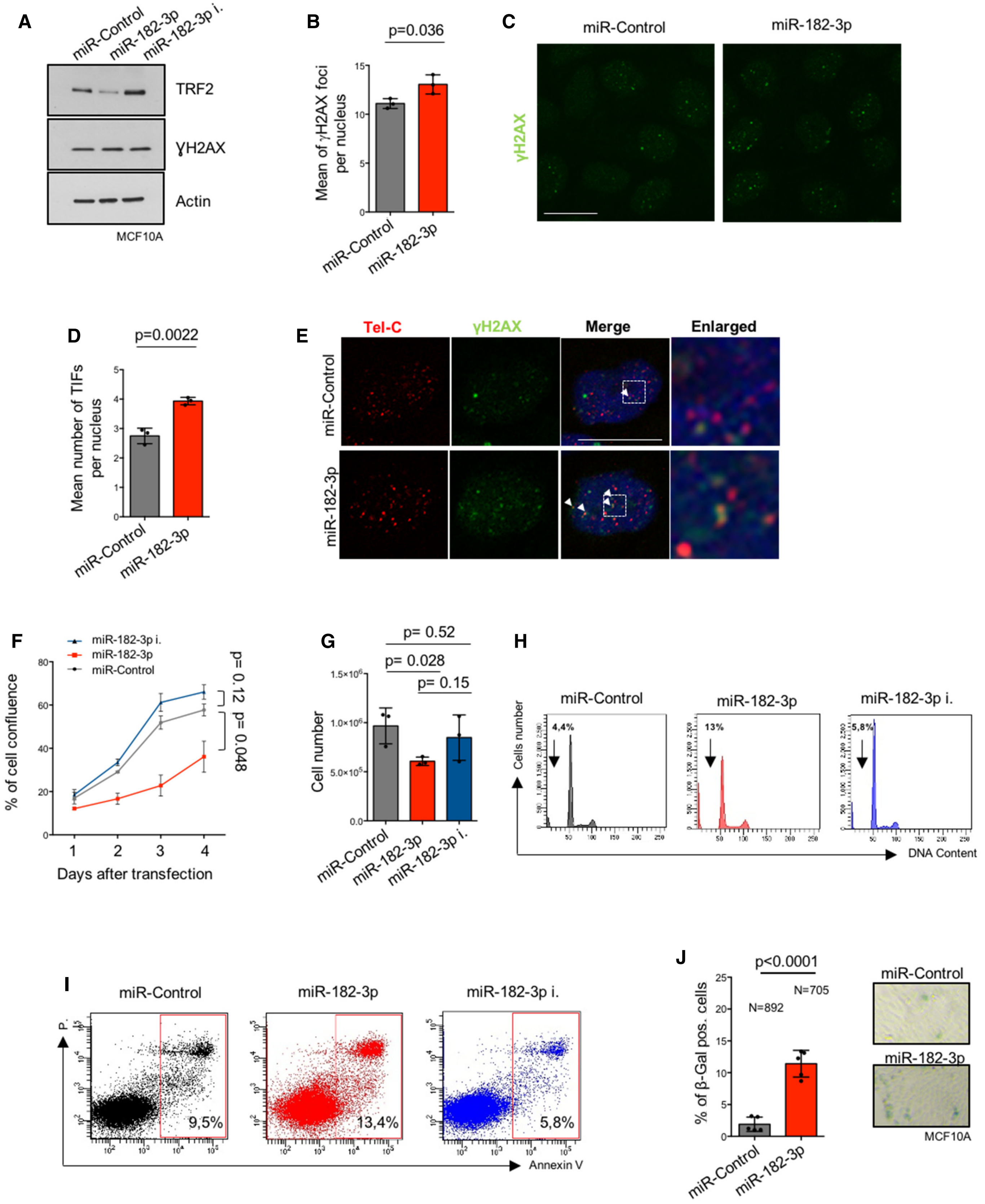
A
miR-Control
miR-182-3p
miR-182-3p i.
TRF2
γH2AX
Actin
MCF10A
B
p=0.036
Mean of γH2AX foci per nucleus
miR-Control
miR-182-3p
C
miR-Control
miR-182-3p
γH2AX
D
p=0.0022
Mean number of TIFs per nucleus
E
Tel-C
γH2AX
Merge
Enlarged
F
miR-182-3p i.
miR-182-3p
miR-Control
% of cell confluence
Days after transfection
p= 0.12
p= 0.048
G
p= 0.52
p= 0.028
p= 0.15
Cell number
H
Cells number
4,4%
13%
5,8%
DNA Content
I
P.I.
9,5%
13,4%
5,8%
Annexin V
J
p<0.0001
N=892
N=705
% of β-Gal pos. cells
MCF10A

Figure EV3.

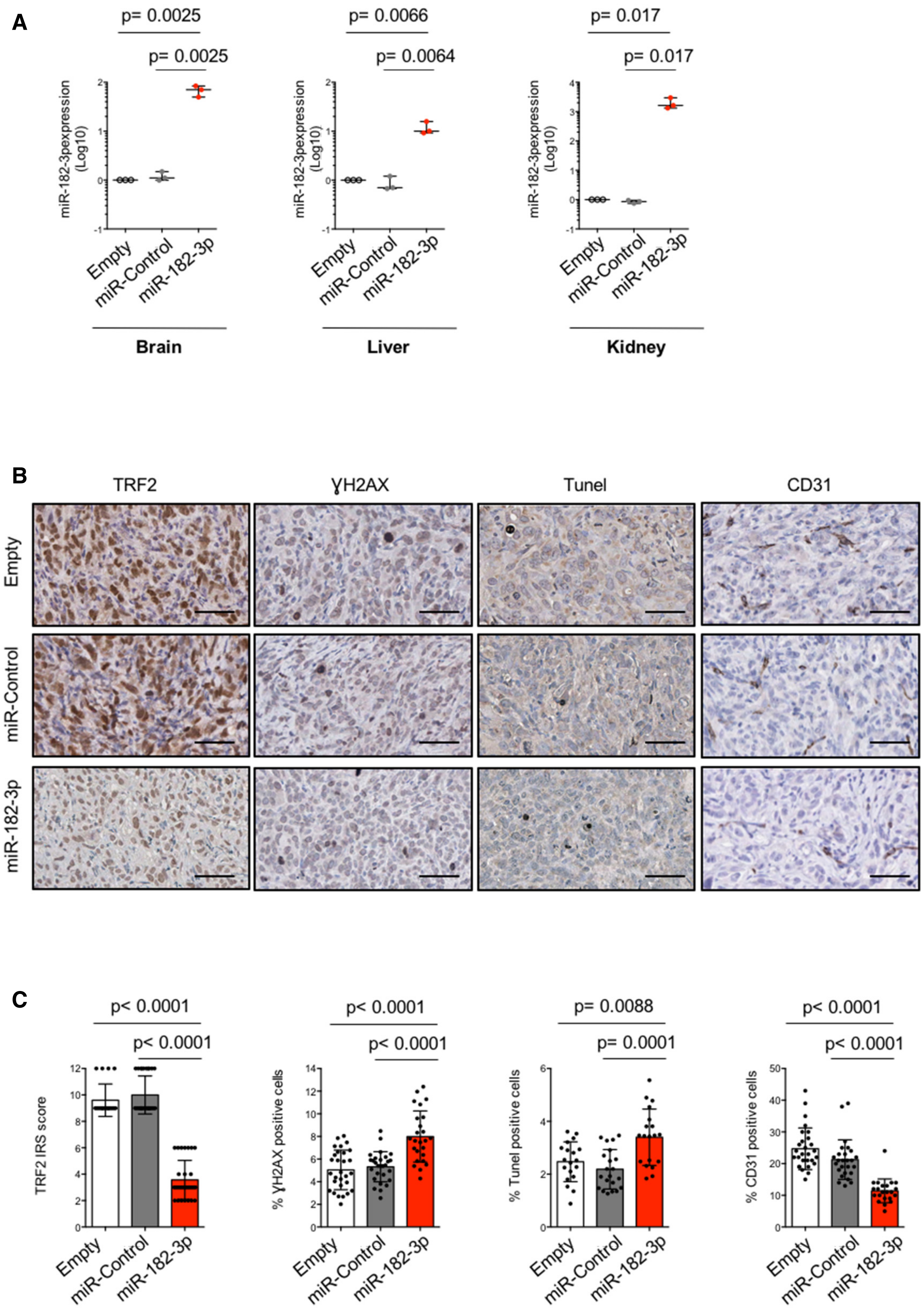

Figure EV4.

 © 2022 The Authors

◀ **Figure EV4. LNPs-miR-182-3p treatment reaches different organs and reduces TRF2 expression in tumor tissue.**

A The organs (brain, liver, kidney) taken from mice, previously engrafted with MDA-MB-231 cells and treated with LNPs-empty, LNPs-miR-Control or LNPs-miR-182-3p, were assayed for miR-182-3p expression by TaqMan qPCR.

B Representative images show IHC analysis on tumor samples, from mice bearing MDA-MB-436 human breast cancer xenografts, with the indicated markers. Scale bar: 50 μm.

C The histograms show the expression of TRF2 indicated as immunoreactivity score (IRS) and the percentage of positive cells to γH2AX, TIUNEL or CD31 staining in MDA-MB-436 xenografts. Three mice per group were analyzed, the points represent the number of field analyzed for each condition.

Data information: For (A, C), data are presented as mean values ± SD. Statistical significance using unpaired (A) or Mann–Whitney *t*-test (C) was calculated. Source data are available online for this figure.

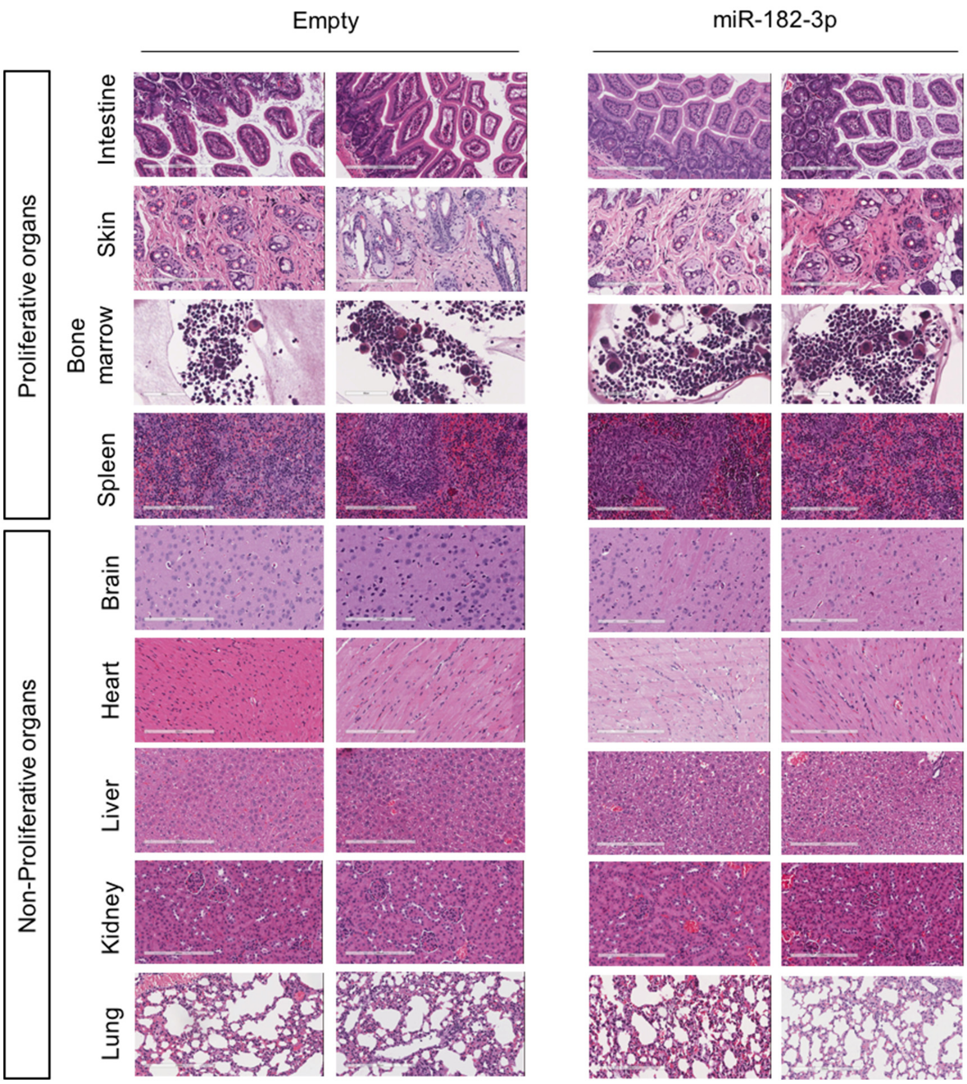

Figure EV5. LNPs-miR-182-3p treatment does not compromise tissue viability in proliferative and non-proliferative organs.

Proliferative (Intestine, Skin, Bone marrow, Spleen) and non-proliferative (Brain, Heart, Liver, Kidney, Lung) organs taken from mice, previously treated with LNPs-empty or LNPs-miR-182-3p, were assayed by hematoxylin and eosin (H&E) staining. Sections from two different mice are shown. Scale bar: 200 μm.

© 2022 The Authors
