# Supplementary material for: MiR‐182‐3p targets TRF2 and impairs tumor growth of triple‐negative breast cancer
Source: EMBO Mol Med. 2022 Nov 25;15(1):e16033. doi: 10.15252/emmm.202216033 (PMC9832842; doi:10.15252/emmm.202216033)
Supplement: Supplementary file 3 — Table EV1 [file EMMM-15-e16033-s010.pdf]

Table EV1. High-throughput luciferase miRNAs screening.
Selected miRNAs targeting the 3'UTR of TRF2 tested in the screening are shown below. In *italics* TRF2 targeting miRNAs already reported in the literature. Two independent experiments were performed.

	Renilla/Firefly Ratio			
**miRNAs**	**Experiment 1**	**Experiment 2**	**Average**	**Stand.Dev**
hsa-miR-182*	0,311467542	0,44608985	0,378778696	0,095192347
hsa-miR-519e*	0,331903826	0,514975042	0,423439434	0,129450898
hsa-miR-296-3p	0,35150408	0,709650582	0,530577331	0,253247821
hsa-miR-508-5p	0,564598783	0,831281198	0,697939991	0,188572944
hsa-miR-1207-5p	0,669195399	0,740099834	0,704647616	0,050137007
hsa-miR-1	0,78289545	0,715806988	0,749351219	0,047438706
hsa-miR-515-5p	0,681550061	0,835274542	0,758412302	0,108699623
hsa-miR-363*	0,607329194	0,957737105	0,78253315	0,24777581
hsa-miR-206	0,723444443	0,8718802	0,797662321	0,10495993
hsa-let-7e	0,545741667	1,054908486	0,800325076	0,360035311
hsa-miR-23a	0,731804741	0,901331115	0,816567928	0,119873249
hsa-miR-760	0,807697667	0,945257903	0,876477785	0,097269776
hsa-miR-596	0,742022882	1,061397671	0,901710276	0,225832078
hsa-miR-23b	0,791627316	1,031447587	0,911537452	0,16957854
hsa-miR-129-5p	1,016798006	0,832113145	0,924455575	0,130591918
hsa-miR-130a*	0,855815826	1,151081531	1,003448678	0,208784382
hsa-miR-1909	0,693533155	1,315973378	1,004753266	0,440131703
hsa-miR-125b-2*	0,69121085	1,565557404	1,128384127	0,618256378
hsa-miR-29b-1*	0,917496246	1,354575707	1,136035976	0,309061851
hsa-miR-148a	1,057484789	1,27437604	1,165930414	0,153365274
hsa-miR-659	0,921862179	1,430948419	1,176405299	0,359978333
hsa-miR-570	0,741186853	1,655574043	1,198380448	0,646569383
hsa-miR-1276	1,108947067	1,353244592	1,23109583	0,172744437
hsa-miR-663	1,184189748	1,416306156	1,300247952	0,164131087
hsa-miR-922	1,187626759	1,423960067	1,305793413	0,167112884
hsa-miR-513b	1,011967612	1,720133111	1,366050362	0,500748627
hsa-miR-412	1,293245189	1,513311148	1,403278169	0,155610132
hsa-miR-373*	1,366351349	1,464392679	1,415372014	0,069325689
hsa-miR-148b	0,988930346	1,91530782	1,452119083	0,655047794
hsa-miR-369-3p	1,597188463	1,328618968	1,462903716	0,189907311
hsa-miR-101	0,90625629	2,023627288	1,464941789	0,79010061
hsa-miR-490-3p	1,317954514	1,66156406	1,489759287	0,24296864
hsa-miR-103-2*	1,53114211	1,519800333	1,525471221	0,008019847
hsa-miR-130b	1,463702373	1,587687188	1,525694781	0,087670503
hsa-miR-186	1,890542026	1,22828619	1,559414108	0,468285593
hsa-miR-130a	1,52863402	1,591846922	1,560240471	0,044698271
hsa-miR-338-3p	1,974609466	1,156239601	1,565424533	0,578674881
hsa-miR-301a	1,628957595	1,568219634	1,598588614	0,042948224
hsa-miR-454	1,724172098	1,594509151	1,659340625	0,091685549
hsa-miR-566	1,6634206	1,684026622	1,673723611	0,014570658
hsa-miR-301b	1,674939233	1,807321131	1,741130182	0,093608138
hsa-miR-409-3p	1,427753093	2,074875208	1,75131415	0,457584436
hsa-miR-214	1,409174653	2,132113145	1,770643899	0,51119471
hsa-miR-152	1,492034494	2,11031614	1,801175317	0,437191144
hsa-miR-1268	1,568298989	2,102163062	1,835231025	0,377498906
hsa-miR-497*	1,75213265	1,973211314	1,862671982	0,156326223
hsa-miR-616*	1,870291527	2,110815308	1,990553417	0,170075997
hsa-miR-96	1,815578022	2,525124792	2,170351407	0,501725333
hsa-miR-7-2*	2,021891595	2,679201331	2,350546463	0,464788172
hsa-miR-550	2,040748711	2,828785358	2,434767034	0,557226057
hsa-miR-664*	1,87001285	3,033610649	2,45181175	0,822787894
hsa-miR-126*	2,494619993	2,565723794	2,530171894	0,050277979
hsa-miR-7-1*	2,195785791	3,151580699	2,673683245	0,675849061
hsa-miR-940	1,713117927	3,686688852	2,699903389	1,395525384
