# Supplementary material for: MiR‐182‐3p targets TRF2 and impairs tumor growth of triple‐negative breast cancer
Source: EMBO Mol Med. 2022 Nov 25;15(1):e16033. doi: 10.15252/emmm.202216033 (PMC9832842; doi:10.15252/emmm.202216033)
Supplement: Supplementary file 4 — Table EV2 [file EMMM-15-e16033-s011.pdf]

Table EV2. In silico analysis to identify other putative targets of miR-182-3p.

Analysis of miR-182-3p putative targets was performed by using the online prediction software TargetScan 8.0 (Agarwal *et al,* 2015; McGeary, Lin *et al,* 2019).

Target gene	Representative transcrip	Representative mi	Cumulative weighted context++ score	Total context++ score
GIPC3	ENST00000322315.5	hsa-miR-182-3p	-1,68	-1,68
BDKRB2	ENST00000306005.3	hsa-miR-182-3p	-1,28	-1,28
TFRC	ENST00000540528.1	hsa-miR-182-3p	-1,17	-1,17
MRPL13	ENST00000306185.3	hsa-miR-182-3p	-0,74	-0,74
GLIPR2	ENST00000396613.3	hsa-miR-182-3p	-0,73	-0,73
FEZ2	ENST00000379245.4	hsa-miR-182-3p	-0,69	-0,7
EFCAB10	ENST00000486180.1	hsa-miR-182-3p	-0,68	-0,82
ZNF622	ENST00000308683.2	hsa-miR-182-3p	-0,67	-0,67
PPP1R27	ENST00000570394.1	hsa-miR-182-3p	-0,66	-0,66
SMLR1	ENST00000541421.2	hsa-miR-182-3p	-0,65	-0,65
AC020922.1	ENST00000539076.1	hsa-miR-182-3p	-0,63	-0,63
GINS3	ENST00000318129.5	hsa-miR-182-3p	-0,59	-0,59
BNIPL	ENST00000368931.3	hsa-miR-182-3p	-0,58	-0,58
RP11-67H2.1	ENST00000521500.2	hsa-miR-182-3p	-0,58	-0,58
DYNC2LI1	ENST00000260605.8	hsa-miR-182-3p	-0,57	-0,57
CLVS1	ENST00000518592.1	hsa-miR-182-3p	-0,56	-0,56
CCDC94	ENST00000262962.7	hsa-miR-182-3p	-0,53	-0,53
VAMP3	ENST00000054666.6	hsa-miR-182-3p	-0,53	-0,61
AL359878.1	ENST00000381466.1	hsa-miR-182-3p	-0,51	-0,51
TRIM44	ENST00000299413.5	hsa-miR-182-3p	-0,5	-0,57
CXXC5	ENST00000302517.3	hsa-miR-182-3p	-0,49	-0,49
H2BFM	ENST00000355016.3	hsa-miR-182-3p	-0,49	-0,49
SLC39A11	ENST00000255559.3	hsa-miR-182-3p	-0,49	-0,49
CYCS	ENST00000305786.2	hsa-miR-182-3p	-0,49	-0,5
SMIM3	ENST00000526627.1	hsa-miR-182-3p	-0,49	-0,49
VSTM1	ENST00000425006.2	hsa-miR-182-3p	-0,48	-0,48
HLA-A	ENST00000396634.1	hsa-miR-182-3p	-0,48	-0,69
OGG1	ENST00000302008.8	hsa-miR-182-3p	-0,48	-0,48
PYROXD1	ENST00000240651.9	hsa-miR-182-3p	-0,48	-0,48
LEPROT	ENST00000371065.4	hsa-miR-182-3p	-0,48	-0,56
CDK8	ENST00000536792.1	hsa-miR-182-3p	-0,47	-0,47
HYAL1	ENST00000395144.2	hsa-miR-182-3p	-0,47	-0,47
C22orf46	ENST00000402966.1	hsa-miR-182-3p	-0,47	-0,47
SLC16A10	ENST00000368850.3	hsa-miR-182-3p	-0,47	-0,76
MTX3	ENST00000509852.1	hsa-miR-182-3p	-0,47	-0,48
HDHD2	ENST00000300605.6	hsa-miR-182-3p	-0,45	-0,51
B4GALT5	ENST00000371711.4	hsa-miR-182-3p	-0,45	-0,46
SYT12	ENST00000393946.2	hsa-miR-182-3p	-0,45	-0,45
ICA1L	ENST00000392237.2	hsa-miR-182-3p	-0,44	-0,45
DIO1	ENST00000322679.6	hsa-miR-182-3p	-0,44	-0,44
C1orf198	ENST00000366663.5	hsa-miR-182-3p	-0,43	-0,45
ISCA1	ENST00000375991.4	hsa-miR-182-3p	-0,43	-0,43
ACYP1	ENST00000357971.3	hsa-miR-182-3p	-0,43	-0,43
HAND1	ENST00000231121.2	hsa-miR-182-3p	-0,43	-0,43
FAM78B	ENST00000354422.3	hsa-miR-182-3p	-0,43	-0,43
SELT	ENST00000471696.1	hsa-miR-182-3p	-0,43	-0,43
YIPF1	ENST00000371399.1	hsa-miR-182-3p	-0,42	-0,56
ZNF135	ENST00000401053.4	hsa-miR-182-3p	-0,42	-0,42
AC016559.1	ENST00000594347.1	hsa-miR-182-3p	-0,42	-0,42
LMO7	ENST00000341547.4	hsa-miR-182-3p	-0,42	-0,42
GNPDA2	ENST00000509756.1	hsa-miR-182-3p	-0,42	-0,42
HHLA1	ENST00000434736.2	hsa-miR-182-3p	-0,42	-0,42
S100G	ENST00000380200.3	hsa-miR-182-3p	-0,41	-0,41
ZPBP2	ENST00000377940.3	hsa-miR-182-3p	-0,41	-0,41
SLC9B2	ENST00000503103.1	hsa-miR-182-3p	-0,41	-0,41
FABP2	ENST00000274024.3	hsa-miR-182-3p	-0,41	-0,41
TEX13A	ENST00000372578.3	hsa-miR-182-3p	-0,41	-0,41
FGF10	ENST00000264664.4	hsa-miR-182-3p	-0,4	-0,4

MATR3	ENST00000510056.1	hsa-miR-182-3p	-0,4	-0,4
KIAA1429	ENST00000437199.1	hsa-miR-182-3p	-0,4	-0,4
SMIM14	ENST00000295958.5	hsa-miR-182-3p	-0,4	-0,4
TMEM207	ENST00000354905.2	hsa-miR-182-3p	-0,4	-0,4
PLCXD3	ENST00000377801.3	hsa-miR-182-3p	-0,4	-0,43
OR9Q1	ENST00000335397.3	hsa-miR-182-3p	-0,39	-0,39
CASP3	ENST00000393585.2	hsa-miR-182-3p	-0,39	-0,39
IL22RA1	ENST00000270800.1	hsa-miR-182-3p	-0,39	-0,39
SLC25A24	ENST00000565488.1	hsa-miR-182-3p	-0,39	-0,39
AC010441.1	ENST00000600109.1	hsa-miR-182-3p	-0,39	-0,39
C8orf17	ENST00000507535.3	hsa-miR-182-3p	-0,39	-0,39
EFNA5	ENST00000333274.6	hsa-miR-182-3p	-0,38	-0,39
NBR1	ENST00000542611.1	hsa-miR-182-3p	-0,38	-0,41
ANKRD29	ENST00000592179.1	hsa-miR-182-3p	-0,38	-0,38
HLA-DQA1	ENST00000343139.5	hsa-miR-182-3p	-0,38	-0,59
CERKL	ENST00000410087.3	hsa-miR-182-3p	-0,38	-0,41
TBPL2	ENST00000247219.5	hsa-miR-182-3p	-0,38	-0,38
SHC4	ENST00000332408.4	hsa-miR-182-3p	-0,38	-0,38
HOXC12	ENST00000243103.3	hsa-miR-182-3p	-0,38	-0,38
GPR37L1	ENST00000367282.5	hsa-miR-182-3p	-0,38	-0,38
AC092811.1	ENST00000366845.2	hsa-miR-182-3p	-0,37	-0,37
ACN9	ENST00000360382.4	hsa-miR-182-3p	-0,37	-0,37
PLEKHS1	ENST00000354462.3	hsa-miR-182-3p	-0,37	-0,38
HNRNPA3	ENST00000411529.2	hsa-miR-182-3p	-0,37	-0,49
CTDSPL	ENST00000443503.2	hsa-miR-182-3p	-0,36	-0,42
C9orf170	ENST00000375941.2	hsa-miR-182-3p	-0,36	-0,36
HTR1F	ENST00000319595.4	hsa-miR-182-3p	-0,36	-0,36
LGI1	ENST00000371413.3	hsa-miR-182-3p	-0,36	-0,36
HPS4	ENST00000398145.2	hsa-miR-182-3p	-0,36	-0,36
SPINK13	ENST00000512953.1	hsa-miR-182-3p	-0,36	-0,36
VASH2	ENST00000366968.4	hsa-miR-182-3p	-0,36	-0,38
GINS4	ENST00000523277.2	hsa-miR-182-3p	-0,36	-0,38
RCAN2	ENST00000330430.6	hsa-miR-182-3p	-0,35	-0,35
LAMTOR1	ENST00000538404.1	hsa-miR-182-3p	-0,35	-0,39
CPA4	ENST00000445470.2	hsa-miR-182-3p	-0,35	-0,35
SYN1	ENST00000340666.4	hsa-miR-182-3p	-0,35	-0,35
LSM14B	ENST00000253001.4	hsa-miR-182-3p	-0,35	-0,46
ZNF441	ENST00000357901.4	hsa-miR-182-3p	-0,35	-0,35
BAAT	ENST00000259407.2	hsa-miR-182-3p	-0,35	-0,35
FAM71E1	ENST00000600100.1	hsa-miR-182-3p	-0,35	-0,35
BOLL	ENST00000392296.4	hsa-miR-182-3p	-0,35	-0,35
VPS13B	ENST00000395996.1	hsa-miR-182-3p	-0,35	-0,35
HECA	ENST00000367658.2	hsa-miR-182-3p	-0,35	-0,35
SRSF10	ENST00000343255.5	hsa-miR-182-3p	-0,34	-0,36
EBNA1BP2	ENST00000431635.2	hsa-miR-182-3p	-0,34	-0,34
HLA-F	ENST00000259951.7	hsa-miR-182-3p	-0,34	-0,34
MORN5	ENST00000373764.3	hsa-miR-182-3p	-0,34	-0,34
GSK3A	ENST00000222330.3	hsa-miR-182-3p	-0,34	-0,34
C10orf11	ENST00000496424.2	hsa-miR-182-3p	-0,34	-0,34
RIC3	ENST00000396677.2	hsa-miR-182-3p	-0,34	-0,34
CCDC78	ENST00000293889.6	hsa-miR-182-3p	-0,34	-0,39
UBE2B	ENST00000265339.2	hsa-miR-182-3p	-0,34	-0,35
TMEM150C	ENST00000515780.2	hsa-miR-182-3p	-0,34	-0,34
GAS8	ENST00000268699.4	hsa-miR-182-3p	-0,34	-0,35
TTC13	ENST00000366661.4	hsa-miR-182-3p	-0,34	-0,35
CCRN4L	ENST00000280614.2	hsa-miR-182-3p	-0,34	-0,57
KRT4	ENST00000551956.1	hsa-miR-182-3p	-0,34	-0,34
ARID5B	ENST00000279873.7	hsa-miR-182-3p	-0,34	-0,34
BCKDHB	ENST00000545529.1	hsa-miR-182-3p	-0,34	-0,34
MYL12B	ENST00000237500.5	hsa-miR-182-3p	-0,34	-0,34
LRRTM2	ENST00000274711.6	hsa-miR-182-3p	-0,34	-0,34
C11orf83	ENST00000377953.3	hsa-miR-182-3p	-0,33	-0,34
CD86	ENST00000330540.2	hsa-miR-182-3p	-0,33	-0,33
UBXN4	ENST00000272638.9	hsa-miR-182-3p	-0,33	-0,34
MUM1L1	ENST00000357175.2	hsa-miR-182-3p	-0,33	-0,33
GABRA2	ENST00000507460.1	hsa-miR-182-3p	-0,33	-0,72

RPGR	ENST00000338898.3	hsa-miR-182-3p	-0,33	-0,33
CMTM7	ENST00000334983.5	hsa-miR-182-3p	-0,33	-0,33
ZNF677	ENST00000599012.1	hsa-miR-182-3p	-0,33	-0,37
C3orf55	ENST00000468043.1	hsa-miR-182-3p	-0,33	-0,33
PMFBP1	ENST00000537465.1	hsa-miR-182-3p	-0,33	-0,33
IL36G	ENST00000259205.4	hsa-miR-182-3p	-0,32	-0,32
C1orf147	ENST00000367119.1	hsa-miR-182-3p	-0,32	-0,32
FOXN4	ENST00000355216.1	hsa-miR-182-3p	-0,32	-0,32
KLHL13	ENST00000371882.1	hsa-miR-182-3p	-0,32	-0,32
NEUROD4	ENST00000242994.3	hsa-miR-182-3p	-0,32	-0,32
SLC38A1	ENST00000398637.5	hsa-miR-182-3p	-0,32	-0,49
CORO2A	ENST00000343933.5	hsa-miR-182-3p	-0,32	-0,32
GABPA	ENST00000354828.3	hsa-miR-182-3p	-0,32	-0,32
CCNL2	ENST00000408952.5	hsa-miR-182-3p	-0,32	-0,79
CRIM1	ENST00000280527.2	hsa-miR-182-3p	-0,32	-0,32
FANCC	ENST00000289081.3	hsa-miR-182-3p	-0,32	-0,32
UBR7	ENST00000013070.6	hsa-miR-182-3p	-0,32	-0,34
CDK19	ENST00000368911.3	hsa-miR-182-3p	-0,32	-0,34
RNF6	ENST00000346166.3	hsa-miR-182-3p	-0,32	-0,32
UBE2I	ENST00000355803.4	hsa-miR-182-3p	-0,32	-0,32
LYVE1	ENST00000256178.3	hsa-miR-182-3p	-0,32	-0,48
TMEM117	ENST00000551577.1	hsa-miR-182-3p	-0,32	-0,32
ZNF281	ENST00000294740.3	hsa-miR-182-3p	-0,31	-0,35
DPH3	ENST00000488423.1	hsa-miR-182-3p	-0,31	-0,31
PBOV1	ENST00000527246.2	hsa-miR-182-3p	-0,31	-0,31
C18orf63	ENST00000579455.1	hsa-miR-182-3p	-0,31	-0,31
RHOJ	ENST00000316754.3	hsa-miR-182-3p	-0,31	-0,49
SBF1	ENST00000380817.3	hsa-miR-182-3p	-0,31	-0,31
TP53INP2	ENST00000374810.3	hsa-miR-182-3p	-0,31	-0,31
SMYD1	ENST00000419482.2	hsa-miR-182-3p	-0,31	-0,31
FYCO1	ENST00000296137.2	hsa-miR-182-3p	-0,31	-0,33
POLR2L	ENST00000322028.4	hsa-miR-182-3p	-0,31	-0,31
IFI27	ENST00000444961.1	hsa-miR-182-3p	-0,31	-0,31
HIAT1	ENST00000370152.3	hsa-miR-182-3p	-0,31	-0,31
RGS19	ENST00000395042.1	hsa-miR-182-3p	-0,31	-0,31
ACTRT3	ENST00000330368.2	hsa-miR-182-3p	-0,31	-0,36
P2RY11	ENST00000321826.4	hsa-miR-182-3p	-0,31	-0,31
TCEAL5	ENST00000372680.1	hsa-miR-182-3p	-0,31	-0,31
IL27RA	ENST00000263379.2	hsa-miR-182-3p	-0,31	-0,38
GREM1	ENST00000560677.1	hsa-miR-182-3p	-0,31	-0,35
RP11-204N11.1	ENST00000555187.1	hsa-miR-182-3p	-0,31	-0,31
IL6ST	ENST00000381287.4	hsa-miR-182-3p	-0,31	-0,31
RABGAP1L	ENST00000489615.1	hsa-miR-182-3p	-0,31	-0,31
TCTN3	ENST00000371217.5	hsa-miR-182-3p	-0,31	-0,31
FAM98C	ENST00000588262.1	hsa-miR-182-3p	-0,31	-0,32
FGF1	ENST00000360966.5	hsa-miR-182-3p	-0,31	-0,48
DYDC2	ENST00000372199.1	hsa-miR-182-3p	-0,3	-0,3
IGSF23	ENST00000402988.1	hsa-miR-182-3p	-0,3	-0,3
YWHAG	ENST00000307630.3	hsa-miR-182-3p	-0,3	-0,31
POLM	ENST00000335195.6	hsa-miR-182-3p	-0,3	-0,43
TNFSF8	ENST00000223795.2	hsa-miR-182-3p	-0,3	-0,3
OPALIN	ENST00000371172.3	hsa-miR-182-3p	-0,3	-0,3
MDM2	ENST00000462284.1	hsa-miR-182-3p	-0,3	-0,35
PIK3IP1	ENST00000441972.1	hsa-miR-182-3p	-0,3	-0,3
SDF4	ENST00000263741.7	hsa-miR-182-3p	-0,3	-0,3
FBXO45	ENST00000311630.6	hsa-miR-182-3p	-0,3	-0,35
SPIN3	ENST00000374919.3	hsa-miR-182-3p	-0,3	-0,3
ZFP36L2	ENST00000282388.3	hsa-miR-182-3p	-0,3	-0,3
RP11-758M4.1	ENST00000523118.1	hsa-miR-182-3p	-0,3	-0,3
KIAA1143	ENST00000296121.4	hsa-miR-182-3p	-0,3	-0,3
SLC22A6	ENST00000360421.4	hsa-miR-182-3p	-0,3	-0,3
HSD17B11	ENST00000358290.4	hsa-miR-182-3p	-0,3	-0,3
OLIG2	ENST00000382357.3	hsa-miR-182-3p	-0,3	-0,3
EML3	ENST00000394773.2	hsa-miR-182-3p	-0,3	-0,32
COX18	ENST00000295890.4	hsa-miR-182-3p	-0,3	-0,39
ABCC11	ENST00000353782.5	hsa-miR-182-3p	-0,29	-0,29

PCNXL4	ENST00000535349.1	hsa-miR-182-3p	-0,29	-0,29
ST8SIA1	ENST00000396037.4	hsa-miR-182-3p	-0,29	-0,29
LYSMD2	ENST00000267838.3	hsa-miR-182-3p	-0,29	-0,29
FAF2	ENST00000261942.6	hsa-miR-182-3p	-0,29	-0,36
EHF	ENST00000257831.3	hsa-miR-182-3p	-0,29	-0,29
MED31	ENST00000225728.3	hsa-miR-182-3p	-0,29	-0,36
XYLT2	ENST00000017003.2	hsa-miR-182-3p	-0,29	-0,29
ARL11	ENST00000282026.1	hsa-miR-182-3p	-0,29	-0,29
CSNK2A2	ENST00000262506.3	hsa-miR-182-3p	-0,29	-0,29
RP11-1C1.5	ENST00000506021.1	hsa-miR-182-3p	-0,29	-0,29
LYRM9	ENST00000460380.2	hsa-miR-182-3p	-0,29	-0,31
EMC4	ENST00000267750.4	hsa-miR-182-3p	-0,28	-0,28
RP11-295D22.1	ENST00000565320.1	hsa-miR-182-3p	-0,28	-0,28
SF3B2	ENST00000528302.1	hsa-miR-182-3p	-0,28	-0,28
PSMF1	ENST00000335877.6	hsa-miR-182-3p	-0,28	-0,35
PXMP2	ENST00000317479.3	hsa-miR-182-3p	-0,28	-0,28
SRPX	ENST00000538295.1	hsa-miR-182-3p	-0,28	-0,28
PTTG1IP	ENST00000397887.3	hsa-miR-182-3p	-0,28	-0,3
RAB15	ENST00000436278.2	hsa-miR-182-3p	-0,28	-0,36
CDKL3	ENST00000521755.1	hsa-miR-182-3p	-0,28	-0,28
CYP3A7	ENST00000336374.2	hsa-miR-182-3p	-0,28	-0,28
KCNE3	ENST00000310128.4	hsa-miR-182-3p	-0,28	-0,28
EDA	ENST00000374553.2	hsa-miR-182-3p	-0,28	-0,28
PPAN	ENST00000556468.1	hsa-miR-182-3p	-0,28	-0,28
C4orf6	ENST00000195455.2	hsa-miR-182-3p	-0,28	-0,28
PDE9A	ENST00000335512.4	hsa-miR-182-3p	-0,28	-0,34
MRPL14	ENST00000372014.3	hsa-miR-182-3p	-0,28	-0,28
CDKL5	ENST00000379996.3	hsa-miR-182-3p	-0,28	-0,28
VPS52	ENST00000482399.1	hsa-miR-182-3p	-0,28	-0,28
GATM	ENST00000396659.3	hsa-miR-182-3p	-0,28	-0,28
CYBB	ENST00000378588.4	hsa-miR-182-3p	-0,28	-0,28
CFL2	ENST00000341223.3	hsa-miR-182-3p	-0,28	-0,28
SMIM12	ENST00000521580.2	hsa-miR-182-3p	-0,28	-0,72
SLC30A4	ENST00000261867.4	hsa-miR-182-3p	-0,28	-0,28
MYADM	ENST00000391770.4	hsa-miR-182-3p	-0,27	-0,28
SNX16	ENST00000353788.4	hsa-miR-182-3p	-0,27	-0,27
ADGB	ENST00000367493.3	hsa-miR-182-3p	-0,27	-0,27
NPPA	ENST00000376480.3	hsa-miR-182-3p	-0,27	-0,27
SPATA5	ENST00000274008.4	hsa-miR-182-3p	-0,27	-0,34
POTEC	ENST00000358970.5	hsa-miR-182-3p	-0,27	-0,27
ZNF250	ENST00000292579.7	hsa-miR-182-3p	-0,27	-0,31
GEMIN6	ENST00000409011.1	hsa-miR-182-3p	-0,27	-0,27
GPR128	ENST00000273352.3	hsa-miR-182-3p	-0,27	-0,27
AL590483.1	ENST00000598000.1	hsa-miR-182-3p	-0,27	-0,27
C2orf91	ENST00000378711.2	hsa-miR-182-3p	-0,27	-0,27
CTB-186H2.3	ENST00000591669.1	hsa-miR-182-3p	-0,27	-0,27
OLFM4	ENST00000219022.2	hsa-miR-182-3p	-0,27	-0,27
ADH7	ENST00000437033.2	hsa-miR-182-3p	-0,27	-0,27
C14orf119	ENST00000319074.4	hsa-miR-182-3p	-0,27	-0,27
FIG4	ENST00000230124.3	hsa-miR-182-3p	-0,27	-0,27
MMP2	ENST00000219070.4	hsa-miR-182-3p	-0,27	-0,32
COX11	ENST00000571584.1	hsa-miR-182-3p	-0,26	-0,27
RHOV	ENST00000220507.4	hsa-miR-182-3p	-0,26	-0,26
GCM2	ENST00000379491.4	hsa-miR-182-3p	-0,26	-0,26
GADD45A	ENST00000370986.4	hsa-miR-182-3p	-0,26	-0,27
SNTB2	ENST00000336278.4	hsa-miR-182-3p	-0,26	-0,34
CTD-2162K18.4	ENST00000590750.1	hsa-miR-182-3p	-0,26	-0,26
MEI4	ENST00000602452.2	hsa-miR-182-3p	-0,26	-0,26
CNTNAP2	ENST00000361727.3	hsa-miR-182-3p	-0,26	-0,28
C15orf40	ENST00000304177.5	hsa-miR-182-3p	-0,26	-0,26
FAM221B	ENST00000423537.2	hsa-miR-182-3p	-0,26	-0,26
FAXC	ENST00000389677.5	hsa-miR-182-3p	-0,26	-0,45
C6	ENST00000337836.5	hsa-miR-182-3p	-0,26	-0,32
PTPRD	ENST00000381196.4	hsa-miR-182-3p	-0,26	-0,31
SALL4	ENST00000217086.4	hsa-miR-182-3p	-0,26	-0,27
TMEM154	ENST00000304385.3	hsa-miR-182-3p	-0,26	-0,26

SERINC4	ENST00000299969.6	hsa-miR-182-3p	-0,26	-0,26
METTL4	ENST00000319888.6	hsa-miR-182-3p	-0,26	-0,29
MMP8	ENST00000236826.3	hsa-miR-182-3p	-0,26	-0,26
CNIH4	ENST00000465271.1	hsa-miR-182-3p	-0,26	-0,26
ZNF667	ENST00000591790.1	hsa-miR-182-3p	-0,26	-0,26
PIGL	ENST00000225609.5	hsa-miR-182-3p	-0,26	-0,3
CBFB	ENST00000290858.6	hsa-miR-182-3p	-0,26	-0,27
SLC7A8	ENST00000469263.1	hsa-miR-182-3p	-0,26	-0,32
PABPC4L	ENST00000421491.3	hsa-miR-182-3p	-0,26	-0,26
RNF141	ENST00000265981.2	hsa-miR-182-3p	-0,26	-0,29
TAF11	ENST00000420584.2	hsa-miR-182-3p	-0,26	-0,48
KCNJ10	ENST00000368089.3	hsa-miR-182-3p	-0,26	-0,26
HSD17B6	ENST00000554643.1	hsa-miR-182-3p	-0,26	-0,26
DUSP6	ENST00000279488.7	hsa-miR-182-3p	-0,26	-0,26
RGS22	ENST00000360863.6	hsa-miR-182-3p	-0,26	-0,26
TNNI3K	ENST00000370891.2	hsa-miR-182-3p	-0,26	-0,26
FPGT-TNNI3K	ENST00000557284.2	hsa-miR-182-3p	-0,26	-0,26
CDCP2	ENST00000371330.1	hsa-miR-182-3p	-0,26	-0,26
TOR1B	ENST00000259339.2	hsa-miR-182-3p	-0,25	-0,32
RP11-650K20.3	ENST00000551650.1	hsa-miR-182-3p	-0,25	-0,25
ANGPT2	ENST00000325203.5	hsa-miR-182-3p	-0,25	-0,25
C10orf111	ENST00000378207.3	hsa-miR-182-3p	-0,25	-0,25
SH3BGR	ENST00000380637.3	hsa-miR-182-3p	-0,25	-0,25
NRAS	ENST00000369535.4	hsa-miR-182-3p	-0,25	-0,37
CASC1	ENST00000354189.5	hsa-miR-182-3p	-0,25	-0,25
ZNF800	ENST00000393313.1	hsa-miR-182-3p	-0,25	-0,25
CALCOCO2	ENST00000258947.3	hsa-miR-182-3p	-0,25	-0,25
DOK1	ENST00000340004.6	hsa-miR-182-3p	-0,25	-0,4
C1orf68	ENST00000368775.2	hsa-miR-182-3p	-0,25	-0,25
CDH11	ENST00000394156.3	hsa-miR-182-3p	-0,25	-0,25
PTDSS2	ENST00000308020.5	hsa-miR-182-3p	-0,25	-0,25
SERPINB7	ENST00000398019.2	hsa-miR-182-3p	-0,25	-0,25
CXCR2	ENST00000318507.2	hsa-miR-182-3p	-0,25	-0,25
PMP2	ENST00000256103.2	hsa-miR-182-3p	-0,25	-0,25
MFAP3	ENST00000436816.1	hsa-miR-182-3p	-0,25	-0,27
NUP153	ENST00000262077.2	hsa-miR-182-3p	-0,25	-0,26
MRPS17	ENST00000426595.1	hsa-miR-182-3p	-0,25	-0,29
AC106017.1	ENST00000436914.1	hsa-miR-182-3p	-0,25	-0,39
FAM216B	ENST00000537894.1	hsa-miR-182-3p	-0,25	-0,36
WWP1	ENST00000517970.1	hsa-miR-182-3p	-0,25	-0,25
AMMECR1	ENST00000262844.5	hsa-miR-182-3p	-0,25	-0,25
DLEU7	ENST00000504404.1	hsa-miR-182-3p	-0,25	-0,25
ZWILCH	ENST00000307897.5	hsa-miR-182-3p	-0,25	-0,25
LUZP2	ENST00000533227.1	hsa-miR-182-3p	-0,25	-0,25
SRSF1	ENST00000258962.4	hsa-miR-182-3p	-0,25	-0,25
CCDC152	ENST00000361970.5	hsa-miR-182-3p	-0,25	-0,25
LRRD1	ENST00000343318.5	hsa-miR-182-3p	-0,25	-0,38
DUSP10	ENST00000366899.3	hsa-miR-182-3p	-0,25	-0,25
LEMD1	ENST00000367154.1	hsa-miR-182-3p	-0,25	-0,25
ALDH1A2	ENST00000249750.4	hsa-miR-182-3p	-0,25	-0,25
CNTD1	ENST00000588408.1	hsa-miR-182-3p	-0,25	-0,25
TMEM155	ENST00000394396.1	hsa-miR-182-3p	-0,25	-0,25
SDC4	ENST00000372733.3	hsa-miR-182-3p	-0,25	-0,25
TUB	ENST00000305253.4	hsa-miR-182-3p	-0,25	-0,25
DUSP16	ENST00000298573.4	hsa-miR-182-3p	-0,25	-0,25
KRT5	ENST00000252242.4	hsa-miR-182-3p	-0,25	-0,25
GPRIN3	ENST00000609438.1	hsa-miR-182-3p	-0,25	-0,25
CCNG1	ENST00000340828.2	hsa-miR-182-3p	-0,25	-0,25
MLKL	ENST00000306247.7	hsa-miR-182-3p	-0,24	-0,24
LRRC18	ENST00000374160.3	hsa-miR-182-3p	-0,24	-0,24
DNAJC5	ENST00000360864.4	hsa-miR-182-3p	-0,24	-0,39
C5orf42	ENST00000508244.1	hsa-miR-182-3p	-0,24	-0,46
GEMIN7	ENST00000270257.4	hsa-miR-182-3p	-0,24	-0,27
DCTN5	ENST00000300087.2	hsa-miR-182-3p	-0,24	-0,26
FAM122C	ENST00000370785.3	hsa-miR-182-3p	-0,24	-0,24
KRTAP5-10	ENST00000398531.1	hsa-miR-182-3p	-0,24	-0,24

LHFPL3	ENST00000535008.1	hsa-miR-182-3p	-0,24	-0,24
ZBTB9	ENST00000395064.2	hsa-miR-182-3p	-0,24	-0,24
LLGL1	ENST00000316843.4	hsa-miR-182-3p	-0,24	-0,24
MLIP	ENST00000274897.5	hsa-miR-182-3p	-0,24	-0,24
ZNF599	ENST00000587354.2	hsa-miR-182-3p	-0,24	-0,24
SEPT11	ENST00000264893.6	hsa-miR-182-3p	-0,24	-0,25
CYP3A5	ENST00000339843.2	hsa-miR-182-3p	-0,24	-0,24
FAM91A1	ENST00000334705.7	hsa-miR-182-3p	-0,24	-0,24
LSM12	ENST00000585388.1	hsa-miR-182-3p	-0,24	-0,38
HAVCR1	ENST00000523175.1	hsa-miR-182-3p	-0,24	-0,26
ZNF185	ENST00000535861.1	hsa-miR-182-3p	-0,24	-0,24
AARS	ENST00000261772.8	hsa-miR-182-3p	-0,24	-0,24
CC2D2A	ENST00000511544.1	hsa-miR-182-3p	-0,24	-0,24
LRRC15	ENST00000347624.3	hsa-miR-182-3p	-0,24	-0,24
VHLL	ENST00000339922.3	hsa-miR-182-3p	-0,24	-0,34
PTGES3L	ENST00000453594.1	hsa-miR-182-3p	-0,24	-0,3
SAMD5	ENST00000367474.1	hsa-miR-182-3p	-0,24	-0,24
AMIGO1	ENST00000369864.4	hsa-miR-182-3p	-0,24	-0,24
GPR31	ENST00000366834.1	hsa-miR-182-3p	-0,24	-0,24
FAT1	ENST00000441802.2	hsa-miR-182-3p	-0,24	-0,3
PAK4	ENST00000599386.1	hsa-miR-182-3p	-0,24	-0,24
TMCC1	ENST00000432054.2	hsa-miR-182-3p	-0,24	-0,34
PHACTR2	ENST00000427704.2	hsa-miR-182-3p	-0,24	-0,28
RTP4	ENST00000259030.2	hsa-miR-182-3p	-0,24	-0,24
TNC	ENST00000340094.3	hsa-miR-182-3p	-0,24	-0,24
DPF3	ENST00000541685.1	hsa-miR-182-3p	-0,24	-0,24
C8orf37	ENST00000286688.5	hsa-miR-182-3p	-0,24	-0,33
TIFA	ENST00000361717.3	hsa-miR-182-3p	-0,24	-0,34
TAGLN	ENST00000532870.1	hsa-miR-182-3p	-0,24	-0,24
SLCO1A2	ENST00000307378.6	hsa-miR-182-3p	-0,23	-0,23
C20orf27	ENST00000217195.8	hsa-miR-182-3p	-0,23	-0,23
SNX22	ENST00000325881.4	hsa-miR-182-3p	-0,23	-0,23
NDRG3	ENST00000373803.2	hsa-miR-182-3p	-0,23	-0,24
ADIPOR2	ENST00000357103.4	hsa-miR-182-3p	-0,23	-0,27
ZSCAN29	ENST00000562072.1	hsa-miR-182-3p	-0,23	-0,26
RTCB	ENST00000451746.2	hsa-miR-182-3p	-0,23	-0,4
C8orf86	ENST00000358138.1	hsa-miR-182-3p	-0,23	-0,23
AP3S2	ENST00000336418.4	hsa-miR-182-3p	-0,23	-0,24
GJA9	ENST00000454994.2	hsa-miR-182-3p	-0,23	-0,26
ZNF684	ENST00000372699.3	hsa-miR-182-3p	-0,23	-0,23
COA6	ENST00000366615.4	hsa-miR-182-3p	-0,23	-0,27
SEC24D	ENST00000429811.2	hsa-miR-182-3p	-0,23	-0,23
SEMA3D	ENST00000284136.6	hsa-miR-182-3p	-0,23	-0,24
TMEM26	ENST00000399298.3	hsa-miR-182-3p	-0,23	-0,39
PARM1	ENST00000513238.1	hsa-miR-182-3p	-0,23	-0,28
SPATA20	ENST00000006658.6	hsa-miR-182-3p	-0,23	-0,23
PTMA	ENST00000409115.3	hsa-miR-182-3p	-0,23	-0,23
UBE3D	ENST00000369747.3	hsa-miR-182-3p	-0,23	-0,23
C9orf47	ENST00000375851.2	hsa-miR-182-3p	-0,23	-0,23
AC093677.1	ENST00000600169.1	hsa-miR-182-3p	-0,23	-0,23
HDAC8	ENST00000439122.2	hsa-miR-182-3p	-0,23	-0,23
DRAXIN	ENST00000294485.5	hsa-miR-182-3p	-0,23	-0,23
MTRF1L	ENST00000367231.5	hsa-miR-182-3p	-0,23	-0,23
KBTBD7	ENST00000379483.3	hsa-miR-182-3p	-0,23	-0,36
DDI2	ENST00000480945.1	hsa-miR-182-3p	-0,23	-0,23
KRTAP5-11	ENST00000398530.1	hsa-miR-182-3p	-0,23	-0,23
PLAC9	ENST00000372263.3	hsa-miR-182-3p	-0,23	-0,23
LGSN	ENST00000370658.5	hsa-miR-182-3p	-0,23	-0,23
PIAS2	ENST00000585916.1	hsa-miR-182-3p	-0,23	-0,23
FRMD6	ENST00000395718.2	hsa-miR-182-3p	-0,23	-0,24
SLC22A5	ENST00000245407.3	hsa-miR-182-3p	-0,23	-0,23
FCGR3B	ENST00000367964.2	hsa-miR-182-3p	-0,23	-0,23
HLA-DQB2	ENST00000435145.2	hsa-miR-182-3p	-0,23	-0,23
RAB6C	ENST00000410061.2	hsa-miR-182-3p	-0,23	-0,23
CARD8	ENST00000520753.1	hsa-miR-182-3p	-0,23	-0,24
RBL1	ENST00000598590.1	hsa-miR-182-3p	-0,23	-0,23

AVIL	ENST00000537081.1	hsa-miR-182-3p	-0,23	-0,23
GIF	ENST00000257248.2	hsa-miR-182-3p	-0,23	-0,23
TCHP	ENST00000405876.4	hsa-miR-182-3p	-0,23	-0,24
SNX31	ENST00000311812.2	hsa-miR-182-3p	-0,23	-0,23
TMEM176A	ENST00000004103.3	hsa-miR-182-3p	-0,23	-0,23
CCDC27	ENST00000294600.2	hsa-miR-182-3p	-0,23	-0,23
VWC2L	ENST00000427124.1	hsa-miR-182-3p	-0,23	-0,23
H2AFX	ENST00000530167.1	hsa-miR-182-3p	-0,22	-0,23
HS2ST1	ENST00000370550.5	hsa-miR-182-3p	-0,22	-0,26
KRTAP5-2	ENST00000412090.1	hsa-miR-182-3p	-0,22	-0,22
CLINT1	ENST00000523094.1	hsa-miR-182-3p	-0,22	-0,22
AJUBA	ENST00000262713.2	hsa-miR-182-3p	-0,22	-0,22
HMGXB4	ENST00000216106.5	hsa-miR-182-3p	-0,22	-0,23
LPCAT2	ENST00000262134.5	hsa-miR-182-3p	-0,22	-0,23
ACTR3C	ENST00000252071.4	hsa-miR-182-3p	-0,22	-0,22
FAM222B	ENST00000582266.1	hsa-miR-182-3p	-0,22	-0,23
SLIT2	ENST00000504154.1	hsa-miR-182-3p	-0,22	-0,32
NTSR2	ENST00000306928.5	hsa-miR-182-3p	-0,22	-0,22
AL033381.1	ENST00000314040.1	hsa-miR-182-3p	-0,22	-0,22
FCGR3A	ENST00000367969.3	hsa-miR-182-3p	-0,22	-0,22
IMPG1	ENST00000369963.3	hsa-miR-182-3p	-0,22	-0,22
ZNF233	ENST00000592581.1	hsa-miR-182-3p	-0,22	-0,23
CABP1	ENST00000453000.1	hsa-miR-182-3p	-0,22	-0,22
ANGPTL7	ENST00000376819.3	hsa-miR-182-3p	-0,22	-0,22
GMEB2	ENST00000370069.1	hsa-miR-182-3p	-0,22	-0,22
FLI1	ENST00000527786.2	hsa-miR-182-3p	-0,22	-0,35
DHODH	ENST00000219240.4	hsa-miR-182-3p	-0,22	-0,22
BMP6	ENST00000283147.6	hsa-miR-182-3p	-0,22	-0,22
IP6K1	ENST00000468463.1	hsa-miR-182-3p	-0,22	-0,27
C11orf87	ENST00000327419.6	hsa-miR-182-3p	-0,22	-0,22
CHRNA9	ENST00000310169.2	hsa-miR-182-3p	-0,22	-0,22
EPS8L3	ENST00000361852.4	hsa-miR-182-3p	-0,22	-0,22
SPDL1	ENST00000265295.4	hsa-miR-182-3p	-0,22	-0,22
VAPB	ENST00000395802.3	hsa-miR-182-3p	-0,22	-0,25
DDHD1	ENST00000323669.5	hsa-miR-182-3p	-0,22	-0,36
EMP2	ENST00000359543.3	hsa-miR-182-3p	-0,22	-0,22
UBL3	ENST00000380680.4	hsa-miR-182-3p	-0,22	-0,22
MTCP1	ENST00000369476.3	hsa-miR-182-3p	-0,22	-0,22
CCT3	ENST00000295688.3	hsa-miR-182-3p	-0,22	-0,22
BIRC6	ENST00000421745.2	hsa-miR-182-3p	-0,22	-0,22
CYP27B1	ENST00000228606.4	hsa-miR-182-3p	-0,22	-0,29
HFM1	ENST00000294696.5	hsa-miR-182-3p	-0,22	-0,22
MTMR2	ENST00000346299.5	hsa-miR-182-3p	-0,22	-0,22
MSANTD3	ENST00000395067.2	hsa-miR-182-3p	-0,22	-0,34
APOL4	ENST00000404685.3	hsa-miR-182-3p	-0,22	-0,22
TTLL9	ENST00000375921.2	hsa-miR-182-3p	-0,22	-0,22
RHOU	ENST00000366691.3	hsa-miR-182-3p	-0,22	-0,22
XG	ENST00000426774.1	hsa-miR-182-3p	-0,22	-0,22
SLC25A6	ENST00000381401.5	hsa-miR-182-3p	-0,22	-0,22
KRT84	ENST00000257951.3	hsa-miR-182-3p	-0,22	-0,22
OR13G1	ENST00000359688.2	hsa-miR-182-3p	-0,22	-0,22
CDKN2A	ENST00000498124.1	hsa-miR-182-3p	-0,21	-0,38
RBMS3	ENST00000396583.3	hsa-miR-182-3p	-0,21	-0,44
ISCU	ENST00000338291.4	hsa-miR-182-3p	-0,21	-0,67
DUSP26	ENST00000256261.4	hsa-miR-182-3p	-0,21	-0,21
AC008267.1	ENST00000600021.1	hsa-miR-182-3p	-0,21	-0,21
LINGO4	ENST00000368820.3	hsa-miR-182-3p	-0,21	-0,21
C9orf152	ENST00000400613.4	hsa-miR-182-3p	-0,21	-0,21
WDR1	ENST00000499869.2	hsa-miR-182-3p	-0,21	-0,23
TMEM164	ENST00000372073.1	hsa-miR-182-3p	-0,21	-0,24
LDLRAP1	ENST00000374338.4	hsa-miR-182-3p	-0,21	-0,21
NKAIN3	ENST00000523211.1	hsa-miR-182-3p	-0,21	-0,21
DOK2	ENST00000276420.4	hsa-miR-182-3p	-0,21	-0,21
SLC9C2	ENST00000367714.3	hsa-miR-182-3p	-0,21	-0,21
SLC4A5	ENST00000423644.1	hsa-miR-182-3p	-0,21	-0,21
SNX10	ENST00000396376.1	hsa-miR-182-3p	-0,21	-0,21

C12orf42	ENST00000548883.1	hsa-miR-182-3p	-0,21	-0,21
FAM206A	ENST00000322940.6	hsa-miR-182-3p	-0,21	-0,21
TRIM10	ENST00000449742.2	hsa-miR-182-3p	-0,21	-0,21
RBM3	ENST00000354480.2	hsa-miR-182-3p	-0,21	-0,25
PSMG4	ENST00000473000.2	hsa-miR-182-3p	-0,21	-0,21
FAM49A	ENST00000381323.3	hsa-miR-182-3p	-0,21	-0,29
MAP3K5	ENST00000359015.4	hsa-miR-182-3p	-0,21	-0,21
TFIP11	ENST00000407690.1	hsa-miR-182-3p	-0,21	-0,21
RASL12	ENST00000220062.4	hsa-miR-182-3p	-0,21	-0,21
HIST1H4I	ENST00000354348.2	hsa-miR-182-3p	-0,21	-0,21
USP18	ENST00000215794.7	hsa-miR-182-3p	-0,21	-0,21
RAB6A	ENST00000310653.6	hsa-miR-182-3p	-0,21	-0,21
AC022498.1	ENST00000392468.2	hsa-miR-182-3p	-0,21	-0,21
SLC2A12	ENST00000275230.5	hsa-miR-182-3p	-0,21	-0,26
ADAT2	ENST00000606514.1	hsa-miR-182-3p	-0,21	-0,21
CD8A	ENST00000456996.2	hsa-miR-182-3p	-0,21	-0,21
CA3	ENST00000285381.2	hsa-miR-182-3p	-0,21	-0,21
ELK3	ENST00000228741.3	hsa-miR-182-3p	-0,21	-0,21
PIN1	ENST00000247970.4	hsa-miR-182-3p	-0,21	-0,33
TBC1D31	ENST00000378080.2	hsa-miR-182-3p	-0,21	-0,21
C5orf45	ENST00000518219.1	hsa-miR-182-3p	-0,21	-0,21
ZNF786	ENST00000316286.9	hsa-miR-182-3p	-0,21	-0,21
DUSP1	ENST00000239223.3	hsa-miR-182-3p	-0,21	-0,21
STXBP4	ENST00000376352.2	hsa-miR-182-3p	-0,21	-0,31
CYP1A1	ENST00000379727.3	hsa-miR-182-3p	-0,21	-0,21
PPAN-P2RY11	ENST00000428358.1	hsa-miR-182-3p	-0,21	-0,21
MOB1B	ENST00000309395.2	hsa-miR-182-3p	-0,21	-0,21
ROM1	ENST00000534093.1	hsa-miR-182-3p	-0,21	-0,21
CELF2	ENST00000379261.4	hsa-miR-182-3p	-0,21	-0,21
PRELID2	ENST00000334744.4	hsa-miR-182-3p	-0,21	-0,21
PACSIN2	ENST00000263246.3	hsa-miR-182-3p	-0,21	-0,22
PEX12	ENST00000225873.4	hsa-miR-182-3p	-0,21	-0,21
CYP3A4	ENST00000354593.2	hsa-miR-182-3p	-0,21	-0,21
OVCA2	ENST00000572195.1	hsa-miR-182-3p	-0,21	-0,21
POLR2K	ENST00000353107.3	hsa-miR-182-3p	-0,21	-0,21
RAB27A	ENST00000396307.2	hsa-miR-182-3p	-0,21	-0,21
SPACA7	ENST00000283550.3	hsa-miR-182-3p	-0,2	-0,2
BCL2L1	ENST00000376062.2	hsa-miR-182-3p	-0,2	-0,2
CHST14	ENST00000306243.5	hsa-miR-182-3p	-0,2	-0,2
CTNND1	ENST00000524630.1	hsa-miR-182-3p	-0,2	-0,2
TRPC5	ENST00000262839.2	hsa-miR-182-3p	-0,2	-0,2
KCNQ3	ENST00000388996.4	hsa-miR-182-3p	-0,2	-0,2
ZFP69B	ENST00000484445.1	hsa-miR-182-3p	-0,2	-0,2
SMIM8	ENST00000392863.1	hsa-miR-182-3p	-0,2	-0,39
MAP6D1	ENST00000318631.3	hsa-miR-182-3p	-0,2	-0,2
IYD	ENST00000344419.3	hsa-miR-182-3p	-0,2	-0,2
TRIQK	ENST00000521988.1	hsa-miR-182-3p	-0,2	-0,27
C1orf131	ENST00000318906.2	hsa-miR-182-3p	-0,2	-0,2
TCERG1	ENST00000296702.5	hsa-miR-182-3p	-0,2	-0,22
OLFML2A	ENST00000288815.5	hsa-miR-182-3p	-0,2	-0,2
LRAT	ENST00000336356.3	hsa-miR-182-3p	-0,2	-0,2
CCDC141	ENST00000420890.2	hsa-miR-182-3p	-0,2	-0,2
GIMAP5	ENST00000358647.3	hsa-miR-182-3p	-0,2	-0,2
ATG4A	ENST00000372232.3	hsa-miR-182-3p	-0,2	-0,2
MAML2	ENST00000524717.1	hsa-miR-182-3p	-0,2	-0,34
WDR26	ENST00000414423.2	hsa-miR-182-3p	-0,2	-0,2
DOCK9	ENST00000376460.1	hsa-miR-182-3p	-0,2	-0,2
FBRSL1	ENST00000434748.2	hsa-miR-182-3p	-0,2	-0,2
ARHGAP24	ENST00000395184.1	hsa-miR-182-3p	-0,2	-0,2
FAM227A	ENST00000535113.1	hsa-miR-182-3p	-0,2	-0,27
MSL3	ENST00000312196.4	hsa-miR-182-3p	-0,2	-0,41
SMC5	ENST00000361138.5	hsa-miR-182-3p	-0,2	-0,23
BEND4	ENST00000504360.1	hsa-miR-182-3p	-0,2	-0,2
TRAK1	ENST00000341421.3	hsa-miR-182-3p	-0,2	-0,21
TMEM150A	ENST00000306353.3	hsa-miR-182-3p	-0,2	-0,21
B3GALT2	ENST00000367434.4	hsa-miR-182-3p	-0,2	-0,2

CDPF1	ENST00000404744.1	hsa-miR-182-3p	-0,2	-0,2
SIRPB2	ENST00000359801.3	hsa-miR-182-3p	-0,2	-0,2
RXRB	ENST00000374685.4	hsa-miR-182-3p	-0,2	-0,2
NAA20	ENST00000310450.4	hsa-miR-182-3p	-0,2	-0,2
ATP6V1H	ENST00000355221.3	hsa-miR-182-3p	-0,2	-0,2
SPTBN1	ENST00000356805.4	hsa-miR-182-3p	-0,2	-0,2
SGTB	ENST00000381007.4	hsa-miR-182-3p	-0,2	-0,25
MEA1	ENST00000244711.3	hsa-miR-182-3p	-0,2	-0,2
GPR97	ENST00000333493.4	hsa-miR-182-3p	-0,2	-0,2
AGT	ENST00000366667.4	hsa-miR-182-3p	-0,2	-0,2
NSFL1C	ENST00000353088.2	hsa-miR-182-3p	-0,2	-0,21
ENTPD6	ENST00000433259.2	hsa-miR-182-3p	-0,2	-0,2
ONECUT1	ENST00000560699.2	hsa-miR-182-3p	-0,2	-0,2
TCP11L2	ENST00000547153.1	hsa-miR-182-3p	-0,2	-0,2
ZNF80	ENST00000482457.2	hsa-miR-182-3p	-0,2	-0,2
ZC4H2	ENST00000545618.1	hsa-miR-182-3p	-0,2	-0,2
SOCS7	ENST00000577233.1	hsa-miR-182-3p	-0,2	-0,23
UBE2G2	ENST00000345496.2	hsa-miR-182-3p	-0,2	-0,2
DAPK2	ENST00000261891.3	hsa-miR-182-3p	-0,2	-0,22
CCZ1B	ENST00000316731.8	hsa-miR-182-3p	-0,2	-0,2
MRPS10	ENST00000053468.3	hsa-miR-182-3p	-0,2	-0,2
S100A7A	ENST00000368729.4	hsa-miR-182-3p	-0,2	-0,2
MAGI2	ENST00000419488.1	hsa-miR-182-3p	-0,2	-0,2
TMPRSS4	ENST00000534111.1	hsa-miR-182-3p	-0,2	-0,2
GSTM5	ENST00000369813.1	hsa-miR-182-3p	-0,2	-0,2
TTC22	ENST00000371276.4	hsa-miR-182-3p	-0,2	-0,2
CCNK	ENST00000389879.5	hsa-miR-182-3p	-0,2	-0,2
CHAD	ENST00000258969.4	hsa-miR-182-3p	-0,2	-0,2
KDSR	ENST00000406396.3	hsa-miR-182-3p	-0,2	-0,31
PIK3CD	ENST00000536656.1	hsa-miR-182-3p	-0,2	-0,2
PPEF2	ENST00000286719.7	hsa-miR-182-3p	-0,2	-0,2
NPFFR2	ENST00000344413.5	hsa-miR-182-3p	-0,2	-0,2
C16orf98	ENST00000561916.2	hsa-miR-182-3p	-0,2	-0,2
HUWE1	ENST00000342160.3	hsa-miR-182-3p	-0,2	-0,2
PLEKHA1	ENST00000538022.1	hsa-miR-182-3p	-0,2	-0,2
SPNS2	ENST00000329078.3	hsa-miR-182-3p	-0,2	-0,2
SLC45A3	ENST00000367145.3	hsa-miR-182-3p	-0,2	-0,2
AP3S1	ENST00000316788.7	hsa-miR-182-3p	-0,2	-0,2
C15orf38-AP3S2	ENST00000398333.3	hsa-miR-182-3p	-0,19	-0,2
DZIP1L	ENST00000327532.2	hsa-miR-182-3p	-0,19	-0,22
FBXO28	ENST00000424254.2	hsa-miR-182-3p	-0,19	-0,38
FCRLA	ENST00000367959.2	hsa-miR-182-3p	-0,19	-0,19
ANKRD34B	ENST00000338682.3	hsa-miR-182-3p	-0,19	-0,19
CNN3	ENST00000370206.4	hsa-miR-182-3p	-0,19	-0,19
ARHGEF35	ENST00000378115.2	hsa-miR-182-3p	-0,19	-0,19
ZNF227	ENST00000313040.7	hsa-miR-182-3p	-0,19	-0,19
CHM	ENST00000357749.2	hsa-miR-182-3p	-0,19	-0,19
GP5	ENST00000401815.1	hsa-miR-182-3p	-0,19	-0,19
LUC7L3	ENST00000505658.1	hsa-miR-182-3p	-0,19	-0,22
AP000867.1	ENST00000343767.3	hsa-miR-182-3p	-0,19	-0,19
TPD52	ENST00000379096.5	hsa-miR-182-3p	-0,19	-0,2
DCLK3	ENST00000416516.2	hsa-miR-182-3p	-0,19	-0,19
SLC39A10	ENST00000409086.3	hsa-miR-182-3p	-0,19	-0,2
CACNG3	ENST00000005284.3	hsa-miR-182-3p	-0,19	-0,19
CDK2	ENST00000266970.4	hsa-miR-182-3p	-0,19	-0,19
LPPR1	ENST00000374874.3	hsa-miR-182-3p	-0,19	-0,19
SH3BP5	ENST00000383791.3	hsa-miR-182-3p	-0,19	-0,3
PPAP2A	ENST00000264775.5	hsa-miR-182-3p	-0,19	-0,25
ALG6	ENST00000371108.4	hsa-miR-182-3p	-0,19	-0,25
CCDC113	ENST00000443128.2	hsa-miR-182-3p	-0,19	-0,2
ELOVL5	ENST00000370918.4	hsa-miR-182-3p	-0,19	-0,19
NRG1	ENST00000341377.5	hsa-miR-182-3p	-0,19	-0,2
CNR2	ENST00000374472.4	hsa-miR-182-3p	-0,19	-0,19
CHST9	ENST00000580774.1	hsa-miR-182-3p	-0,19	-0,19
AL590452.1	ENST00000596396.1	hsa-miR-182-3p	-0,19	-0,19
WWC3	ENST00000380861.4	hsa-miR-182-3p	-0,19	-0,19

CHMP7	ENST00000313219.7	hsa-miR-182-3p	-0,19	-0,19
NCALD	ENST00000395923.1	hsa-miR-182-3p	-0,19	-0,19
ADARB2	ENST00000381312.1	hsa-miR-182-3p	-0,19	-0,19
CNTF	ENST00000361987.4	hsa-miR-182-3p	-0,19	-0,22
RWDD2B	ENST00000493196.1	hsa-miR-182-3p	-0,19	-0,23
PRDX3	ENST00000356951.3	hsa-miR-182-3p	-0,19	-0,19
ITK	ENST00000422843.3	hsa-miR-182-3p	-0,19	-0,19
SIGLECL1	ENST00000316401.7	hsa-miR-182-3p	-0,19	-0,19
DDX21	ENST00000354185.4	hsa-miR-182-3p	-0,19	-0,21
RABGEF1	ENST00000284957.5	hsa-miR-182-3p	-0,19	-0,29
ZNF709	ENST00000397732.3	hsa-miR-182-3p	-0,19	-0,26
MURC	ENST00000307584.5	hsa-miR-182-3p	-0,19	-0,32
SYNJ2BP	ENST00000256366.4	hsa-miR-182-3p	-0,19	-0,46
C3orf52	ENST00000264848.5	hsa-miR-182-3p	-0,19	-0,19
PCK1	ENST00000319441.4	hsa-miR-182-3p	-0,19	-0,19
YWHAE	ENST00000264335.8	hsa-miR-182-3p	-0,19	-0,19
BRINP2	ENST00000361539.4	hsa-miR-182-3p	-0,19	-0,19
SLC38A11	ENST00000303735.4	hsa-miR-182-3p	-0,19	-0,19
GNG2	ENST00000556752.1	hsa-miR-182-3p	-0,19	-0,19
ADAM9	ENST00000487273.2	hsa-miR-182-3p	-0,19	-0,19
SH3TC2	ENST00000502274.1	hsa-miR-182-3p	-0,19	-0,59
SCGB2A2	ENST00000525380.1	hsa-miR-182-3p	-0,19	-0,19
TM4SF1	ENST00000472441.1	hsa-miR-182-3p	-0,19	-0,19
SLC6A4	ENST00000401766.2	hsa-miR-182-3p	-0,19	-0,19
CCDC18	ENST00000334652.5	hsa-miR-182-3p	-0,19	-0,19
LIN54	ENST00000395282.2	hsa-miR-182-3p	-0,19	-0,23
SPAG1	ENST00000251809.3	hsa-miR-182-3p	-0,19	-0,19
HDGFL1	ENST00000510882.2	hsa-miR-182-3p	-0,19	-0,19
AZI2	ENST00000479665.1	hsa-miR-182-3p	-0,19	-0,34
ZNF891	ENST00000537226.1	hsa-miR-182-3p	-0,19	-0,27
DYNLT3	ENST00000378581.3	hsa-miR-182-3p	-0,19	-0,23
IQCA1	ENST00000409907.3	hsa-miR-182-3p	-0,19	-0,19
NF1	ENST00000358273.4	hsa-miR-182-3p	-0,19	-0,19
POU2F1	ENST00000367866.2	hsa-miR-182-3p	-0,19	-0,2
TTC3	ENST00000399017.2	hsa-miR-182-3p	-0,19	-0,21
RNASEH2A	ENST00000221486.4	hsa-miR-182-3p	-0,19	-0,25
DNAH6	ENST00000389394.3	hsa-miR-182-3p	-0,19	-0,19
SENP8	ENST00000544411.1	hsa-miR-182-3p	-0,19	-0,19
CEACAM3	ENST00000221999.4	hsa-miR-182-3p	-0,19	-0,19
DDAH2	ENST00000375789.2	hsa-miR-182-3p	-0,19	-0,2
TRIM71	ENST00000383763.5	hsa-miR-182-3p	-0,19	-0,22
ACTG1	ENST00000331925.2	hsa-miR-182-3p	-0,19	-0,19
C2orf43	ENST00000440866.2	hsa-miR-182-3p	-0,19	-0,19
SLC35B4	ENST00000378509.4	hsa-miR-182-3p	-0,19	-0,21
FOXJ3	ENST00000372571.1	hsa-miR-182-3p	-0,19	-0,19
FGF7	ENST00000267843.4	hsa-miR-182-3p	-0,19	-0,19
KALRN	ENST00000393496.1	hsa-miR-182-3p	-0,19	-0,19
SLC6A16	ENST00000454748.3	hsa-miR-182-3p	-0,19	-0,19
ADM2	ENST00000395738.2	hsa-miR-182-3p	-0,19	-0,19
RALGPS1	ENST00000259351.5	hsa-miR-182-3p	-0,19	-0,22
FAM105A	ENST00000274217.3	hsa-miR-182-3p	-0,19	-0,29
B4GALT1	ENST00000379731.4	hsa-miR-182-3p	-0,19	-0,2
MUC22	ENST00000561890.1	hsa-miR-182-3p	-0,18	-0,18
CBLN2	ENST00000585159.1	hsa-miR-182-3p	-0,18	-0,18
CTAGE8	ENST00000487179.1	hsa-miR-182-3p	-0,18	-0,18
CMKLR1	ENST00000312143.7	hsa-miR-182-3p	-0,18	-0,18
CTAGE4	ENST00000486333.1	hsa-miR-182-3p	-0,18	-0,18
FAM154A	ENST00000380530.1	hsa-miR-182-3p	-0,18	-0,18
RAB3IP	ENST00000483530.2	hsa-miR-182-3p	-0,18	-0,32
TAZ	ENST00000299328.5	hsa-miR-182-3p	-0,18	-0,18
NAP1L1	ENST00000261182.8	hsa-miR-182-3p	-0,18	-0,18
NR1I2	ENST00000393716.2	hsa-miR-182-3p	-0,18	-0,18
ST8SIA3	ENST00000324000.3	hsa-miR-182-3p	-0,18	-0,18
BTN1A1	ENST00000244513.6	hsa-miR-182-3p	-0,18	-0,18
B4GALT4	ENST00000467604.1	hsa-miR-182-3p	-0,18	-0,18
TPM3	ENST00000368531.2	hsa-miR-182-3p	-0,18	-0,19

DKK2	ENST00000285311.3	hsa-miR-182-3p	-0,18	-0,18
RAN	ENST00000543796.1	hsa-miR-182-3p	-0,18	-0,29
CDKL2	ENST00000429927.2	hsa-miR-182-3p	-0,18	-0,22
PCMTD2	ENST00000308824.6	hsa-miR-182-3p	-0,18	-0,21
STT3B	ENST00000295770.2	hsa-miR-182-3p	-0,18	-0,19
MDM4	ENST00000391947.2	hsa-miR-182-3p	-0,18	-0,19
NARS2	ENST00000281038.5	hsa-miR-182-3p	-0,18	-0,18
TMEM248	ENST00000341567.4	hsa-miR-182-3p	-0,18	-0,18
ANAPC15	ENST00000543587.1	hsa-miR-182-3p	-0,18	-0,18
PRAMEF19	ENST00000540591.1	hsa-miR-182-3p	-0,18	-0,18
GNAS	ENST00000371075.3	hsa-miR-182-3p	-0,18	-0,18
C1orf177	ENST00000358193.3	hsa-miR-182-3p	-0,18	-0,18
PHYHIPL	ENST00000373880.4	hsa-miR-182-3p	-0,18	-0,18
MINPP1	ENST00000371994.4	hsa-miR-182-3p	-0,18	-0,18
PRAMEF18	ENST00000376126.2	hsa-miR-182-3p	-0,18	-0,18
BNIP2	ENST00000267859.3	hsa-miR-182-3p	-0,18	-0,18
ANKRD50	ENST00000504087.1	hsa-miR-182-3p	-0,18	-0,34
DAZ3	ENST00000382365.2	hsa-miR-182-3p	-0,18	-0,18
CWC25	ENST00000225428.5	hsa-miR-182-3p	-0,18	-0,32
GORAB	ENST00000367763.3	hsa-miR-182-3p	-0,18	-0,18
GRAMD1C	ENST00000358160.4	hsa-miR-182-3p	-0,18	-0,18
LRRC66	ENST00000343457.3	hsa-miR-182-3p	-0,18	-0,18
CGN	ENST00000271636.7	hsa-miR-182-3p	-0,18	-0,18
FZD4	ENST00000531380.1	hsa-miR-182-3p	-0,18	-0,18
PTDSS1	ENST00000517309.1	hsa-miR-182-3p	-0,18	-0,21
RASSF3	ENST00000542104.1	hsa-miR-182-3p	-0,18	-0,2
NUDT5	ENST00000491614.1	hsa-miR-182-3p	-0,18	-0,19
NT5DC1	ENST00000319550.4	hsa-miR-182-3p	-0,18	-0,27
ATXN7L1	ENST00000419735.3	hsa-miR-182-3p	-0,18	-0,2
VEZF1	ENST00000584396.1	hsa-miR-182-3p	-0,18	-0,18
TRIP13	ENST00000166345.3	hsa-miR-182-3p	-0,18	-0,18
TRABD2A	ENST00000409133.1	hsa-miR-182-3p	-0,18	-0,19
PAX9	ENST00000361487.6	hsa-miR-182-3p	-0,18	-0,18
ZNF217	ENST00000371471.2	hsa-miR-182-3p	-0,18	-0,18
DCAF4L1	ENST00000333141.5	hsa-miR-182-3p	-0,18	-0,18
GTF2H1	ENST00000265963.4	hsa-miR-182-3p	-0,18	-0,18
GOLGA7B	ENST00000370602.1	hsa-miR-182-3p	-0,18	-0,18
RP5-1052I5.2	ENST00000370548.2	hsa-miR-182-3p	-0,18	-0,18
POU4F1	ENST00000377208.5	hsa-miR-182-3p	-0,18	-0,19
RSF1	ENST00000308488.6	hsa-miR-182-3p	-0,18	-0,21
COTL1	ENST00000262428.4	hsa-miR-182-3p	-0,18	-0,18
APOBEC3C	ENST00000361441.4	hsa-miR-182-3p	-0,18	-0,18
MORC3	ENST00000400485.1	hsa-miR-182-3p	-0,18	-0,18
ACTN4	ENST00000252699.2	hsa-miR-182-3p	-0,18	-0,18
LONRF2	ENST00000393437.3	hsa-miR-182-3p	-0,18	-0,19
EWSR1	ENST00000397938.2	hsa-miR-182-3p	-0,18	-0,18
MEIS2	ENST00000397624.3	hsa-miR-182-3p	-0,18	-0,19
TMTC1	ENST00000256062.5	hsa-miR-182-3p	-0,18	-0,19
ZDHHC11	ENST00000424784.2	hsa-miR-182-3p	-0,18	-0,18
PGGT1B	ENST00000419445.1	hsa-miR-182-3p	-0,18	-0,2
CCBL1	ENST00000302586.3	hsa-miR-182-3p	-0,18	-0,26
PLA2R1	ENST00000283243.7	hsa-miR-182-3p	-0,18	-0,18
AC135983.2	ENST00000593303.1	hsa-miR-182-3p	-0,18	-0,18
BSDC1	ENST00000341071.7	hsa-miR-182-3p	-0,18	-0,18
MARCH1	ENST00000274056.7	hsa-miR-182-3p	-0,18	-0,18
APOL1	ENST00000422706.1	hsa-miR-182-3p	-0,18	-0,18
EXD2	ENST00000409018.3	hsa-miR-182-3p	-0,18	-0,18
WDR12	ENST00000261015.4	hsa-miR-182-3p	-0,18	-0,18
PIRT	ENST00000580256.2	hsa-miR-182-3p	-0,18	-0,18
SCRG1	ENST00000296506.3	hsa-miR-182-3p	-0,18	-0,18
SYT14	ENST00000399639.2	hsa-miR-182-3p	-0,18	-0,18
RIPK1	ENST00000380409.2	hsa-miR-182-3p	-0,18	-0,19
THAP1	ENST00000345117.2	hsa-miR-182-3p	-0,18	-0,32
SYT4	ENST00000255224.3	hsa-miR-182-3p	-0,18	-0,18
MAP2K4	ENST00000415385.3	hsa-miR-182-3p	-0,18	-0,18
HES2	ENST00000377834.4	hsa-miR-182-3p	-0,18	-0,18

TEX35	ENST00000367642.3	hsa-miR-182-3p	-0,18	-0,18
DPPA4	ENST00000335658.6	hsa-miR-182-3p	-0,18	-0,18
GPR151	ENST00000311104.2	hsa-miR-182-3p	-0,18	-0,18
LRRC57	ENST00000397130.3	hsa-miR-182-3p	-0,18	-0,18
MISP	ENST00000215582.6	hsa-miR-182-3p	-0,18	-0,18
ZNF256	ENST00000598928.1	hsa-miR-182-3p	-0,18	-0,18
C1orf123	ENST00000294360.4	hsa-miR-182-3p	-0,18	-0,27
ERO1L	ENST00000395686.3	hsa-miR-182-3p	-0,18	-0,23
RAPGEF5	ENST00000401957.2	hsa-miR-182-3p	-0,17	-0,17
FABP7	ENST00000356535.4	hsa-miR-182-3p	-0,17	-0,17
DPYSL3	ENST00000398514.3	hsa-miR-182-3p	-0,17	-0,19
FBXW8	ENST00000455858.2	hsa-miR-182-3p	-0,17	-0,24
CMBL	ENST00000296658.3	hsa-miR-182-3p	-0,17	-0,23
SLC22A3	ENST00000392145.1	hsa-miR-182-3p	-0,17	-0,17
CSF1R	ENST00000286301.3	hsa-miR-182-3p	-0,17	-0,17
METTL13	ENST00000362019.3	hsa-miR-182-3p	-0,17	-0,2
UAP1L1	ENST00000360271.3	hsa-miR-182-3p	-0,17	-0,17
LARP4B	ENST00000316157.3	hsa-miR-182-3p	-0,17	-0,17
TNR	ENST00000367674.2	hsa-miR-182-3p	-0,17	-0,17
MED27	ENST00000357028.2	hsa-miR-182-3p	-0,17	-0,17
YEATS2	ENST00000305135.5	hsa-miR-182-3p	-0,17	-0,17
HOXB4	ENST00000332503.5	hsa-miR-182-3p	-0,17	-0,18
KCTD15	ENST00000284006.6	hsa-miR-182-3p	-0,17	-0,19
LRRC55	ENST00000497933.1	hsa-miR-182-3p	-0,17	-0,17
MS4A6A	ENST00000528851.1	hsa-miR-182-3p	-0,17	-0,17
BMP2	ENST00000378827.4	hsa-miR-182-3p	-0,17	-0,25
CTAGE9	ENST00000314099.8	hsa-miR-182-3p	-0,17	-0,17
DAZ2	ENST00000382449.1	hsa-miR-182-3p	-0,17	-0,17
SNAI3	ENST00000332281.5	hsa-miR-182-3p	-0,17	-0,17
ACVR1B	ENST00000257963.4	hsa-miR-182-3p	-0,17	-0,17
C2orf68	ENST00000306336.5	hsa-miR-182-3p	-0,17	-0,23
ZNF197	ENST00000383745.2	hsa-miR-182-3p	-0,17	-0,18
NPL	ENST00000367553.1	hsa-miR-182-3p	-0,17	-0,17
TPH2	ENST00000333850.3	hsa-miR-182-3p	-0,17	-0,17
GYPB	ENST00000283126.7	hsa-miR-182-3p	-0,17	-0,17
THTPA	ENST00000288014.6	hsa-miR-182-3p	-0,17	-0,17
PA2G4	ENST00000303305.6	hsa-miR-182-3p	-0,17	-0,17
FAM117B	ENST00000392238.2	hsa-miR-182-3p	-0,17	-0,17
ZNF410	ENST00000555044.1	hsa-miR-182-3p	-0,17	-0,17
ATOX1	ENST00000521264.1	hsa-miR-182-3p	-0,17	-0,19
CTD-2267D19.3	ENST00000578774.1	hsa-miR-182-3p	-0,17	-0,18
MFSD9	ENST00000258436.5	hsa-miR-182-3p	-0,17	-0,2
ENTPD7	ENST00000370489.4	hsa-miR-182-3p	-0,17	-0,18
RND2	ENST00000544533.1	hsa-miR-182-3p	-0,17	-0,17
RTN4RL1	ENST00000331238.6	hsa-miR-182-3p	-0,17	-0,17
GOLGA1	ENST00000373555.4	hsa-miR-182-3p	-0,17	-0,17
SCAMP1	ENST00000538629.1	hsa-miR-182-3p	-0,17	-0,17
FAM160B1	ENST00000369248.4	hsa-miR-182-3p	-0,17	-0,17
RAB3D	ENST00000222120.3	hsa-miR-182-3p	-0,17	-0,17
FUT9	ENST00000302103.5	hsa-miR-182-3p	-0,17	-0,17
IL2RA	ENST00000379959.3	hsa-miR-182-3p	-0,17	-0,17
HCAR2	ENST00000328880.5	hsa-miR-182-3p	-0,17	-0,17
CSTF2T	ENST00000331173.4	hsa-miR-182-3p	-0,17	-0,17
KCTD21	ENST00000340067.3	hsa-miR-182-3p	-0,17	-0,17
BCAM	ENST00000270233.6	hsa-miR-182-3p	-0,17	-0,17
PDXDC1	ENST00000396410.4	hsa-miR-182-3p	-0,17	-0,17
FRY	ENST00000380250.3	hsa-miR-182-3p	-0,17	-0,17
METTL6	ENST00000443029.1	hsa-miR-182-3p	-0,17	-0,17
ACO1	ENST00000309951.6	hsa-miR-182-3p	-0,17	-0,21
SCML1	ENST00000380045.3	hsa-miR-182-3p	-0,17	-0,17
CLOCK	ENST00000309964.4	hsa-miR-182-3p	-0,17	-0,19
ADAM12	ENST00000368679.4	hsa-miR-182-3p	-0,17	-0,17
KIT	ENST00000288135.5	hsa-miR-182-3p	-0,17	-0,17
ANKRD13A	ENST00000261739.4	hsa-miR-182-3p	-0,17	-0,17
MKS1	ENST00000313863.6	hsa-miR-182-3p	-0,17	-0,17
ZNF768	ENST00000380412.5	hsa-miR-182-3p	-0,17	-0,17

ESCO2	ENST00000305188.8	hsa-miR-182-3p	-0,17	-0,23
KLHL32	ENST00000369261.4	hsa-miR-182-3p	-0,17	-0,17
KCNJ15	ENST00000328656.4	hsa-miR-182-3p	-0,17	-0,17
ING5	ENST00000313552.6	hsa-miR-182-3p	-0,17	-0,17
PRKAA2	ENST00000371244.4	hsa-miR-182-3p	-0,17	-0,17
ZIC3	ENST00000287538.5	hsa-miR-182-3p	-0,17	-0,17
XPOT	ENST00000332707.5	hsa-miR-182-3p	-0,17	-0,23
PRPF38B	ENST00000370025.4	hsa-miR-182-3p	-0,17	-0,19
CAV1	ENST00000405348.1	hsa-miR-182-3p	-0,17	-0,23
AGGF1	ENST00000312916.7	hsa-miR-182-3p	-0,17	-0,17
DUSP27	ENST00000443333.1	hsa-miR-182-3p	-0,17	-0,17
IPO9	ENST00000361565.4	hsa-miR-182-3p	-0,17	-0,23
NME1	ENST00000511355.1	hsa-miR-182-3p	-0,17	-0,17
GJA3	ENST00000241125.3	hsa-miR-182-3p	-0,17	-0,17
GPR62	ENST00000322241.4	hsa-miR-182-3p	-0,17	-0,17
ABCA5	ENST00000392676.3	hsa-miR-182-3p	-0,17	-0,29
CD300LB	ENST00000392621.1	hsa-miR-182-3p	-0,17	-0,28
CUL5	ENST00000393094.2	hsa-miR-182-3p	-0,17	-0,17
POTEE	ENST00000358087.5	hsa-miR-182-3p	-0,17	-0,17
GABRA1	ENST00000428797.2	hsa-miR-182-3p	-0,17	-0,17
IGF1R	ENST00000268035.6	hsa-miR-182-3p	-0,17	-0,19
C17orf77	ENST00000392620.1	hsa-miR-182-3p	-0,17	-0,17
DGAT2L6	ENST00000333026.3	hsa-miR-182-3p	-0,17	-0,17
COL8A2	ENST00000303143.4	hsa-miR-182-3p	-0,17	-0,17
ARL4C	ENST00000390645.2	hsa-miR-182-3p	-0,17	-0,18
ATRNL1	ENST00000355044.3	hsa-miR-182-3p	-0,17	-0,17
RAB11B	ENST00000328024.6	hsa-miR-182-3p	-0,17	-0,17
GPR35	ENST00000319838.5	hsa-miR-182-3p	-0,17	-0,2
C21orf58	ENST00000397683.1	hsa-miR-182-3p	-0,17	-0,17
PBX1	ENST00000367897.1	hsa-miR-182-3p	-0,17	-0,23
POGLUT1	ENST00000295588.4	hsa-miR-182-3p	-0,17	-0,2
PTK2	ENST00000522684.1	hsa-miR-182-3p	-0,17	-0,23
HIST1H2BK	ENST00000396891.4	hsa-miR-182-3p	-0,17	-0,39
LRG1	ENST00000306390.6	hsa-miR-182-3p	-0,17	-0,17
ADRA1A	ENST00000380586.1	hsa-miR-182-3p	-0,17	-0,17
FAM196A	ENST00000522781.1	hsa-miR-182-3p	-0,17	-0,17
RNF223	ENST00000453464.2	hsa-miR-182-3p	-0,17	-0,17
IL4R	ENST00000395762.2	hsa-miR-182-3p	-0,17	-0,17
TMEM106C	ENST00000429772.2	hsa-miR-182-3p	-0,17	-0,17
TGFB3	ENST00000238682.3	hsa-miR-182-3p	-0,17	-0,17
JPH3	ENST00000284262.2	hsa-miR-182-3p	-0,17	-0,17
SRRM4	ENST00000267260.4	hsa-miR-182-3p	-0,17	-0,17
PRADC1	ENST00000258083.2	hsa-miR-182-3p	-0,17	-0,17
CABP5	ENST00000293255.2	hsa-miR-182-3p	-0,17	-0,17
WDR48	ENST00000302313.5	hsa-miR-182-3p	-0,16	-0,17
SREK1	ENST00000334121.6	hsa-miR-182-3p	-0,16	-0,26
MPHOSPH8	ENST00000361479.5	hsa-miR-182-3p	-0,16	-0,22
ZNF354C	ENST00000315475.6	hsa-miR-182-3p	-0,16	-0,16
RND1	ENST00000309739.5	hsa-miR-182-3p	-0,16	-0,16
FXR2	ENST00000250113.7	hsa-miR-182-3p	-0,16	-0,16
ZNF282	ENST00000479907.1	hsa-miR-182-3p	-0,16	-0,16
PWWP2A	ENST00000456329.3	hsa-miR-182-3p	-0,16	-0,17
EBF2	ENST00000535548.1	hsa-miR-182-3p	-0,16	-0,32
BVES	ENST00000314641.5	hsa-miR-182-3p	-0,16	-0,2
SLC6A7	ENST00000524041.1	hsa-miR-182-3p	-0,16	-0,16
ABHD12B	ENST00000353130.1	hsa-miR-182-3p	-0,16	-0,16
DSPP	ENST00000282478.7	hsa-miR-182-3p	-0,16	-0,16
TERF2	ENST00000254942.3	hsa-miR-182-3p	-0,16	-0,16
TNFAIP2	ENST00000560869.1	hsa-miR-182-3p	-0,16	-0,16
TLR7	ENST00000380659.3	hsa-miR-182-3p	-0,16	-0,16
PATZ1	ENST00000405309.3	hsa-miR-182-3p	-0,16	-0,16
RPRD1B	ENST00000373433.4	hsa-miR-182-3p	-0,16	-0,16
SLC16A13	ENST00000308027.6	hsa-miR-182-3p	-0,16	-0,17
TMEM192	ENST00000306480.6	hsa-miR-182-3p	-0,16	-0,17
PHOSPHO1	ENST00000310544.4	hsa-miR-182-3p	-0,16	-0,16
GRAP	ENST00000284154.5	hsa-miR-182-3p	-0,16	-0,16

NHLH1	ENST00000302101.5	hsa-miR-182-3p	-0,16	-0,16
RPS6KB1	ENST00000225577.4	hsa-miR-182-3p	-0,16	-0,16
SLC1A2	ENST00000278379.3	hsa-miR-182-3p	-0,16	-0,16
HTR5A-AS1	ENST00000395731.2	hsa-miR-182-3p	-0,16	-0,16
FAM126A	ENST00000409923.1	hsa-miR-182-3p	-0,16	-0,16
ARL10	ENST00000310389.5	hsa-miR-182-3p	-0,16	-0,16
RAC1	ENST00000348035.4	hsa-miR-182-3p	-0,16	-0,17
OPCML	ENST00000331898.7	hsa-miR-182-3p	-0,16	-0,16
ABCA13	ENST00000544596.1	hsa-miR-182-3p	-0,16	-0,16
ITGA8	ENST00000378076.3	hsa-miR-182-3p	-0,16	-0,16
SUV39H1	ENST00000453214.2	hsa-miR-182-3p	-0,16	-0,16
ROCK1	ENST00000399799.2	hsa-miR-182-3p	-0,16	-0,16
UBE2Z	ENST00000360943.5	hsa-miR-182-3p	-0,16	-0,16
NT5DC3	ENST00000392876.3	hsa-miR-182-3p	-0,16	-0,16
DAZ4	ENST00000382290.3	hsa-miR-182-3p	-0,16	-0,16
MCUR1	ENST00000379170.4	hsa-miR-182-3p	-0,16	-0,16
PTPRK	ENST00000368226.4	hsa-miR-182-3p	-0,16	-0,17
GZF1	ENST00000338121.5	hsa-miR-182-3p	-0,16	-0,16
LTBP2	ENST00000261978.4	hsa-miR-182-3p	-0,16	-0,22
PRPF19	ENST00000227524.4	hsa-miR-182-3p	-0,16	-0,16
PTPLB	ENST00000383657.5	hsa-miR-182-3p	-0,16	-0,16
GHR	ENST00000230882.4	hsa-miR-182-3p	-0,16	-0,21
R3HDML	ENST00000217043.2	hsa-miR-182-3p	-0,16	-0,16
CTPS2	ENST00000443824.1	hsa-miR-182-3p	-0,16	-0,16
PFKFB3	ENST00000536985.1	hsa-miR-182-3p	-0,16	-0,16
VCL	ENST00000372755.3	hsa-miR-182-3p	-0,16	-0,23
AARS2	ENST00000244571.4	hsa-miR-182-3p	-0,16	-0,16
WARS2	ENST00000369426.5	hsa-miR-182-3p	-0,16	-0,18
PALM2	ENST00000448454.2	hsa-miR-182-3p	-0,16	-0,16
GNAI3	ENST00000369851.4	hsa-miR-182-3p	-0,16	-0,16
TDG	ENST00000392872.3	hsa-miR-182-3p	-0,16	-0,16
TMEM100	ENST00000424486.2	hsa-miR-182-3p	-0,16	-0,16
SLA2	ENST00000262866.4	hsa-miR-182-3p	-0,16	-0,16
ARRDC2	ENST00000379656.3	hsa-miR-182-3p	-0,16	-0,16
TAT	ENST00000355962.4	hsa-miR-182-3p	-0,16	-0,16
ZFR	ENST00000265069.8	hsa-miR-182-3p	-0,16	-0,16
KCNQ5	ENST00000370398.1	hsa-miR-182-3p	-0,16	-0,16
ZNF200	ENST00000396868.3	hsa-miR-182-3p	-0,16	-0,16
FAM110B	ENST00000361488.3	hsa-miR-182-3p	-0,16	-0,23
AIF1L	ENST00000372300.1	hsa-miR-182-3p	-0,16	-0,24
TMEM216	ENST00000334888.5	hsa-miR-182-3p	-0,16	-0,19
TSPAN3	ENST00000267970.4	hsa-miR-182-3p	-0,16	-0,16
TTPAL	ENST00000372906.2	hsa-miR-182-3p	-0,16	-0,19
TIMM23	ENST00000260867.4	hsa-miR-182-3p	-0,16	-0,25
RUNX1T1	ENST00000523629.1	hsa-miR-182-3p	-0,16	-0,16
ASRGL1	ENST00000415229.2	hsa-miR-182-3p	-0,16	-0,16
DAZ1	ENST00000405239.1	hsa-miR-182-3p	-0,16	-0,16
ARL14EP	ENST00000282032.3	hsa-miR-182-3p	-0,16	-0,27
RNF217	ENST00000521654.2	hsa-miR-182-3p	-0,16	-0,31
NUMBL	ENST00000252891.4	hsa-miR-182-3p	-0,16	-0,18
SNRNP200	ENST00000323853.5	hsa-miR-182-3p	-0,16	-0,17
RGS9	ENST00000449996.3	hsa-miR-182-3p	-0,16	-0,16
HORMAD2	ENST00000336726.6	hsa-miR-182-3p	-0,16	-0,16
GJB4	ENST00000339480.1	hsa-miR-182-3p	-0,16	-0,16
SMPD1	ENST00000299397.3	hsa-miR-182-3p	-0,16	-0,16
NUDT4	ENST00000337179.5	hsa-miR-182-3p	-0,16	-0,16
FAM107B	ENST00000378470.1	hsa-miR-182-3p	-0,16	-0,53
SLC39A14	ENST00000381237.1	hsa-miR-182-3p	-0,16	-0,16
PHKG1	ENST00000452681.2	hsa-miR-182-3p	-0,16	-0,16
BMP5	ENST00000370830.3	hsa-miR-182-3p	-0,16	-0,16
RAB40C	ENST00000248139.3	hsa-miR-182-3p	-0,16	-0,16
LHX9	ENST00000367390.3	hsa-miR-182-3p	-0,16	-0,16
ITGA5	ENST00000293379.4	hsa-miR-182-3p	-0,16	-0,16
CEACAM21	ENST00000187608.9	hsa-miR-182-3p	-0,16	-0,16
INSR	ENST00000341500.5	hsa-miR-182-3p	-0,16	-0,18
ACER3	ENST00000532485.1	hsa-miR-182-3p	-0,16	-0,17

RASSF4	ENST00000374417.2	hsa-miR-182-3p	-0,16	-0,46
FAM161A	ENST00000404929.1	hsa-miR-182-3p	-0,15	-0,15
GYS2	ENST00000261195.2	hsa-miR-182-3p	-0,15	-0,15
RAB9B	ENST00000243298.2	hsa-miR-182-3p	-0,15	-0,15
TRPC3	ENST00000264811.5	hsa-miR-182-3p	-0,15	-0,16
KIF4B	ENST00000435029.4	hsa-miR-182-3p	-0,15	-0,15
TUBGCP5	ENST00000283645.4	hsa-miR-182-3p	-0,15	-0,15
PSTPIP1	ENST00000558012.1	hsa-miR-182-3p	-0,15	-0,15
ATF3	ENST00000366983.1	hsa-miR-182-3p	-0,15	-0,15
ZBTB34	ENST00000319119.4	hsa-miR-182-3p	-0,15	-0,15
ARNTL2	ENST00000546179.1	hsa-miR-182-3p	-0,15	-0,16
SLC46A3	ENST00000266943.6	hsa-miR-182-3p	-0,15	-0,15
ARHGEF3	ENST00000296315.3	hsa-miR-182-3p	-0,15	-0,15
CD164L2	ENST00000374027.3	hsa-miR-182-3p	-0,15	-0,15
AL031663.2	ENST00000595203.1	hsa-miR-182-3p	-0,15	-0,15
SLC22A9	ENST00000310969.4	hsa-miR-182-3p	-0,15	-0,15
RNF220	ENST00000372247.2	hsa-miR-182-3p	-0,15	-0,15
ATAD2B	ENST00000238789.5	hsa-miR-182-3p	-0,15	-0,15
TSPAN11	ENST00000261177.9	hsa-miR-182-3p	-0,15	-0,15
FCGR2A	ENST00000271450.6	hsa-miR-182-3p	-0,15	-0,15
PHTF2	ENST00000416283.2	hsa-miR-182-3p	-0,15	-0,15
WNT9A	ENST00000272164.5	hsa-miR-182-3p	-0,15	-0,15
GXYLT1	ENST00000398675.3	hsa-miR-182-3p	-0,15	-0,15
SORCS1	ENST00000369698.1	hsa-miR-182-3p	-0,15	-0,2
C5orf51	ENST00000381647.2	hsa-miR-182-3p	-0,15	-0,15
MXRA5	ENST00000217939.6	hsa-miR-182-3p	-0,15	-0,15
C10orf85	ENST00000369071.2	hsa-miR-182-3p	-0,15	-0,15
TAOK1	ENST00000261716.3	hsa-miR-182-3p	-0,15	-0,16
GAB3	ENST00000369575.3	hsa-miR-182-3p	-0,15	-0,15
WNT16	ENST00000222462.2	hsa-miR-182-3p	-0,15	-0,15
PLEKHG1	ENST00000367328.1	hsa-miR-182-3p	-0,15	-0,16
EIF4H	ENST00000265753.8	hsa-miR-182-3p	-0,15	-0,15
PPTC7	ENST00000354300.3	hsa-miR-182-3p	-0,15	-0,15
SKA3	ENST00000314759.5	hsa-miR-182-3p	-0,15	-0,17
DCP2	ENST00000389063.2	hsa-miR-182-3p	-0,15	-0,17
TADA2A	ENST00000394395.2	hsa-miR-182-3p	-0,15	-0,17
SETD1B	ENST00000267197.5	hsa-miR-182-3p	-0,15	-0,15
CARM1	ENST00000327064.4	hsa-miR-182-3p	-0,15	-0,15
CCR2	ENST00000400888.2	hsa-miR-182-3p	-0,15	-0,15
FGR	ENST00000374005.3	hsa-miR-182-3p	-0,15	-0,15
AADACL3	ENST00000332530.3	hsa-miR-182-3p	-0,15	-0,15
GCM1	ENST00000259803.7	hsa-miR-182-3p	-0,15	-0,15
BCL6B	ENST00000293805.5	hsa-miR-182-3p	-0,15	-0,15
ZNF383	ENST00000352998.3	hsa-miR-182-3p	-0,15	-0,22
PCDHA10	ENST00000307360.5	hsa-miR-182-3p	-0,15	-0,15
PCDHA2	ENST00000526136.1	hsa-miR-182-3p	-0,15	-0,15
PCDHA13	ENST00000289272.2	hsa-miR-182-3p	-0,15	-0,15
GPR55	ENST00000392040.1	hsa-miR-182-3p	-0,15	-0,15
PCDHA7	ENST00000525929.1	hsa-miR-182-3p	-0,15	-0,15
PCDHA5	ENST00000529859.1	hsa-miR-182-3p	-0,15	-0,15
PCDHA12	ENST00000398631.2	hsa-miR-182-3p	-0,15	-0,15
PCDHA6	ENST00000529310.1	hsa-miR-182-3p	-0,15	-0,15
PCDHA8	ENST00000531613.1	hsa-miR-182-3p	-0,15	-0,15
PCDHA11	ENST00000398640.2	hsa-miR-182-3p	-0,15	-0,15
PCDHA1	ENST00000504120.2	hsa-miR-182-3p	-0,15	-0,15
SCNN1A	ENST00000360168.3	hsa-miR-182-3p	-0,15	-0,15
PCDHAC1	ENST00000253807.2	hsa-miR-182-3p	-0,15	-0,15
SYNPR	ENST00000479198.1	hsa-miR-182-3p	-0,15	-0,15
PCDHA3	ENST00000522353.2	hsa-miR-182-3p	-0,15	-0,15
OR2H1	ENST00000377136.1	hsa-miR-182-3p	-0,15	-0,15
PCDHA4	ENST00000530339.1	hsa-miR-182-3p	-0,15	-0,15
TRPM1	ENST00000397795.2	hsa-miR-182-3p	-0,15	-0,15
PCDHA9	ENST00000532602.1	hsa-miR-182-3p	-0,15	-0,15
SIK2	ENST00000304987.3	hsa-miR-182-3p	-0,15	-0,32
CR769776.1	ENST00000414341.2	hsa-miR-182-3p	-0,15	-0,15
SIRPB1	ENST00000279477.7	hsa-miR-182-3p	-0,15	-0,15

LPAR1	ENST00000374431.3	hsa-miR-182-3p	-0,15	-0,16
EMC10	ENST00000376918.3	hsa-miR-182-3p	-0,15	-0,15
GPD2	ENST00000540309.1	hsa-miR-182-3p	-0,15	-0,15
TIPRL	ENST00000367833.2	hsa-miR-182-3p	-0,15	-0,18
NID1	ENST00000366595.3	hsa-miR-182-3p	-0,15	-0,15
PLEKHM2	ENST00000375799.3	hsa-miR-182-3p	-0,15	-0,15
LMO4	ENST00000370544.5	hsa-miR-182-3p	-0,15	-0,22
IFT140	ENST00000361339.5	hsa-miR-182-3p	-0,15	-0,15
THUMPD1	ENST00000381337.2	hsa-miR-182-3p	-0,15	-0,15
CKAP2L	ENST00000541405.1	hsa-miR-182-3p	-0,15	-0,18
LPAR5	ENST00000329858.4	hsa-miR-182-3p	-0,15	-0,15
KLF7	ENST00000423015.1	hsa-miR-182-3p	-0,15	-0,17
RAP2B	ENST00000323534.2	hsa-miR-182-3p	-0,15	-0,21
MIS18A	ENST00000290130.3	hsa-miR-182-3p	-0,15	-0,15
CRY2	ENST00000443527.2	hsa-miR-182-3p	-0,15	-0,15
UBXN10	ENST00000375099.3	hsa-miR-182-3p	-0,15	-0,15
PRLHR	ENST00000239032.2	hsa-miR-182-3p	-0,15	-0,15
KMO	ENST00000366559.4	hsa-miR-182-3p	-0,15	-0,15
PCDHAC2	ENST00000289269.5	hsa-miR-182-3p	-0,15	-0,15
TRPM7	ENST00000560955.1	hsa-miR-182-3p	-0,15	-0,15
PRPF38A	ENST00000257181.9	hsa-miR-182-3p	-0,15	-0,15
EARS2	ENST00000449606.1	hsa-miR-182-3p	-0,15	-0,2
NUTF2	ENST00000219169.4	hsa-miR-182-3p	-0,15	-0,27
TNPO1	ENST00000337273.5	hsa-miR-182-3p	-0,15	-0,17
FAM163A	ENST00000341785.4	hsa-miR-182-3p	-0,15	-0,15
ADAMDEC1	ENST00000256412.4	hsa-miR-182-3p	-0,15	-0,15
PACRGL	ENST00000502374.1	hsa-miR-182-3p	-0,15	-0,19
USP31	ENST00000219689.7	hsa-miR-182-3p	-0,15	-0,18
AMER2	ENST00000357816.2	hsa-miR-182-3p	-0,15	-0,17
ACSF3	ENST00000317447.4	hsa-miR-182-3p	-0,15	-0,16
EDNRA	ENST00000339690.5	hsa-miR-182-3p	-0,15	-0,15
TDRP	ENST00000324079.6	hsa-miR-182-3p	-0,15	-0,23
TMEM55B	ENST00000250489.4	hsa-miR-182-3p	-0,15	-0,15
TGM5	ENST00000220420.5	hsa-miR-182-3p	-0,15	-0,15
VTI1A	ENST00000393077.2	hsa-miR-182-3p	-0,15	-0,18
PCDHGA11	ENST00000398587.2	hsa-miR-182-3p	-0,15	-0,15
AGXT2	ENST00000231420.6	hsa-miR-182-3p	-0,15	-0,16
HERC4	ENST00000277817.6	hsa-miR-182-3p	-0,15	-0,15
MMACHC	ENST00000401061.4	hsa-miR-182-3p	-0,15	-0,39
ZSCAN4	ENST00000318203.5	hsa-miR-182-3p	-0,15	-0,26
KCNH5	ENST00000322893.7	hsa-miR-182-3p	-0,15	-0,2
PSKH1	ENST00000291041.5	hsa-miR-182-3p	-0,15	-0,15
SRPK2	ENST00000393651.3	hsa-miR-182-3p	-0,15	-0,31
AK7	ENST00000267584.4	hsa-miR-182-3p	-0,14	-0,14
EGFLAM	ENST00000322350.5	hsa-miR-182-3p	-0,14	-0,21
PER2	ENST00000254658.3	hsa-miR-182-3p	-0,14	-0,14
MFAP3L	ENST00000393704.3	hsa-miR-182-3p	-0,14	-0,15
AGK	ENST00000355413.4	hsa-miR-182-3p	-0,14	-0,15
FERMT1	ENST00000217289.4	hsa-miR-182-3p	-0,14	-0,16
GDAP2	ENST00000369443.5	hsa-miR-182-3p	-0,14	-0,24
CTSO	ENST00000433477.3	hsa-miR-182-3p	-0,14	-0,2
NAP1L5	ENST00000323061.5	hsa-miR-182-3p	-0,14	-0,2
PXN	ENST00000458477.2	hsa-miR-182-3p	-0,14	-0,14
ANKHD1	ENST00000360839.2	hsa-miR-182-3p	-0,14	-0,14
LPL	ENST00000311322.8	hsa-miR-182-3p	-0,14	-0,14
PI15	ENST00000260113.2	hsa-miR-182-3p	-0,14	-0,14
SNCAIP	ENST00000261368.8	hsa-miR-182-3p	-0,14	-0,14
PLD1	ENST00000342215.6	hsa-miR-182-3p	-0,14	-0,14
MSS51	ENST00000299432.2	hsa-miR-182-3p	-0,14	-0,14
TGFBR2	ENST00000359013.4	hsa-miR-182-3p	-0,14	-0,16
EHD4	ENST00000220325.4	hsa-miR-182-3p	-0,14	-0,29
CYP7B1	ENST00000310193.3	hsa-miR-182-3p	-0,14	-0,16
KIFC3	ENST00000465878.2	hsa-miR-182-3p	-0,14	-0,14
AL391421.1	ENST00000372387.1	hsa-miR-182-3p	-0,14	-0,14
AC145676.2	ENST00000514988.1	hsa-miR-182-3p	-0,14	-0,14
TAF5L	ENST00000366676.1	hsa-miR-182-3p	-0,14	-0,14

AL645730.2	ENST00000448346.1	hsa-miR-182-3p	-0,14	-0,14
SLMAP	ENST00000295951.3	hsa-miR-182-3p	-0,14	-0,14
DUOX1	ENST00000321429.4	hsa-miR-182-3p	-0,14	-0,14
TBL1X	ENST00000407597.2	hsa-miR-182-3p	-0,14	-0,22
VPS26B	ENST00000281187.5	hsa-miR-182-3p	-0,14	-0,15
MEF2A	ENST00000354410.5	hsa-miR-182-3p	-0,14	-0,15
TOR1AIP2	ENST00000367612.3	hsa-miR-182-3p	-0,14	-0,18
GNB4	ENST00000232564.3	hsa-miR-182-3p	-0,14	-0,15
LDB3	ENST00000542786.1	hsa-miR-182-3p	-0,14	-0,14
GRK5	ENST00000392870.2	hsa-miR-182-3p	-0,14	-0,14
RPS6KA1	ENST00000374168.2	hsa-miR-182-3p	-0,14	-0,14
SLAMF8	ENST00000289707.5	hsa-miR-182-3p	-0,14	-0,14
CASKIN2	ENST00000321617.3	hsa-miR-182-3p	-0,14	-0,14
TMEM139	ENST00000359333.3	hsa-miR-182-3p	-0,14	-0,14
PTGFR	ENST00000370756.3	hsa-miR-182-3p	-0,14	-0,14
ASXL2	ENST00000435504.4	hsa-miR-182-3p	-0,14	-0,18
NPPC	ENST00000409852.1	hsa-miR-182-3p	-0,14	-0,14
WDR31	ENST00000341761.4	hsa-miR-182-3p	-0,14	-0,14
STIM2	ENST00000467011.1	hsa-miR-182-3p	-0,14	-0,14
KIF4A	ENST00000374403.3	hsa-miR-182-3p	-0,14	-0,14
TEX22	ENST00000451127.2	hsa-miR-182-3p	-0,14	-0,14
DSG4	ENST00000359747.4	hsa-miR-182-3p	-0,14	-0,14
MANEA	ENST00000358812.4	hsa-miR-182-3p	-0,14	-0,16
FBXL20	ENST00000394294.3	hsa-miR-182-3p	-0,14	-0,34
RAPGEF4	ENST00000409036.1	hsa-miR-182-3p	-0,14	-0,15
SH3D19	ENST00000409598.4	hsa-miR-182-3p	-0,14	-0,2
MAX	ENST00000284165.6	hsa-miR-182-3p	-0,14	-0,14
ARHGEF37	ENST00000333677.6	hsa-miR-182-3p	-0,14	-0,14
GGA3	ENST00000351904.7	hsa-miR-182-3p	-0,14	-0,14
C11orf82	ENST00000528759.1	hsa-miR-182-3p	-0,14	-0,14
UBAP1	ENST00000545103.1	hsa-miR-182-3p	-0,14	-0,14
AKAP5	ENST00000394718.4	hsa-miR-182-3p	-0,14	-0,14
FAM210A	ENST00000322247.3	hsa-miR-182-3p	-0,14	-0,14
UBA2	ENST00000246548.4	hsa-miR-182-3p	-0,14	-0,14
SYT9	ENST00000318881.6	hsa-miR-182-3p	-0,14	-0,14
SHISA9	ENST00000558583.1	hsa-miR-182-3p	-0,14	-0,14
CD4	ENST00000011653.4	hsa-miR-182-3p	-0,14	-0,14
NCEH1	ENST00000475381.1	hsa-miR-182-3p	-0,14	-0,14
GAL3ST3	ENST00000312006.4	hsa-miR-182-3p	-0,14	-0,24
XXbac-BPG32J3.20	ENST00000461287.1	hsa-miR-182-3p	-0,14	-0,14
PCDHGA4	ENST00000571252.1	hsa-miR-182-3p	-0,14	-0,14
PCDHGB3	ENST00000576222.1	hsa-miR-182-3p	-0,14	-0,14
HERC5	ENST00000264350.3	hsa-miR-182-3p	-0,14	-0,14
PCDHGB1	ENST00000523390.1	hsa-miR-182-3p	-0,14	-0,14
PCDHGB2	ENST00000522605.1	hsa-miR-182-3p	-0,14	-0,14
ZNF384	ENST00000319770.3	hsa-miR-182-3p	-0,14	-0,14
PCDHGA1	ENST00000517417.1	hsa-miR-182-3p	-0,14	-0,14
PCDHGB4	ENST00000519479.1	hsa-miR-182-3p	-0,14	-0,14
ORAOV1	ENST00000279147.4	hsa-miR-182-3p	-0,14	-0,14
PCDHGB6	ENST00000520790.1	hsa-miR-182-3p	-0,14	-0,14
PCDHGB7	ENST00000398594.2	hsa-miR-182-3p	-0,14	-0,14
PCDHGA5	ENST00000518069.1	hsa-miR-182-3p	-0,14	-0,14
ZBTB7A	ENST00000322357.4	hsa-miR-182-3p	-0,14	-0,19
ATL2	ENST00000406122.1	hsa-miR-182-3p	-0,14	-0,14
SCRN3	ENST00000272732.6	hsa-miR-182-3p	-0,14	-0,17
PCDHGA8	ENST00000398604.2	hsa-miR-182-3p	-0,14	-0,14
PCDHGA7	ENST00000518325.1	hsa-miR-182-3p	-0,14	-0,14
PCDHGA9	ENST00000573521.1	hsa-miR-182-3p	-0,14	-0,14
PCDHGA6	ENST00000517434.1	hsa-miR-182-3p	-0,14	-0,14
PCDHGA3	ENST00000253812.6	hsa-miR-182-3p	-0,14	-0,14
DPH1	ENST00000263083.6	hsa-miR-182-3p	-0,14	-0,14
PABPC1L2A	ENST00000373519.1	hsa-miR-182-3p	-0,14	-0,14
PCDHGA2	ENST00000394576.2	hsa-miR-182-3p	-0,14	-0,14
SMAD7	ENST00000262158.2	hsa-miR-182-3p	-0,14	-0,14
MLC1	ENST00000395876.2	hsa-miR-182-3p	-0,14	-0,14
PCDHGA12	ENST00000252085.3	hsa-miR-182-3p	-0,14	-0,14

PCDHGC3	ENST00000308177.3	hsa-miR-182-3p	-0,14	-0,14
EPB41L1	ENST00000441639.1	hsa-miR-182-3p	-0,14	-0,23
ZFYVE21	ENST00000216602.6	hsa-miR-182-3p	-0,14	-0,14
EPOR	ENST00000592375.2	hsa-miR-182-3p	-0,14	-0,14
PCDHGC5	ENST00000252087.1	hsa-miR-182-3p	-0,14	-0,14
SASH1	ENST00000367467.3	hsa-miR-182-3p	-0,14	-0,14
PCDHGA10	ENST00000398610.2	hsa-miR-182-3p	-0,14	-0,14
PCDHGC4	ENST00000306593.1	hsa-miR-182-3p	-0,14	-0,14
RIN2	ENST00000255006.6	hsa-miR-182-3p	-0,14	-0,17
PAPD5	ENST00000357464.3	hsa-miR-182-3p	-0,14	-0,14
KLHDC10	ENST00000335420.5	hsa-miR-182-3p	-0,14	-0,16
CCR9	ENST00000357632.2	hsa-miR-182-3p	-0,14	-0,14
SLC23A2	ENST00000338244.1	hsa-miR-182-3p	-0,14	-0,14
NRXN1	ENST00000342183.5	hsa-miR-182-3p	-0,14	-0,14
PCYT1B	ENST00000379145.1	hsa-miR-182-3p	-0,14	-0,14
PAX7	ENST00000420770.2	hsa-miR-182-3p	-0,14	-0,14
GBP6	ENST00000370456.4	hsa-miR-182-3p	-0,14	-0,14
GDI2	ENST00000380191.4	hsa-miR-182-3p	-0,13	-0,16
PANK4	ENST00000378466.3	hsa-miR-182-3p	-0,13	-0,16
LHPP	ENST00000368839.1	hsa-miR-182-3p	-0,13	-0,14
SGCD	ENST00000435422.3	hsa-miR-182-3p	-0,13	-0,17
TAB2	ENST00000367456.1	hsa-miR-182-3p	-0,13	-0,13
TRPM4	ENST00000252826.5	hsa-miR-182-3p	-0,13	-0,13
NTRK3	ENST00000394480.2	hsa-miR-182-3p	-0,13	-0,13
RNPC3	ENST00000533099.1	hsa-miR-182-3p	-0,13	-0,13
SPC24	ENST00000592540.1	hsa-miR-182-3p	-0,13	-0,15
ZNHIT6	ENST00000431532.2	hsa-miR-182-3p	-0,13	-0,38
MTFR1	ENST00000458689.2	hsa-miR-182-3p	-0,13	-0,14
TRIM45	ENST00000256649.4	hsa-miR-182-3p	-0,13	-0,2
FAM47E-STBD1	ENST00000237642.6	hsa-miR-182-3p	-0,13	-0,17
ACSL4	ENST00000469796.2	hsa-miR-182-3p	-0,13	-0,13
AL020996.1	ENST00000536896.1	hsa-miR-182-3p	-0,13	-0,13
VIPAS39	ENST00000343765.2	hsa-miR-182-3p	-0,13	-0,13
C10orf118	ENST00000543782.1	hsa-miR-182-3p	-0,13	-0,14
NAT16	ENST00000300303.2	hsa-miR-182-3p	-0,13	-0,13
SEPT14	ENST00000388975.3	hsa-miR-182-3p	-0,13	-0,13
COX6B2	ENST00000590900.1	hsa-miR-182-3p	-0,13	-0,13
PHLDB1	ENST00000361417.2	hsa-miR-182-3p	-0,13	-0,17
ENTPD4	ENST00000358689.4	hsa-miR-182-3p	-0,13	-0,18
HIST1H3G	ENST00000305910.3	hsa-miR-182-3p	-0,13	-0,24
GMFB	ENST00000554908.1	hsa-miR-182-3p	-0,13	-0,43
ZNF775	ENST00000329630.5	hsa-miR-182-3p	-0,13	-0,13
CLNK	ENST00000226951.6	hsa-miR-182-3p	-0,13	-0,13
ZFP36L1	ENST00000555997.1	hsa-miR-182-3p	-0,13	-0,13
ILDR2	ENST00000469934.2	hsa-miR-182-3p	-0,13	-0,13
SLC34A2	ENST00000382051.3	hsa-miR-182-3p	-0,13	-0,13
USP26	ENST00000370832.1	hsa-miR-182-3p	-0,13	-0,13
KIAA0753	ENST00000361413.3	hsa-miR-182-3p	-0,13	-0,13
MRPS2	ENST00000371785.1	hsa-miR-182-3p	-0,13	-0,13
PIK3C2A	ENST00000265970.7	hsa-miR-182-3p	-0,13	-0,17
ZNF554	ENST00000317243.5	hsa-miR-182-3p	-0,13	-0,17
MCM10	ENST00000378694.1	hsa-miR-182-3p	-0,13	-0,21
NUP98	ENST00000359171.4	hsa-miR-182-3p	-0,13	-0,17
RANBP6	ENST00000259569.5	hsa-miR-182-3p	-0,13	-0,14
FOXA2	ENST00000419308.2	hsa-miR-182-3p	-0,13	-0,13
HOXC11	ENST00000546378.1	hsa-miR-182-3p	-0,13	-0,13
LYPD6	ENST00000334166.4	hsa-miR-182-3p	-0,13	-0,13
TRIM2	ENST00000338700.5	hsa-miR-182-3p	-0,13	-0,13
NFIB	ENST00000397575.3	hsa-miR-182-3p	-0,13	-0,14
ENPP4	ENST00000321037.4	hsa-miR-182-3p	-0,13	-0,14
C12orf66	ENST00000544871.1	hsa-miR-182-3p	-0,13	-0,2
KIF5A	ENST00000455537.2	hsa-miR-182-3p	-0,13	-0,13
CSTF3	ENST00000323959.4	hsa-miR-182-3p	-0,13	-0,13
XKR6	ENST00000304437.2	hsa-miR-182-3p	-0,13	-0,13
FOXO1	ENST00000379561.5	hsa-miR-182-3p	-0,13	-0,13
ZNF517	ENST00000359971.3	hsa-miR-182-3p	-0,13	-0,13

ZNF654	ENST00000309495.5	hsa-miR-182-3p	-0,13	-0,13
PRR11	ENST00000262293.4	hsa-miR-182-3p	-0,13	-0,13
FGF23	ENST00000237837.1	hsa-miR-182-3p	-0,13	-0,13
ARHGAP19-SLIT1	ENST00000453547.2	hsa-miR-182-3p	-0,13	-0,13
GALNT11	ENST00000430044.2	hsa-miR-182-3p	-0,13	-0,13
MAF	ENST00000393350.1	hsa-miR-182-3p	-0,13	-0,14
TBC1D9B	ENST00000519746.1	hsa-miR-182-3p	-0,13	-0,13
SDC1	ENST00000254351.4	hsa-miR-182-3p	-0,13	-0,13
SLC25A34	ENST00000294454.5	hsa-miR-182-3p	-0,13	-0,13
SSH1	ENST00000360239.3	hsa-miR-182-3p	-0,13	-0,13
B3GALNT2	ENST00000366600.3	hsa-miR-182-3p	-0,13	-0,13
PCDH11Y	ENST00000215473.6	hsa-miR-182-3p	-0,13	-0,13
SLC30A7	ENST00000370112.4	hsa-miR-182-3p	-0,13	-0,26
ITPRIPL1	ENST00000536814.1	hsa-miR-182-3p	-0,13	-0,13
FAM8A1	ENST00000259963.3	hsa-miR-182-3p	-0,13	-0,13
TMEM43	ENST00000306077.4	hsa-miR-182-3p	-0,13	-0,14
OPHN1	ENST00000355520.5	hsa-miR-182-3p	-0,13	-0,13
CBFA2T3	ENST00000327483.5	hsa-miR-182-3p	-0,13	-0,13
VPS8	ENST00000287546.4	hsa-miR-182-3p	-0,13	-0,13
MIER3	ENST00000381226.3	hsa-miR-182-3p	-0,13	-0,13
CLDN16	ENST00000264734.2	hsa-miR-182-3p	-0,13	-0,13
KCNJ9	ENST00000368088.3	hsa-miR-182-3p	-0,13	-0,13
XIRP2	ENST00000409195.1	hsa-miR-182-3p	-0,13	-0,13
DAZAP1	ENST00000336761.6	hsa-miR-182-3p	-0,13	-0,13
VRTN	ENST00000256362.4	hsa-miR-182-3p	-0,13	-0,13
ITFG3	ENST00000399932.3	hsa-miR-182-3p	-0,13	-0,13
ZNF597	ENST00000301744.4	hsa-miR-182-3p	-0,13	-0,15
UBE2G1	ENST00000396981.2	hsa-miR-182-3p	-0,13	-0,15
ZNF362	ENST00000539719.1	hsa-miR-182-3p	-0,13	-0,14
TCTA	ENST00000273590.3	hsa-miR-182-3p	-0,13	-0,15
GLCCI1	ENST00000223145.5	hsa-miR-182-3p	-0,13	-0,13
POTEG	ENST00000409832.3	hsa-miR-182-3p	-0,13	-0,13
ZNF582	ENST00000301310.4	hsa-miR-182-3p	-0,13	-0,13
GABRA4	ENST00000264318.3	hsa-miR-182-3p	-0,13	-0,13
POTEM	ENST00000551509.1	hsa-miR-182-3p	-0,13	-0,13
SRP72	ENST00000342756.5	hsa-miR-182-3p	-0,13	-0,19
MBP	ENST00000397863.1	hsa-miR-182-3p	-0,13	-0,15
TSKU	ENST00000333090.4	hsa-miR-182-3p	-0,13	-0,21
GRAP2	ENST00000344138.4	hsa-miR-182-3p	-0,13	-0,16
SF3B3	ENST00000302516.5	hsa-miR-182-3p	-0,13	-0,17
ARHGAP20	ENST00000260283.4	hsa-miR-182-3p	-0,13	-0,21
DNAJB12	ENST00000338820.3	hsa-miR-182-3p	-0,13	-0,13
TMEM213	ENST00000442682.2	hsa-miR-182-3p	-0,13	-0,13
MUC13	ENST00000311075.3	hsa-miR-182-3p	-0,13	-0,21
PTPN4	ENST00000263708.2	hsa-miR-182-3p	-0,13	-0,25
TBCEL	ENST00000422003.2	hsa-miR-182-3p	-0,13	-0,14
ZNF787	ENST00000270459.3	hsa-miR-182-3p	-0,12	-0,12
NIPAL4	ENST00000311946.7	hsa-miR-182-3p	-0,12	-0,12
UBE2D4	ENST00000222402.3	hsa-miR-182-3p	-0,12	-0,12
TRIM15	ENST00000376694.4	hsa-miR-182-3p	-0,12	-0,12
NAA15	ENST00000296543.5	hsa-miR-182-3p	-0,12	-0,12
SALL2	ENST00000327430.3	hsa-miR-182-3p	-0,12	-0,12
CASP14	ENST00000427043.3	hsa-miR-182-3p	-0,12	-0,12
NRK	ENST00000243300.9	hsa-miR-182-3p	-0,12	-0,12
RAB22A	ENST00000244040.3	hsa-miR-182-3p	-0,12	-0,22
NRIP1	ENST00000400199.1	hsa-miR-182-3p	-0,12	-0,13
ACSL1	ENST00000454703.2	hsa-miR-182-3p	-0,12	-0,14
KCNMB3	ENST00000497599.1	hsa-miR-182-3p	-0,12	-0,36
ARHGEF7	ENST00000426073.2	hsa-miR-182-3p	-0,12	-0,13
CMTM6	ENST00000205636.3	hsa-miR-182-3p	-0,12	-0,21
NBPF14	ENST00000369219.1	hsa-miR-182-3p	-0,12	-0,12
SLC25A25	ENST00000373069.5	hsa-miR-182-3p	-0,12	-0,12
CXCL5	ENST00000296027.4	hsa-miR-182-3p	-0,12	-0,12
WDFY1	ENST00000233055.4	hsa-miR-182-3p	-0,12	-0,12
ZNF532	ENST00000336078.4	hsa-miR-182-3p	-0,12	-0,12
SYP	ENST00000263233.4	hsa-miR-182-3p	-0,12	-0,12

FAM219B	ENST00000357635.5	hsa-miR-182-3p	-0,12	-0,16
POMGNT1	ENST00000396420.3	hsa-miR-182-3p	-0,12	-0,12
OTUD3	ENST00000375120.3	hsa-miR-182-3p	-0,12	-0,13
GATAD2A	ENST00000360315.3	hsa-miR-182-3p	-0,12	-0,12
MYLK	ENST00000360772.3	hsa-miR-182-3p	-0,12	-0,12
STON2	ENST00000267540.2	hsa-miR-182-3p	-0,12	-0,17
BCL2	ENST00000398117.1	hsa-miR-182-3p	-0,12	-0,14
ETV6	ENST00000396373.4	hsa-miR-182-3p	-0,12	-0,14
CDC42SE1	ENST00000439374.2	hsa-miR-182-3p	-0,12	-0,15
MARVELD1	ENST00000285605.6	hsa-miR-182-3p	-0,12	-0,12
SCD5	ENST00000319540.4	hsa-miR-182-3p	-0,12	-0,12
PURB	ENST00000395699.2	hsa-miR-182-3p	-0,12	-0,12
SERPINA11	ENST00000334708.3	hsa-miR-182-3p	-0,12	-0,12
GPAM	ENST00000348367.4	hsa-miR-182-3p	-0,12	-0,13
CREM	ENST00000333809.8	hsa-miR-182-3p	-0,12	-0,42
ACACA	ENST00000353139.5	hsa-miR-182-3p	-0,12	-0,16
CLIP4	ENST00000320081.5	hsa-miR-182-3p	-0,12	-0,14
PHKA1	ENST00000373542.4	hsa-miR-182-3p	-0,12	-0,17
ST3GAL5	ENST00000393808.3	hsa-miR-182-3p	-0,12	-0,12
FAM154B	ENST00000339465.5	hsa-miR-182-3p	-0,12	-0,12
ZNF672	ENST00000306562.3	hsa-miR-182-3p	-0,12	-0,12
IGFN1	ENST00000295591.8	hsa-miR-182-3p	-0,12	-0,12
LPGAT1	ENST00000366997.4	hsa-miR-182-3p	-0,12	-0,12
CCDC107	ENST00000421582.2	hsa-miR-182-3p	-0,12	-0,12
ZBTB40	ENST00000404138.1	hsa-miR-182-3p	-0,12	-0,12
DIO2	ENST00000438257.4	hsa-miR-182-3p	-0,12	-0,16
TRIM67	ENST00000444294.3	hsa-miR-182-3p	-0,12	-0,13
TEFM	ENST00000580840.1	hsa-miR-182-3p	-0,12	-0,13
ZMAT3	ENST00000311417.2	hsa-miR-182-3p	-0,12	-0,17
RAB5A	ENST00000273047.4	hsa-miR-182-3p	-0,12	-0,3
DKC1	ENST00000369550.5	hsa-miR-182-3p	-0,12	-0,12
ADRA2B	ENST00000409345.3	hsa-miR-182-3p	-0,12	-0,12
RASEF	ENST00000376447.3	hsa-miR-182-3p	-0,12	-0,12
ZC3H13	ENST00000242848.4	hsa-miR-182-3p	-0,12	-0,12
MAGI3	ENST00000307546.9	hsa-miR-182-3p	-0,12	-0,12
NARFL	ENST00000251588.2	hsa-miR-182-3p	-0,12	-0,12
SHPRH	ENST00000367505.2	hsa-miR-182-3p	-0,12	-0,12
HS6ST3	ENST00000376705.2	hsa-miR-182-3p	-0,12	-0,12
STK4	ENST00000372801.1	hsa-miR-182-3p	-0,12	-0,18
GRPEL1	ENST00000264954.4	hsa-miR-182-3p	-0,12	-0,35
PPP1R3B	ENST00000310455.3	hsa-miR-182-3p	-0,12	-0,2
C1orf189	ENST00000368525.3	hsa-miR-182-3p	-0,12	-0,3
GFI1	ENST00000370332.1	hsa-miR-182-3p	-0,12	-0,18
GMPS	ENST00000496455.2	hsa-miR-182-3p	-0,12	-0,13
CXorf36	ENST00000398000.2	hsa-miR-182-3p	-0,12	-0,12
MPZ	ENST00000533357.1	hsa-miR-182-3p	-0,12	-0,12
ZBTB22	ENST00000418724.1	hsa-miR-182-3p	-0,12	-0,12
SHOX2	ENST00000490689.2	hsa-miR-182-3p	-0,12	-0,12
MACC1	ENST00000400331.5	hsa-miR-182-3p	-0,12	-0,12
STARD4	ENST00000512160.1	hsa-miR-182-3p	-0,12	-0,16
MTMR7	ENST00000180173.5	hsa-miR-182-3p	-0,12	-0,16
ARSD	ENST00000381154.1	hsa-miR-182-3p	-0,12	-0,13
CYB5D1	ENST00000571846.1	hsa-miR-182-3p	-0,12	-0,14
CPEB1	ENST00000450751.2	hsa-miR-182-3p	-0,12	-0,12
MAP1LC3B	ENST00000268607.5	hsa-miR-182-3p	-0,12	-0,12
WNK1	ENST00000315939.6	hsa-miR-182-3p	-0,12	-0,15
PABPC1L2B	ENST00000373521.2	hsa-miR-182-3p	-0,12	-0,12
MMP16	ENST00000286614.6	hsa-miR-182-3p	-0,12	-0,12
RASA4B	ENST00000541662.1	hsa-miR-182-3p	-0,12	-0,12
JAG1	ENST00000254958.5	hsa-miR-182-3p	-0,12	-0,12
INPP5E	ENST00000371712.3	hsa-miR-182-3p	-0,12	-0,12
CCDC3	ENST00000378825.3	hsa-miR-182-3p	-0,12	-0,12
TMEM65	ENST00000297632.6	hsa-miR-182-3p	-0,12	-0,33
KLF3	ENST00000261438.5	hsa-miR-182-3p	-0,12	-0,18
RPL23	ENST00000479035.2	hsa-miR-182-3p	-0,12	-0,17
RRP7A	ENST00000323013.6	hsa-miR-182-3p	-0,12	-0,14

KLHL40	ENST00000287777.4	hsa-miR-182-3p	-0,12	-0,12
VAMP1	ENST00000361716.3	hsa-miR-182-3p	-0,12	-0,12
C15orf53	ENST00000318792.1	hsa-miR-182-3p	-0,12	-0,12
EYA3	ENST00000373871.3	hsa-miR-182-3p	-0,12	-0,12
ZNF746	ENST00000340622.3	hsa-miR-182-3p	-0,12	-0,12
CCR5	ENST00000292303.4	hsa-miR-182-3p	-0,12	-0,12
DCT	ENST00000377028.5	hsa-miR-182-3p	-0,12	-0,12
SLC39A7	ENST00000374675.3	hsa-miR-182-3p	-0,12	-0,12
GOT2	ENST00000245206.5	hsa-miR-182-3p	-0,12	-0,16
TMEM170B	ENST00000379426.1	hsa-miR-182-3p	-0,12	-0,16
GPHN	ENST00000478722.1	hsa-miR-182-3p	-0,12	-0,12
CATSPER4	ENST00000456354.2	hsa-miR-182-3p	-0,12	-0,12
IRG1	ENST00000377462.1	hsa-miR-182-3p	-0,12	-0,12
LHX1	ENST00000254457.5	hsa-miR-182-3p	-0,12	-0,12
THAP11	ENST00000303596.1	hsa-miR-182-3p	-0,12	-0,12
ACSL6	ENST00000379264.2	hsa-miR-182-3p	-0,12	-0,15
DDA1	ENST00000359866.4	hsa-miR-182-3p	-0,12	-0,13
MMP19	ENST00000409200.3	hsa-miR-182-3p	-0,12	-0,12
HLA-DPB1	ENST00000418931.2	hsa-miR-182-3p	-0,12	-0,18
RAD51L3-RFFL	ENST00000593039.1	hsa-miR-182-3p	-0,12	-0,14
DNMT3B	ENST00000344505.4	hsa-miR-182-3p	-0,12	-0,12
ANKRD23	ENST00000318357.4	hsa-miR-182-3p	-0,12	-0,12
ZSCAN12	ENST00000361028.1	hsa-miR-182-3p	-0,12	-0,12
E2F4	ENST00000379378.3	hsa-miR-182-3p	-0,12	-0,12
KLHL23	ENST00000392647.2	hsa-miR-182-3p	-0,12	-0,14
EPHB2	ENST00000374632.3	hsa-miR-182-3p	-0,12	-0,12
F2RL1	ENST00000296677.4	hsa-miR-182-3p	-0,12	-0,12
RDX	ENST00000343115.4	hsa-miR-182-3p	-0,12	-0,12
FAM199X	ENST00000493442.1	hsa-miR-182-3p	-0,11	-0,12
PEX19	ENST00000368072.5	hsa-miR-182-3p	-0,11	-0,11
PLEKHG7	ENST00000344636.3	hsa-miR-182-3p	-0,11	-0,11
ESRRG	ENST00000361525.3	hsa-miR-182-3p	-0,11	-0,11
NBPF10	ENST00000369339.3	hsa-miR-182-3p	-0,11	-0,11
KRAS	ENST00000256078.4	hsa-miR-182-3p	-0,11	-0,33
PHF17	ENST00000226319.6	hsa-miR-182-3p	-0,11	-0,11
SYNE1	ENST00000367255.5	hsa-miR-182-3p	-0,11	-0,11
MYH9	ENST00000216181.5	hsa-miR-182-3p	-0,11	-0,14
FAM47E	ENST00000515604.1	hsa-miR-182-3p	-0,11	-0,14
CCNF	ENST00000397066.4	hsa-miR-182-3p	-0,11	-0,12
GTPBP1	ENST00000216044.5	hsa-miR-182-3p	-0,11	-0,12
RFWD3	ENST00000361070.4	hsa-miR-182-3p	-0,11	-0,15
FKBP5	ENST00000536438.1	hsa-miR-182-3p	-0,11	-0,2
SH3BP4	ENST00000392011.2	hsa-miR-182-3p	-0,11	-0,11
NCOA2	ENST00000452400.2	hsa-miR-182-3p	-0,11	-0,11
CAMKK2	ENST00000538733.1	hsa-miR-182-3p	-0,11	-0,12
NBPF3	ENST00000318220.6	hsa-miR-182-3p	-0,11	-0,11
KPNA6	ENST00000373625.3	hsa-miR-182-3p	-0,11	-0,13
PLA2G2D	ENST00000375105.3	hsa-miR-182-3p	-0,11	-0,11
NBPF16	ENST00000417839.1	hsa-miR-182-3p	-0,11	-0,11
NBPF15	ENST00000442702.2	hsa-miR-182-3p	-0,11	-0,11
SFRP1	ENST00000220772.3	hsa-miR-182-3p	-0,11	-0,25
DESI2	ENST00000302550.11	hsa-miR-182-3p	-0,11	-0,11
MTR	ENST00000366577.5	hsa-miR-182-3p	-0,11	-0,13
XXYLT1	ENST00000310380.6	hsa-miR-182-3p	-0,11	-0,11
INADL	ENST00000371158.2	hsa-miR-182-3p	-0,11	-0,11
NAA30	ENST00000556492.1	hsa-miR-182-3p	-0,11	-0,27
ASB16	ENST00000293414.1	hsa-miR-182-3p	-0,11	-0,11
GPR124	ENST00000315215.7	hsa-miR-182-3p	-0,11	-0,11
ROBO1	ENST00000436010.2	hsa-miR-182-3p	-0,11	-0,11
PNPLA8	ENST00000426128.2	hsa-miR-182-3p	-0,11	-0,21
COL25A1	ENST00000399132.1	hsa-miR-182-3p	-0,11	-0,12
BLOC1S6	ENST00000220531.3	hsa-miR-182-3p	-0,11	-0,11
EMR2	ENST00000315576.3	hsa-miR-182-3p	-0,11	-0,25
CHIT1	ENST00000367229.1	hsa-miR-182-3p	-0,11	-0,11
SCLY	ENST00000254663.6	hsa-miR-182-3p	-0,11	-0,11
EVX2	ENST00000308618.4	hsa-miR-182-3p	-0,11	-0,11

CS	ENST00000548567.1	hsa-miR-182-3p	-0,11	-0,11
C15orf54	ENST00000318578.3	hsa-miR-182-3p	-0,11	-0,11
DGKB	ENST00000403951.2	hsa-miR-182-3p	-0,11	-0,11
CHRM5	ENST00000383263.5	hsa-miR-182-3p	-0,11	-0,11
ADAMTS18	ENST00000282849.5	hsa-miR-182-3p	-0,11	-0,11
KLF9	ENST00000377126.2	hsa-miR-182-3p	-0,11	-0,17
FKBP14	ENST00000222803.5	hsa-miR-182-3p	-0,11	-0,12
GPR126	ENST00000230173.6	hsa-miR-182-3p	-0,11	-0,11
ELL2	ENST00000237853.4	hsa-miR-182-3p	-0,11	-0,12
TSPAN14	ENST00000429989.3	hsa-miR-182-3p	-0,11	-0,15
PREPL	ENST00000541738.1	hsa-miR-182-3p	-0,11	-0,14
ANO3	ENST00000256737.3	hsa-miR-182-3p	-0,11	-0,11
ATP13A1	ENST00000291503.5	hsa-miR-182-3p	-0,11	-0,11
RBM12	ENST00000374114.3	hsa-miR-182-3p	-0,11	-0,11
ZNF142	ENST00000449707.1	hsa-miR-182-3p	-0,11	-0,11
ATP13A3	ENST00000439040.1	hsa-miR-182-3p	-0,11	-0,11
ARMC1	ENST00000276569.3	hsa-miR-182-3p	-0,11	-0,13
TIMM8B	ENST00000504148.2	hsa-miR-182-3p	-0,11	-0,27
NOTCH2	ENST00000256646.2	hsa-miR-182-3p	-0,11	-0,12
LRRC2	ENST00000395905.3	hsa-miR-182-3p	-0,11	-0,11
ZNF491	ENST00000323169.5	hsa-miR-182-3p	-0,11	-0,11
CREBZF	ENST00000398294.2	hsa-miR-182-3p	-0,11	-0,24
CPNE3	ENST00000198765.4	hsa-miR-182-3p	-0,11	-0,11
LILRA1	ENST00000453777.1	hsa-miR-182-3p	-0,11	-0,11
MTERF	ENST00000419292.1	hsa-miR-182-3p	-0,11	-0,11
FSD2	ENST00000334574.8	hsa-miR-182-3p	-0,11	-0,11
PRUNE	ENST00000271620.3	hsa-miR-182-3p	-0,11	-0,11
FCRL1	ENST00000368176.3	hsa-miR-182-3p	-0,11	-0,11
PRICKLE3	ENST00000376317.3	hsa-miR-182-3p	-0,11	-0,31
NANOS2	ENST00000341294.2	hsa-miR-182-3p	-0,11	-0,11
NBPF9	ENST00000440491.2	hsa-miR-182-3p	-0,11	-0,11
PRDM14	ENST00000276594.2	hsa-miR-182-3p	-0,11	-0,11
PHLPP2	ENST00000568954.1	hsa-miR-182-3p	-0,11	-0,11
XIRP1	ENST00000396251.1	hsa-miR-182-3p	-0,11	-0,11
KLHL42	ENST00000381271.2	hsa-miR-182-3p	-0,11	-0,11
UBN1	ENST00000262376.6	hsa-miR-182-3p	-0,11	-0,11
ARHGAP28	ENST00000419673.2	hsa-miR-182-3p	-0,11	-0,15
TTC9	ENST00000256367.2	hsa-miR-182-3p	-0,11	-0,13
C16orf52	ENST00000542527.2	hsa-miR-182-3p	-0,11	-0,14
FBXW11	ENST00000296933.6	hsa-miR-182-3p	-0,11	-0,11
KIAA0586	ENST00000423743.3	hsa-miR-182-3p	-0,11	-0,17
CALU	ENST00000535011.2	hsa-miR-182-3p	-0,11	-0,17
MBD1	ENST00000591416.1	hsa-miR-182-3p	-0,11	-0,12
H6PD	ENST00000377403.2	hsa-miR-182-3p	-0,11	-0,11
UNC5D	ENST00000287272.2	hsa-miR-182-3p	-0,11	-0,13
HIST1H2BD	ENST00000289316.2	hsa-miR-182-3p	-0,11	-0,26
NEK1	ENST00000439128.2	hsa-miR-182-3p	-0,11	-0,11
SLCO4C1	ENST00000310954.6	hsa-miR-182-3p	-0,11	-0,11
NOS1AP	ENST00000361897.5	hsa-miR-182-3p	-0,11	-0,11
HLTF	ENST00000465259.1	hsa-miR-182-3p	-0,11	-0,11
PPP3R2	ENST00000374806.1	hsa-miR-182-3p	-0,11	-0,11
SERINC3	ENST00000342374.4	hsa-miR-182-3p	-0,11	-0,16
SDE2	ENST00000272091.7	hsa-miR-182-3p	-0,11	-0,13
KIAA1737	ENST00000361786.2	hsa-miR-182-3p	-0,11	-0,12
PPFIA2	ENST00000549396.1	hsa-miR-182-3p	-0,11	-0,11
ACVR1C	ENST00000243349.8	hsa-miR-182-3p	-0,11	-0,11
UBE2QL1	ENST00000399816.3	hsa-miR-182-3p	-0,11	-0,23
SEMA6D	ENST00000355997.3	hsa-miR-182-3p	-0,11	-0,11
DOK6	ENST00000382713.5	hsa-miR-182-3p	-0,11	-0,11
TMPRSS3	ENST00000398405.1	hsa-miR-182-3p	-0,11	-0,11
FAM115C	ENST00000441159.2	hsa-miR-182-3p	-0,11	-0,11
PAX8	ENST00000263335.7	hsa-miR-182-3p	-0,11	-0,11
S1PR2	ENST00000590320.1	hsa-miR-182-3p	-0,11	-0,11
FOXP1	ENST00000318789.4	hsa-miR-182-3p	-0,11	-0,19
ATPAF1	ENST00000576409.1	hsa-miR-182-3p	-0,11	-0,12
ARL5B	ENST00000377275.3	hsa-miR-182-3p	-0,11	-0,28

ORAI2	ENST00000356387.2	hsa-miR-182-3p	-0,11	-0,14
EPGN	ENST00000413830.1	hsa-miR-182-3p	-0,11	-0,25
DHRS12	ENST00000218981.1	hsa-miR-182-3p	-0,1	-0,1
RASGRF1	ENST00000558480.2	hsa-miR-182-3p	-0,1	-0,1
SMPD3	ENST00000219334.5	hsa-miR-182-3p	-0,1	-0,1
PTGER3	ENST00000370924.4	hsa-miR-182-3p	-0,1	-0,1
PLEKHA8	ENST00000449726.1	hsa-miR-182-3p	-0,1	-0,11
ZNF449	ENST00000339249.4	hsa-miR-182-3p	-0,1	-0,11
BCAT1	ENST00000261192.7	hsa-miR-182-3p	-0,1	-0,11
RAB30	ENST00000533486.1	hsa-miR-182-3p	-0,1	-0,16
PAX2	ENST00000370296.2	hsa-miR-182-3p	-0,1	-0,1
OTUD5	ENST00000156084.4	hsa-miR-182-3p	-0,1	-0,1
STXBP5L	ENST00000273666.6	hsa-miR-182-3p	-0,1	-0,1
PAK7	ENST00000378423.1	hsa-miR-182-3p	-0,1	-0,1
TAF1	ENST00000373790.4	hsa-miR-182-3p	-0,1	-0,1
UNC119B	ENST00000344651.4	hsa-miR-182-3p	-0,1	-0,1
KIF1B	ENST00000377086.1	hsa-miR-182-3p	-0,1	-0,1
CEBPE	ENST00000206513.5	hsa-miR-182-3p	-0,1	-0,1
HIF1AN	ENST00000299163.6	hsa-miR-182-3p	-0,1	-0,16
ETNK2	ENST00000367201.3	hsa-miR-182-3p	-0,1	-0,1
ZNF592	ENST00000299927.3	hsa-miR-182-3p	-0,1	-0,12
ARHGAP17	ENST00000441763.2	hsa-miR-182-3p	-0,1	-0,1
POLR3F	ENST00000377603.4	hsa-miR-182-3p	-0,1	-0,12
TRAK2	ENST00000332624.3	hsa-miR-182-3p	-0,1	-0,16
RAD54B	ENST00000297592.5	hsa-miR-182-3p	-0,1	-0,13
UBE3C	ENST00000348165.5	hsa-miR-182-3p	-0,1	-0,1
ATP10D	ENST00000273859.3	hsa-miR-182-3p	-0,1	-0,1
B3GNT6	ENST00000533140.1	hsa-miR-182-3p	-0,1	-0,1
EREG	ENST00000244869.2	hsa-miR-182-3p	-0,1	-0,1
IL6R	ENST00000344086.4	hsa-miR-182-3p	-0,1	-0,11
UBXN2A	ENST00000309033.4	hsa-miR-182-3p	-0,1	-0,14
SLC41A1	ENST00000367137.3	hsa-miR-182-3p	-0,1	-0,1
NARG2	ENST00000261520.4	hsa-miR-182-3p	-0,1	-0,16
NLRP10	ENST00000328600.2	hsa-miR-182-3p	-0,1	-0,14
MYO5A	ENST00000399231.3	hsa-miR-182-3p	-0,1	-0,12
ADAM19	ENST00000257527.4	hsa-miR-182-3p	-0,1	-0,1
SIAH3	ENST00000400405.2	hsa-miR-182-3p	-0,1	-0,1
PPP2R2D	ENST00000422256.2	hsa-miR-182-3p	-0,1	-0,18
FLVCR2	ENST00000238667.4	hsa-miR-182-3p	-0,1	-0,1
ENPEP	ENST00000265162.5	hsa-miR-182-3p	-0,1	-0,16
MTSS1	ENST00000378017.3	hsa-miR-182-3p	-0,1	-0,2
MOCS3	ENST00000244051.1	hsa-miR-182-3p	-0,1	-0,11
NMNAT2	ENST00000294868.4	hsa-miR-182-3p	-0,1	-0,1
TNFAIP3	ENST00000237289.4	hsa-miR-182-3p	-0,1	-0,1
RBPJ	ENST00000504907.1	hsa-miR-182-3p	-0,1	-0,1
AC079210.1	ENST00000600820.1	hsa-miR-182-3p	-0,1	-0,1
CNST	ENST00000366513.4	hsa-miR-182-3p	-0,1	-0,1
KLF12	ENST00000377669.2	hsa-miR-182-3p	-0,1	-0,11
KPNB1	ENST00000290158.4	hsa-miR-182-3p	-0,1	-0,2
LIX1L	ENST00000369308.3	hsa-miR-182-3p	-0,1	-0,12
METTL8	ENST00000375258.4	hsa-miR-182-3p	-0,1	-0,16
CA12	ENST00000178638.3	hsa-miR-182-3p	-0,1	-0,23
AGPAT4	ENST00000366911.5	hsa-miR-182-3p	-0,1	-0,19
HOXB9	ENST00000311177.5	hsa-miR-182-3p	-0,1	-0,17
TPP2	ENST00000376052.3	hsa-miR-182-3p	-0,1	-0,18
TMEM194A	ENST00000379391.3	hsa-miR-182-3p	-0,1	-0,11
ANAPC10	ENST00000507656.1	hsa-miR-182-3p	-0,1	-0,2
NBPF1	ENST00000430580.2	hsa-miR-182-3p	-0,1	-0,1
SLC5A1	ENST00000266088.4	hsa-miR-182-3p	-0,1	-0,1
ARL14EPL	ENST00000601302.2	hsa-miR-182-3p	-0,1	-0,21
PCSK5	ENST00000376752.4	hsa-miR-182-3p	-0,1	-0,1
DCHS1	ENST00000299441.3	hsa-miR-182-3p	-0,1	-0,1
MAK16	ENST00000360128.6	hsa-miR-182-3p	-0,1	-0,13
TATDN3	ENST00000526997.1	hsa-miR-182-3p	-0,1	-0,38
P4HB	ENST00000331483.4	hsa-miR-182-3p	-0,1	-0,1
WDR37	ENST00000358220.1	hsa-miR-182-3p	-0,1	-0,18

C10orf105	ENST00000441508.2	hsa-miR-182-3p	-0,1	-0,1
PEX5L	ENST00000467460.1	hsa-miR-182-3p	-0,1	-0,1
NBPF12	ENST00000446760.2	hsa-miR-182-3p	-0,1	-0,1
ENDOD1	ENST00000278505.4	hsa-miR-182-3p	-0,1	-0,2
WNT8B	ENST00000343737.5	hsa-miR-182-3p	-0,1	-0,12
BMPR1A	ENST00000372037.3	hsa-miR-182-3p	-0,1	-0,28
ALOX15B	ENST00000380183.4	hsa-miR-182-3p	-0,1	-0,1
CYP19A1	ENST00000396402.1	hsa-miR-182-3p	-0,1	-0,1
KCNJ16	ENST00000589377.1	hsa-miR-182-3p	-0,1	-0,1
SHROOM4	ENST00000376020.2	hsa-miR-182-3p	-0,1	-0,1
SNRK	ENST00000429705.2	hsa-miR-182-3p	-0,1	-0,1
FAM124A	ENST00000322475.8	hsa-miR-182-3p	-0,1	-0,1
E2F3	ENST00000346618.3	hsa-miR-182-3p	-0,1	-0,1
NBPF11	ENST00000604938.1	hsa-miR-182-3p	-0,1	-0,1
PTCD1	ENST00000292478.4	hsa-miR-182-3p	-0,1	-0,11
GNPDA1	ENST00000311337.6	hsa-miR-182-3p	-0,1	-0,1
YPEL1	ENST00000339468.3	hsa-miR-182-3p	-0,1	-0,18
GPATCH2L	ENST00000553588.1	hsa-miR-182-3p	-0,1	-0,11
B3GNT4	ENST00000546192.1	hsa-miR-182-3p	-0,1	-0,12
FAM86A	ENST00000458008.4	hsa-miR-182-3p	-0,1	-0,1
TIGIT	ENST00000486257.1	hsa-miR-182-3p	-0,1	-0,1
KLHL29	ENST00000486442.1	hsa-miR-182-3p	-0,1	-0,1
RIMBP3C	ENST00000331505.5	hsa-miR-182-3p	-0,1	-0,1
HS3ST1	ENST00000002596.5	hsa-miR-182-3p	-0,1	-0,18
CD96	ENST00000352690.4	hsa-miR-182-3p	-0,1	-0,1
ZNF831	ENST00000371030.2	hsa-miR-182-3p	-0,1	-0,1
FAM161B	ENST00000286544.3	hsa-miR-182-3p	-0,1	-0,1
ZNF525	ENST00000467003.1	hsa-miR-182-3p	-0,1	-0,1
SOCS2	ENST00000548537.1	hsa-miR-182-3p	-0,1	-0,1
TMEM239	ENST00000361033.1	hsa-miR-182-3p	-0,1	-0,1
LDLRAD4	ENST00000399848.3	hsa-miR-182-3p	-0,1	-0,17
PRLR	ENST00000342362.5	hsa-miR-182-3p	-0,1	-0,14
MID2	ENST00000262843.6	hsa-miR-182-3p	-0,1	-0,12
CTNNA3	ENST00000433211.2	hsa-miR-182-3p	-0,1	-0,1
CACHD1	ENST00000371073.2	hsa-miR-182-3p	-0,1	-0,1
ALDH1A3	ENST00000329841.5	hsa-miR-182-3p	-0,1	-0,1
MRPL3	ENST00000264995.3	hsa-miR-182-3p	-0,1	-0,31
FUT1	ENST00000310160.3	hsa-miR-182-3p	-0,1	-0,1
KLHL15	ENST00000328046.8	hsa-miR-182-3p	-0,1	-0,2
ZRANB1	ENST00000359653.4	hsa-miR-182-3p	-0,1	-0,1
SLC25A44	ENST00000359511.4	hsa-miR-182-3p	-0,1	-0,1
FOXN3	ENST00000345097.4	hsa-miR-182-3p	-0,1	-0,2
DENR	ENST00000280557.6	hsa-miR-182-3p	-0,1	-0,14
DAP	ENST00000230895.6	hsa-miR-182-3p	-0,1	-0,38
SORBS3	ENST00000240123.7	hsa-miR-182-3p	-0,1	-0,1
POTEI	ENST00000451531.2	hsa-miR-182-3p	-0,1	-0,1
NIN	ENST00000389868.3	hsa-miR-182-3p	-0,1	-0,1
DNAJC16	ENST00000375847.3	hsa-miR-182-3p	-0,1	-0,1
GDF7	ENST00000272224.3	hsa-miR-182-3p	-0,1	-0,1
ERBB2IP	ENST00000284037.5	hsa-miR-182-3p	-0,1	-0,14
KCNA7	ENST00000221444.1	hsa-miR-182-3p	-0,1	-0,1
SLK	ENST00000335753.4	hsa-miR-182-3p	-0,09	-0,14
ARHGEF12	ENST00000397843.2	hsa-miR-182-3p	-0,09	-0,11
C6orf141	ENST00000529246.2	hsa-miR-182-3p	-0,09	-0,15
MDH1B	ENST00000374412.3	hsa-miR-182-3p	-0,09	-0,25
NAV2	ENST00000360655.4	hsa-miR-182-3p	-0,09	-0,1
RPS9	ENST00000441429.1	hsa-miR-182-3p	-0,09	-0,09
TSHZ1	ENST00000322038.5	hsa-miR-182-3p	-0,09	-0,09
SEC14L5	ENST00000251170.7	hsa-miR-182-3p	-0,09	-0,09
KCNS2	ENST00000287042.4	hsa-miR-182-3p	-0,09	-0,09
CNTN5	ENST00000524871.1	hsa-miR-182-3p	-0,09	-0,09
MRVI1	ENST00000421747.1	hsa-miR-182-3p	-0,09	-0,14
PLCXD1	ENST00000381657.2	hsa-miR-182-3p	-0,09	-0,09
KANSL3	ENST00000431828.1	hsa-miR-182-3p	-0,09	-0,09
TRIM5	ENST00000396847.3	hsa-miR-182-3p	-0,09	-0,09
KLLN	ENST00000445946.3	hsa-miR-182-3p	-0,09	-0,09

RFFL	ENST00000315249.7	hsa-miR-182-3p	-0,09	-0,11
DCP1A	ENST00000607628.1	hsa-miR-182-3p	-0,09	-0,11
SCD	ENST00000370355.2	hsa-miR-182-3p	-0,09	-0,09
XRN1	ENST00000264951.4	hsa-miR-182-3p	-0,09	-0,09
PMEPA1	ENST00000341744.3	hsa-miR-182-3p	-0,09	-0,16
PERP	ENST00000421351.3	hsa-miR-182-3p	-0,09	-0,37
SLC16A7	ENST00000261187.4	hsa-miR-182-3p	-0,09	-0,28
FRRS1L	ENST00000561981.2	hsa-miR-182-3p	-0,09	-0,09
FSTL1	ENST00000295633.3	hsa-miR-182-3p	-0,09	-0,1
SH3BP5L	ENST00000366472.5	hsa-miR-182-3p	-0,09	-0,09
C1orf204	ENST00000368102.1	hsa-miR-182-3p	-0,09	-0,09
SMOC2	ENST00000356284.2	hsa-miR-182-3p	-0,09	-0,09
KIF2A	ENST00000381103.2	hsa-miR-182-3p	-0,09	-0,32
C9orf40	ENST00000376854.5	hsa-miR-182-3p	-0,09	-0,13
ZBED3	ENST00000255198.2	hsa-miR-182-3p	-0,09	-0,24
TPMT	ENST00000309983.4	hsa-miR-182-3p	-0,09	-0,13
SRPK1	ENST00000373825.2	hsa-miR-182-3p	-0,09	-0,61
WDR36	ENST00000506538.2	hsa-miR-182-3p	-0,09	-0,13
RAB41	ENST00000374473.2	hsa-miR-182-3p	-0,09	-1
LMAN1	ENST00000251047.5	hsa-miR-182-3p	-0,09	-0,11
AK4	ENST00000545314.1	hsa-miR-182-3p	-0,09	-0,09
MYOM3	ENST00000338909.5	hsa-miR-182-3p	-0,09	-0,09
RCVRN	ENST00000226193.5	hsa-miR-182-3p	-0,09	-0,09
ABCG1	ENST00000398457.2	hsa-miR-182-3p	-0,09	-0,09
TRPV1	ENST00000399759.3	hsa-miR-182-3p	-0,09	-0,09
ADAMTSL1	ENST00000380548.4	hsa-miR-182-3p	-0,09	-0,12
POLG	ENST00000268124.5	hsa-miR-182-3p	-0,09	-0,09
SEC62	ENST00000337002.4	hsa-miR-182-3p	-0,09	-0,32
CCDC81	ENST00000528728.1	hsa-miR-182-3p	-0,09	-0,09
TRIM23	ENST00000231524.9	hsa-miR-182-3p	-0,09	-0,09
SHPK	ENST00000572705.1	hsa-miR-182-3p	-0,09	-0,09
ATP5J2-PTCD1	ENST00000413834.1	hsa-miR-182-3p	-0,09	-0,14
MIEF1	ENST00000325301.2	hsa-miR-182-3p	-0,09	-0,12
LMBR1	ENST00000353442.5	hsa-miR-182-3p	-0,09	-0,14
PSEN2	ENST00000340188.4	hsa-miR-182-3p	-0,09	-0,09
AGL	ENST00000370161.2	hsa-miR-182-3p	-0,09	-0,09
CLSTN3	ENST00000266546.6	hsa-miR-182-3p	-0,09	-0,09
BRPF3	ENST00000534400.1	hsa-miR-182-3p	-0,09	-0,09
DLC1	ENST00000276297.4	hsa-miR-182-3p	-0,09	-0,12
C4orf3	ENST00000399075.4	hsa-miR-182-3p	-0,09	-0,14
C1RL	ENST00000544702.1	hsa-miR-182-3p	-0,09	-0,12
YRDC	ENST00000373044.2	hsa-miR-182-3p	-0,09	-0,22
C14orf132	ENST00000555004.1	hsa-miR-182-3p	-0,09	-0,22
ZCWPW2	ENST00000383768.2	hsa-miR-182-3p	-0,09	-0,19
MKL2	ENST00000318282.5	hsa-miR-182-3p	-0,09	-0,09
LRCH1	ENST00000311191.6	hsa-miR-182-3p	-0,09	-0,09
ABCC10	ENST00000372530.4	hsa-miR-182-3p	-0,09	-0,09
CDHR1	ENST00000372117.3	hsa-miR-182-3p	-0,09	-0,09
RASD2	ENST00000216127.4	hsa-miR-182-3p	-0,09	-0,09
PRMT8	ENST00000382622.3	hsa-miR-182-3p	-0,09	-0,09
ERGIC1	ENST00000393784.3	hsa-miR-182-3p	-0,09	-0,09
CIT	ENST00000392521.2	hsa-miR-182-3p	-0,09	-0,1
ONECUT2	ENST00000491143.2	hsa-miR-182-3p	-0,09	-0,09
C5orf47	ENST00000340147.6	hsa-miR-182-3p	-0,09	-0,29
LAMP2	ENST00000371335.4	hsa-miR-182-3p	-0,09	-0,09
MYH15	ENST00000273353.3	hsa-miR-182-3p	-0,09	-0,09
TAP1	ENST00000354258.4	hsa-miR-182-3p	-0,09	-0,09
C10orf53	ENST00000374112.3	hsa-miR-182-3p	-0,09	-0,09
NHSL2	ENST00000540800.1	hsa-miR-182-3p	-0,09	-0,09
ZNF646	ENST00000394979.2	hsa-miR-182-3p	-0,09	-0,09
C17orf105	ENST00000449302.3	hsa-miR-182-3p	-0,09	-0,09
EMILIN3	ENST00000332312.3	hsa-miR-182-3p	-0,09	-0,09
OVOL1	ENST00000335987.3	hsa-miR-182-3p	-0,09	-0,09
CCNJ	ENST00000265992.5	hsa-miR-182-3p	-0,09	-0,09
COL14A1	ENST00000297848.3	hsa-miR-182-3p	-0,09	-0,11
NACC2	ENST00000371753.1	hsa-miR-182-3p	-0,09	-0,13

CHST11	ENST00000549260.1	hsa-miR-182-3p	-0,09	-0,1
ZNF641	ENST00000301042.3	hsa-miR-182-3p	-0,09	-0,16
FGF11	ENST00000293829.4	hsa-miR-182-3p	-0,09	-0,17
RBM48	ENST00000481551.1	hsa-miR-182-3p	-0,09	-0,28
TCF20	ENST00000359486.3	hsa-miR-182-3p	-0,09	-0,09
KCTD9	ENST00000221200.4	hsa-miR-182-3p	-0,09	-0,09
LCP2	ENST00000046794.5	hsa-miR-182-3p	-0,09	-0,09
RAB3B	ENST00000371655.3	hsa-miR-182-3p	-0,09	-0,11
FSBP	ENST00000481490.2	hsa-miR-182-3p	-0,09	-0,11
ZZEF1	ENST00000381638.2	hsa-miR-182-3p	-0,09	-0,1
SEZ6L	ENST00000360929.3	hsa-miR-182-3p	-0,09	-0,09
SLC6A8	ENST00000253122.5	hsa-miR-182-3p	-0,09	-0,09
KIF5C	ENST00000435030.1	hsa-miR-182-3p	-0,09	-0,09
FGD6	ENST00000343958.4	hsa-miR-182-3p	-0,09	-0,12
PSD3	ENST00000327040.8	hsa-miR-182-3p	-0,09	-0,1
UBE2K	ENST00000261427.5	hsa-miR-182-3p	-0,09	-0,32
CASP2	ENST00000310447.5	hsa-miR-182-3p	-0,09	-0,09
FAM168A	ENST00000064778.4	hsa-miR-182-3p	-0,09	-0,09
SAMD4A	ENST00000392067.3	hsa-miR-182-3p	-0,09	-0,1
FBLN7	ENST00000331203.2	hsa-miR-182-3p	-0,09	-0,23
SEC61A1	ENST00000243253.3	hsa-miR-182-3p	-0,09	-0,09
HMBOX1	ENST00000397358.3	hsa-miR-182-3p	-0,09	-0,09
CDHR5	ENST00000397542.2	hsa-miR-182-3p	-0,09	-0,09
TMEM215	ENST00000342743.5	hsa-miR-182-3p	-0,09	-0,09
HELLS	ENST00000394036.1	hsa-miR-182-3p	-0,09	-0,32
POLR3D	ENST00000397802.4	hsa-miR-182-3p	-0,09	-0,09
LYN	ENST00000520220.2	hsa-miR-182-3p	-0,09	-0,2
DPH2	ENST00000255108.3	hsa-miR-182-3p	-0,09	-0,15
KPNA4	ENST00000334256.4	hsa-miR-182-3p	-0,09	-0,12
SPARC	ENST00000231061.4	hsa-miR-182-3p	-0,09	-0,09
CLTC	ENST00000269122.3	hsa-miR-182-3p	-0,09	-0,34
ZNF609	ENST00000326648.3	hsa-miR-182-3p	-0,09	-0,09
PCSK1	ENST00000311106.3	hsa-miR-182-3p	-0,09	-0,2
CCDC12	ENST00000425441.1	hsa-miR-182-3p	-0,09	-0,09
CD28	ENST00000324106.8	hsa-miR-182-3p	-0,09	-0,09
LANCL3	ENST00000378621.3	hsa-miR-182-3p	-0,08	-0,27
GAREML	ENST00000401533.2	hsa-miR-182-3p	-0,08	-0,09
DNAJC18	ENST00000302060.5	hsa-miR-182-3p	-0,08	-0,31
DIXDC1	ENST00000440460.2	hsa-miR-182-3p	-0,08	-0,15
RPE	ENST00000429907.1	hsa-miR-182-3p	-0,08	-0,09
ENTPD5	ENST00000334696.6	hsa-miR-182-3p	-0,08	-0,09
FZD8	ENST00000374694.1	hsa-miR-182-3p	-0,08	-0,21
AGTR2	ENST00000371906.4	hsa-miR-182-3p	-0,08	-0,08
GPR176	ENST00000299092.3	hsa-miR-182-3p	-0,08	-0,08
RNASEL	ENST00000367559.3	hsa-miR-182-3p	-0,08	-0,08
AGXT	ENST00000307503.3	hsa-miR-182-3p	-0,08	-0,08
FGF5	ENST00000456523.3	hsa-miR-182-3p	-0,08	-0,38
NFYA	ENST00000341376.6	hsa-miR-182-3p	-0,08	-0,12
ZNF462	ENST00000277225.5	hsa-miR-182-3p	-0,08	-0,1
MYOCD	ENST00000425538.1	hsa-miR-182-3p	-0,08	-0,12
SMAD3	ENST00000327367.4	hsa-miR-182-3p	-0,08	-0,11
CACNA1D	ENST00000288139.4	hsa-miR-182-3p	-0,08	-0,18
TECPR2	ENST00000359520.7	hsa-miR-182-3p	-0,08	-0,08
RNF44	ENST00000274811.4	hsa-miR-182-3p	-0,08	-0,08
ZNF638	ENST00000355812.3	hsa-miR-182-3p	-0,08	-0,08
GABBR2	ENST00000259455.2	hsa-miR-182-3p	-0,08	-0,08
KLHL18	ENST00000232766.5	hsa-miR-182-3p	-0,08	-0,08
AC002472.13	ENST00000543388.1	hsa-miR-182-3p	-0,08	-0,08
MOCS2	ENST00000450852.3	hsa-miR-182-3p	-0,08	-0,39
DGKE	ENST00000284061.3	hsa-miR-182-3p	-0,08	-0,11
NME6	ENST00000450160.1	hsa-miR-182-3p	-0,08	-0,34
CLIC5	ENST00000339561.6	hsa-miR-182-3p	-0,08	-0,11
WBP1L	ENST00000369889.4	hsa-miR-182-3p	-0,08	-0,08
GABRG1	ENST00000295452.4	hsa-miR-182-3p	-0,08	-0,08
TAF1D	ENST00000448108.2	hsa-miR-182-3p	-0,08	-0,11
JMY	ENST00000396137.4	hsa-miR-182-3p	-0,08	-0,09

RASSF6	ENST00000307439.5	hsa-miR-182-3p	-0,08	-0,18
ZBTB37	ENST00000367701.5	hsa-miR-182-3p	-0,08	-0,09
TRHDE	ENST00000261180.4	hsa-miR-182-3p	-0,08	-0,15
GPR153	ENST00000377893.2	hsa-miR-182-3p	-0,08	-0,08
SRL	ENST00000399609.3	hsa-miR-182-3p	-0,08	-0,08
KCNJ5	ENST00000529694.1	hsa-miR-182-3p	-0,08	-0,08
VAV2	ENST00000406606.3	hsa-miR-182-3p	-0,08	-0,14
KDM4B	ENST00000159111.4	hsa-miR-182-3p	-0,08	-0,08
APOL2	ENST00000358502.5	hsa-miR-182-3p	-0,08	-0,2
PDK1	ENST00000282077.3	hsa-miR-182-3p	-0,08	-0,28
IL7R	ENST00000343305.4	hsa-miR-182-3p	-0,08	-0,08
BBS9	ENST00000242067.6	hsa-miR-182-3p	-0,08	-0,21
MED13L	ENST00000281928.3	hsa-miR-182-3p	-0,08	-0,11
CNOT6	ENST00000393356.1	hsa-miR-182-3p	-0,08	-0,08
KIAA1244	ENST00000251691.4	hsa-miR-182-3p	-0,08	-0,1
GOSR2	ENST00000576910.2	hsa-miR-182-3p	-0,08	-0,22
TGFBR3	ENST00000212355.4	hsa-miR-182-3p	-0,08	-0,08
ZNF74	ENST00000357502.5	hsa-miR-182-3p	-0,08	-0,08
CLSTN2	ENST00000458420.3	hsa-miR-182-3p	-0,08	-0,09
FAM211A	ENST00000409083.3	hsa-miR-182-3p	-0,08	-0,14
GPRC5B	ENST00000300571.2	hsa-miR-182-3p	-0,08	-0,08
ZNF827	ENST00000379448.4	hsa-miR-182-3p	-0,08	-0,08
TTC5	ENST00000258821.3	hsa-miR-182-3p	-0,08	-0,12
RREB1	ENST00000379938.2	hsa-miR-182-3p	-0,08	-0,08
NOC3L	ENST00000371361.3	hsa-miR-182-3p	-0,08	-0,08
ASIC1	ENST00000228468.4	hsa-miR-182-3p	-0,08	-0,08
PPP4R2	ENST00000356692.5	hsa-miR-182-3p	-0,08	-0,08
C1orf216	ENST00000270815.4	hsa-miR-182-3p	-0,08	-0,08
AC132186.1	ENST00000544589.1	hsa-miR-182-3p	-0,08	-0,08
KRTAP4-9	ENST00000391415.1	hsa-miR-182-3p	-0,08	-0,16
CEMP1	ENST00000382350.1	hsa-miR-182-3p	-0,08	-0,08
TANGO6	ENST00000261778.1	hsa-miR-182-3p	-0,08	-0,12
FER	ENST00000281092.4	hsa-miR-182-3p	-0,08	-0,08
GNS	ENST00000418919.2	hsa-miR-182-3p	-0,08	-0,09
KCTD2	ENST00000322444.6	hsa-miR-182-3p	-0,08	-0,08
ADNP2	ENST00000262198.4	hsa-miR-182-3p	-0,08	-0,1
SEMA5A	ENST00000382496.5	hsa-miR-182-3p	-0,08	-0,1
CALM3	ENST00000291295.9	hsa-miR-182-3p	-0,08	-0,12
SIM1	ENST00000369208.3	hsa-miR-182-3p	-0,08	-0,08
AAGAB	ENST00000261880.5	hsa-miR-182-3p	-0,08	-0,27
MOB1A	ENST00000396049.4	hsa-miR-182-3p	-0,08	-0,15
BICD1	ENST00000548411.1	hsa-miR-182-3p	-0,08	-0,09
CDYL2	ENST00000570137.2	hsa-miR-182-3p	-0,08	-0,1
MPP6	ENST00000222644.5	hsa-miR-182-3p	-0,08	-0,15
RBM39	ENST00000253363.6	hsa-miR-182-3p	-0,08	-0,18
NKX3-1	ENST00000380871.4	hsa-miR-182-3p	-0,08	-0,08
RAD54L2	ENST00000409535.2	hsa-miR-182-3p	-0,08	-0,08
CTC-432M15.3	ENST00000514667.1	hsa-miR-182-3p	-0,08	-0,08
ANKRD13C	ENST00000370944.4	hsa-miR-182-3p	-0,08	-0,28
FFAR4	ENST00000371481.4	hsa-miR-182-3p	-0,08	-0,08
TNPO2	ENST00000425528.1	hsa-miR-182-3p	-0,08	-0,08
PSD4	ENST00000441564.3	hsa-miR-182-3p	-0,08	-0,1
ORC2	ENST00000234296.2	hsa-miR-182-3p	-0,08	-0,09
ZNF347	ENST00000334197.7	hsa-miR-182-3p	-0,08	-0,08
ERAP1	ENST00000443439.2	hsa-miR-182-3p	-0,08	-0,08
USP49	ENST00000394253.3	hsa-miR-182-3p	-0,08	-0,22
LARS	ENST00000394434.2	hsa-miR-182-3p	-0,08	-0,29
ARHGAP19	ENST00000358531.4	hsa-miR-182-3p	-0,08	-0,08
LRRTM4	ENST00000409911.1	hsa-miR-182-3p	-0,08	-0,08
SLC26A4	ENST00000265715.3	hsa-miR-182-3p	-0,08	-0,08
PSD2	ENST00000274710.3	hsa-miR-182-3p	-0,08	-0,08
FRAS1	ENST00000264895.6	hsa-miR-182-3p	-0,08	-0,08
IRF4	ENST00000380956.4	hsa-miR-182-3p	-0,08	-0,08
MED7	ENST00000286317.5	hsa-miR-182-3p	-0,08	-0,38
ETS1	ENST00000531611.1	hsa-miR-182-3p	-0,08	-0,08
KCNAB3	ENST00000303790.2	hsa-miR-182-3p	-0,08	-0,18

AGFG1	ENST00000310078.8	hsa-miR-182-3p	-0,08	-0,08
PRKCA	ENST00000413366.3	hsa-miR-182-3p	-0,08	-0,14
FAM126B	ENST00000418596.3	hsa-miR-182-3p	-0,08	-0,08
RRM2B	ENST00000251810.3	hsa-miR-182-3p	-0,07	-0,07
GJD3	ENST00000578689.1	hsa-miR-182-3p	-0,07	-0,07
DLG5	ENST00000372391.2	hsa-miR-182-3p	-0,07	-0,07
FAXDC2	ENST00000326080.5	hsa-miR-182-3p	-0,07	-0,07
RPS6KA2	ENST00000265678.4	hsa-miR-182-3p	-0,07	-0,07
PCF11	ENST00000298281.4	hsa-miR-182-3p	-0,07	-0,11
LAD1	ENST00000391967.2	hsa-miR-182-3p	-0,07	-0,24
PLAA	ENST00000397292.3	hsa-miR-182-3p	-0,07	-0,16
IPCEF1	ENST00000265198.4	hsa-miR-182-3p	-0,07	-0,07
SYPL2	ENST00000369872.3	hsa-miR-182-3p	-0,07	-0,07
GPR68	ENST00000531499.2	hsa-miR-182-3p	-0,07	-0,07
FLT1	ENST00000282397.4	hsa-miR-182-3p	-0,07	-0,07
ACTN2	ENST00000366578.4	hsa-miR-182-3p	-0,07	-0,17
RTKN2	ENST00000373789.3	hsa-miR-182-3p	-0,07	-0,07
FAM69A	ENST00000370310.4	hsa-miR-182-3p	-0,07	-0,17
OLA1	ENST00000284719.3	hsa-miR-182-3p	-0,07	-0,16
SLC25A45	ENST00000527174.1	hsa-miR-182-3p	-0,07	-0,09
LRRFIP2	ENST00000421307.1	hsa-miR-182-3p	-0,07	-0,15
RFTN2	ENST00000295049.4	hsa-miR-182-3p	-0,07	-0,12
GID4	ENST00000268719.4	hsa-miR-182-3p	-0,07	-0,16
DOCK3	ENST00000266037.9	hsa-miR-182-3p	-0,07	-0,07
NECAP1	ENST00000339754.5	hsa-miR-182-3p	-0,07	-0,07
PLIN4	ENST00000301286.3	hsa-miR-182-3p	-0,07	-0,07
YPEL2	ENST00000312655.4	hsa-miR-182-3p	-0,07	-0,07
FAM155A	ENST00000375915.2	hsa-miR-182-3p	-0,07	-0,12
KCNH8	ENST00000328405.2	hsa-miR-182-3p	-0,07	-0,07
SULT2A1	ENST00000222002.3	hsa-miR-182-3p	-0,07	-0,1
PNPLA3	ENST00000216180.3	hsa-miR-182-3p	-0,07	-0,09
USP13	ENST00000263966.3	hsa-miR-182-3p	-0,07	-0,08
AP1S3	ENST00000396654.2	hsa-miR-182-3p	-0,07	-0,24
ACER2	ENST00000340967.2	hsa-miR-182-3p	-0,07	-0,08
FZR1	ENST00000441788.2	hsa-miR-182-3p	-0,07	-0,08
DIEXF	ENST00000491415.2	hsa-miR-182-3p	-0,07	-0,1
SNX29	ENST00000566228.1	hsa-miR-182-3p	-0,07	-0,07
ABCC12	ENST00000416054.1	hsa-miR-182-3p	-0,07	-0,07
ITPKC	ENST00000263370.2	hsa-miR-182-3p	-0,07	-0,07
CD300E	ENST00000392619.1	hsa-miR-182-3p	-0,07	-0,07
HHAT	ENST00000413764.2	hsa-miR-182-3p	-0,07	-0,07
KIAA0319	ENST00000537886.1	hsa-miR-182-3p	-0,07	-0,1
CYB5B	ENST00000512062.1	hsa-miR-182-3p	-0,07	-0,08
GATAD1	ENST00000287957.3	hsa-miR-182-3p	-0,07	-0,14
RACGAP1	ENST00000427314.2	hsa-miR-182-3p	-0,07	-0,13
GNAL	ENST00000334049.6	hsa-miR-182-3p	-0,07	-0,11
MAP2	ENST00000360351.4	hsa-miR-182-3p	-0,07	-0,1
VTI1B	ENST00000554659.1	hsa-miR-182-3p	-0,07	-0,13
POM121	ENST00000395270.1	hsa-miR-182-3p	-0,07	-0,07
BLOC1S2	ENST00000441611.1	hsa-miR-182-3p	-0,07	-0,15
CSNK1A1	ENST00000261798.5	hsa-miR-182-3p	-0,07	-0,2
SULT1B1	ENST00000310613.3	hsa-miR-182-3p	-0,07	-0,07
GYS1	ENST00000323798.3	hsa-miR-182-3p	-0,07	-0,07
POU5F1B	ENST00000465342.2	hsa-miR-182-3p	-0,07	-0,07
TOX4	ENST00000405508.1	hsa-miR-182-3p	-0,07	-0,1
MPRIP	ENST00000341712.4	hsa-miR-182-3p	-0,07	-0,09
GPR107	ENST00000372410.3	hsa-miR-182-3p	-0,07	-0,11
BRD7	ENST00000394688.3	hsa-miR-182-3p	-0,07	-0,14
TMC5	ENST00000381414.4	hsa-miR-182-3p	-0,07	-0,08
LIN7A	ENST00000552864.1	hsa-miR-182-3p	-0,07	-0,2
ZNRF3	ENST00000544604.2	hsa-miR-182-3p	-0,07	-0,08
CACNA1E	ENST00000526775.1	hsa-miR-182-3p	-0,07	-0,07
CXXC4	ENST00000394767.2	hsa-miR-182-3p	-0,07	-0,08
CNTNAP5	ENST00000431078.1	hsa-miR-182-3p	-0,07	-0,07
LPHN3	ENST00000512091.2	hsa-miR-182-3p	-0,07	-0,07
PCDH17	ENST00000377918.3	hsa-miR-182-3p	-0,07	-0,16

RAPGEF6	ENST00000509018.1	hsa-miR-182-3p	-0,07	-0,07
DCUN1D3	ENST00000324344.4	hsa-miR-182-3p	-0,07	-0,11
MOCOS	ENST00000261326.5	hsa-miR-182-3p	-0,07	-0,1
SERBP1	ENST00000370994.4	hsa-miR-182-3p	-0,07	-0,08
FREM1	ENST00000380881.4	hsa-miR-182-3p	-0,07	-0,09
LINC00346	ENST00000538077.1	hsa-miR-182-3p	-0,07	-0,12
KBTBD8	ENST00000295568.4	hsa-miR-182-3p	-0,07	-0,07
PRPH2	ENST00000230381.5	hsa-miR-182-3p	-0,07	-0,07
SORD	ENST00000267814.9	hsa-miR-182-3p	-0,07	-0,07
AC005477.1	ENST00000542853.1	hsa-miR-182-3p	-0,07	-0,07
HRH1	ENST00000397056.1	hsa-miR-182-3p	-0,07	-0,07
SEC31B	ENST00000370345.3	hsa-miR-182-3p	-0,07	-0,07
ANKRD34A	ENST00000323397.4	hsa-miR-182-3p	-0,07	-0,07
LHX6	ENST00000373755.2	hsa-miR-182-3p	-0,07	-0,07
GCH1	ENST00000491895.2	hsa-miR-182-3p	-0,07	-0,07
GSTO2	ENST00000369707.2	hsa-miR-182-3p	-0,07	-0,13
SLC8A3	ENST00000356921.2	hsa-miR-182-3p	-0,07	-0,07
SGSM1	ENST00000400358.4	hsa-miR-182-3p	-0,07	-0,07
DACH1	ENST00000305425.4	hsa-miR-182-3p	-0,07	-0,07
GRIN1	ENST00000371561.3	hsa-miR-182-3p	-0,07	-0,07
GPR158	ENST00000376351.3	hsa-miR-182-3p	-0,07	-0,07
RBM8A	ENST00000330165.8	hsa-miR-182-3p	-0,07	-0,07
ATG10	ENST00000282185.3	hsa-miR-182-3p	-0,07	-0,12
CD180	ENST00000256447.4	hsa-miR-182-3p	-0,07	-0,16
RNF14	ENST00000394520.2	hsa-miR-182-3p	-0,07	-0,09
ASAH2	ENST00000395526.4	hsa-miR-182-3p	-0,07	-0,07
DLGAP2	ENST00000421627.2	hsa-miR-182-3p	-0,07	-0,07
CHRM2	ENST00000445907.2	hsa-miR-182-3p	-0,07	-0,07
TMEM132D	ENST00000389441.4	hsa-miR-182-3p	-0,07	-0,07
GLG1	ENST00000422840.2	hsa-miR-182-3p	-0,07	-0,07
CNTN2	ENST00000331830.4	hsa-miR-182-3p	-0,07	-0,07
IFNE	ENST00000448696.3	hsa-miR-182-3p	-0,07	-0,24
HNRNPU	ENST00000444376.2	hsa-miR-182-3p	-0,07	-0,18
TULP4	ENST00000367094.2	hsa-miR-182-3p	-0,07	-0,08
COLGALT1	ENST00000252599.4	hsa-miR-182-3p	-0,07	-0,08
ZBTB39	ENST00000300101.2	hsa-miR-182-3p	-0,07	-0,07
ERCC4	ENST00000311895.7	hsa-miR-182-3p	-0,07	-0,07
KLHL6	ENST00000341319.3	hsa-miR-182-3p	-0,07	-0,07
RUNX3	ENST00000399916.1	hsa-miR-182-3p	-0,07	-0,07
CPED1	ENST00000310396.5	hsa-miR-182-3p	-0,07	-0,07
KREMEN1	ENST00000400335.4	hsa-miR-182-3p	-0,07	-0,07
FHDC1	ENST00000260008.3	hsa-miR-182-3p	-0,07	-0,07
ZC3H6	ENST00000343936.4	hsa-miR-182-3p	-0,07	-0,07
FAM3C	ENST00000359943.3	hsa-miR-182-3p	-0,07	-0,07
SAMD8	ENST00000372687.4	hsa-miR-182-3p	-0,07	-0,14
ZNF799	ENST00000419318.1	hsa-miR-182-3p	-0,07	-0,09
CYTIP	ENST00000264192.3	hsa-miR-182-3p	-0,07	-0,11
CYP2U1	ENST00000332884.6	hsa-miR-182-3p	-0,07	-0,07
FUT11	ENST00000394790.1	hsa-miR-182-3p	-0,07	-0,07
RIPPLY3	ENST00000329553.2	hsa-miR-182-3p	-0,07	-0,07
ITGA11	ENST00000423218.2	hsa-miR-182-3p	-0,07	-0,18
FBXO22	ENST00000308275.3	hsa-miR-182-3p	-0,07	-0,24
AKNA	ENST00000307564.4	hsa-miR-182-3p	-0,07	-0,07
RPS6KA6	ENST00000262752.2	hsa-miR-182-3p	-0,06	-0,07
GLI3	ENST00000395925.3	hsa-miR-182-3p	-0,06	-0,07
PDPK1	ENST00000441549.3	hsa-miR-182-3p	-0,06	-0,07
ALS2	ENST00000457679.2	hsa-miR-182-3p	-0,06	-0,07
CALML4	ENST00000395465.3	hsa-miR-182-3p	-0,06	-1,05
NR4A3	ENST00000330847.1	hsa-miR-182-3p	-0,06	-0,12
SLC4A8	ENST00000453097.2	hsa-miR-182-3p	-0,06	-0,09
FADS1	ENST00000350997.7	hsa-miR-182-3p	-0,06	-0,2
UNC13C	ENST00000545554.1	hsa-miR-182-3p	-0,06	-0,06
IFI44L	ENST00000370751.5	hsa-miR-182-3p	-0,06	-0,06
LL22NC03-63E9.3	ENST00000407120.1	hsa-miR-182-3p	-0,06	-0,06
PLEKHG4B	ENST00000283426.6	hsa-miR-182-3p	-0,06	-0,06
SPN	ENST00000360121.3	hsa-miR-182-3p	-0,06	-0,06

NEURL1B	ENST00000369800.5	hsa-miR-182-3p	-0,06	-0,06
KIAA1644	ENST00000381176.4	hsa-miR-182-3p	-0,06	-0,09
OSBPL3	ENST00000313367.2	hsa-miR-182-3p	-0,06	-0,11
PDE8A	ENST00000310298.4	hsa-miR-182-3p	-0,06	-0,09
GABPB2	ENST00000368918.3	hsa-miR-182-3p	-0,06	-0,09
STARD5	ENST00000302824.6	hsa-miR-182-3p	-0,06	-0,27
RAB8B	ENST00000321437.4	hsa-miR-182-3p	-0,06	-0,07
DTL	ENST00000366991.4	hsa-miR-182-3p	-0,06	-0,07
UNC5C	ENST00000453304.1	hsa-miR-182-3p	-0,06	-0,08
ZNF852	ENST00000436261.1	hsa-miR-182-3p	-0,06	-0,08
SCAMP5	ENST00000425597.3	hsa-miR-182-3p	-0,06	-0,06
KSR2	ENST00000425217.1	hsa-miR-182-3p	-0,06	-0,06
TAS2R5	ENST00000247883.4	hsa-miR-182-3p	-0,06	-0,18
FOXK2	ENST00000335255.5	hsa-miR-182-3p	-0,06	-0,17
TAF8	ENST00000372977.3	hsa-miR-182-3p	-0,06	-0,09
POLR3H	ENST00000396504.2	hsa-miR-182-3p	-0,06	-0,06
KLF13	ENST00000307145.3	hsa-miR-182-3p	-0,06	-0,06
ZBTB43	ENST00000449886.1	hsa-miR-182-3p	-0,06	-0,06
MET	ENST00000397752.3	hsa-miR-182-3p	-0,06	-0,06
PITPNM3	ENST00000421306.3	hsa-miR-182-3p	-0,06	-0,06
UBE2Q2	ENST00000267938.4	hsa-miR-182-3p	-0,06	-0,42
JHDM1D	ENST00000397560.2	hsa-miR-182-3p	-0,06	-0,07
PEX26	ENST00000329627.7	hsa-miR-182-3p	-0,06	-0,13
OXTR	ENST00000316793.3	hsa-miR-182-3p	-0,06	-0,1
HNRNPA1	ENST00000546500.1	hsa-miR-182-3p	-0,06	-0,06
GPR161	ENST00000367838.1	hsa-miR-182-3p	-0,06	-0,07
PSEN1	ENST00000344094.3	hsa-miR-182-3p	-0,06	-0,1
MYNN	ENST00000544106.1	hsa-miR-182-3p	-0,06	-0,21
PNO1	ENST00000263657.2	hsa-miR-182-3p	-0,06	-0,27
WDPCP	ENST00000272321.7	hsa-miR-182-3p	-0,06	-0,08
FAF1	ENST00000396153.2	hsa-miR-182-3p	-0,06	-0,15
ST8SIA2	ENST00000268164.3	hsa-miR-182-3p	-0,06	-0,06
FSTL3	ENST00000166139.4	hsa-miR-182-3p	-0,06	-0,15
FAM120AOS	ENST00000423591.1	hsa-miR-182-3p	-0,06	-0,09
SCML2	ENST00000398048.3	hsa-miR-182-3p	-0,06	-0,28
ZIC4	ENST00000383075.3	hsa-miR-182-3p	-0,06	-0,06
ACAP2	ENST00000326793.6	hsa-miR-182-3p	-0,06	-0,07
UBR1	ENST00000382177.2	hsa-miR-182-3p	-0,06	-0,14
RORA	ENST00000335670.6	hsa-miR-182-3p	-0,06	-0,07
SLC5A12	ENST00000396005.3	hsa-miR-182-3p	-0,06	-0,1
PCMTD1	ENST00000360540.5	hsa-miR-182-3p	-0,06	-0,06
PANK3	ENST00000239231.6	hsa-miR-182-3p	-0,06	-0,16
ADD1	ENST00000398125.1	hsa-miR-182-3p	-0,06	-0,06
MINA	ENST00000333396.7	hsa-miR-182-3p	-0,06	-0,18
PPP1R12B	ENST00000608999.1	hsa-miR-182-3p	-0,06	-0,08
CCDC176	ENST00000394009.3	hsa-miR-182-3p	-0,06	-0,06
SLX4IP	ENST00000334534.5	hsa-miR-182-3p	-0,06	-0,06
FDX1	ENST00000260270.2	hsa-miR-182-3p	-0,06	-0,32
NCOA1	ENST00000405141.1	hsa-miR-182-3p	-0,06	-0,06
TYRO3	ENST00000263798.3	hsa-miR-182-3p	-0,06	-0,09
EPHA5	ENST00000273854.3	hsa-miR-182-3p	-0,06	-0,09
STEAP3	ENST00000409811.1	hsa-miR-182-3p	-0,06	-0,16
MED13	ENST00000397786.2	hsa-miR-182-3p	-0,06	-0,09
GSTM4	ENST00000369833.1	hsa-miR-182-3p	-0,06	-0,06
MTUS1	ENST00000381869.3	hsa-miR-182-3p	-0,06	-0,06
KCND3	ENST00000369697.1	hsa-miR-182-3p	-0,06	-0,06
ZNF747	ENST00000395094.3	hsa-miR-182-3p	-0,06	-0,11
FAM20B	ENST00000263733.4	hsa-miR-182-3p	-0,06	-0,08
GMEB1	ENST00000373816.1	hsa-miR-182-3p	-0,06	-0,17
DGKI	ENST00000453654.2	hsa-miR-182-3p	-0,06	-0,19
ADAMTS5	ENST00000284987.5	hsa-miR-182-3p	-0,06	-0,14
ZNF230	ENST00000429154.2	hsa-miR-182-3p	-0,06	-0,19
ENTPD1	ENST00000371207.3	hsa-miR-182-3p	-0,06	-0,09
DUOX2	ENST00000389039.6	hsa-miR-182-3p	-0,06	-0,06
EZH1	ENST00000428826.2	hsa-miR-182-3p	-0,06	-0,06
ABCG2	ENST00000515655.1	hsa-miR-182-3p	-0,06	-0,17

TMPPE	ENST00000416695.2	hsa-miR-182-3p	-0,06	-0,23
STS	ENST00000217961.4	hsa-miR-182-3p	-0,06	-0,07
SYNPO2	ENST00000429713.2	hsa-miR-182-3p	-0,06	-0,1
MTIF2	ENST00000403721.1	hsa-miR-182-3p	-0,06	-0,14
FBXO46	ENST00000317683.3	hsa-miR-182-3p	-0,06	-0,06
ADAMTS17	ENST00000268070.4	hsa-miR-182-3p	-0,06	-0,06
ZBTB10	ENST00000430430.1	hsa-miR-182-3p	-0,06	-0,11
MAVS	ENST00000428216.2	hsa-miR-182-3p	-0,06	-0,08
NR2C2	ENST00000425241.1	hsa-miR-182-3p	-0,06	-0,06
TOMM40	ENST00000592434.1	hsa-miR-182-3p	-0,06	-0,08
GOPC	ENST00000368498.2	hsa-miR-182-3p	-0,06	-0,11
CARNS1	ENST00000307823.3	hsa-miR-182-3p	-0,06	-0,06
CALN1	ENST00000329008.5	hsa-miR-182-3p	-0,06	-0,06
PKDREJ	ENST00000253255.5	hsa-miR-182-3p	-0,06	-0,06
ADAMTS14	ENST00000373208.1	hsa-miR-182-3p	-0,06	-0,06
TP63	ENST00000392460.3	hsa-miR-182-3p	-0,06	-0,06
ZNF26	ENST00000328654.5	hsa-miR-182-3p	-0,06	-0,19
CTBP2	ENST00000337195.5	hsa-miR-182-3p	-0,06	-0,17
CAB39L	ENST00000355854.4	hsa-miR-182-3p	-0,06	-0,14
ZNF497	ENST00000311044.3	hsa-miR-182-3p	-0,06	-0,06
AMER3	ENST00000423981.1	hsa-miR-182-3p	-0,06	-0,06
LYST	ENST00000389794.3	hsa-miR-182-3p	-0,06	-0,06
NBPF20	ENST00000369202.1	hsa-miR-182-3p	-0,06	-0,06
RRP1B	ENST00000340648.4	hsa-miR-182-3p	-0,05	-0,06
ADAR	ENST00000292205.5	hsa-miR-182-3p	-0,05	-0,06
FAM175A	ENST00000321945.7	hsa-miR-182-3p	-0,05	-0,15
CD99L2	ENST00000370377.3	hsa-miR-182-3p	-0,05	-0,06
DAB1	ENST00000371236.2	hsa-miR-182-3p	-0,05	-0,08
SH3PXD2A	ENST00000369774.4	hsa-miR-182-3p	-0,05	-0,06
COG3	ENST00000349995.5	hsa-miR-182-3p	-0,05	-0,05
TFE3	ENST00000315869.7	hsa-miR-182-3p	-0,05	-0,05
KIAA1199	ENST00000220244.3	hsa-miR-182-3p	-0,05	-0,05
ATG12	ENST00000500945.2	hsa-miR-182-3p	-0,05	-0,2
EIF4E3	ENST00000425534.3	hsa-miR-182-3p	-0,05	-0,21
ACVR2B	ENST00000352511.4	hsa-miR-182-3p	-0,05	-0,11
TSHZ2	ENST00000371497.5	hsa-miR-182-3p	-0,05	-0,05
PAG1	ENST00000220597.4	hsa-miR-182-3p	-0,05	-0,06
HELZ2	ENST00000427522.2	hsa-miR-182-3p	-0,05	-0,05
MRI1	ENST00000319545.8	hsa-miR-182-3p	-0,05	-0,05
PPP4R1L	ENST00000334187.8	hsa-miR-182-3p	-0,05	-0,07
EOGT	ENST00000383701.3	hsa-miR-182-3p	-0,05	-0,25
GMDS	ENST00000530927.1	hsa-miR-182-3p	-0,05	-0,17
PADI2	ENST00000375486.4	hsa-miR-182-3p	-0,05	-0,12
RNMT	ENST00000383314.2	hsa-miR-182-3p	-0,05	-0,08
AUTS2	ENST00000342771.4	hsa-miR-182-3p	-0,05	-0,08
TELO2	ENST00000262319.6	hsa-miR-182-3p	-0,05	-0,05
XIAP	ENST00000371199.3	hsa-miR-182-3p	-0,05	-0,08
ZNF629	ENST00000262525.4	hsa-miR-182-3p	-0,05	-0,05
SLC8A2	ENST00000236877.6	hsa-miR-182-3p	-0,05	-0,05
CRLF1	ENST00000392386.3	hsa-miR-182-3p	-0,05	-0,05
FRMPD4	ENST00000380682.1	hsa-miR-182-3p	-0,05	-0,05
GPR110	ENST00000371253.2	hsa-miR-182-3p	-0,05	-0,05
ZBTB8B	ENST00000609129.1	hsa-miR-182-3p	-0,05	-0,07
ZNF460	ENST00000360338.3	hsa-miR-182-3p	-0,05	-0,07
DTWD2	ENST00000304058.4	hsa-miR-182-3p	-0,05	-0,07
TET3	ENST00000409262.3	hsa-miR-182-3p	-0,05	-0,05
MBNL3	ENST00000370839.3	hsa-miR-182-3p	-0,05	-0,06
C9orf91	ENST00000374049.4	hsa-miR-182-3p	-0,05	-0,15
BTBD7	ENST00000334746.5	hsa-miR-182-3p	-0,05	-0,06
PARVA	ENST00000334956.8	hsa-miR-182-3p	-0,05	-0,15
DHDDS	ENST00000360009.2	hsa-miR-182-3p	-0,05	-0,1
RPP14	ENST00000445193.3	hsa-miR-182-3p	-0,05	-0,18
GALNT2	ENST00000366672.4	hsa-miR-182-3p	-0,05	-0,05
HIF3A	ENST00000377670.4	hsa-miR-182-3p	-0,05	-0,05
HPCAL4	ENST00000372844.3	hsa-miR-182-3p	-0,05	-0,05
MAP4	ENST00000383737.4	hsa-miR-182-3p	-0,05	-0,05

NAIP	ENST00000517649.1	hsa-miR-182-3p	-0,05	-0,05
IMPAD1	ENST00000262644.4	hsa-miR-182-3p	-0,05	-0,1
ZC3H12C	ENST00000278590.3	hsa-miR-182-3p	-0,05	-0,06
PRKAR2A	ENST00000265563.8	hsa-miR-182-3p	-0,05	-0,05
PPFIBP1	ENST00000318304.8	hsa-miR-182-3p	-0,05	-0,09
SAP30L	ENST00000297109.6	hsa-miR-182-3p	-0,05	-0,13
DNAJA2	ENST00000317089.5	hsa-miR-182-3p	-0,05	-0,14
EPT1	ENST00000260585.7	hsa-miR-182-3p	-0,05	-0,06
ZKSCAN3	ENST00000377255.3	hsa-miR-182-3p	-0,05	-0,23
PHF21B	ENST00000403565.1	hsa-miR-182-3p	-0,05	-0,05
PLXNB2	ENST00000449103.1	hsa-miR-182-3p	-0,05	-0,05
ERBB4	ENST00000342788.4	hsa-miR-182-3p	-0,05	-0,05
CCNJL	ENST00000393977.3	hsa-miR-182-3p	-0,05	-0,08
GRIN2B	ENST00000609686.1	hsa-miR-182-3p	-0,05	-0,05
FGF19	ENST00000294312.3	hsa-miR-182-3p	-0,05	-0,12
MSL2	ENST00000309993.2	hsa-miR-182-3p	-0,05	-0,13
API5	ENST00000534600.1	hsa-miR-182-3p	-0,05	-0,17
NLRC5	ENST00000436936.1	hsa-miR-182-3p	-0,05	-0,05
MREG	ENST00000263268.6	hsa-miR-182-3p	-0,05	-0,07
EDC3	ENST00000315127.4	hsa-miR-182-3p	-0,05	-0,15
SENP2	ENST00000296257.5	hsa-miR-182-3p	-0,05	-0,17
CREB3L2	ENST00000330387.6	hsa-miR-182-3p	-0,05	-0,05
GAN	ENST00000568107.2	hsa-miR-182-3p	-0,05	-0,08
PPP6C	ENST00000373547.4	hsa-miR-182-3p	-0,05	-0,18
ZNF585B	ENST00000532828.2	hsa-miR-182-3p	-0,05	-0,08
CYTH1	ENST00000585509.1	hsa-miR-182-3p	-0,05	-0,05
IKZF4	ENST00000262032.5	hsa-miR-182-3p	-0,05	-0,05
PML	ENST00000565898.1	hsa-miR-182-3p	-0,05	-0,05
TFCP2L1	ENST00000263707.5	hsa-miR-182-3p	-0,05	-0,05
UBN2	ENST00000473989.3	hsa-miR-182-3p	-0,05	-0,05
KNOP1	ENST00000219837.7	hsa-miR-182-3p	-0,05	-0,09
ACSM3	ENST00000289416.5	hsa-miR-182-3p	-0,05	-0,17
CACNA1G	ENST00000352832.5	hsa-miR-182-3p	-0,05	-0,11
TTBK2	ENST00000267890.6	hsa-miR-182-3p	-0,05	-0,05
NCOA3	ENST00000341724.6	hsa-miR-182-3p	-0,05	-0,06
RFX3	ENST00000382004.3	hsa-miR-182-3p	-0,05	-0,07
ALG1	ENST00000262374.5	hsa-miR-182-3p	-0,05	-0,09
SYT2	ENST00000367267.1	hsa-miR-182-3p	-0,05	-0,05
PPM1L	ENST00000498165.1	hsa-miR-182-3p	-0,05	-0,09
WNK3	ENST00000375169.3	hsa-miR-182-3p	-0,05	-0,05
DNM3	ENST00000367731.1	hsa-miR-182-3p	-0,05	-0,05
DCTN4	ENST00000447998.2	hsa-miR-182-3p	-0,05	-0,2
SCRN1	ENST00000242059.5	hsa-miR-182-3p	-0,05	-0,05
IGF2BP2	ENST00000382199.2	hsa-miR-182-3p	-0,05	-0,05
NADK2	ENST00000397338.1	hsa-miR-182-3p	-0,05	-0,19
RAB3GAP2	ENST00000358951.2	hsa-miR-182-3p	-0,05	-0,09
PPP2R1B	ENST00000527614.1	hsa-miR-182-3p	-0,05	-0,25
PGM2L1	ENST00000298198.4	hsa-miR-182-3p	-0,05	-0,1
KIAA1432	ENST00000414202.2	hsa-miR-182-3p	-0,05	-0,05
ADSS	ENST00000366535.3	hsa-miR-182-3p	-0,05	-0,27
RIOK2	ENST00000283109.3	hsa-miR-182-3p	-0,05	-0,35
FUT10	ENST00000327671.5	hsa-miR-182-3p	-0,05	-0,17
QRSL1	ENST00000369046.4	hsa-miR-182-3p	-0,05	-0,09
AMBRA1	ENST00000314845.3	hsa-miR-182-3p	-0,05	-0,05
ZNF502	ENST00000296091.4	hsa-miR-182-3p	-0,05	-0,05
REL	ENST00000295025.8	hsa-miR-182-3p	-0,05	-0,05
SYT13	ENST00000020926.3	hsa-miR-182-3p	-0,05	-0,05
NSG2	ENST00000303177.3	hsa-miR-182-3p	-0,05	-0,05
MOCS1	ENST00000373186.4	hsa-miR-182-3p	-0,05	-0,05
SPAG16	ENST00000432529.2	hsa-miR-182-3p	-0,05	-0,29
AMZ2	ENST00000577273.1	hsa-miR-182-3p	-0,05	-0,33
MYEF2	ENST00000324324.7	hsa-miR-182-3p	-0,05	-0,09
DUSP3	ENST00000226004.3	hsa-miR-182-3p	-0,05	-0,11
CERS6	ENST00000305747.6	hsa-miR-182-3p	-0,05	-0,06
PKIA	ENST00000396418.2	hsa-miR-182-3p	-0,05	-0,98
RPP40	ENST00000380051.2	hsa-miR-182-3p	-0,05	-0,48

USP22	ENST00000261497.4	hsa-miR-182-3p	-0,05	-0,05
SESTD1	ENST00000428443.3	hsa-miR-182-3p	-0,05	-0,35
PRDM15	ENST00000422911.1	hsa-miR-182-3p	-0,05	-0,05
GDPD5	ENST00000443276.2	hsa-miR-182-3p	-0,05	-0,05
PKD2	ENST00000237596.2	hsa-miR-182-3p	-0,05	-0,05
DST	ENST00000312431.6	hsa-miR-182-3p	-0,05	-0,05
GIGYF2	ENST00000373566.3	hsa-miR-182-3p	-0,05	-0,11
DYRK1A	ENST00000339659.4	hsa-miR-182-3p	-0,05	-0,07
RP11-315D16.2	ENST00000562767.1	hsa-miR-182-3p	-0,05	-0,34
EFR3B	ENST00000403714.3	hsa-miR-182-3p	-0,05	-0,07
THBS2	ENST00000366787.3	hsa-miR-182-3p	-0,05	-0,13
ELF4	ENST00000335997.7	hsa-miR-182-3p	-0,04	-0,04
FAM102B	ENST00000370035.3	hsa-miR-182-3p	-0,04	-0,04
TTC33	ENST00000337702.4	hsa-miR-182-3p	-0,04	-0,26
C1QTNF8	ENST00000328449.5	hsa-miR-182-3p	-0,04	-0,18
TIMM8A	ENST00000372902.3	hsa-miR-182-3p	-0,04	-0,16
TRABD2B	ENST00000606738.2	hsa-miR-182-3p	-0,04	-0,06
NEK8	ENST00000268766.6	hsa-miR-182-3p	-0,04	-0,05
ATF6	ENST00000367942.3	hsa-miR-182-3p	-0,04	-0,27
PSTPIP2	ENST00000409746.5	hsa-miR-182-3p	-0,04	-0,04
SOX5	ENST00000546136.1	hsa-miR-182-3p	-0,04	-0,14
HIST1H2AK	ENST00000330180.2	hsa-miR-182-3p	-0,04	-0,21
KLHL9	ENST00000359039.4	hsa-miR-182-3p	-0,04	-0,05
BCL2L2	ENST00000250405.5	hsa-miR-182-3p	-0,04	-0,04
SRGAP1	ENST00000355086.3	hsa-miR-182-3p	-0,04	-0,06
PVRL1	ENST00000264025.3	hsa-miR-182-3p	-0,04	-0,11
RP11-156E8.1	ENST00000607453.1	hsa-miR-182-3p	-0,04	-0,04
ZNF208	ENST00000397126.4	hsa-miR-182-3p	-0,04	-0,04
GLRA3	ENST00000274093.3	hsa-miR-182-3p	-0,04	-0,04
SCN4B	ENST00000324727.4	hsa-miR-182-3p	-0,04	-0,04
TYW3	ENST00000457880.2	hsa-miR-182-3p	-0,04	-0,18
MMAA	ENST00000281317.5	hsa-miR-182-3p	-0,04	-0,17
INCENP	ENST00000394818.3	hsa-miR-182-3p	-0,04	-0,14
TTL	ENST00000233336.6	hsa-miR-182-3p	-0,04	-0,2
CXADR	ENST00000356275.6	hsa-miR-182-3p	-0,04	-0,2
NPR3	ENST00000265074.8	hsa-miR-182-3p	-0,04	-0,13
SLBP	ENST00000429429.2	hsa-miR-182-3p	-0,04	-0,22
WWC1	ENST00000265293.4	hsa-miR-182-3p	-0,04	-0,07
GRK1	ENST00000335678.6	hsa-miR-182-3p	-0,04	-0,04
GIT2	ENST00000355312.3	hsa-miR-182-3p	-0,04	-0,04
DPYSL5	ENST00000288699.6	hsa-miR-182-3p	-0,04	-0,06
RPL10	ENST00000424325.2	hsa-miR-182-3p	-0,04	-0,1
ANKRD33B	ENST00000296657.5	hsa-miR-182-3p	-0,04	-0,1
CBX5	ENST00000209875.4	hsa-miR-182-3p	-0,04	-0,13
NFYB	ENST00000240055.3	hsa-miR-182-3p	-0,04	-0,23
MKLN1	ENST00000352689.6	hsa-miR-182-3p	-0,04	-0,06
SRA1	ENST00000336283.6	hsa-miR-182-3p	-0,04	-0,52
TTYH2	ENST00000269346.4	hsa-miR-182-3p	-0,04	-0,05
ZNF623	ENST00000501748.2	hsa-miR-182-3p	-0,04	-0,05
PAPPA	ENST00000328252.3	hsa-miR-182-3p	-0,04	-0,05
MAPK13	ENST00000373766.5	hsa-miR-182-3p	-0,04	-0,04
SRSF6	ENST00000244020.3	hsa-miR-182-3p	-0,04	-0,14
GPR156	ENST00000464295.1	hsa-miR-182-3p	-0,04	-0,1
ZNF704	ENST00000327835.3	hsa-miR-182-3p	-0,04	-0,07
PCYOX1	ENST00000264441.5	hsa-miR-182-3p	-0,04	-0,25
MCAT	ENST00000327555.5	hsa-miR-182-3p	-0,04	-0,18
RHBDD1	ENST00000341329.3	hsa-miR-182-3p	-0,04	-0,13
DUSP4	ENST00000240100.2	hsa-miR-182-3p	-0,04	-0,04
GLIS3	ENST00000324333.10	hsa-miR-182-3p	-0,04	-0,04
PRR14L	ENST00000434485.1	hsa-miR-182-3p	-0,04	-0,04
FOXP4	ENST00000373063.3	hsa-miR-182-3p	-0,04	-0,04
TEC	ENST00000381501.3	hsa-miR-182-3p	-0,04	-0,09
UBE2R2	ENST00000263228.3	hsa-miR-182-3p	-0,04	-0,3
KIAA1324	ENST00000369939.3	hsa-miR-182-3p	-0,04	-0,07
RALGAPA2	ENST00000202677.7	hsa-miR-182-3p	-0,04	-0,05
MEGF8	ENST00000334370.4	hsa-miR-182-3p	-0,04	-0,04

AC132872.2	ENST00000598222.1	hsa-miR-182-3p	-0,04	-0,05
ZNF621	ENST00000339296.5	hsa-miR-182-3p	-0,04	-0,05
MRE11A	ENST00000323929.3	hsa-miR-182-3p	-0,04	-0,17
ARHGAP5	ENST00000345122.3	hsa-miR-182-3p	-0,04	-0,04
NFKBID	ENST00000396901.1	hsa-miR-182-3p	-0,04	-0,22
SH3BGRL2	ENST00000369838.4	hsa-miR-182-3p	-0,04	-0,11
ABLIM3	ENST00000326685.7	hsa-miR-182-3p	-0,04	-0,07
CCDC80	ENST00000206423.3	hsa-miR-182-3p	-0,04	-0,14
RASAL2	ENST00000448150.3	hsa-miR-182-3p	-0,04	-0,11
TLK1	ENST00000431350.2	hsa-miR-182-3p	-0,04	-0,15
C22orf29	ENST00000407472.1	hsa-miR-182-3p	-0,04	-0,06
RP11-10A14.4	ENST00000518496.1	hsa-miR-182-3p	-0,04	-0,1
STX6	ENST00000258301.5	hsa-miR-182-3p	-0,04	-0,08
AR	ENST00000374690.3	hsa-miR-182-3p	-0,04	-0,04
EPB41L5	ENST00000443902.2	hsa-miR-182-3p	-0,04	-0,04
SCRT1	ENST00000332135.4	hsa-miR-182-3p	-0,04	-0,04
PLXNA2	ENST00000367033.3	hsa-miR-182-3p	-0,04	-0,04
FOXO3	ENST00000343882.6	hsa-miR-182-3p	-0,04	-0,04
RASA4	ENST00000262940.7	hsa-miR-182-3p	-0,04	-0,04
KIF11	ENST00000260731.3	hsa-miR-182-3p	-0,04	-0,14
RABEP1	ENST00000262477.6	hsa-miR-182-3p	-0,04	-0,09
SPRED1	ENST00000299084.4	hsa-miR-182-3p	-0,04	-0,16
ACHE	ENST00000302913.4	hsa-miR-182-3p	-0,04	-0,11
CLCN4	ENST00000380833.4	hsa-miR-182-3p	-0,04	-0,05
ZFHX3	ENST00000268489.5	hsa-miR-182-3p	-0,04	-0,05
FBLIM1	ENST00000375771.1	hsa-miR-182-3p	-0,04	-0,04
KCNC1	ENST00000379472.3	hsa-miR-182-3p	-0,04	-0,04
FTO	ENST00000471389.1	hsa-miR-182-3p	-0,04	-0,14
ARHGEF5	ENST00000056217.5	hsa-miR-182-3p	-0,04	-0,15
RNF112	ENST00000461366.1	hsa-miR-182-3p	-0,04	-0,04
NAA35	ENST00000361671.5	hsa-miR-182-3p	-0,04	-0,12
ESRRB	ENST00000261532.7	hsa-miR-182-3p	-0,04	-0,18
MMP15	ENST00000219271.3	hsa-miR-182-3p	-0,04	-0,04
NEGR1	ENST00000357731.5	hsa-miR-182-3p	-0,04	-0,23
HOXA13	ENST00000222753.4	hsa-miR-182-3p	-0,04	-0,14
PDK3	ENST00000441463.2	hsa-miR-182-3p	-0,04	-0,07
LRRC8C	ENST00000370454.4	hsa-miR-182-3p	-0,04	-0,15
DIAPH2	ENST00000324765.8	hsa-miR-182-3p	-0,04	-0,04
MED21	ENST00000282892.3	hsa-miR-182-3p	-0,04	-0,16
RGS4	ENST00000531057.1	hsa-miR-182-3p	-0,04	-0,32
TOR1A	ENST00000351698.4	hsa-miR-182-3p	-0,04	-0,44
IPO5	ENST00000261574.5	hsa-miR-182-3p	-0,04	-0,13
RAB19	ENST00000275874.5	hsa-miR-182-3p	-0,04	-0,17
UHRF1BP1L	ENST00000279907.7	hsa-miR-182-3p	-0,04	-0,07
ZSCAN23	ENST00000289788.4	hsa-miR-182-3p	-0,04	-0,04
HIPK2	ENST00000406875.3	hsa-miR-182-3p	-0,04	-0,04
COL9A1	ENST00000357250.6	hsa-miR-182-3p	-0,04	-0,07
SUMF2	ENST00000434526.2	hsa-miR-182-3p	-0,04	-0,04
SEMA3E	ENST00000307792.3	hsa-miR-182-3p	-0,04	-0,18
PRSS21	ENST00000455114.1	hsa-miR-182-3p	-0,04	-0,18
VSTM4	ENST00000332853.4	hsa-miR-182-3p	-0,04	-0,14
CCDC142	ENST00000393965.3	hsa-miR-182-3p	-0,04	-0,1
RWDD1	ENST00000466444.2	hsa-miR-182-3p	-0,04	-0,19
ITGA10	ENST00000369304.3	hsa-miR-182-3p	-0,04	-0,04
NKD1	ENST00000268459.3	hsa-miR-182-3p	-0,04	-0,08
RETSAT	ENST00000295802.4	hsa-miR-182-3p	-0,04	-0,04
CLCN6	ENST00000312413.6	hsa-miR-182-3p	-0,04	-0,04
FAM135B	ENST00000395297.1	hsa-miR-182-3p	-0,04	-0,04
SAR1A	ENST00000373242.2	hsa-miR-182-3p	-0,04	-0,39
AGO3	ENST00000373191.4	hsa-miR-182-3p	-0,04	-0,1
PTPRF	ENST00000372414.3	hsa-miR-182-3p	-0,04	-0,04
HHIP	ENST00000296575.3	hsa-miR-182-3p	-0,04	-0,1
FRZB	ENST00000295113.4	hsa-miR-182-3p	-0,04	-0,35
RASAL1	ENST00000546530.1	hsa-miR-182-3p	-0,04	-0,09
DYNLL2	ENST00000579991.2	hsa-miR-182-3p	-0,04	-0,2
ROBO4	ENST00000306534.3	hsa-miR-182-3p	-0,04	-0,04

AGO4	ENST00000373210.3	hsa-miR-182-3p	-0,04	-0,04
TBR1	ENST00000389554.3	hsa-miR-182-3p	-0,04	-0,04
CNTN3	ENST00000263665.6	hsa-miR-182-3p	-0,04	-0,21
ACSS1	ENST00000323482.4	hsa-miR-182-3p	-0,04	-0,04
ADCY9	ENST00000294016.3	hsa-miR-182-3p	-0,04	-0,05
SDC2	ENST00000302190.4	hsa-miR-182-3p	-0,04	-0,17
ADNP	ENST00000371602.4	hsa-miR-182-3p	-0,04	-0,14
GUCY1A2	ENST00000526355.2	hsa-miR-182-3p	-0,04	-0,18
ZNF488	ENST00000494156.1	hsa-miR-182-3p	-0,04	-0,04
ATP8A2	ENST00000381655.2	hsa-miR-182-3p	-0,04	-0,07
ATXN7	ENST00000295900.6	hsa-miR-182-3p	-0,04	-0,04
CEP170	ENST00000366542.1	hsa-miR-182-3p	-0,04	-0,04
IL20RB	ENST00000309741.5	hsa-miR-182-3p	-0,04	-0,04
KIAA1549L	ENST00000321505.4	hsa-miR-182-3p	-0,04	-0,04
PRKAR1B	ENST00000537384.1	hsa-miR-182-3p	-0,03	-0,04
PDE12	ENST00000311180.8	hsa-miR-182-3p	-0,03	-0,06
GXYLT2	ENST00000389617.4	hsa-miR-182-3p	-0,03	-0,11
RRAGD	ENST00000369415.4	hsa-miR-182-3p	-0,03	-0,41
MFSD1	ENST00000415822.2	hsa-miR-182-3p	-0,03	-0,25
ARSE	ENST00000540563.1	hsa-miR-182-3p	-0,03	-0,14
ARMC8	ENST00000469044.1	hsa-miR-182-3p	-0,03	-0,22
CCDC50	ENST00000392455.3	hsa-miR-182-3p	-0,03	-0,2
NCAPG	ENST00000251496.2	hsa-miR-182-3p	-0,03	-0,27
RICTOR	ENST00000357387.3	hsa-miR-182-3p	-0,03	-0,04
HCN1	ENST00000303230.4	hsa-miR-182-3p	-0,03	-0,16
EFCAB14	ENST00000371933.3	hsa-miR-182-3p	-0,03	-0,15
TBC1D16	ENST00000310924.2	hsa-miR-182-3p	-0,03	-0,04
AC005606.1	ENST00000598236.1	hsa-miR-182-3p	-0,03	-0,18
FRK	ENST00000606080.1	hsa-miR-182-3p	-0,03	-0,07
SORCS2	ENST00000507866.2	hsa-miR-182-3p	-0,03	-0,03
MEAF6	ENST00000373075.2	hsa-miR-182-3p	-0,03	-0,13
RCC2	ENST00000375436.4	hsa-miR-182-3p	-0,03	-0,03
DISC1	ENST00000439617.2	hsa-miR-182-3p	-0,03	-0,03
TMEM184A	ENST00000297477.5	hsa-miR-182-3p	-0,03	-0,04
ABT1	ENST00000274849.1	hsa-miR-182-3p	-0,03	-0,14
RPL12	ENST00000361436.5	hsa-miR-182-3p	-0,03	-0,06
FBXO31	ENST00000311635.7	hsa-miR-182-3p	-0,03	-0,09
HEATR6	ENST00000184956.6	hsa-miR-182-3p	-0,03	-0,15
AC007040.11	ENST00000606025.1	hsa-miR-182-3p	-0,03	-0,08
ANAPC16	ENST00000299381.4	hsa-miR-182-3p	-0,03	-0,17
KIAA0930	ENST00000336156.5	hsa-miR-182-3p	-0,03	-0,14
NFATC3	ENST00000329524.4	hsa-miR-182-3p	-0,03	-0,03
SMUG1	ENST00000505128.1	hsa-miR-182-3p	-0,03	-0,44
DPP10	ENST00000410059.1	hsa-miR-182-3p	-0,03	-0,03
TMEM178B	ENST00000565468.1	hsa-miR-182-3p	-0,03	-0,03
IQSEC3	ENST00000538872.1	hsa-miR-182-3p	-0,03	-0,03
SPTLC3	ENST00000399002.2	hsa-miR-182-3p	-0,03	-0,14
FLJ27365	ENST00000360737.3	hsa-miR-182-3p	-0,03	-0,03
ACVRL1	ENST00000550683.1	hsa-miR-182-3p	-0,03	-0,06
PTPLAD2	ENST00000495827.2	hsa-miR-182-3p	-0,03	-0,12
SCUBE3	ENST00000274938.7	hsa-miR-182-3p	-0,03	-0,06
INPP5K	ENST00000421807.2	hsa-miR-182-3p	-0,03	-0,15
DMGDH	ENST00000380311.4	hsa-miR-182-3p	-0,03	-0,1
GBA	ENST00000368373.3	hsa-miR-182-3p	-0,03	-0,04
PTBP2	ENST00000609116.1	hsa-miR-182-3p	-0,03	-0,07
ARHGAP31	ENST00000264245.4	hsa-miR-182-3p	-0,03	-0,1
NETO2	ENST00000562435.1	hsa-miR-182-3p	-0,03	-0,67
SAP30BP	ENST00000584667.1	hsa-miR-182-3p	-0,03	-0,15
MAT1A	ENST00000372213.3	hsa-miR-182-3p	-0,03	-0,16
ZNRF1	ENST00000335325.4	hsa-miR-182-3p	-0,03	-0,05
ALDH5A1	ENST00000357578.3	hsa-miR-182-3p	-0,03	-0,06
CSNK1G1	ENST00000303052.7	hsa-miR-182-3p	-0,03	-0,03
BORA	ENST00000390667.5	hsa-miR-182-3p	-0,03	-0,11
PIFO	ENST00000369738.4	hsa-miR-182-3p	-0,03	-0,14
BLOC1S4	ENST00000320776.3	hsa-miR-182-3p	-0,03	-0,27
KDM5A	ENST00000399788.2	hsa-miR-182-3p	-0,03	-0,04

PPARGC1B	ENST00000309241.5	hsa-miR-182-3p	-0,03	-0,04
TUBB	ENST00000327892.8	hsa-miR-182-3p	-0,03	-0,33
LOH12CR1	ENST00000314565.4	hsa-miR-182-3p	-0,03	-0,07
DRP2	ENST00000402866.1	hsa-miR-182-3p	-0,03	-0,03
PRICKLE2	ENST00000295902.6	hsa-miR-182-3p	-0,03	-0,03
SHISA6	ENST00000441885.3	hsa-miR-182-3p	-0,03	-0,03
C1orf95	ENST00000366788.3	hsa-miR-182-3p	-0,03	-0,14
ZC3HAV1	ENST00000242351.5	hsa-miR-182-3p	-0,03	-0,03
RAD9B	ENST00000409425.1	hsa-miR-182-3p	-0,03	-0,2
PPM1A	ENST00000395076.4	hsa-miR-182-3p	-0,03	-0,16
WDYHV1	ENST00000523984.1	hsa-miR-182-3p	-0,03	-0,21
SAMD9	ENST00000379958.2	hsa-miR-182-3p	-0,03	-0,06
ZC3H4	ENST00000253048.5	hsa-miR-182-3p	-0,03	-0,03
NCR3LG1	ENST00000338965.4	hsa-miR-182-3p	-0,03	-0,03
NUCB2	ENST00000529010.1	hsa-miR-182-3p	-0,03	-0,22
TRAF6	ENST00000526995.1	hsa-miR-182-3p	-0,03	-0,07
UBXN7	ENST00000296328.4	hsa-miR-182-3p	-0,03	-0,11
GGACT	ENST00000376250.2	hsa-miR-182-3p	-0,03	-0,11
MUC19	ENST00000454784.4	hsa-miR-182-3p	-0,03	-0,03
LPP	ENST00000312675.4	hsa-miR-182-3p	-0,03	-0,03
NHS	ENST00000380060.3	hsa-miR-182-3p	-0,03	-0,03
CRTC3	ENST00000420329.2	hsa-miR-182-3p	-0,03	-0,03
PTPRT	ENST00000373187.1	hsa-miR-182-3p	-0,03	-0,03
STARD9	ENST00000290607.7	hsa-miR-182-3p	-0,03	-0,03
XYLT1	ENST00000261381.6	hsa-miR-182-3p	-0,03	-0,03
TMEM200C	ENST00000581347.2	hsa-miR-182-3p	-0,03	-0,29
ZC3H12D	ENST00000416573.2	hsa-miR-182-3p	-0,03	-0,03
SLC24A2	ENST00000341998.2	hsa-miR-182-3p	-0,03	-0,03
PREX2	ENST00000288368.4	hsa-miR-182-3p	-0,03	-0,03
FAM58A	ENST00000406277.2	hsa-miR-182-3p	-0,03	-0,24
THUMPD3	ENST00000345094.3	hsa-miR-182-3p	-0,03	-0,16
MDGA1	ENST00000297153.7	hsa-miR-182-3p	-0,03	-0,03
HEG1	ENST00000311127.4	hsa-miR-182-3p	-0,03	-0,03
SPPL3	ENST00000353487.2	hsa-miR-182-3p	-0,03	-0,07
PPIL6	ENST00000521072.2	hsa-miR-182-3p	-0,03	-0,26
RPL27A	ENST00000314138.6	hsa-miR-182-3p	-0,03	-0,28
GPSM2	ENST00000406462.2	hsa-miR-182-3p	-0,03	-0,1
ZNF730	ENST00000593635.1	hsa-miR-182-3p	-0,03	-0,08
FOXI2	ENST00000388920.4	hsa-miR-182-3p	-0,03	-0,03
FAM53B	ENST00000337318.3	hsa-miR-182-3p	-0,03	-0,03
HECTD4	ENST00000377560.5	hsa-miR-182-3p	-0,03	-0,03
HHIPL1	ENST00000330710.5	hsa-miR-182-3p	-0,03	-0,03
STOX2	ENST00000308497.4	hsa-miR-182-3p	-0,03	-0,03
RPGRIP1L	ENST00000262135.4	hsa-miR-182-3p	-0,03	-0,07
HEBP2	ENST00000607197.1	hsa-miR-182-3p	-0,03	-0,14
RBP2	ENST00000232217.2	hsa-miR-182-3p	-0,03	-0,32
LRRC3	ENST00000291592.4	hsa-miR-182-3p	-0,03	-0,03
CENPL	ENST00000356198.2	hsa-miR-182-3p	-0,03	-0,2
COQ7	ENST00000321998.5	hsa-miR-182-3p	-0,03	-0,08
TSN	ENST00000536142.1	hsa-miR-182-3p	-0,03	-0,18
CNIH1	ENST00000395573.4	hsa-miR-182-3p	-0,03	-0,45
MTUS2	ENST00000431530.3	hsa-miR-182-3p	-0,03	-0,07
ERLIN2	ENST00000276461.5	hsa-miR-182-3p	-0,03	-0,03
AHCYL2	ENST00000325006.3	hsa-miR-182-3p	-0,03	-0,13
ZNF780B	ENST00000434248.1	hsa-miR-182-3p	-0,03	-0,15
PCDH12	ENST00000231484.3	hsa-miR-182-3p	-0,03	-0,03
VASH1	ENST00000167106.4	hsa-miR-182-3p	-0,03	-0,03
TPCN1	ENST00000335509.6	hsa-miR-182-3p	-0,03	-0,03
DNAJC6	ENST00000395325.3	hsa-miR-182-3p	-0,03	-0,03
ZDHHC3	ENST00000296127.3	hsa-miR-182-3p	-0,03	-0,12
LONRF3	ENST00000304778.7	hsa-miR-182-3p	-0,03	-0,07
LSAMP	ENST00000490035.2	hsa-miR-182-3p	-0,03	-0,11
CTNNBIP1	ENST00000377263.1	hsa-miR-182-3p	-0,03	-0,15
TBC1D12	ENST00000225235.4	hsa-miR-182-3p	-0,03	-0,04
NFIC	ENST00000346156.5	hsa-miR-182-3p	-0,03	-0,03
SPATA18	ENST00000295213.4	hsa-miR-182-3p	-0,03	-0,11

HSP90B1	ENST00000299767.5	hsa-miR-182-3p	-0,03	-0,23
LSG1	ENST00000265245.5	hsa-miR-182-3p	-0,03	-0,04
SNPH	ENST00000381873.3	hsa-miR-182-3p	-0,03	-0,03
CNOT6L	ENST00000504123.1	hsa-miR-182-3p	-0,03	-0,12
MLXIP	ENST00000319080.7	hsa-miR-182-3p	-0,03	-0,03
LRRC28	ENST00000301981.3	hsa-miR-182-3p	-0,03	-0,12
CELF1	ENST00000395290.2	hsa-miR-182-3p	-0,03	-0,03
LCORL	ENST00000326877.4	hsa-miR-182-3p	-0,03	-0,24
CABLES2	ENST00000279101.5	hsa-miR-182-3p	-0,03	-0,08
NR3C1	ENST00000394464.2	hsa-miR-182-3p	-0,03	-0,09
NDUFAF5	ENST00000378106.5	hsa-miR-182-3p	-0,03	-0,08
NUDT16	ENST00000359850.3	hsa-miR-182-3p	-0,03	-0,08
GPATCH2	ENST00000366935.3	hsa-miR-182-3p	-0,03	-0,15
GIPR	ENST00000590918.1	hsa-miR-182-3p	-0,03	-0,03
INPP5F	ENST00000361976.2	hsa-miR-182-3p	-0,03	-0,03
GATAD2B	ENST00000368655.4	hsa-miR-182-3p	-0,03	-0,11
SPTLC2	ENST00000216484.2	hsa-miR-182-3p	-0,03	-0,08
KHNYN	ENST00000251343.5	hsa-miR-182-3p	-0,03	-0,16
SLC31A1	ENST00000374212.4	hsa-miR-182-3p	-0,03	-0,24
LAMTOR3	ENST00000499666.2	hsa-miR-182-3p	-0,03	-0,23
TMEM30A	ENST00000230461.6	hsa-miR-182-3p	-0,03	-0,38
SLC40A1	ENST00000261024.2	hsa-miR-182-3p	-0,03	-0,13
PIP4K2B	ENST00000269554.3	hsa-miR-182-3p	-0,03	-0,03
SMCR8	ENST00000406438.3	hsa-miR-182-3p	-0,03	-0,03
GLRX3	ENST00000368644.1	hsa-miR-182-3p	-0,03	-0,06
SAPCD2	ENST00000409687.3	hsa-miR-182-3p	-0,03	-0,03
LCA5	ENST00000369846.4	hsa-miR-182-3p	-0,03	-0,03
MAP3K2	ENST00000409947.1	hsa-miR-182-3p	-0,03	-0,03
POLR1A	ENST00000263857.6	hsa-miR-182-3p	-0,03	-0,03
ZNF573	ENST00000590414.2	hsa-miR-182-3p	-0,03	-0,05
SCN8A	ENST00000354534.6	hsa-miR-182-3p	-0,03	-0,03
FAM208B	ENST00000328090.5	hsa-miR-182-3p	-0,02	-0,1
TTF2	ENST00000369466.4	hsa-miR-182-3p	-0,02	-0,06
LBX1	ENST00000370193.2	hsa-miR-182-3p	-0,02	-0,3
TCEANC2	ENST00000234827.1	hsa-miR-182-3p	-0,02	-0,13
PAIP2B	ENST00000244221.8	hsa-miR-182-3p	-0,02	-0,12
TXNDC17	ENST00000250101.5	hsa-miR-182-3p	-0,02	-0,34
ARHGAP26	ENST00000378004.3	hsa-miR-182-3p	-0,02	-0,04
ADD2	ENST00000264436.4	hsa-miR-182-3p	-0,02	-0,05
ELP5	ENST00000574993.1	hsa-miR-182-3p	-0,02	-0,11
METTL14	ENST00000388822.5	hsa-miR-182-3p	-0,02	-0,18
PSMG1	ENST00000331573.3	hsa-miR-182-3p	-0,02	-0,15
TENM1	ENST00000371130.3	hsa-miR-182-3p	-0,02	-0,02
ANKRD9	ENST00000559651.1	hsa-miR-182-3p	-0,02	-0,17
MYO18B	ENST00000335473.7	hsa-miR-182-3p	-0,02	-0,2
PHF14	ENST00000403050.3	hsa-miR-182-3p	-0,02	-0,12
TBL3	ENST00000568546.1	hsa-miR-182-3p	-0,02	-0,07
LACE1	ENST00000368977.4	hsa-miR-182-3p	-0,02	-0,07
ATG14	ENST00000247178.5	hsa-miR-182-3p	-0,02	-0,12
SLC25A36	ENST00000446041.2	hsa-miR-182-3p	-0,02	-0,07
DIP2C	ENST00000280886.6	hsa-miR-182-3p	-0,02	-0,03
CLMN	ENST00000298912.4	hsa-miR-182-3p	-0,02	-0,1
NOL10	ENST00000345985.3	hsa-miR-182-3p	-0,02	-0,07
SPTSSA	ENST00000298130.4	hsa-miR-182-3p	-0,02	-0,13
CBX1	ENST00000393408.3	hsa-miR-182-3p	-0,02	-0,12
UQCC1	ENST00000349714.5	hsa-miR-182-3p	-0,02	-0,13
CNNM2	ENST00000369878.4	hsa-miR-182-3p	-0,02	-0,06
GLDN	ENST00000335449.6	hsa-miR-182-3p	-0,02	-0,02
ATP10A	ENST00000356865.6	hsa-miR-182-3p	-0,02	-0,07
CREBRF	ENST00000540014.1	hsa-miR-182-3p	-0,02	-0,09
LPPR5	ENST00000370188.3	hsa-miR-182-3p	-0,02	-0,11
EIF2AK2	ENST00000233057.4	hsa-miR-182-3p	-0,02	-0,04
AMDHD2	ENST00000302956.4	hsa-miR-182-3p	-0,02	-0,06
HSD17B12	ENST00000278353.4	hsa-miR-182-3p	-0,02	-0,19
P2RY6	ENST00000393590.2	hsa-miR-182-3p	-0,02	-0,13
MRPS5	ENST00000272418.2	hsa-miR-182-3p	-0,02	-0,17

ITPRIP	ENST00000337478.1	hsa-miR-182-3p	-0,02	-0,03
SLC7A14	ENST00000231706.5	hsa-miR-182-3p	-0,02	-0,02
GLYCTK	ENST00000354773.4	hsa-miR-182-3p	-0,02	-0,02
FBXO48	ENST00000377957.3	hsa-miR-182-3p	-0,02	-0,06
GALNT6	ENST00000543196.2	hsa-miR-182-3p	-0,02	-0,13
ETNK1	ENST00000266517.4	hsa-miR-182-3p	-0,02	-0,11
RAD1	ENST00000382038.2	hsa-miR-182-3p	-0,02	-0,11
PNPLA4	ENST00000381042.4	hsa-miR-182-3p	-0,02	-0,2
PPIE	ENST00000324379.5	hsa-miR-182-3p	-0,02	-0,6
XPNPEP3	ENST00000357137.4	hsa-miR-182-3p	-0,02	-0,22
MYPN	ENST00000358913.5	hsa-miR-182-3p	-0,02	-0,09
FAM177A1	ENST00000280987.4	hsa-miR-182-3p	-0,02	-0,21
NF2	ENST00000347330.5	hsa-miR-182-3p	-0,02	-0,03
TTC39A	ENST00000530004.1	hsa-miR-182-3p	-0,02	-0,27
CYLD	ENST00000540145.1	hsa-miR-182-3p	-0,02	-0,09
TRIM24	ENST00000343526.4	hsa-miR-182-3p	-0,02	-0,11
VPS53	ENST00000437048.2	hsa-miR-182-3p	-0,02	-0,08
MAP9	ENST00000311277.4	hsa-miR-182-3p	-0,02	-0,09
SPEG	ENST00000312358.7	hsa-miR-182-3p	-0,02	-0,02
KIF6	ENST00000287152.7	hsa-miR-182-3p	-0,02	-0,05
SEC22A	ENST00000481965.2	hsa-miR-182-3p	-0,02	-0,17
FMNL3	ENST00000335154.5	hsa-miR-182-3p	-0,02	-0,08
SYNRG	ENST00000339208.6	hsa-miR-182-3p	-0,02	-0,04
SYT7	ENST00000263846.4	hsa-miR-182-3p	-0,02	-0,15
FAM134A	ENST00000430297.2	hsa-miR-182-3p	-0,02	-0,36
ZNF778	ENST00000433976.2	hsa-miR-182-3p	-0,02	-0,04
C1GALT1	ENST00000223122.3	hsa-miR-182-3p	-0,02	-0,09
C5orf63	ENST00000535381.1	hsa-miR-182-3p	-0,02	-0,18
TEAD1	ENST00000361905.4	hsa-miR-182-3p	-0,02	-0,02
LRIG2	ENST00000361127.5	hsa-miR-182-3p	-0,02	-0,02
DDX3X	ENST00000399959.2	hsa-miR-182-3p	-0,02	-0,08
C15orf38	ENST00000357484.5	hsa-miR-182-3p	-0,02	-0,16
MRRF	ENST00000344641.3	hsa-miR-182-3p	-0,02	-0,13
EPC1	ENST00000319778.6	hsa-miR-182-3p	-0,02	-0,13
AQP2	ENST00000199280.3	hsa-miR-182-3p	-0,02	-0,02
CACNA2D4	ENST00000382722.5	hsa-miR-182-3p	-0,02	-0,02
ETV3	ENST00000368192.4	hsa-miR-182-3p	-0,02	-0,02
GPR180	ENST00000376958.4	hsa-miR-182-3p	-0,02	-0,08
NOX5	ENST00000260364.5	hsa-miR-182-3p	-0,02	-0,02
WNT4	ENST00000290167.6	hsa-miR-182-3p	-0,02	-0,02
SDK2	ENST00000392650.3	hsa-miR-182-3p	-0,02	-0,02
KIAA2018	ENST00000316407.4	hsa-miR-182-3p	-0,02	-0,02
ABCA1	ENST00000374736.3	hsa-miR-182-3p	-0,02	-0,02
BEND3	ENST00000369042.1	hsa-miR-182-3p	-0,02	-0,02
KCNAB2	ENST00000378097.1	hsa-miR-182-3p	-0,02	-0,02
NFASC	ENST00000401399.1	hsa-miR-182-3p	-0,02	-0,02
SAMD12	ENST00000409003.4	hsa-miR-182-3p	-0,02	-0,02
ZNF862	ENST00000223210.4	hsa-miR-182-3p	-0,02	-0,02
ATP1B4	ENST00000218008.3	hsa-miR-182-3p	-0,02	-0,02
TECPR1	ENST00000447648.2	hsa-miR-182-3p	-0,02	-0,02
LZTS3	ENST00000329152.3	hsa-miR-182-3p	-0,02	-0,02
PDE11A	ENST00000286063.6	hsa-miR-182-3p	-0,02	-0,02
CARD14	ENST00000573882.1	hsa-miR-182-3p	-0,02	-0,02
FAT3	ENST00000298047.6	hsa-miR-182-3p	-0,02	-0,02
APLF	ENST00000303795.4	hsa-miR-182-3p	-0,02	-0,09
AFF1	ENST00000395146.4	hsa-miR-182-3p	-0,02	-0,05
PYGO2	ENST00000368457.2	hsa-miR-182-3p	-0,02	-0,08
GSE1	ENST00000253458.7	hsa-miR-182-3p	-0,02	-0,02
KMT2A	ENST00000534358.1	hsa-miR-182-3p	-0,02	-0,02
C17orf51	ENST00000391411.5	hsa-miR-182-3p	-0,02	-0,05
ST6GALNAC5	ENST00000477717.1	hsa-miR-182-3p	-0,02	-0,06
CYP1B1	ENST00000260630.3	hsa-miR-182-3p	-0,02	-0,13
OSBPL8	ENST00000393249.2	hsa-miR-182-3p	-0,02	-0,1
BID	ENST00000317361.7	hsa-miR-182-3p	-0,02	-0,19
FBXO41	ENST00000295133.5	hsa-miR-182-3p	-0,02	-0,02
PIK3CA	ENST00000263967.3	hsa-miR-182-3p	-0,02	-0,3

DHX33	ENST00000225296.3	hsa-miR-182-3p	-0,02	-0,02
SPA17	ENST00000532692.1	hsa-miR-182-3p	-0,02	-0,16
RAB14	ENST00000373840.4	hsa-miR-182-3p	-0,02	-0,05
CCL1	ENST00000225842.3	hsa-miR-182-3p	-0,02	-0,15
RPH3A	ENST00000415485.3	hsa-miR-182-3p	-0,02	-0,02
FAM204A	ENST00000369183.4	hsa-miR-182-3p	-0,02	-0,25
CPLX1	ENST00000304062.6	hsa-miR-182-3p	-0,02	-0,11
ZBTB17	ENST00000375743.4	hsa-miR-182-3p	-0,02	-0,14
ANKS6	ENST00000375018.1	hsa-miR-182-3p	-0,02	-0,03
KCTD16	ENST00000507359.3	hsa-miR-182-3p	-0,02	-0,1
UVRAG	ENST00000356136.3	hsa-miR-182-3p	-0,02	-0,04
ACOX1	ENST00000293217.5	hsa-miR-182-3p	-0,02	-0,02
LHX4	ENST00000263726.2	hsa-miR-182-3p	-0,02	-0,05
SGPL1	ENST00000373202.3	hsa-miR-182-3p	-0,02	-0,07
SFT2D3	ENST00000310981.4	hsa-miR-182-3p	-0,02	-0,18
SMURF1	ENST00000361368.2	hsa-miR-182-3p	-0,02	-0,07
SLC25A21	ENST00000331299.5	hsa-miR-182-3p	-0,02	-0,19
KLHL38	ENST00000325995.7	hsa-miR-182-3p	-0,02	-0,06
ULK1	ENST00000321867.4	hsa-miR-182-3p	-0,02	-0,02
NOVA2	ENST00000263257.5	hsa-miR-182-3p	-0,02	-0,03
WDR70	ENST00000265107.4	hsa-miR-182-3p	-0,02	-0,03
SEMA3A	ENST00000265362.4	hsa-miR-182-3p	-0,02	-0,13
EPHA6	ENST00000470610.2	hsa-miR-182-3p	-0,02	-0,23
GTPBP10	ENST00000222511.6	hsa-miR-182-3p	-0,02	-0,15
SPRED3	ENST00000587013.1	hsa-miR-182-3p	-0,02	-0,02
SDK1	ENST00000404826.2	hsa-miR-182-3p	-0,02	-0,02
ZNF394	ENST00000426306.2	hsa-miR-182-3p	-0,02	-0,11
RANBP3	ENST00000439268.2	hsa-miR-182-3p	-0,02	-0,06
AKAP8	ENST00000269701.2	hsa-miR-182-3p	-0,02	-0,18
ZDHHC21	ENST00000380916.4	hsa-miR-182-3p	-0,02	-0,17
CNKSR3	ENST00000607772.1	hsa-miR-182-3p	-0,02	-0,35
SPATS2	ENST00000553127.1	hsa-miR-182-3p	-0,02	-0,12
SFXN5	ENST00000410065.1	hsa-miR-182-3p	-0,02	-0,04
EGFR	ENST00000275493.2	hsa-miR-182-3p	-0,02	-0,02
TEP1	ENST00000262715.5	hsa-miR-182-3p	-0,02	-0,05
PLXDC1	ENST00000315392.4	hsa-miR-182-3p	-0,02	-0,03
ZNF652	ENST00000362063.2	hsa-miR-182-3p	-0,02	-0,11
ADAMTS9	ENST00000295903.4	hsa-miR-182-3p	-0,02	-0,02
DNAJB13	ENST00000339764.1	hsa-miR-182-3p	-0,02	-0,07
FRMD5	ENST00000484674.1	hsa-miR-182-3p	-0,02	-0,04
PRDM6	ENST00000407847.4	hsa-miR-182-3p	-0,02	-0,02
PTRH1	ENST00000423807.1	hsa-miR-182-3p	-0,02	-0,22
MYO6	ENST00000369981.3	hsa-miR-182-3p	-0,02	-0,13
CARHSP1	ENST00000396593.2	hsa-miR-182-3p	-0,02	-0,08
PPM1H	ENST00000228705.6	hsa-miR-182-3p	-0,02	-0,09
PTBP3	ENST00000374257.1	hsa-miR-182-3p	-0,02	-0,05
ENY2	ENST00000520147.1	hsa-miR-182-3p	-0,02	-0,1
G3BP1	ENST00000394123.3	hsa-miR-182-3p	-0,02	-0,07
SVIP	ENST00000354193.4	hsa-miR-182-3p	-0,02	-0,36
IVNS1ABP	ENST00000367498.3	hsa-miR-182-3p	-0,02	-0,15
INSIG2	ENST00000245787.4	hsa-miR-182-3p	-0,02	-0,28
CERS5	ENST00000317551.6	hsa-miR-182-3p	-0,02	-0,18
MRPS30	ENST00000507110.1	hsa-miR-182-3p	-0,02	-0,34
TMEM110	ENST00000355083.5	hsa-miR-182-3p	-0,02	-0,03
GLIS2	ENST00000262366.3	hsa-miR-182-3p	-0,02	-0,02
MRC2	ENST00000303375.5	hsa-miR-182-3p	-0,02	-0,02
ABCF2	ENST00000287844.2	hsa-miR-182-3p	-0,02	-0,2
ZNF326	ENST00000370447.3	hsa-miR-182-3p	-0,02	-0,12
HIP1	ENST00000336926.6	hsa-miR-182-3p	-0,02	-0,02
DRG1	ENST00000331457.4	hsa-miR-182-3p	-0,02	-0,15
UBA52	ENST00000442744.2	hsa-miR-182-3p	-0,02	-0,19
RYK	ENST00000296084.4	hsa-miR-182-3p	-0,02	-0,07
NT5C3B	ENST00000269534.8	hsa-miR-182-3p	-0,02	-0,29
CSPP1	ENST00000262210.5	hsa-miR-182-3p	-0,02	-0,08
ITGA1	ENST00000282588.6	hsa-miR-182-3p	-0,02	-0,04
CCDC93	ENST00000376300.2	hsa-miR-182-3p	-0,02	-0,24

FAM83F	ENST00000333407.6	hsa-miR-182-3p	-0,01	-0,01
UGT3A1	ENST00000274278.3	hsa-miR-182-3p	-0,01	-0,1
SIKE1	ENST00000369528.5	hsa-miR-182-3p	-0,01	-0,17
MRPS14	ENST00000476371.1	hsa-miR-182-3p	-0,01	-0,39
CACNB4	ENST00000539935.1	hsa-miR-182-3p	-0,01	-0,07
YIPF4	ENST00000238831.4	hsa-miR-182-3p	-0,01	-0,03
RBM28	ENST00000223073.2	hsa-miR-182-3p	-0,01	-0,05
STOML1	ENST00000564777.1	hsa-miR-182-3p	-0,01	-0,02
CLDN19	ENST00000296387.1	hsa-miR-182-3p	-0,01	-0,11
MR1	ENST00000367580.5	hsa-miR-182-3p	-0,01	-0,08
ZNF274	ENST00000326804.4	hsa-miR-182-3p	-0,01	-0,15
SLC2A11	ENST00000345044.6	hsa-miR-182-3p	-0,01	-0,14
SHROOM3	ENST00000296043.6	hsa-miR-182-3p	-0,01	-0,03
RAB11A	ENST00000569896.1	hsa-miR-182-3p	-0,01	-0,23
NAA60	ENST00000610180.1	hsa-miR-182-3p	-0,01	-0,01
TCF21	ENST00000367882.4	hsa-miR-182-3p	-0,01	-0,13
PHC3	ENST00000495893.2	hsa-miR-182-3p	-0,01	-0,05
ARL2BP	ENST00000219204.3	hsa-miR-182-3p	-0,01	-0,2
ATRX	ENST00000373344.5	hsa-miR-182-3p	-0,01	-0,02
ATP6V0A2	ENST00000330342.3	hsa-miR-182-3p	-0,01	-0,13
KIAA0408	ENST00000483725.3	hsa-miR-182-3p	-0,01	-0,04
AFF2	ENST00000370460.2	hsa-miR-182-3p	-0,01	-0,02
CBLB	ENST00000264122.4	hsa-miR-182-3p	-0,01	-0,07
RAP1GAP2	ENST00000254695.8	hsa-miR-182-3p	-0,01	-0,02
SMAD2	ENST00000262160.6	hsa-miR-182-3p	-0,01	-0,15
CCDC127	ENST00000296824.3	hsa-miR-182-3p	-0,01	-0,09
ACPL2	ENST00000286353.4	hsa-miR-182-3p	-0,01	-0,21
FGF12	ENST00000445105.2	hsa-miR-182-3p	-0,01	-0,13
TTC26	ENST00000430935.1	hsa-miR-182-3p	-0,01	-0,02
FMN1	ENST00000334528.9	hsa-miR-182-3p	-0,01	-0,07
UMPS	ENST00000232607.2	hsa-miR-182-3p	-0,01	-0,08
LPIN2	ENST00000261596.4	hsa-miR-182-3p	-0,01	-0,01
SNX27	ENST00000368843.3	hsa-miR-182-3p	-0,01	-0,01
AMD1	ENST00000368885.3	hsa-miR-182-3p	-0,01	-0,07
NCAPH	ENST00000240423.4	hsa-miR-182-3p	-0,01	-0,3
FOSL2	ENST00000379619.1	hsa-miR-182-3p	-0,01	-0,16
KLHDC7B	ENST00000395676.2	hsa-miR-182-3p	-0,01	-0,02
SIRT5	ENST00000606117.1	hsa-miR-182-3p	-0,01	-0,03
MSH6	ENST00000234420.5	hsa-miR-182-3p	-0,01	-0,17
CHRM3	ENST00000255380.4	hsa-miR-182-3p	-0,01	-0,06
DONSON	ENST00000453626.1	hsa-miR-182-3p	-0,01	-0,19
PDIA3	ENST00000300289.5	hsa-miR-182-3p	-0,01	-0,4
PGR	ENST00000325455.5	hsa-miR-182-3p	-0,01	-0,02
UBE2F	ENST00000272930.4	hsa-miR-182-3p	-0,01	-0,22
TSPAN2	ENST00000369516.2	hsa-miR-182-3p	-0,01	-0,07
NMT1	ENST00000592782.1	hsa-miR-182-3p	-0,01	-0,01
RAB23	ENST00000317483.3	hsa-miR-182-3p	-0,01	-0,16
GNL1	ENST00000376621.3	hsa-miR-182-3p	-0,01	-0,07
RNF115	ENST00000369291.5	hsa-miR-182-3p	-0,01	-0,1
MCMBP	ENST00000360003.3	hsa-miR-182-3p	-0,01	-0,16
IRF2BP2	ENST00000366610.3	hsa-miR-182-3p	-0,01	-0,12
TMEM53	ENST00000372237.3	hsa-miR-182-3p	-0,01	-0,14
RAB6B	ENST00000285208.4	hsa-miR-182-3p	-0,01	-0,16
KLF11	ENST00000305883.1	hsa-miR-182-3p	-0,01	-0,07
BPTF	ENST00000321892.4	hsa-miR-182-3p	-0,01	-0,05
IKZF1	ENST00000331340.3	hsa-miR-182-3p	-0,01	-0,01
TFAP2B	ENST00000393655.3	hsa-miR-182-3p	-0,01	-0,06
DUSP11	ENST00000272444.3	hsa-miR-182-3p	-0,01	-0,3
THAP2	ENST00000308086.2	hsa-miR-182-3p	-0,01	-0,06
OLFM2	ENST00000264833.4	hsa-miR-182-3p	-0,01	-0,09
PAPLN	ENST00000381166.3	hsa-miR-182-3p	-0,01	-0,04
STAMBP	ENST00000394070.2	hsa-miR-182-3p	-0,01	-0,11
IRAK3	ENST00000261233.4	hsa-miR-182-3p	-0,01	-0,03
NIPA2	ENST00000337451.3	hsa-miR-182-3p	-0,01	-0,2
JAG2	ENST00000331782.3	hsa-miR-182-3p	-0,01	-0,16
PSMB9	ENST00000395330.1	hsa-miR-182-3p	-0,01	-0,29

SCN9A	ENST00000409672.1	hsa-miR-182-3p	-0,01	-0,13
C6orf223	ENST00000439969.2	hsa-miR-182-3p	-0,01	-0,03
CLVS2	ENST00000275162.5	hsa-miR-182-3p	-0,01	-0,03
MMADHC	ENST00000303319.5	hsa-miR-182-3p	-0,01	-0,13
TMOD2	ENST00000249700.4	hsa-miR-182-3p	-0,01	-0,01
ADAMTS2	ENST00000251582.7	hsa-miR-182-3p	-0,01	-0,01
FBXO25	ENST00000276326.5	hsa-miR-182-3p	-0,01	-0,18
SOX6	ENST00000316399.6	hsa-miR-182-3p	-0,01	-0,03
CRKL	ENST00000354336.3	hsa-miR-182-3p	-0,01	-0,01
SRD5A3	ENST00000264228.4	hsa-miR-182-3p	-0,01	-0,19
CTSB	ENST00000353047.6	hsa-miR-182-3p	-0,01	-0,16
SLC14A1	ENST00000321925.4	hsa-miR-182-3p	-0,01	-0,09
ZMIZ2	ENST00000309315.4	hsa-miR-182-3p	-0,01	-0,03
GABRQ	ENST00000370306.2	hsa-miR-182-3p	-0,01	-0,03
SH3BP2	ENST00000356331.5	hsa-miR-182-3p	-0,01	-0,04
PSMC4	ENST00000157812.2	hsa-miR-182-3p	-0,01	-0,2
COX5A	ENST00000322347.6	hsa-miR-182-3p	-0,01	-0,39
MESDC2	ENST00000261758.4	hsa-miR-182-3p	-0,01	-0,11
EIF4EBP3	ENST00000310331.2	hsa-miR-182-3p	-0,01	-0,25
ABCG8	ENST00000272286.2	hsa-miR-182-3p	-0,01	-0,09
STRBP	ENST00000447404.2	hsa-miR-182-3p	-0,01	-0,1
FAM26E	ENST00000368599.3	hsa-miR-182-3p	-0,01	-0,07
IER3IP1	ENST00000256433.3	hsa-miR-182-3p	-0,01	-0,1
TGFBR1	ENST00000374994.4	hsa-miR-182-3p	-0,01	-0,11
IFNAR2	ENST00000404220.3	hsa-miR-182-3p	-0,01	-0,02
CACNA1I	ENST00000402142.3	hsa-miR-182-3p	-0,01	-0,01
SLC36A1	ENST00000243389.3	hsa-miR-182-3p	-0,01	-0,01
NR3C2	ENST00000344721.4	hsa-miR-182-3p	-0,01	-0,01
ARHGEF4	ENST00000392953.3	hsa-miR-182-3p	-0,01	-0,01
PPFIA4	ENST00000447715.2	hsa-miR-182-3p	-0,01	-0,01
ATXN1L	ENST00000427980.2	hsa-miR-182-3p	-0,01	-0,01
REXO1L1	ENST00000379010.2	hsa-miR-182-3p	-0,01	-0,01
CACNG8	ENST00000270458.2	hsa-miR-182-3p	-0,01	-0,01
LMX1B	ENST00000355497.5	hsa-miR-182-3p	-0,01	-0,01
EP400	ENST00000333577.4	hsa-miR-182-3p	-0,01	-0,01
KIAA1755	ENST00000279024.4	hsa-miR-182-3p	-0,01	-0,01
HAS3	ENST00000306560.1	hsa-miR-182-3p	-0,01	-0,01
HIVEP3	ENST00000372583.1	hsa-miR-182-3p	-0,01	-0,01
KCNN3	ENST00000271915.4	hsa-miR-182-3p	-0,01	-0,01
MICAL3	ENST00000441493.2	hsa-miR-182-3p	-0,01	-0,01
NT5C1A	ENST00000235628.1	hsa-miR-182-3p	-0,01	-0,01
CTDSP1	ENST00000273062.2	hsa-miR-182-3p	-0,01	-0,01
PTPRS	ENST00000372412.4	hsa-miR-182-3p	-0,01	-0,01
CDON	ENST00000392693.3	hsa-miR-182-3p	-0,01	-0,1
C14orf37	ENST00000267485.7	hsa-miR-182-3p	-0,01	-0,05
NR6A1	ENST00000487099.2	hsa-miR-182-3p	-0,01	-0,01
SCARB2	ENST00000264896.2	hsa-miR-182-3p	-0,01	-0,26
EPB41L4B	ENST00000374557.4	hsa-miR-182-3p	-0,01	-0,11
UCHL5	ENST00000367455.4	hsa-miR-182-3p	-0,01	-0,17
SAR1B	ENST00000402673.2	hsa-miR-182-3p	-0,01	-0,34
RABGGTB	ENST00000319942.3	hsa-miR-182-3p	-0,01	-0,27
ERC1	ENST00000355446.5	hsa-miR-182-3p	-0,01	-0,01
SH3GLB1	ENST00000370558.4	hsa-miR-182-3p	-0,01	-0,19
ADRBK2	ENST00000324198.6	hsa-miR-182-3p	-0,01	-0,07
SLC6A6	ENST00000454876.2	hsa-miR-182-3p	-0,01	-0,01
RNF11	ENST00000242719.3	hsa-miR-182-3p	-0,01	-0,19
FAM149B1	ENST00000242505.6	hsa-miR-182-3p	-0,01	-0,06
KNTC1	ENST00000537348.1	hsa-miR-182-3p	-0,01	-0,17
ZNF426	ENST00000253115.2	hsa-miR-182-3p	-0,01	-0,04
DCPS	ENST00000263579.4	hsa-miR-182-3p	-0,01	-0,03
SOX11	ENST00000322002.3	hsa-miR-182-3p	-0,01	-0,05
TMEM11	ENST00000317635.5	hsa-miR-182-3p	-0,01	-0,1
GRID1	ENST00000327946.7	hsa-miR-182-3p	-0,01	-0,09
KLHL20	ENST00000209884.4	hsa-miR-182-3p	-0,01	-0,27
TM9SF3	ENST00000371142.4	hsa-miR-182-3p	-0,01	-0,05
SLC46A1	ENST00000440501.1	hsa-miR-182-3p	-0,01	-0,01

WDFY2	ENST00000298125.5	hsa-miR-182-3p	-0,01	-0,07
MYO1A	ENST00000300119.3	hsa-miR-182-3p	-0,01	-0,02
PTPN3	ENST00000412145.1	hsa-miR-182-3p	-0,01	-0,02
BACH2	ENST00000257749.4	hsa-miR-182-3p	-0,01	-0,01
ADAM23	ENST00000264377.3	hsa-miR-182-3p	-0,01	-0,02
ALG9	ENST00000531154.1	hsa-miR-182-3p	-0,01	-0,12
DDX20	ENST00000369702.4	hsa-miR-182-3p	-0,01	-0,04
FXN	ENST00000396366.2	hsa-miR-182-3p	-0,01	-0,06
KDM5B	ENST00000367265.3	hsa-miR-182-3p	-0,01	-0,06
RGS5	ENST00000313961.5	hsa-miR-182-3p	-0,01	-0,07
ADSL	ENST00000216194.7	hsa-miR-182-3p	-0,01	-0,17
TMED7	ENST00000456936.3	hsa-miR-182-3p	-0,01	-0,1
SOGA3	ENST00000556132.1	hsa-miR-182-3p	-0,01	-0,02
CDC42EP4	ENST00000335793.3	hsa-miR-182-3p	-0,01	-0,12
SOWAHC	ENST00000356454.3	hsa-miR-182-3p	-0,01	-0,01
ZC3H10	ENST00000257940.2	hsa-miR-182-3p	-0,01	-0,09
ECI1	ENST00000301729.4	hsa-miR-182-3p	-0,01	-0,26
FZD3	ENST00000240093.3	hsa-miR-182-3p	-0,01	-0,01
RRP8	ENST00000254605.6	hsa-miR-182-3p	-0,01	-0,14
LGALS8	ENST00000526589.1	hsa-miR-182-3p	-0,01	-0,08
ESRP2	ENST00000565858.1	hsa-miR-182-3p	-0,01	-0,14
NUDT3	ENST00000607016.1	hsa-miR-182-3p	-0,01	-0,02
RRP15	ENST00000366932.3	hsa-miR-182-3p	-0,01	-0,04
WIPF1	ENST00000392547.2	hsa-miR-182-3p	-0,01	-0,04
PHF3	ENST00000262043.3	hsa-miR-182-3p	-0,01	-0,17
NFE2L3	ENST00000056233.3	hsa-miR-182-3p	-0,01	-0,14
EEFSEC	ENST00000483457.1	hsa-miR-182-3p	-0,01	-0,15
MED1	ENST00000300651.6	hsa-miR-182-3p	-0,01	-0,01
FBXW2	ENST00000608872.1	hsa-miR-182-3p	-0,01	-0,07
RPAP2	ENST00000610020.1	hsa-miR-182-3p	-0,01	-0,32
IFRD1	ENST00000403825.3	hsa-miR-182-3p	-0,01	-0,3
USP47	ENST00000339865.5	hsa-miR-182-3p	-0,01	-0,06
CENPF	ENST00000366955.3	hsa-miR-182-3p	-0,01	-0,1
SLC35F5	ENST00000245680.2	hsa-miR-182-3p	-0,01	-0,15
IRS1	ENST00000305123.5	hsa-miR-182-3p	-0,01	-0,03
TMED5	ENST00000479918.1	hsa-miR-182-3p	-0,01	-0,06
TRPC4	ENST00000379705.3	hsa-miR-182-3p	-0,01	-0,07
PDXK	ENST00000468090.1	hsa-miR-182-3p	-0,01	-0,04
MARCH7	ENST00000259050.4	hsa-miR-182-3p	-0,01	-0,12
AGMAT	ENST00000375826.3	hsa-miR-182-3p	-0,01	-0,25
SSR1	ENST00000244763.4	hsa-miR-182-3p	-0,01	-0,2
ROCK2	ENST00000315872.6	hsa-miR-182-3p	-0,01	-0,03
LSM3	ENST00000306024.3	hsa-miR-182-3p	-0,01	-0,12
ZNF189	ENST00000374861.3	hsa-miR-182-3p	-0,01	-0,15
ERVMER34-1	ENST00000443173.1	hsa-miR-182-3p	-0,01	-0,07
ERCC6	ENST00000355832.5	hsa-miR-182-3p	-0,01	-0,11
SIPA1L2	ENST00000366630.1	hsa-miR-182-3p	-0,01	-0,17
ARPC2	ENST00000295685.10	hsa-miR-182-3p	-0,01	-0,03
ZNF324	ENST00000536459.2	hsa-miR-182-3p	-0,01	-0,06
SCIN	ENST00000297029.5	hsa-miR-182-3p	-0,01	-0,08
ALG11	ENST00000523764.1	hsa-miR-182-3p	-0,01	-0,19
RHBDL3	ENST00000269051.4	hsa-miR-182-3p	-0,01	-0,07
FARP1	ENST00000595437.1	hsa-miR-182-3p	-0,01	-0,07
VSNL1	ENST00000406397.1	hsa-miR-182-3p	-0,01	-0,32
GALNT1	ENST00000269195.5	hsa-miR-182-3p	-0,01	-0,18
SESN3	ENST00000536441.1	hsa-miR-182-3p	-0,01	-0,12
MYO9A	ENST00000564571.1	hsa-miR-182-3p	-0,01	-0,01
ZNF285	ENST00000330997.4	hsa-miR-182-3p	-0,01	-0,02
ZNF404	ENST00000587539.1	hsa-miR-182-3p	-0,01	-0,19
SLC35C2	ENST00000372227.1	hsa-miR-182-3p	-0,01	-0,26
ACADSB	ENST00000358776.4	hsa-miR-182-3p	-0,01	-0,07
SH3GL2	ENST00000380607.4	hsa-miR-182-3p	-0,01	-0,19
DPM3	ENST00000368399.1	hsa-miR-182-3p	-0,01	-0,31
SF3A1	ENST00000215793.8	hsa-miR-182-3p	-0,01	-0,11
PHKA2	ENST00000379942.4	hsa-miR-182-3p	-0,01	-0,05
SMU1	ENST00000397149.3	hsa-miR-182-3p	-0,01	-0,12

NUDT19	ENST00000397061.3	hsa-miR-182-3p	-0,01	-0,07
APTX	ENST00000436040.2	hsa-miR-182-3p	-0,01	-0,2
CALCRL	ENST00000392370.3	hsa-miR-182-3p	-0,01	-0,15
ARID3A	ENST00000263620.3	hsa-miR-182-3p	-0,01	-0,01
LBP	ENST00000217407.2	hsa-miR-182-3p	-0,01	-0,12
ZNF177	ENST00000541595.2	hsa-miR-182-3p	-0,01	-0,17
INTU	ENST00000335251.6	hsa-miR-182-3p	-0,01	-0,01
ACTR1A	ENST00000487599.1	hsa-miR-182-3p	-0,01	-0,1
VEGFC	ENST00000280193.2	hsa-miR-182-3p	-0,01	-0,09
INTS6	ENST00000311234.4	hsa-miR-182-3p	-0,01	-0,02
DCAF5	ENST00000341516.5	hsa-miR-182-3p	-0,01	-0,06
CEP89	ENST00000305768.5	hsa-miR-182-3p	-0,01	-0,07
CEP97	ENST00000341893.3	hsa-miR-182-3p	-0,01	-0,2
RAD50	ENST00000378823.3	hsa-miR-182-3p	-0,01	-0,03
HEPH	ENST00000519389.1	hsa-miR-182-3p	-0,01	-0,06
GPKOW	ENST00000156109.5	hsa-miR-182-3p	-0,01	-0,18
SLC9A7	ENST00000328306.4	hsa-miR-182-3p	-0,01	-0,01
PAGR1	ENST00000609618.1	hsa-miR-182-3p	-0,01	-0,13
GJC1	ENST00000426548.1	hsa-miR-182-3p	-0,01	-0,03
STXBP5	ENST00000367481.3	hsa-miR-182-3p	-0,01	-0,07
CMTM4	ENST00000394106.2	hsa-miR-182-3p	-0,01	-0,02
HDAC5	ENST00000225983.6	hsa-miR-182-3p	-0,01	-0,03
CEP68	ENST00000377990.2	hsa-miR-182-3p	-0,01	-0,07
NUFIP2	ENST00000225388.4	hsa-miR-182-3p	-0,01	-0,06
CCNT2	ENST00000295238.6	hsa-miR-182-3p	-0,01	-0,15
DDX52	ENST00000349699.2	hsa-miR-182-3p	-0,01	-0,1
DDR1	ENST00000446312.1	hsa-miR-182-3p	-0,01	-0,04
WAC	ENST00000375664.4	hsa-miR-182-3p	-0,01	-0,04
PPP1R9A	ENST00000289495.5	hsa-miR-182-3p	-0,01	-0,01
A1CF	ENST00000374001.2	hsa-miR-182-3p	-0,01	-0,03
TRAPPC13	ENST00000399438.3	hsa-miR-182-3p	-0,01	-0,24
CENPM	ENST00000404067.1	hsa-miR-182-3p	-0,01	-0,18
C1orf27	ENST00000287859.6	hsa-miR-182-3p	-0,01	-0,28
ZFP37	ENST00000374227.3	hsa-miR-182-3p	-0,01	-0,03
ESF1	ENST00000202816.1	hsa-miR-182-3p	-0,01	-0,07
RIMKLA	ENST00000431473.3	hsa-miR-182-3p	-0,01	-0,02
GMPR	ENST00000259727.4	hsa-miR-182-3p	-0,01	-0,13
TTLL7	ENST00000260505.8	hsa-miR-182-3p	-0,01	-0,11
ABAT	ENST00000569156.1	hsa-miR-182-3p	-0,01	-0,01
SERTAD4	ENST00000367012.3	hsa-miR-182-3p	-0,01	-0,02
PIAS1	ENST00000249636.6	hsa-miR-182-3p	-0,01	-0,04
ALDH6A1	ENST00000553458.1	hsa-miR-182-3p	-0,01	-0,15
COX15	ENST00000016171.5	hsa-miR-182-3p	-0,01	-0,12
TRAPPC2L	ENST00000565504.1	hsa-miR-182-3p	-0,01	-0,03
ZFYVE28	ENST00000508471.1	hsa-miR-182-3p	-0,01	-0,01
TMEM218	ENST00000532156.1	hsa-miR-182-3p	-0,01	-0,41
SLC2A8	ENST00000373360.3	hsa-miR-182-3p	-0,01	-0,11
NBN	ENST00000265433.3	hsa-miR-182-3p	-0,01	-0,07
MED28	ENST00000237380.7	hsa-miR-182-3p	-0,01	-0,22
MED20	ENST00000409312.1	hsa-miR-182-3p	-0,01	-0,21
NCAM1	ENST00000316851.7	hsa-miR-182-3p	-0,01	-0,02
EMC2	ENST00000220853.3	hsa-miR-182-3p	-0,01	-0,53
DNAJC15	ENST00000379221.2	hsa-miR-182-3p	-0,01	-0,32
TNIK	ENST00000436636.2	hsa-miR-182-3p	-0,01	-0,1
TCEAL4	ENST00000472484.1	hsa-miR-182-3p	-0,01	-0,29
SLC41A2	ENST00000258538.3	hsa-miR-182-3p	-0,01	-0,17
ZNF670	ENST00000366503.2	hsa-miR-182-3p	-0,01	-0,26
TRMT10B	ENST00000297994.3	hsa-miR-182-3p	-0,01	-0,13
TMEM98	ENST00000579849.1	hsa-miR-182-3p	-0,01	-0,1
AKT2	ENST00000392038.2	hsa-miR-182-3p	-0,01	-0,02
TMEM180	ENST00000238936.4	hsa-miR-182-3p	-0,01	-0,03
MAGIX	ENST00000376338.3	hsa-miR-182-3p	-0,01	-0,12
NEK10	ENST00000295720.6	hsa-miR-182-3p	0	-0,1
TRMT5	ENST00000261249.6	hsa-miR-182-3p	0	-0,07
ABCD4	ENST00000356924.4	hsa-miR-182-3p	0	-0,14
TIMP3	ENST00000266085.6	hsa-miR-182-3p	0	-0,04

STAMBPL1	ENST00000371926.3	hsa-miR-182-3p	0	-0,1
SNX24	ENST00000513881.1	hsa-miR-182-3p	0	-0,1
NEK3	ENST00000339406.3	hsa-miR-182-3p	0	-0,15
PPP3CC	ENST00000289963.8	hsa-miR-182-3p	0	-0,12
PELI3	ENST00000320740.7	hsa-miR-182-3p	0	-0,03
MBD2	ENST00000256429.3	hsa-miR-182-3p	0	-0,3
DNAL1	ENST00000553645.2	hsa-miR-182-3p	0	-0,08
CISD3	ENST00000439660.2	hsa-miR-182-3p	0	-0,1
KAL1	ENST00000262648.3	hsa-miR-182-3p	0	-0,01
HEXIM2	ENST00000307275.3	hsa-miR-182-3p	0	-0,18
SLC48A1	ENST00000442218.2	hsa-miR-182-3p	0	-0,1
ANKHD1-EIF4EBP3	ENST00000532219.1	hsa-miR-182-3p	0	-0,1
GPRASP2	ENST00000543253.1	hsa-miR-182-3p	0	-0,19
CDH6	ENST00000265071.2	hsa-miR-182-3p	0	-0,03
AAK1	ENST00000409085.4	hsa-miR-182-3p	0	-0,01
SDHAF2	ENST00000543265.1	hsa-miR-182-3p	0	-0,31
PDPR	ENST00000568530.1	hsa-miR-182-3p	0	-0,06
FUNDC2	ENST00000369498.3	hsa-miR-182-3p	0	-0,12
SUSD1	ENST00000374263.3	hsa-miR-182-3p	0	-0,04
SEMA6A	ENST00000343348.6	hsa-miR-182-3p	0	-0,05
PDS5A	ENST00000303538.8	hsa-miR-182-3p	0	-0,08
OBFC1	ENST00000224950.3	hsa-miR-182-3p	0	-0,06
LEF1	ENST00000265165.1	hsa-miR-182-3p	0	-0,05
VWDE	ENST00000275358.3	hsa-miR-182-3p	0	-0,01
PTCHD1	ENST00000379361.4	hsa-miR-182-3p	0	-0,04
ADAMTS4	ENST00000367996.5	hsa-miR-182-3p	0	-0,02
NAV1	ENST00000295624.6	hsa-miR-182-3p	0	-0,01
FZD1	ENST00000287934.2	hsa-miR-182-3p	0	-0,14
EXO1	ENST00000518483.1	hsa-miR-182-3p	0	-0,09
SERPINA4	ENST00000555095.1	hsa-miR-182-3p	0	-0,14
DHRS7B	ENST00000395511.3	hsa-miR-182-3p	0	-0,06
TMEM39A	ENST00000319172.5	hsa-miR-182-3p	0	-0,05
SPECC1	ENST00000395530.2	hsa-miR-182-3p	0	-0,1
EED	ENST00000327320.4	hsa-miR-182-3p	0	-0,16
COBLL1	ENST00000375458.2	hsa-miR-182-3p	0	-0,03
UBE2W	ENST00000517608.1	hsa-miR-182-3p	0	-0,08
RBFA	ENST00000262197.7	hsa-miR-182-3p	0	-0,1
ZBTB25	ENST00000608382.1	hsa-miR-182-3p	0	-0,03
MRPS11	ENST00000325844.4	hsa-miR-182-3p	0	-0,04
SRFBP1	ENST00000339397.4	hsa-miR-182-3p	0	-0,14
BRIX1	ENST00000336767.5	hsa-miR-182-3p	0	-0,17
HM13	ENST00000335574.5	hsa-miR-182-3p	0	-0,13
TUSC2	ENST00000232496.4	hsa-miR-182-3p	0	-0,16
SLC45A4	ENST00000519067.1	hsa-miR-182-3p	0	-0,06
GFRA1	ENST00000439649.3	hsa-miR-182-3p	0	-0,05
FGL2	ENST00000248598.5	hsa-miR-182-3p	0	-0,15
FNBP1	ENST00000355681.3	hsa-miR-182-3p	0	-0,02
RFC5	ENST00000229043.3	hsa-miR-182-3p	0	-0,11
ABCB1	ENST00000265724.3	hsa-miR-182-3p	0	-0,08
SPHAR	ENST00000366688.3	hsa-miR-182-3p	0	-0,13
HPX	ENST00000265983.3	hsa-miR-182-3p	0	-0,06
UBE2N	ENST00000550657.1	hsa-miR-182-3p	0	-0,21
PLEKHA3	ENST00000234453.5	hsa-miR-182-3p	0	-0,06
NSL1	ENST00000422588.2	hsa-miR-182-3p	0	-0,03
KRR1	ENST00000229214.4	hsa-miR-182-3p	0	-0,02
SUGT1	ENST00000310528.8	hsa-miR-182-3p	0	-0,02
MAP2K6	ENST00000590474.1	hsa-miR-182-3p	0	-0,02
WDR5B	ENST00000330689.4	hsa-miR-182-3p	0	-0,04
UBD	ENST00000377050.4	hsa-miR-182-3p	0	-0,13
CADM1	ENST00000452722.3	hsa-miR-182-3p	0	-0,32
RNPEPL1	ENST00000270357.4	hsa-miR-182-3p	0	-0,17
CLPB	ENST00000294053.3	hsa-miR-182-3p	0	-0,02
SUMO2	ENST00000420826.2	hsa-miR-182-3p	0	-0,07
WDR53	ENST00000332629.5	hsa-miR-182-3p	0	-0,11
ZNF639	ENST00000326361.3	hsa-miR-182-3p	0	-0,1
ANXA11	ENST00000372231.3	hsa-miR-182-3p	0	-0,03

GNB1L	ENST00000329517.6	hsa-miR-182-3p	0	-0,14
DCTN6	ENST00000221114.3	hsa-miR-182-3p	0	-0,14
CDKN2C	ENST00000262662.1	hsa-miR-182-3p	0	-0,2
IKZF3	ENST00000346872.3	hsa-miR-182-3p	0	-0,01
NUP205	ENST00000285968.6	hsa-miR-182-3p	0	-0,01
PHB2	ENST00000546111.1	hsa-miR-182-3p	0	-0,12
ZNF71	ENST00000328070.6	hsa-miR-182-3p	0	-0,03
PAOX	ENST00000368539.4	hsa-miR-182-3p	0	-0,11
SRSF3	ENST00000373715.6	hsa-miR-182-3p	0	-0,19
NOTCH1	ENST00000277541.6	hsa-miR-182-3p	0	-0,01
F10	ENST00000375551.3	hsa-miR-182-3p	0	-0,05
OXSM	ENST00000280701.3	hsa-miR-182-3p	0	-0,17
FANCB	ENST00000324138.3	hsa-miR-182-3p	0	-0,1
MBOAT1	ENST00000541730.1	hsa-miR-182-3p	0	-0,08
TPBG	ENST00000369750.3	hsa-miR-182-3p	0	-0,1
SNAP47	ENST00000366760.1	hsa-miR-182-3p	0	-0,19
TTLL5	ENST00000298832.9	hsa-miR-182-3p	0	-0,34
TTC4	ENST00000371281.3	hsa-miR-182-3p	0	-0,14
TACO1	ENST00000258975.6	hsa-miR-182-3p	0	-0,15
TRIM38	ENST00000357085.3	hsa-miR-182-3p	0	-0,04
COX10	ENST00000261643.3	hsa-miR-182-3p	0	-0,06
TMEM135	ENST00000340353.7	hsa-miR-182-3p	0	-0,13
SFXN2	ENST00000369893.5	hsa-miR-182-3p	0	-0,17
NAGS	ENST00000293404.3	hsa-miR-182-3p	0	-0,1
KB-1507C5.2	ENST00000524007.1	hsa-miR-182-3p	0	-0,19
INPP4A	ENST00000409016.4	hsa-miR-182-3p	0	-0,06
UQCRB	ENST00000287022.5	hsa-miR-182-3p	0	-0,1
STK25	ENST00000316586.4	hsa-miR-182-3p	0	-0,1
COMTD1	ENST00000372538.3	hsa-miR-182-3p	0	-0,17
POLK	ENST00000241436.4	hsa-miR-182-3p	0	-0,15
ORC4	ENST00000392857.5	hsa-miR-182-3p	0	-0,29
FAM96A	ENST00000557835.1	hsa-miR-182-3p	0	-0,15
CSRP2BP	ENST00000435364.3	hsa-miR-182-3p	0	-0,11
GNB2L1	ENST00000514455.1	hsa-miR-182-3p	0	-0,46
NDUFB9	ENST00000276689.3	hsa-miR-182-3p	0	-0,14
ST5	ENST00000526757.1	hsa-miR-182-3p	0	-0,24
FOXRED2	ENST00000397224.4	hsa-miR-182-3p	0	-0,1
FAM179A	ENST00000379558.4	hsa-miR-182-3p	0	-0,01
KCMF1	ENST00000409785.4	hsa-miR-182-3p	0	-0,21
CGRRF1	ENST00000216420.7	hsa-miR-182-3p	0	-0,11
CEP128	ENST00000281129.3	hsa-miR-182-3p	0	-0,18
GRK6	ENST00000393576.3	hsa-miR-182-3p	0	-0,04
DLK1	ENST00000341267.4	hsa-miR-182-3p	0	-0,11
AGPS	ENST00000264167.4	hsa-miR-182-3p	0	-0,25
GAB2	ENST00000340149.2	hsa-miR-182-3p	0	-0,06
LRIG3	ENST00000379141.4	hsa-miR-182-3p	0	-0,04
PAPOLA	ENST00000557471.1	hsa-miR-182-3p	0	-0,26
ITGB5	ENST00000296181.4	hsa-miR-182-3p	0	-0,17
ZSCAN2	ENST00000541040.1	hsa-miR-182-3p	0	-0,02
RNF20	ENST00000389120.3	hsa-miR-182-3p	0	-0,03
MSX2	ENST00000239243.6	hsa-miR-182-3p	0	-0,11
STX7	ENST00000367941.2	hsa-miR-182-3p	0	-0,34
C17orf85	ENST00000158149.3	hsa-miR-182-3p	0	-0,05
GGCX	ENST00000233838.4	hsa-miR-182-3p	0	-0,11
UROS	ENST00000368797.4	hsa-miR-182-3p	0	-0,15
TRIM27	ENST00000377194.3	hsa-miR-182-3p	0	-0,2
UFC1	ENST00000368003.5	hsa-miR-182-3p	0	-0,23
TNFAIP8	ENST00000504771.2	hsa-miR-182-3p	0	-0,06
TBC1D19	ENST00000264866.4	hsa-miR-182-3p	0	-0,03
NFATC4	ENST00000413692.2	hsa-miR-182-3p	0	-0,03
NGDN	ENST00000397154.3	hsa-miR-182-3p	0	-0,29
NABP1	ENST00000410026.2	hsa-miR-182-3p	0	-0,03
PAX6	ENST00000419022.1	hsa-miR-182-3p	0	-0,07
ARRB1	ENST00000420843.2	hsa-miR-182-3p	0	-0,01
PFDN4	ENST00000371419.2	hsa-miR-182-3p	0	-0,16
INTS8	ENST00000523731.1	hsa-miR-182-3p	0	-0,22

MCM5	ENST00000216122.4	hsa-miR-182-3p	0	-0,08
EFHC1	ENST00000371068.5	hsa-miR-182-3p	0	-0,01
ZMYM2	ENST00000382869.3	hsa-miR-182-3p	0	-0,06
TRMT10C	ENST00000309922.6	hsa-miR-182-3p	0	-0,21
ASTN2	ENST00000341734.4	hsa-miR-182-3p	0	-0,03
SMC6	ENST00000448223.2	hsa-miR-182-3p	0	-0,01
NGRN	ENST00000379095.3	hsa-miR-182-3p	0	-0,1
RPS23	ENST00000296674.8	hsa-miR-182-3p	0	-0,31
ACP6	ENST00000369238.6	hsa-miR-182-3p	0	-0,24
MAPK14	ENST00000229795.3	hsa-miR-182-3p	0	-0,15
RAB3C	ENST00000282878.4	hsa-miR-182-3p	0	-0,02
TSPAN6	ENST00000373020.4	hsa-miR-182-3p	0	-0,13
TBC1D15	ENST00000550746.1	hsa-miR-182-3p	0	-0,1
SCFD2	ENST00000401642.3	hsa-miR-182-3p	0	-0,15
TIGD6	ENST00000296736.3	hsa-miR-182-3p	0	-0,09
ERO1LB	ENST00000354619.5	hsa-miR-182-3p	0	-0,15
SUPT7L	ENST00000337768.5	hsa-miR-182-3p	0	-0,14
FOCAD	ENST00000380249.1	hsa-miR-182-3p	0	-0,08
AHSA2	ENST00000394457.3	hsa-miR-182-3p	0	-0,15
CHRNA5	ENST00000299565.5	hsa-miR-182-3p	0	-0,3
PSMB7	ENST00000259457.3	hsa-miR-182-3p	0	-0,16
MKKS	ENST00000347364.3	hsa-miR-182-3p	0	-0,1
NTPCR	ENST00000366628.5	hsa-miR-182-3p	0	-0,1
RPL31	ENST00000409320.3	hsa-miR-182-3p	0	-0,44
FUT4	ENST00000358752.2	hsa-miR-182-3p	0	-0,06
DCAF10	ENST00000242323.7	hsa-miR-182-3p	0	-0,07
KLHL5	ENST00000261425.3	hsa-miR-182-3p	0	-0,14
TUBD1	ENST00000346141.6	hsa-miR-182-3p	0	-0,15
IKBKAP	ENST00000374647.5	hsa-miR-182-3p	0	-0,07
MRGPRF	ENST00000441623.1	hsa-miR-182-3p	0	-0,04
NUPL2	ENST00000258742.5	hsa-miR-182-3p	0	-0,17
GFM1	ENST00000486715.1	hsa-miR-182-3p	0	-0,02
PCDHB2	ENST00000194155.4	hsa-miR-182-3p	0	-0,11
HPS1	ENST00000361490.4	hsa-miR-182-3p	0	-0,09
CLNS1A	ENST00000525428.1	hsa-miR-182-3p	0	-0,15
TMEM246	ENST00000374847.1	hsa-miR-182-3p	0	-0,16
KCTD7	ENST00000275532.3	hsa-miR-182-3p	0	-0,19
LONP2	ENST00000285737.4	hsa-miR-182-3p	0	-0,01
POLR2E	ENST00000215587.7	hsa-miR-182-3p	0	-0,2
CDKN2AIP	ENST00000302350.4	hsa-miR-182-3p	0	-0,15
TPT1	ENST00000379056.1	hsa-miR-182-3p	0	-0,08
AKT1	ENST00000554581.1	hsa-miR-182-3p	0	-0,06
RCAN1	ENST00000482533.1	hsa-miR-182-3p	0	-0,18
SEPHS1	ENST00000545675.1	hsa-miR-182-3p	0	-0,07
ST3GAL6	ENST00000265261.6	hsa-miR-182-3p	0	-0,13
PYCR1	ENST00000329875.8	hsa-miR-182-3p	0	-0,09
ISL2	ENST00000290759.4	hsa-miR-182-3p	0	-0,01
GTPBP8	ENST00000383677.3	hsa-miR-182-3p	0	-0,13
LETMD1	ENST00000380123.2	hsa-miR-182-3p	0	-0,45
VIL1	ENST00000248444.5	hsa-miR-182-3p	0	-0,09
NSDHL	ENST00000370274.3	hsa-miR-182-3p	0	-0,17
SMIM4	ENST00000477703.1	hsa-miR-182-3p	0	-0,76
RBM15B	ENST00000323686.4	hsa-miR-182-3p	0	-0,03
PRSS23	ENST00000280258.5	hsa-miR-182-3p	0	-0,19
G6PC3	ENST00000269097.4	hsa-miR-182-3p	0	-0,21
LIG3	ENST00000378526.4	hsa-miR-182-3p	0	-0,02
AGPAT6	ENST00000396987.3	hsa-miR-182-3p	0	-0,02
RAE1	ENST00000395841.2	hsa-miR-182-3p	0	-0,14
PQLC2	ENST00000375155.3	hsa-miR-182-3p	0	-0,01
NPC2	ENST00000541064.1	hsa-miR-182-3p	0	-0,28
TMEM194B	ENST00000409150.3	hsa-miR-182-3p	0	-0,05
C1orf112	ENST00000359326.4	hsa-miR-182-3p	0	-0,24
MON2	ENST00000546600.1	hsa-miR-182-3p	0	-0,08
KDM5C	ENST00000375401.3	hsa-miR-182-3p	0	-0,2
GTF2B	ENST00000370500.5	hsa-miR-182-3p	0	-0,2
POMT2	ENST00000261534.4	hsa-miR-182-3p	0	-0,03

LIPC	ENST00000433326.2	hsa-miR-182-3p	0	-0,12
CLCC1	ENST00000356970.2	hsa-miR-182-3p	0	-0,14
C3orf17	ENST00000314400.5	hsa-miR-182-3p	0	-0,12
DMXL1	ENST00000311085.8	hsa-miR-182-3p	0	-0,02
RBP4	ENST00000371464.3	hsa-miR-182-3p	0	-0,17
VPS18	ENST00000220509.5	hsa-miR-182-3p	0	-0,02
HINFP	ENST00000350777.2	hsa-miR-182-3p	0	-0,08
SMARCA5	ENST00000283131.3	hsa-miR-182-3p	0	-0,1
BRMS1L	ENST00000216807.7	hsa-miR-182-3p	0	-0,05
GAS7	ENST00000437099.2	hsa-miR-182-3p	0	-0,01
KIAA1551	ENST00000312561.4	hsa-miR-182-3p	0	-0,01
CKAP4	ENST00000378026.4	hsa-miR-182-3p	0	-0,04
TMEM199	ENST00000292114.3	hsa-miR-182-3p	0	-0,08
G6PC2	ENST00000421979.1	hsa-miR-182-3p	0	-0,53
PRMT6	ENST00000370078.1	hsa-miR-182-3p	0	-0,16
PHKG2	ENST00000563588.1	hsa-miR-182-3p	0	-0,02
GOSR1	ENST00000225724.5	hsa-miR-182-3p	0	-0,01
FAH	ENST00000407106.1	hsa-miR-182-3p	0	-0,41
SHB	ENST00000377707.3	hsa-miR-182-3p	0	-0,02
DSN1	ENST00000426836.1	hsa-miR-182-3p	0	-0,19
CMC1	ENST00000466830.1	hsa-miR-182-3p	0	-0,2
POFUT2	ENST00000349485.5	hsa-miR-182-3p	0	-0,14
CHD9	ENST00000566029.1	hsa-miR-182-3p	0	-0,07
WEE1	ENST00000299613.6	hsa-miR-182-3p	0	-0,09
EMC7	ENST00000256545.4	hsa-miR-182-3p	0	-0,39
PHIP	ENST00000275034.4	hsa-miR-182-3p	0	-0,11
RPL37A	ENST00000491306.1	hsa-miR-182-3p	0	-0,31
RBM46	ENST00000514866.1	hsa-miR-182-3p	0	-0,47
CCDC149	ENST00000428116.2	hsa-miR-182-3p	0	-0,05
IGSF3	ENST00000369486.3	hsa-miR-182-3p	0	-0,02
C19orf10	ENST00000262947.3	hsa-miR-182-3p	0	-0,15
HMGCR	ENST00000287936.4	hsa-miR-182-3p	0	-0,14
FZD2	ENST00000315323.3	hsa-miR-182-3p	0	-0,12
ERP44	ENST00000262455.6	hsa-miR-182-3p	0	-0,06
GHITM	ENST00000372134.3	hsa-miR-182-3p	0	-0,17
WIPI2	ENST00000288828.4	hsa-miR-182-3p	0	-0,07
WBP11	ENST00000261167.2	hsa-miR-182-3p	0	-0,13
VANGL1	ENST00000355485.2	hsa-miR-182-3p	0	-0,03
RPL10L	ENST00000298283.3	hsa-miR-182-3p	0	-0,38
PLXNA3	ENST00000369682.3	hsa-miR-182-3p	0	-0,07
PTGDR	ENST00000553372.1	hsa-miR-182-3p	0	-0,37
SPCS1	ENST00000233025.7	hsa-miR-182-3p	0	-0,65
SCO1	ENST00000255390.5	hsa-miR-182-3p	0	-0,06
LRRC4C	ENST00000278198.2	hsa-miR-182-3p	0	-0,35
SGIP1	ENST00000371036.3	hsa-miR-182-3p	0	-0,1
PPIP5K2	ENST00000321521.9	hsa-miR-182-3p	0	-0,01
ACYP2	ENST00000303536.4	hsa-miR-182-3p	0	-0,3
CCDC84	ENST00000334418.1	hsa-miR-182-3p	0	-0,33
TIMMDC1	ENST00000494664.1	hsa-miR-182-3p	0	-0,18
RAB4A	ENST00000366690.4	hsa-miR-182-3p	0	-0,05
COLEC10	ENST00000332843.2	hsa-miR-182-3p	0	-0,36
SLITRK4	ENST00000381779.4	hsa-miR-182-3p	0	-0,01
EPPK1	ENST00000525985.1	hsa-miR-182-3p	0	-0,01
PLCG1	ENST00000244007.3	hsa-miR-182-3p	0	-0,02
MRPL36	ENST00000505059.2	hsa-miR-182-3p	0	-0,07
AKR1D1	ENST00000432161.1	hsa-miR-182-3p	0	-0,29
CLEC12B	ENST00000338896.5	hsa-miR-182-3p	0	-0,25
MED14	ENST00000324817.1	hsa-miR-182-3p	0	-0,11
CSE1L	ENST00000262982.2	hsa-miR-182-3p	0	-0,02
CFD	ENST00000327726.6	hsa-miR-182-3p	0	-0,01
GALNT8	ENST00000252318.2	hsa-miR-182-3p	0	-0,28
HSPH1	ENST00000320027.5	hsa-miR-182-3p	0	-0,16
BAG1	ENST00000472232.3	hsa-miR-182-3p	0	-0,23
POLR3A	ENST00000372371.3	hsa-miR-182-3p	0	-0,02
S1PR1	ENST00000305352.6	hsa-miR-182-3p	0	-0,05
ECHDC3	ENST00000379215.4	hsa-miR-182-3p	0	-0,04

GIMAP6	ENST00000493969.1	hsa-miR-182-3p	0	-0,3
C12orf73	ENST00000547975.1	hsa-miR-182-3p	0	-0,19
MGAT4C	ENST00000604798.1	hsa-miR-182-3p	0	-0,3
SPTY2D1	ENST00000336349.5	hsa-miR-182-3p	0	-0,02
CHSY1	ENST00000254190.3	hsa-miR-182-3p	0	-0,02
ABRACL	ENST00000367660.3	hsa-miR-182-3p	0	-0,06
SIX1	ENST00000247182.6	hsa-miR-182-3p	0	-0,12
ZBTB14	ENST00000357006.4	hsa-miR-182-3p	0	-0,02
PPIF	ENST00000394579.3	hsa-miR-182-3p	0	-0,15
NCKAP1	ENST00000361354.4	hsa-miR-182-3p	0	-0,06
EVI2B	ENST00000330927.4	hsa-miR-182-3p	0	-0,24
MOBP	ENST00000420739.1	hsa-miR-182-3p	0	-0,21
PLGLB1	ENST00000355705.3	hsa-miR-182-3p	0	-0,21
FAM19A1	ENST00000478136.1	hsa-miR-182-3p	0	-0,2
ISY1	ENST00000393292.3	hsa-miR-182-3p	0	-0,06
CAPN7	ENST00000253693.2	hsa-miR-182-3p	0	-0,02
PLGLB2	ENST00000359481.4	hsa-miR-182-3p	0	-0,21
PIGP	ENST00000360525.4	hsa-miR-182-3p	0	-0,12
TMEM115	ENST00000266025.3	hsa-miR-182-3p	0	-0,26
RBM18	ENST00000417201.3	hsa-miR-182-3p	0	-0,07
WDR77	ENST00000235090.5	hsa-miR-182-3p	0	-0,07
SIPA1	ENST00000534313.1	hsa-miR-182-3p	0	-0,02
ECHDC2	ENST00000371522.4	hsa-miR-182-3p	0	-0,03
ENPP6	ENST00000296741.2	hsa-miR-182-3p	0	-0,19
GPR22	ENST00000304402.4	hsa-miR-182-3p	0	-0,2
ACKR2	ENST00000442925.1	hsa-miR-182-3p	0	-0,24
RBM25	ENST00000261973.7	hsa-miR-182-3p	0	-0,08
CDR1	ENST00000370532.2	hsa-miR-182-3p	0	-0,22
DCUN1D5	ENST00000260247.5	hsa-miR-182-3p	0	-0,12
SMIM17	ENST00000598409.1	hsa-miR-182-3p	0	-0,23
QDPR	ENST00000508623.1	hsa-miR-182-3p	0	-0,1
LRRTM3	ENST00000361320.4	hsa-miR-182-3p	0	-0,23
PFN4	ENST00000313213.4	hsa-miR-182-3p	0	-0,3
TSC22D2	ENST00000361875.3	hsa-miR-182-3p	0	-0,1
C10orf71	ENST00000323868.4	hsa-miR-182-3p	0	-0,2
RP11-210M15.2	ENST00000559008.1	hsa-miR-182-3p	0	-0,15
VCAN	ENST00000265077.3	hsa-miR-182-3p	0	-0,1
TP53TG5	ENST00000372726.3	hsa-miR-182-3p	0	-0,18
CABP4	ENST00000438189.2	hsa-miR-182-3p	0	-0,15
AGPAT3	ENST00000291572.8	hsa-miR-182-3p	0	-0,36
CTC1	ENST00000315684.8	hsa-miR-182-3p	0	-0,04
IL9R	ENST00000424344.3	hsa-miR-182-3p	0	-0,15
MAL	ENST00000309988.4	hsa-miR-182-3p	0	-0,18
XPNPEP2	ENST00000371106.3	hsa-miR-182-3p	0	-0,14
XRCC5	ENST00000392133.3	hsa-miR-182-3p	0	-0,19
TGIF2LX	ENST00000283891.5	hsa-miR-182-3p	0	-0,16
GRIN2A	ENST00000562109.1	hsa-miR-182-3p	0	-0,18
OMD	ENST00000375550.4	hsa-miR-182-3p	0	-0,13
FHL5	ENST00000541107.1	hsa-miR-182-3p	0	-0,15
ZNF843	ENST00000315678.5	hsa-miR-182-3p	0	-0,14
CUL3	ENST00000264414.4	hsa-miR-182-3p	0	-0,03
BCKDK	ENST00000394950.3	hsa-miR-182-3p	0	-0,13
MAPK1	ENST00000215832.6	hsa-miR-182-3p	0	-0,04
CLEC4C	ENST00000542353.1	hsa-miR-182-3p	0	-0,14
NAALAD2	ENST00000534061.1	hsa-miR-182-3p	0	-0,15
F5	ENST00000367797.3	hsa-miR-182-3p	0	-0,17
CHRDL1	ENST00000372045.1	hsa-miR-182-3p	0	-0,15
KNCN	ENST00000481882.2	hsa-miR-182-3p	0	-0,15
GLYAT	ENST00000344743.3	hsa-miR-182-3p	0	-0,18
CRISP1	ENST00000335847.4	hsa-miR-182-3p	0	-0,15
F2	ENST00000311907.5	hsa-miR-182-3p	0	-0,17
IGSF10	ENST00000282466.3	hsa-miR-182-3p	0	-0,01
SLC29A3	ENST00000373189.5	hsa-miR-182-3p	0	-0,02
INHBC	ENST00000309668.2	hsa-miR-182-3p	0	-0,09
CHRND	ENST00000258385.3	hsa-miR-182-3p	0	-0,1
ABI2	ENST00000295851.5	hsa-miR-182-3p	0	-0,04

CDC42EP1	ENST00000249014.4	hsa-miR-182-3p	0	-0,14
SVOP	ENST00000299134.5	hsa-miR-182-3p	0	-0,15
CYSLTR1	ENST00000373304.3	hsa-miR-182-3p	0	-0,09
MGAT1	ENST00000333055.3	hsa-miR-182-3p	0	-0,01
NLRP9	ENST00000332836.2	hsa-miR-182-3p	0	-0,11
PPIL1	ENST00000373699.5	hsa-miR-182-3p	0	-0,1
POU2F2	ENST00000342301.4	hsa-miR-182-3p	0	-0,11
SMC1A	ENST00000322213.4	hsa-miR-182-3p	0	-0,02
RGPD8	ENST00000302558.3	hsa-miR-182-3p	0	-0,08
RGPD5	ENST00000016946.3	hsa-miR-182-3p	0	-0,08
FAM162B	ENST00000368557.4	hsa-miR-182-3p	0	-0,22
CRIPT	ENST00000238892.3	hsa-miR-182-3p	0	-0,09
ASAH2B	ENST00000374007.1	hsa-miR-182-3p	0	-0,11
BTN3A2	ENST00000396948.1	hsa-miR-182-3p	0	-0,12
AKR7A2	ENST00000235835.3	hsa-miR-182-3p	0	-0,03
POTED	ENST00000299443.5	hsa-miR-182-3p	0	-0,11
GLUL	ENST00000331872.6	hsa-miR-182-3p	0	-0,03
ANGPTL1	ENST00000234816.2	hsa-miR-182-3p	0	-0,13
METTL24	ENST00000338882.4	hsa-miR-182-3p	0	-0,13
PCDH11X	ENST00000504220.2	hsa-miR-182-3p	0	-0,13
LAIR1	ENST00000391743.3	hsa-miR-182-3p	0	-0,08
RBCK1	ENST00000356286.5	hsa-miR-182-3p	0	-0,13
RNF213	ENST00000582970.1	hsa-miR-182-3p	0	-0,03
SLC25A4	ENST00000281456.6	hsa-miR-182-3p	0	-0,04
DOCK7	ENST00000251157.5	hsa-miR-182-3p	0	-0,02
MRPL11	ENST00000329819.4	hsa-miR-182-3p	0	-0,09
COL19A1	ENST00000322773.4	hsa-miR-182-3p	0	-0,09
NBPF24	ENST00000369226.3	hsa-miR-182-3p	0	-0,1
CAPZA2	ENST00000361183.3	hsa-miR-182-3p	0	-0,09
NUP88	ENST00000573584.1	hsa-miR-182-3p	0	-0,06
TRMU	ENST00000381019.3	hsa-miR-182-3p	0	-0,01
GTF3C2	ENST00000359541.2	hsa-miR-182-3p	0	-0,21
DNAJC10	ENST00000264065.7	hsa-miR-182-3p	0	-0,02
RGPD6	ENST00000329516.3	hsa-miR-182-3p	0	-0,08
ADORA3	ENST00000369716.4	hsa-miR-182-3p	0	-0,12
TGFBI	ENST00000442011.2	hsa-miR-182-3p	0	-0,06
MTO1	ENST00000498286.1	hsa-miR-182-3p	0	-0,01
ACSM2B	ENST00000329697.6	hsa-miR-182-3p	0	-0,21
G6PC	ENST00000253801.2	hsa-miR-182-3p	0	-0,06
EBF4	ENST00000380648.4	hsa-miR-182-3p	0	-0,05
ABCB11	ENST00000263817.6	hsa-miR-182-3p	0	-0,07
SOD2	ENST00000538183.2	hsa-miR-182-3p	0	-0,01
TSHR	ENST00000541158.2	hsa-miR-182-3p	0	-0,05
CYP2C8	ENST00000371270.3	hsa-miR-182-3p	0	-0,14
ZBTB20	ENST00000462705.1	hsa-miR-182-3p	0	-0,07
CDH18	ENST00000382275.1	hsa-miR-182-3p	0	-0,07
YAE1D1	ENST00000223273.2	hsa-miR-182-3p	0	-0,37
APRT	ENST00000426324.2	hsa-miR-182-3p	0	-0,19
FNDC5	ENST00000496770.1	hsa-miR-182-3p	0	-0,05
ANKRD32	ENST00000265140.5	hsa-miR-182-3p	0	-0,02
KY	ENST00000508956.1	hsa-miR-182-3p	0	-0,06
TG	ENST00000519543.1	hsa-miR-182-3p	0	-0,04
ENPP5	ENST00000371383.2	hsa-miR-182-3p	0	-0,11
PAM	ENST00000438793.3	hsa-miR-182-3p	0	-0,21
SLC19A1	ENST00000311124.4	hsa-miR-182-3p	0	-0,04
ACSM2A	ENST00000573854.1	hsa-miR-182-3p	0	-0,29
BMP10	ENST00000295379.1	hsa-miR-182-3p	0	-0,03
KRBA1	ENST00000255992.10	hsa-miR-182-3p	0	-0,02
ATP5G3	ENST00000284727.4	hsa-miR-182-3p	0	-0,36
CD1D	ENST00000368171.3	hsa-miR-182-3p	0	-0,09
PFKM	ENST00000312352.7	hsa-miR-182-3p	0	-0,02
SHMT1	ENST00000316694.3	hsa-miR-182-3p	0	-0,2
HSPA14	ENST00000378372.3	hsa-miR-182-3p	0	-0,07
BSND	ENST00000371265.4	hsa-miR-182-3p	0	-0,03
EPS8	ENST00000543523.1	hsa-miR-182-3p	0	-0,03
HLA-DRB1	ENST00000360004.5	hsa-miR-182-3p	0	-0,4

KIAA1468	ENST00000398130.2	hsa-miR-182-3p	0	-0,03
UGT2B4	ENST00000305107.6	hsa-miR-182-3p	0	-0,07
NSMCE1	ENST00000361439.4	hsa-miR-182-3p	0	-0,01
NUDCD3	ENST00000355451.7	hsa-miR-182-3p	0	-0,01
CCS	ENST00000533244.1	hsa-miR-182-3p	0	-0,05
KDM3A	ENST00000409556.1	hsa-miR-182-3p	0	-0,07
LSM10	ENST00000315732.2	hsa-miR-182-3p	0	-0,02
ASTN1	ENST00000361833.2	hsa-miR-182-3p	0	-0,09
TRDN	ENST00000398178.3	hsa-miR-182-3p	0	-0,06
APOBEC3F	ENST00000308521.5	hsa-miR-182-3p	0	-0,07
ADCYAP1R1	ENST00000304166.4	hsa-miR-182-3p	0	-0,14
GPR174	ENST00000276077.1	hsa-miR-182-3p	0	-0,05
SLFN12L	ENST00000260908.7	hsa-miR-182-3p	0	-0,04
FAM20A	ENST00000592554.1	hsa-miR-182-3p	0	-0,2
PLSCR1	ENST00000342435.4	hsa-miR-182-3p	0	-0,07
ETV4	ENST00000319349.5	hsa-miR-182-3p	0	0
LRRC40	ENST00000370952.3	hsa-miR-182-3p	0	-0,18
ACOT2	ENST00000538782.1	hsa-miR-182-3p	0	-0,01
MFSD2A	ENST00000372811.5	hsa-miR-182-3p	0	-0,1
BTN3A1	ENST00000425234.2	hsa-miR-182-3p	0	-0,03
FAM208A	ENST00000493960.2	hsa-miR-182-3p	0	-0,16
LIPG	ENST00000261292.4	hsa-miR-182-3p	0	-0,11
SAP30	ENST00000296504.3	hsa-miR-182-3p	0	-0,18
SEC23IP	ENST00000369075.3	hsa-miR-182-3p	0	-0,02
RNF103-CHMP3	ENST00000604011.1	hsa-miR-182-3p	0	-0,11
SF1	ENST00000377390.3	hsa-miR-182-3p	0	-0,08
CCR7	ENST00000246657.2	hsa-miR-182-3p	0	-0,01
ZNF277	ENST00000361822.3	hsa-miR-182-3p	0	-0,11
GPR26	ENST00000284674.1	hsa-miR-182-3p	0	-0,01
SPATS2L	ENST00000358677.5	hsa-miR-182-3p	0	-0,07
SSTR2	ENST00000315332.2	hsa-miR-182-3p	0	-0,1
FRRS1	ENST00000287474.5	hsa-miR-182-3p	0	-0,11
ZNF512	ENST00000355467.4	hsa-miR-182-3p	0	0
SHC3	ENST00000375835.4	hsa-miR-182-3p	0	-0,07
CYB5A	ENST00000299438.9	hsa-miR-182-3p	0	-0,34
ALDOA	ENST00000569798.1	hsa-miR-182-3p	0	-0,05
IDS	ENST00000422081.2	hsa-miR-182-3p	0	-0,06
RPS24	ENST00000435275.1	hsa-miR-182-3p	0	-0,13
ZNF562	ENST00000293648.4	hsa-miR-182-3p	0	-0,09
MYO10	ENST00000513610.1	hsa-miR-182-3p	0	-0,04
SMAD6	ENST00000288840.5	hsa-miR-182-3p	0	-0,07
BCL2L13	ENST00000418951.2	hsa-miR-182-3p	0	0
CHRNA4	ENST00000370263.4	hsa-miR-182-3p	0	-0,02
KAT7	ENST00000259021.4	hsa-miR-182-3p	0	-0,04
PPAP2B	ENST00000371250.3	hsa-miR-182-3p	0	-0,08
ARL5A	ENST00000295087.8	hsa-miR-182-3p	0	-0,1
DDRGK1	ENST00000354488.3	hsa-miR-182-3p	0	-0,26
SYAP1	ENST00000380155.3	hsa-miR-182-3p	0	-0,1
ARHGAP29	ENST00000260526.6	hsa-miR-182-3p	0	0
RSL1D1	ENST00000571133.1	hsa-miR-182-3p	0	0
ZNF141	ENST00000240499.7	hsa-miR-182-3p	0	-0,09
CRISPLD2	ENST00000262424.5	hsa-miR-182-3p	0	-0,02
EMC3	ENST00000245046.2	hsa-miR-182-3p	0	-0,1
SFMBT1	ENST00000394752.3	hsa-miR-182-3p	0	-0,06
WDR75	ENST00000314761.4	hsa-miR-182-3p	0	-0,17
SOCS5	ENST00000306503.5	hsa-miR-182-3p	0	-0,06
PLCL1	ENST00000428675.1	hsa-miR-182-3p	0	-0,02
TCF3	ENST00000262965.5	hsa-miR-182-3p	0	-0,01
CARD16	ENST00000375704.3	hsa-miR-182-3p	0	-0,59
CCNB1	ENST00000256442.5	hsa-miR-182-3p	0	-0,2
WT1	ENST00000379079.2	hsa-miR-182-3p	0	-0,11
CD3D	ENST00000300692.4	hsa-miR-182-3p	0	-0,03
ANGPTL3	ENST00000371129.3	hsa-miR-182-3p	0	-0,05
MAG	ENST00000392213.3	hsa-miR-182-3p	0	-0,01
PHC1	ENST00000433083.2	hsa-miR-182-3p	0	0
CACNA1B	ENST00000277551.2	hsa-miR-182-3p	0	-0,01

EHD3	ENST00000322054.5	hsa-miR-182-3p	0	-0,01
THBS1	ENST00000260356.5	hsa-miR-182-3p	0	0
ZNF713	ENST00000429591.2	hsa-miR-182-3p	0	0
PON1	ENST00000222381.3	hsa-miR-182-3p	0	-0,17
LIMK2	ENST00000331728.4	hsa-miR-182-3p	0	-0,02
XBP1	ENST00000344347.5	hsa-miR-182-3p	0	-0,12
ALS2CR11	ENST00000439802.1	hsa-miR-182-3p	0	-0,54
CDC73	ENST00000367435.3	hsa-miR-182-3p	0	-0,03
UBC	ENST00000536769.1	hsa-miR-182-3p	0	-0,05
CEP85	ENST00000252992.4	hsa-miR-182-3p	0	-0,18
SEMA4F	ENST00000357877.2	hsa-miR-182-3p	0	-0,14
DNAH17	ENST00000585328.1	hsa-miR-182-3p	0	-0,01
PLA2G12A	ENST00000243501.5	hsa-miR-182-3p	0	-0,03
CARD11	ENST00000396946.4	hsa-miR-182-3p	0	-0,05
ESR2	ENST00000557772.1	hsa-miR-182-3p	0	0
CCNO	ENST00000282572.4	hsa-miR-182-3p	0	-0,19
NDNF	ENST00000379692.4	hsa-miR-182-3p	0	-0,21
CACUL1	ENST00000369151.3	hsa-miR-182-3p	0	-0,16
SHISA4	ENST00000362011.6	hsa-miR-182-3p	0	-0,16
GPR183	ENST00000376414.4	hsa-miR-182-3p	0	-0,15
PRDX2	ENST00000301522.2	hsa-miR-182-3p	0	-0,13
ZFAND5	ENST00000237937.3	hsa-miR-182-3p	0	-0,16
SERPINC1	ENST00000367698.3	hsa-miR-182-3p	0	-0,17
SLC2A9	ENST00000264784.3	hsa-miR-182-3p	0	0
ELK1	ENST00000247161.3	hsa-miR-182-3p	0	-0,03
FAM53A	ENST00000489363.1	hsa-miR-182-3p	0	-0,06
SYTL4	ENST00000372989.1	hsa-miR-182-3p	0	-0,15
ABHD15	ENST00000307201.4	hsa-miR-182-3p	0	-0,12
STRADA	ENST00000375840.4	hsa-miR-182-3p	0	-0,1
IGF2R	ENST00000356956.1	hsa-miR-182-3p	0	-0,01
SF3B1	ENST00000335508.6	hsa-miR-182-3p	0	-0,02
SMTNL2	ENST00000338859.4	hsa-miR-182-3p	0	-0,01
CHMP3	ENST00000263856.4	hsa-miR-182-3p	0	-0,12
VWA1	ENST00000338660.5	hsa-miR-182-3p	0	-0,14
UBE2D3	ENST00000453744.2	hsa-miR-182-3p	0	-0,26
SLC16A3	ENST00000581287.1	hsa-miR-182-3p	0	-0,01
