# Supplementary figures and images for: MiR‐182‐3p targets TRF2 and impairs tumor growth of triple‐negative breast cancer
Source: EMBO Mol Med. 2022 Nov 25;15(1):e16033. doi: 10.15252/emmm.202216033 (PMC9832842; doi:10.15252/emmm.202216033)

Source Data Figure 1F

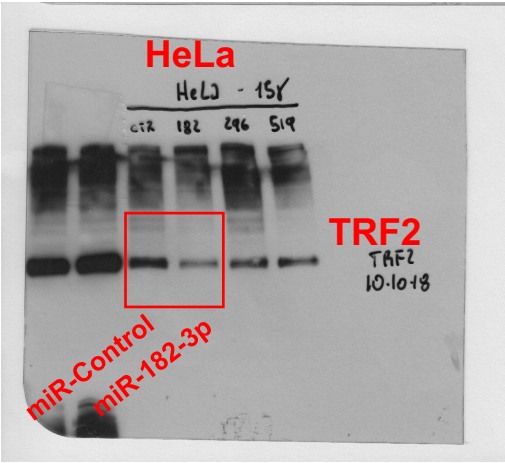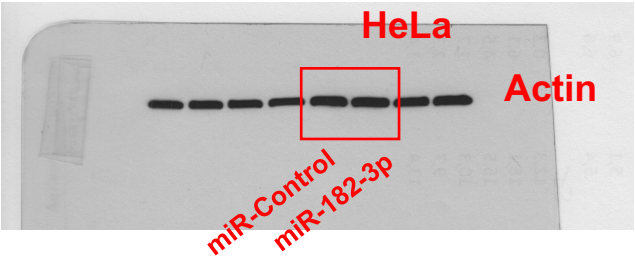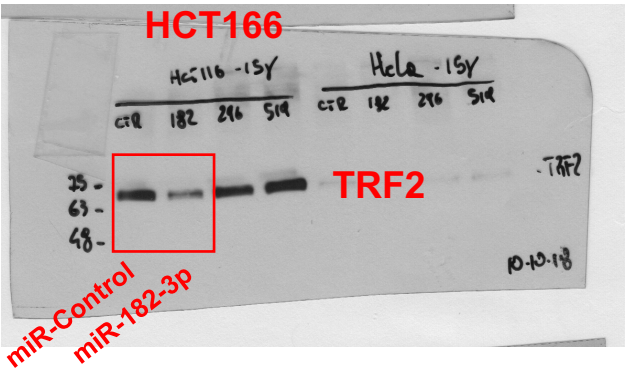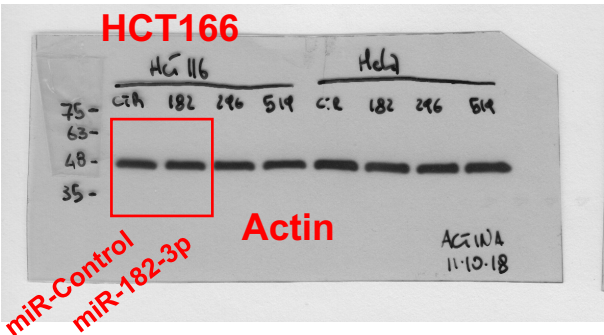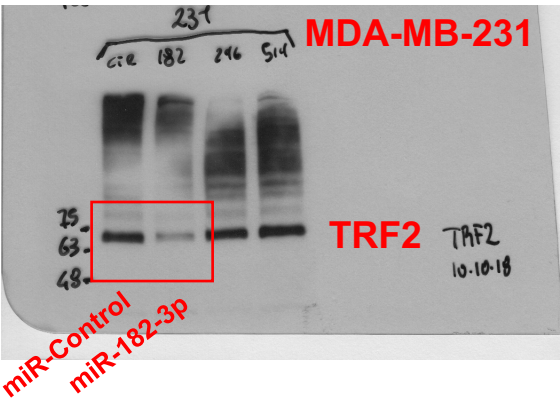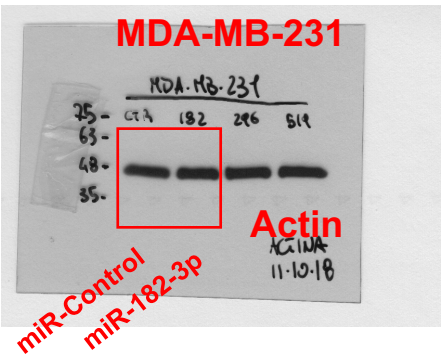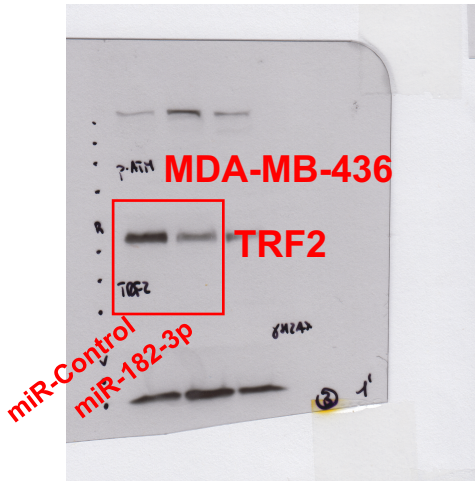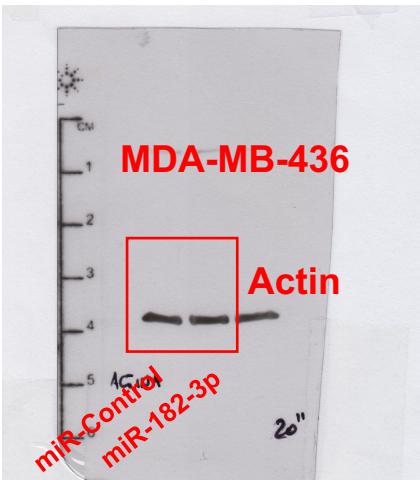

Source Data Figure 1G

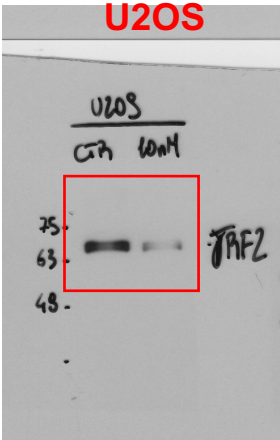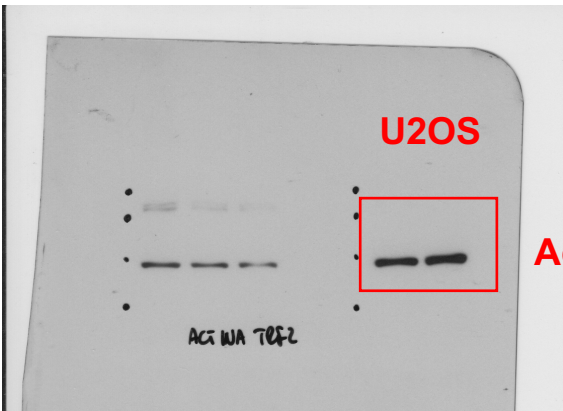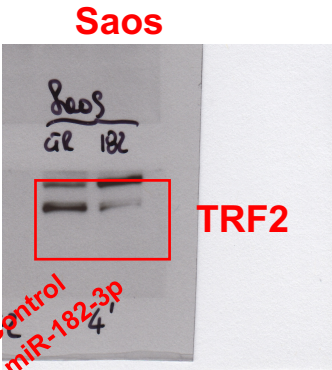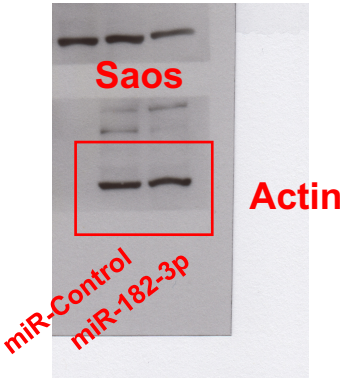

Supplement: Supplementary file 7 — Source Data for Figure 1 [file EMMM-15-e16033-s013.zip › EMM-2022-16033-V5-Figure_1_Source_Data-sd.pdf]

Source Data Figure 3C,D

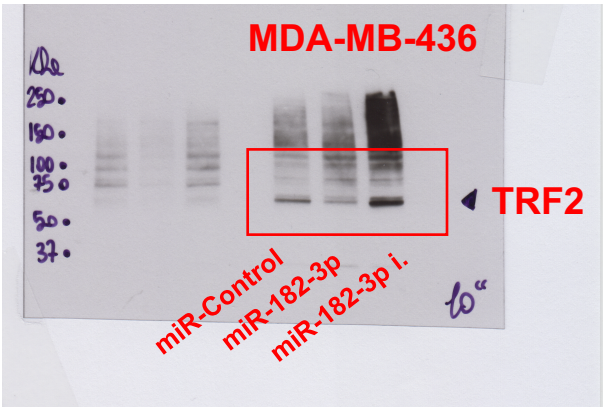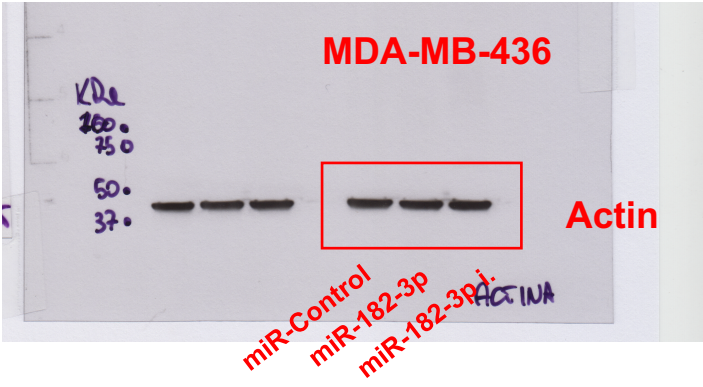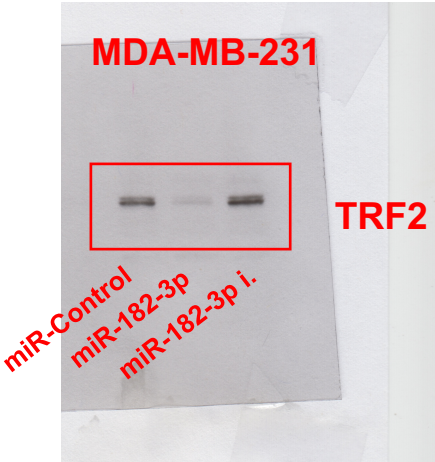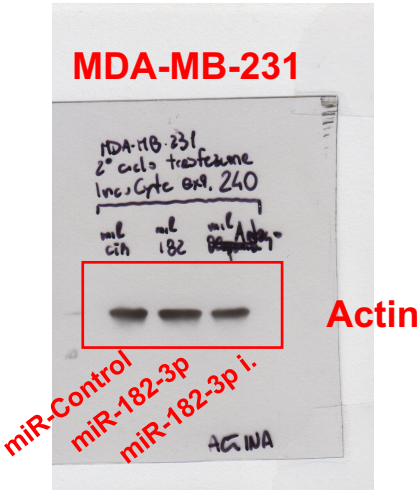

Supplement: Supplementary file 9 — Source Data for Figure 3 [file EMMM-15-e16033-s003.zip › EMM-2022-16033-V5-Figure_3_Source_Data-sd.pdf]

Source Data Figure 4A

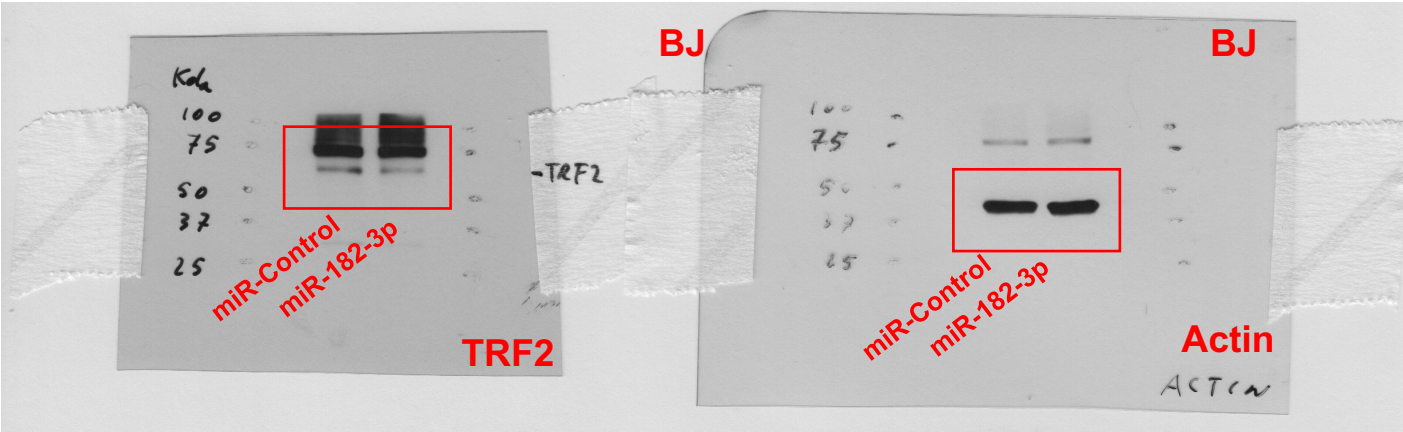

Supplement: Supplementary file 10 — Source Data for Figure 4 [file EMMM-15-e16033-s002.zip › EMM-2022-16033-V5-Figure_4_Source_Data-sd.pdf]

Source Data Figure 6

PDTC#1

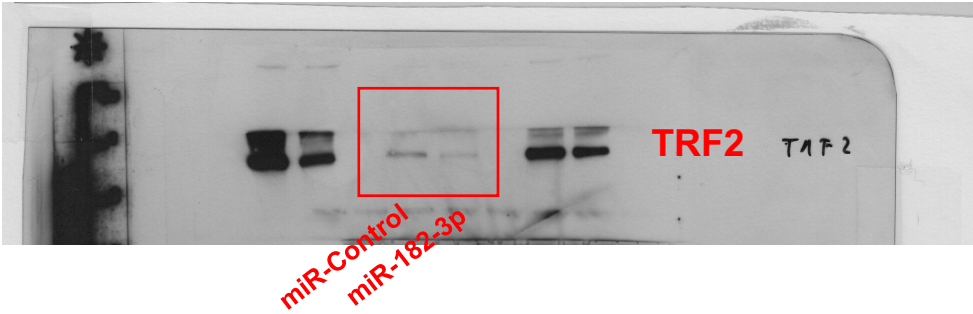

PDTC#1

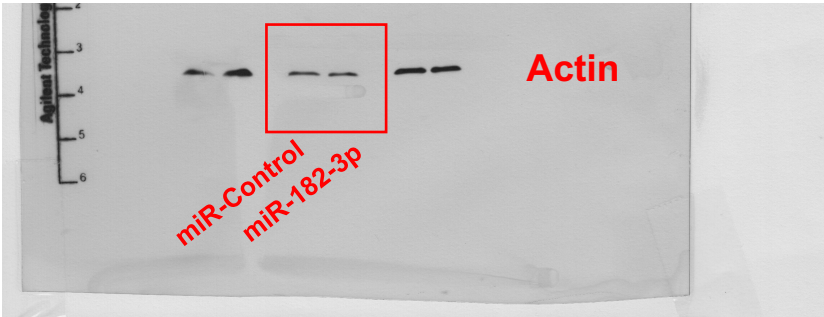

PDTC#2

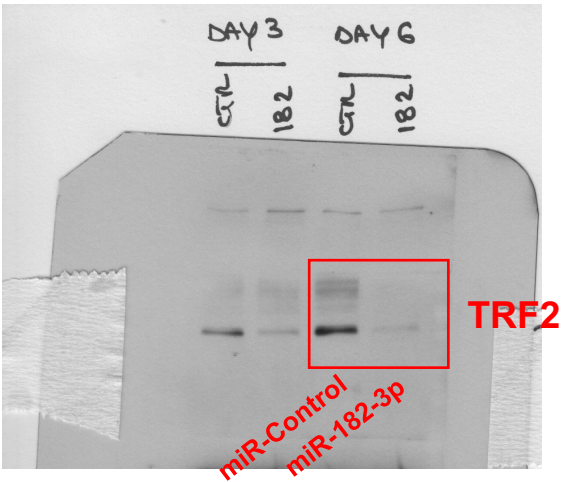

PDTC#2

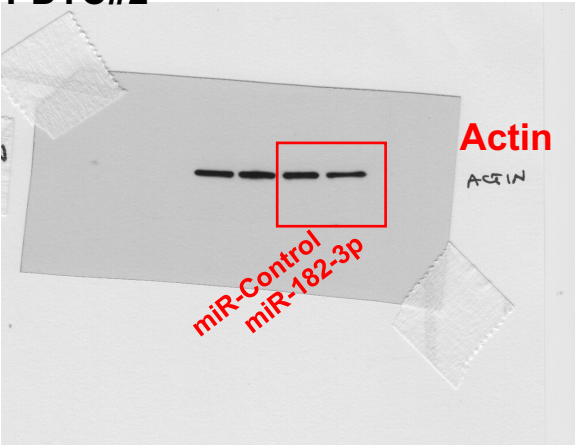

Supplement: Supplementary file 12 — Source Data for Figure 6 [file EMMM-15-e16033-s001.zip › EMM-2022-16033-V5-Figure_6_Source_Data-sd.pdf]
