# Supplementary material for: MiR‐182‐3p targets TRF2 and impairs tumor growth of triple‐negative breast cancer
Source: EMBO Mol Med. 2022 Nov 25;15(1):e16033. doi: 10.15252/emmm.202216033 (PMC9832842; doi:10.15252/emmm.202216033)
Supplement: Supplementary file 14 [file EMMM-15-e16033-s007.pdf]

EMBO Molecular Medicine

MiR-182-3p targets TRF2 and impairs tumor growth of triple negative breast cancer

Roberto Dinami, Luca Pompili, Eleonora Petti, Manuela Porru, Carmen D'Angelo, Serena Di Vito, Angela Rizzo, Virginia Campani, Giuseppe De Rosa, Alejandra Bruna, Violeta Serra, Miguel Mano, Mauro Giacca, Carlo Leonetti, Gennaro Ciliberto, Madalena Tarsounas, Antonella Stoppacciaro, Stefan Schoeftner, and Annamaria Biroccio
DOI: 10.15252/emmm.202216033

Corresponding author: Annamaria Biroccio (annamaria.biroccio@ifo.it)

Review Timeline:

Submission Date:	18th Mar 22
Editorial Decision:	12th Apr 22
Editor Correspondence:	13th Apr 22
Revision Received:	11th Jul 22
Editorial Decision:	10th Aug 22
Revision Received:	29th Sep 22
Editorial Decision:	14th Oct 22
Revision Received:	25th Oct 22
Editorial Decision:	27th Oct 22
Revision Received:	29th Oct 22
Accepted:	5th Nov 22

Editor: Lise Roth

Transaction Report:

(Note: With the exception of the correction of typographical or spelling errors that could be a source of ambiguity, letters and reports are not edited. Depending on transfer agreements, referee reports obtained elsewhere may or may not be included in this compilation. Referee reports are anonymous unless the Referee chooses to sign their reports.)

12th Apr 2022

Dear Dr. Biroccio,

Thank you for the submission of your manuscript to EMBO Molecular Medicine. We have now received feedback from two of the three reviewers who agreed to evaluate your manuscript. Given that referee #2 has not yet returned his/her report, and that both referees #1 and #3 provide similar recommendations, we prefer to make a decision now in order to avoid further delay in the process. Should referee #2 provide a report, we will send it to you, with the understanding that we will not ask you extensive experiments in addition to the ones required in the enclosed reports from referee #1 and #3.

Addressing the reviewers' concerns in full will be necessary for further considering the manuscript in our journal, and acceptance of the manuscript will entail a second round of review. EMBO Molecular Medicine encourages a single round of revision only and therefore, acceptance or rejection of the manuscript will depend on the completeness of your responses included in the next, final version of the manuscript. For this reason, and to save you from any frustrations in the end, I would strongly advise against returning an incomplete revision.

Revised manuscripts should be submitted within three months of a request for revision; they will otherwise be treated as new submissions, except under exceptional circumstances in which a short extension is obtained from the editor.

When submitting your revised manuscript, please carefully review the instructions that follow below. We perform an initial quality control of all revised manuscripts before re-review; failure to include requested items will delay the evaluation of your revision.

We require:

1) A .docx formatted version of the manuscript text (including legends for main figures, EV figures and tables). Please make sure that the changes are highlighted to be clearly visible.

2) Individual production quality figure files as .eps, .tif, .jpg (one file per figure). For guidance, download the 'Figure Guide PDF' (https://www.embopress.org/page/journal/17574684/authorguide#figureformat).

3) A .docx formatted letter INCLUDING the reviewers' reports and your detailed point-by-point responses to their comments. As part of the EMBO Press transparent editorial process, the point-by-point response is part of the Review Process File (RPF), which will be published alongside your paper.

4) A complete author checklist, which you can download from our author guidelines (https://www.embopress.org/page/journal/17574684/authorguide#submissionofrevisions). Please insert information in the checklist that is also reflected in the manuscript. The completed author checklist will also be part of the RPF.

5) Please note that all corresponding authors are required to supply an ORCID ID for their name upon submission of a revised manuscript.

6) It is mandatory to include a 'Data Availability' section after the Materials and Methods. Before submitting your revision, primary datasets produced in this study need to be deposited in an appropriate public database, and the accession numbers and database listed under 'Data Availability'. Please remember to provide a reviewer password if the datasets are not yet public (see https://www.embopress.org/page/journal/17574684/authorguide#dataavailability).
In case you have no data that requires deposition in a public database, please state so in this section. Note that the Data Availability Section is restricted to new primary data that are part of this study.

7) For data quantification: please specify the name of the statistical test used to generate error bars and P values, the number (n) of independent experiments (specify technical or biological replicates) underlying each data point and the test used to calculate p-values in each figure legend. The figure legends should contain a basic description of n, P and the test applied. Graphs must include a description of the bars and the error bars (s.d., s.e.m.).

8) We would also encourage you to include the source data for figure panels that show essential data. Numerical data should be provided as individual .xls or .csv files (including a tab describing the data). For blots or microscopy, uncropped images should be submitted (using a zip archive if multiple images need to be supplied for one panel). Additional information on source data and instruction on how to label the files are available at
.

9) Our journal encourages inclusion of *data citations in the reference list* to directly cite datasets that were re-used and obtained from public databases. Data citations in the article text are distinct from normal bibliographical citations and should directly link to the database records from which the data can be accessed. In the main text, data citations are formatted as

follows: "Data ref: Smith et al, 2001" or "Data ref: NCBI Sequence Read Archive PRJNA342805, 2017". In the Reference list, data citations must be labeled with "[DATASET]". A data reference must provide the database name, accession number/identifiers and a resolvable link to the landing page from which the data can be accessed at the end of the reference. Further instructions are available at .

10) We replaced Supplementary Information with Expanded View (EV) Figures and Tables that are collapsible/expandable online. A maximum of 5 EV Figures can be typeset. EV Figures should be cited as 'Figure EV1, Figure EV2" etc... in the text and their respective legends should be included in the main text after the legends of regular figures.
- For the figures that you do NOT wish to display as Expanded View figures, they should be bundled together with their legends in a single PDF file called *Appendix*, which should start with a short Table of Content. Appendix figures should be referred to in the main text as: "Appendix Figure S1, Appendix Figure S2" etc.
- Additional Tables/Datasets should be labeled and referred to as Table EV1, Dataset EV1, etc. Legends have to be provided in a separate tab in case of .xls files. Alternatively, the legend can be supplied as a separate text file (README) and zipped together with the Table/Dataset file.
See detailed instructions here:
.

11) The paper explained: EMBO Molecular Medicine articles are accompanied by a summary of the articles to emphasize the major findings in the paper and their medical implications for the non-specialist reader. Please provide a draft summary of your article highlighting
- the medical issue you are addressing,
- the results obtained and
- their clinical impact.
This may be edited to ensure that readers understand the significance and context of the research. Please refer to any of our published articles for an example.

12) For more information: There is space at the end of each article to list relevant web links for further consultation by our readers. Could you identify some relevant ones and provide such information as well? Some examples are patient associations, relevant databases, OMIM/proteins/genes links, author's websites, etc...

13) Author contributions: the contribution of every author must be detailed in a separate section (before the acknowledgments).

14) Conflict of interest: We updated our journal's competing interests policy in January 2022 and request authors to consider both actual and perceived competing interests. Please review the policy https://www.embopress.org/competing-interests and update your competing interests if necessary.

15) Every published paper now includes a 'Synopsis' to further enhance discoverability. Synopses are displayed on the journal webpage and are freely accessible to all readers. They include a short stand first (maximum of 300 characters, including space) as well as 2-5 one-sentences bullet points that summarizes the paper. Please write the bullet points to summarize the key NEW findings. They should be designed to be complementary to the abstract - i.e. not repeat the same text. We encourage inclusion of key acronyms and quantitative information (maximum of 30 words / bullet point). Please use the passive voice. Please attach these in a separate file or send them by email, we will incorporate them accordingly.

Please also suggest a striking image or visual abstract to illustrate your article as a PNG file 550 px wide x 300-600 px high.

16) As part of the EMBO Publications transparent editorial process initiative (see our Editorial at http://embomolmed.embopress.org/content/2/9/329), EMBO Molecular Medicine will publish online a Review Process File (RPF) to accompany accepted manuscripts.
In the event of acceptance, this file will be published in conjunction with your paper and will include the anonymous referee reports, your point-by-point response and all pertinent correspondence relating to the manuscript. Let us know whether you agree with the publication of the RPF and as here, if you want to remove or not any figures from it prior to publication.
Please note that the Authors checklist will be published at the end of the RPF.

EMBO Molecular Medicine has a "scooping protection" policy, whereby similar findings that are published by others during review or revision are not a criterion for rejection. Should you decide to submit a revised version, I do ask that you get in touch after three months if you have not completed it, to update us on the status.

I look forward to receiving your revised manuscript.

Yours sincerely,

Lise Roth

Lise Roth, PhD
Editor
EMBO Molecular Medicine

***** Reviewer's comments *****

Referee #1 (Comments on Novelty/Model System for Author):

Although TRF2 overexpression is detected in many human cancers, it is not well understood how TRF2 overexpression contributes to tumorigenesis or tumor growth. TRF2 plays a key role in telomere capping in all cell types. Thus, disruption of TRF2 function may lead to cellular senescence or apoptosis in non-cancerous cells.

Referee #1 (Remarks for Author):

Telomere repeat binding-factor 2 (TRF2) is overexpressed in various human cancers. Whether TRF2 overexpression plays any role in tumorigenesis and tumor growth is intriguing and worth exploring. This study by Dinami and co-authors presented their efforts in identification and characterization of an anticancer miRNA-based approach to target TRF2 in human cancer cell lines and in tumor mouse models. They have demonstrated that miR-182-3p is a specific and efficient post-transcriptional regulator of TRF2. Ectopic expression of miR-182-3p drastically reduced TRF2 protein levels, induced DNA damage response (DDR) at telomeric and pericentromeric sites, impaired cell proliferation, and activated cell apoptosis in a panel of cancer cell lines in culture. In addition, lipid nanoparticle-containing miR-182-3p was able to cross the blood brain barrier in mice and impaired tumor growth in aggressive cancer mouse models, including patient-derived tumor xenografts. This study is of great interests in cancer therapy and telomere biology field. The manuscript was well written, and the experiments were well designed and executed. But some data presentation and interpretation need further clarification.

Major points:

1. The authors showed that ectopic expression of miR-182-3p drastically reduced TRF2 protein levels. It is known that TRF2 levels affects the stability of its interacting protein, RAP1 (Sfeir et al, Science 2010). Although miR-182-3p appears to specifically target TRF2, it may indirectly affect RAP1 protein levels, which needs to be addressed.
2. Given that ectopic expression of TRF2 drastically reduced miR-182-3p-induced DDR, I wonder whether TRF2 expression levels in individual tumors affect the effectiveness of miR-182-3p intervention?
3. TRF2 plays a key role in telomere capping, and its deficiency results in telomere dysfunction-induced DDR, leading to cellular senescence or apoptosis. The authors claimed that treatment with LNPs-miR-182-3p does not cause a systematic toxicity in organs, such as brain, liver, and kidney. Although post-mitotic cells, such as neurons tolerate telomere dysfunction-induced DDR (Li et al, Genes Dev. 31(7): 639-47, 2017), telomere dysfunction-induced DDR could lead to cell death or cellular senescence in proliferative cell types. Thus, it is crucial to investigate if lipid nanoparticle-containing miR-182-3p has any adverse effect on proliferative organs, such as bone marrow, skin, and intestine.

Minor points:

Figure S2: The effect of miR-182-3p on DDR does not appear to be strong, judged from the WB images. Please provide the quantification of pATM and gamma-H2AX protein levels in S2A, S2B, S2G, and S2H.

Referee #3 (Comments on Novelty/Model System for Author):

Mouse is an appropriate model system to study efficacy of cancer therapies

Referee #3 (Remarks for Author):

Inhibition of telomerase and/or inducing telomere dysfunction remains a potentially powerful anti-cancer therapy. Indeed, strategies to target shelterin components such as TRF1 have shown promising results in in suppressing cancer growth. In the study by Dinami et al. the authors conduct a screen to identify microRNAs that can suppress the expression of TRF2, another component of shelterin and a factor that is critical for maintaining telomere function. They identify miR-182-3p as an efficient post-transcriptional regulator of TRF2 levels. Overexpression of miR-182-3p caused telomere dysfunction, double stranded DNA breaks at pericentromeric sites and consequently apoptosis in a number of human cancer cell lines. Delivery of miR-182-3p in liquid nanoparticles (LNPs) also caused telomere dysfunction in cancer cells transplanted into animals leading to impaired tumor

growth, in addition to preventing growth of Patient-Derived Tumor Xenografts. This is an interesting and potentially important study as it reveals a potentially new anti-cancer therapy. However, a number of controls and experiments are missing that would make this study stronger. Major and minor comments are listed below.

Major comments:
1) It is surprising that miR-179 182-3p does not affect viability of normal cells as animal experiments suggest. One would expect that TRF2 downregulation leads to cellular senescence or apoptosis in normal human somatic cells, just like TRF2 knockdown does, as demonstrated in previous studies. Data should be shown that reveal the effects of miR-182-3p on normal somatic human cells (cell proliferation curves, SAbGal, TUNEL, gH2AX, etc) and on normal cells in mouse tissue (see below).

2) Fig 1H shows that miR-179 182-3p decreases telomere signal intensities, while a miR-179 182-3p inhibitor enhances TRF2 signal intensity in an ALT cell line, U2-OS cells. It is unclear why is this relevant for this study that primarily uses MDA-MB-231 and MDA-MB-436 cells? Along these lines, based on Figure 2B and F, telomere signal intensities do not appear to be affected by miR-179 182-3p in MDA 231 cancer cells (and in HeLa cells shown in Fig S2D), which is unlike U2OS cells (Fig 1H). Why is this? Telomere signal intensity reduction, following miR-179 182-3p treatment, and enhancement, following miR-179 182-3p inhibitor treatment, should be shown for at least one of these telomerase expressing cancer cells in Figure 1H.

3) If data on U2OS cells will remain in the study, more data on these cells should be shown. For example, does miR-179 182-3p also cause TIF, PIF, and apoptosis in ALT cells, such as U2OS cells? These data should be shown in Figure 2, or in the supplemental Figures, as it would be highly relevant when considering treating ALT cancers

4) Data should be shown that would reveal why miR-179 182-3p causes apoptotic cell death. For example, is the miR-179 182-3p-induced apoptotic cell death response due to telomeric bfb cycles? Evidence for or against this this should be provided.

5) More data should be shown on the absence/presence of side effects of administering LNP containing miR-179 182-3p on non-tumor tissue in animal experiments. Markers of senescence (SA-bGal, p16, p21, gH2AX, ...) or apoptosis (TUNEL) should be analyzed, in addition to morphological changes in normal tissue. Also, levels of TRF2 should be compared in normal tissue, similar to what is shown for the tumors.

Minor comments:
- It should be discussed whether TRF2 inhibition also affects non-telomeric functions of TRF2. This seems unlikely given that the primary response to miR-182-3p is apoptosis induced by telomere dysfunction.

- The role of TRF2 at pericentromeric sites, and how it suppresses a DDR there, should be mentioned.

- It should be discussed why BRCA1 compromised cancers are more sensitive to TRF2 inhibition.

Dear Dr. Biroccio,

We have now received the report from referee #2 (attached). As you will see, this referee's main concern is about the potential systemic toxicity of the proposed approach, which was already mentioned by the other referees.
In a revised version of your manuscript, we would therefore like you to address this point, as well as the other points from referee #2.

Looking forward to receiving your revised manuscript,

With kind regards,

Lise Roth

Dinami et al. identifies a novel and potentially interesting strategy how to suppress the growth of tumor cells in vitro and in vivo using miR-182-3p overexpression. They nicely document that this miR directly binds to the 3'UTR of TRF2 and inhibits its expression in vitro and in vivo. They make a proof of concept that it is possible to shrink the tumor size in mouse xenographs by administrating miR-182-3p intravenously. Authors claim that this treatment does not cause a systemic toxicity.

My main concern is that it has been already shown that TRF2 inhibition triggers apoptosis and reduces tumorigenicity of human melanoma cells (Biroccio, 2006) and therefore the novelty of this study is limited to the discovery that TRF2 levels can also be modulated using a miR, an approach that may be of a potential therapeutic interest. While this study provides promising in vivo data about the feasibility of such an approach it fails to addresses properly the systemic toxicity. Authors shall provide data for proliferation, apoptosis, DNA damage and TRF2 levels for all main organs (currently only one unspecified organ was evaluated). Another main concern, which authors themselves address briefly in discussion, is that this miR has other known targets that regulate tumor progression including a transcription factor EBF. With current evidence it is impossible to evaluate to what extend the proposed therapy acts through TRF2. According to the authors this question is beyond the scope, however it can be experimentally addresed by evaluating the growth of cancer cells mutated in 3'UTR-TRF2 in xenografts recieving miR-182-3p injections.

General remarks:

Scale bars for histograms in figures 4 and 5 are cut. Please avoid this practice and rather choose the log scale. It is particularly disturbing for Fig4B where the graph is cut twice, once inbetween data points and by consequence the error bar is gone as well.

Statistical analysis needs to be provided for all data in a particular graph and for all graphs. Currently there is no statistics applied to data with visually smaller differences.

Legends need to be more concise (lots of repetition within each legend). When several images are shown under one Image letter they needs to be unambigously annotated (I imaging legends state conditions from left to right).

For immunofluorescence images, please provide representative images with fleshes pointing to the colocalization events, consistently accompanied with enlarged sections (sections of nuclei rather than one chosen focus) and scale bars. For quantifications I strongly recommend showing the actual number of colocalization events for each nucleus or at least the average number of colocalisation events for each experimental condition. Current quantifications are based on the observer defined cutoff values for identifying cells with DNA damage which are poorly justified and are not the norm in the field today.

Point by point remarks:

51 - Please specify in the abstract that the high-throughput screen was done with a set of candidate miRs. As written it suggests that all known miRs were screened.

74 - Human telomeres are composed BY à replace with OF

164 - TRF1 displays high sequence homology with TRF2 à sequence homology has little importance when it comes to targeting by miRs. Either the target sequence is there or it is not. The paragraph 162-170 has to be rephrased and I strongly recommend to shorten it.

175 - in Fig S1C only mRNA expression data for Telomerase+ cell lines are shown. Please do not make general conclusions. Either rephrase to specify that TRF2 mRNA levels are not affected in telomerase-positive cell lines or provide data for ALT.

176 - correcte syntaxe : impacts something or an impact on something

177 - The conclusion is beyond data provided. Only TRF2 staining is shown therefore no conclusions about TRF2 abundance at telomeres can be made. This figure needs to be replaced by a combined anti-TRF2 telomeric FISH approach.

198 - reproducing the effect induced by siTRF2. Reference is missing. If Mendez-Bermudez is referred to, please note that data in this particual paper is analysed and presented differently.

229 - authors suggest that miR-182-3p induces a drastic change of cell morphology without specifying what this change might be. Indeed in S31-B figure we can observe that cells are rounded up and at least some look apoptotic, a phenotype that authors address elsewhere in the manuscript. Precisely because of this change in morphology, quantifying proliferation using a confluency approach may be problematic. Growth curves with population doublings need to be shown.

231-240: The paragraph about the cell cycle analysis is written in an ambiguous way without real conclusions. The opening sentence claims that they wanted to evaluate whether there is an inhibition of cell proliferation and/or apoptosis. Authors suggest that there is a reduced percentage of cells in the proliferative compartment but this phrase is misleading since it may be understood that there is a problem with replication. Authors do not mention that there is a reduction in G1 as well, therefore the only conclusion is that cells are dying. The paragraph should be shortened to one sentence.

Figure 1B: Justify why only 6 out of 7 nucleotides recognized by miR in 3'UTR of TRF2 were deleted.

Figure 1C-E. Specify what miR-Control corresponds to. Provide statistics for all images. Is the difference in E statistically significant?

Figure 1H: Image needs to be replaced with an immune-FISH. TRF2 signals colocalizing with telomeres need to be quantified and such quantification needs to be limited to G1 cells since telomeric content and therefore TRF2 signal at telomeres varies with the cell cycle. Imaging only 60 nuclei is not sufficient if cell phases are not discriminated.

Figure 2A:

My recommendations are in the general remarks.

From representative images the suppression of TIFs by TRF2 overexpression is not evident. No enlarged sections shown.

Figure 3:

Please provide data for population doublings rather than confluence considering that you consider there is an effect or mir-182-3p on cell morphology. I recommend putting all FACS scatter plots into Supplemental data. Rather than labeling +/-Dox label with the gene that is modulated.

Figure 4:

Consider putting histograms on log scale rather than cutting the axe.

Figure 5:

« Area of each PDTCs was measures » - not clear what this refers to, spheres? If so, you need to perform the proper assay to measure sphere size.

Figure S3:

Profiles for BrdU FACS are represented in an unconventional. It looks like most G1 cells were excluded and some G1 may be excluded as well.

Figure S4C - legend does not specify for which organ the analysis was done. All organs sholud have been analyzed.

REPLY TO REFEREE' COMMENTS

Dinami et al. 2022

Referee #1 (Comments on Novelty/Model System for Author):

Although TRF2 overexpression is detected in many human cancers, it is not well understood how TRF2 overexpression contributes to tumorigenesis or tumor growth. TRF2 plays a key role in telomere capping in all cell types. Thus, disruption of TRF2 function may lead to cellular senescence or apoptosis in non-cancerous cells.

Referee #1 (Remarks for Author):

Telomere repeat binding-factor 2 (TRF2) is overexpressed in various human cancers. Whether TRF2 overexpression plays any role in tumorigenesis and tumor growth is intriguing and worth exploring. This study by Dinami and co-authors presented their efforts in identification and characterization of an anticancer miRNA-based approach to target TRF2 in human cancer cell lines and in tumor mouse models. They have demonstrated that miR-182-3p is a specific and efficient post-transcriptional regulator of TRF2. Ectopic expression of miR-182-3p drastically reduced TRF2 protein levels, induced DNA damage response (DDR) at telomeric and pericentromeric sites, impaired cell proliferation, and activated cell apoptosis in a panel of cancer cell lines in culture. In addition, lipid nanoparticle-containing miR-182-3p was able to cross the blood brain barrier in mice and impaired tumor growth in aggressive cancer mouse models, including patient-derived tumor xenografts. This study is of great interests in cancer therapy and telomere biology field. The manuscript was well written, and the experiments were well designed and executed. But some data presentation and interpretation need further clarification.

Answer to Referee #1:

We thank the Referee for considering our manuscript of great interest and for providing useful suggestions to improve our work.

Major points:

1. The authors showed that ectopic expression of miR-182-3p drastically reduced TRF2 protein levels. It is known that TRF2 levels affects the stability of its interacting protein, RAP1 (Sfeir et al, Science 2010). Although miR-182-3p appears to specifically target TRF2, it may indirectly affect RAP1 protein levels, which needs to be addressed.
As requested by the Referee, we analyzed the expression levels of RAP1 by western blot after transfection with miR-182-3p. The result is reported in Figure EV1B of the revised version of the manuscript.

2. Given that ectopic expression of TRF2 drastically reduced miR-182-3p-induced DDR, I wonder whether TRF2 expression levels in individual tumors affect the effectiveness of miR-182-3p intervention?
We thank the Referee for this interesting observation.
First of all, it is important to specify that the construct used throughout the paper to over-express TRF2 contains the TRF2 coding sequence lacking its 3'-UTR, thus resulting un-targetable by miR-182-3p. Therefore, the results obtained by performing ectopic expression of TRF2 are not informative about the role of TRF2 expression levels in the response to the miR-182-3p. They, on the contrary, are useful to demonstrate that the effects of the miR-182-3p are specific due to TRF2 targeting. Indeed, consistently with the DDR data, TRF2 over-expression (lacking the 3'UTR) rescues the anti-proliferative and apoptotic effects mediated by miR-182-3p. These results have been included in the new version of the manuscript (Fig 3I,J).
Moreover, to try to address the request of referee about the effectiveness of miR-182-3p on individual tumors with different endogenous expression levels of TRF2, a new experiment transfecting the miRNA in different cancer cells lines was performed. Preliminary results (given to the referee only) showed that cell growth inhibition by miR-182-3p appears to be not directly correlated to TRF2 expression levels in the

cancer cell lines analyzed, suggesting that even tumors with high levels of TRF2 could take advantage to this therapeutic strategy. Indeed, miR-182-3p is very effective also in HCT116 cells which display high levels of TRF2.

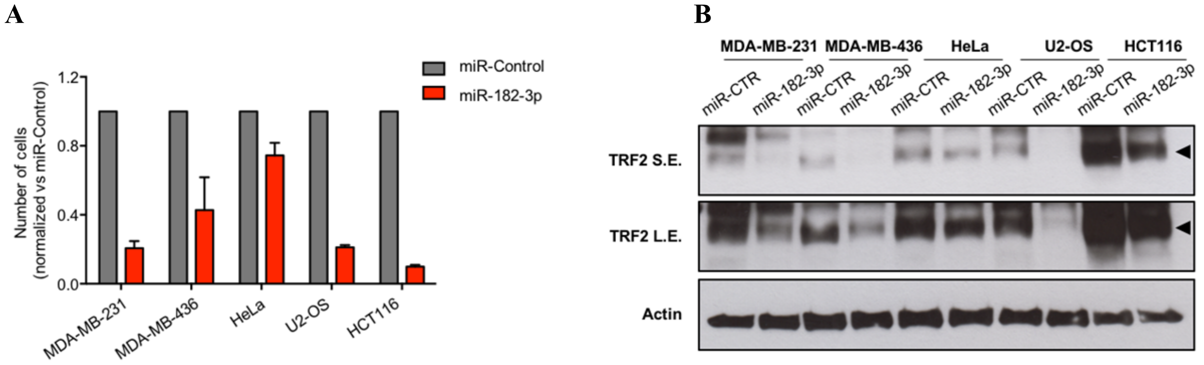

Figure for Referee: Different cancer cell lines were subjected to two cycles of transfection with miR-Control or miR-182-3p. Number of cells at the end of the experiment is shown in (A). Western blotting analysis of TRF2 expression is shown in (B).

3. TRF2 plays a key role in telomere capping, and its deficiency results in telomere dysfunction-induced DDR, leading to cellular senescence or apoptosis. The authors claimed that treatment with LNPs-miR-182-3p does not cause a systematic toxicity in organs, such as brain, liver, and kidney. Although post-mitotic cells, such as neurons tolerate telomere dysfunction-induced DDR (Li et al, Genes Dev. 31(7): 639-47, 2017), telomere dysfunction-induced DDR could lead to cell death or cellular senescence in proliferative cell types. Thus, it is crucial to investigate if lipid nanoparticle-containing miR-182-3p has any adverse effect on proliferative organs, such as bone marrow, skin, and intestine.

The issue of the Referee was specifically addressed by performing H&E analysis in proliferative (bone marrow, skin, and intestine) and non-proliferative organs. Moreover, general markers of DNA damage (γH2AX), apoptosis (Caspase 3 activated), senescence (p21/p16/β-Gal) and proliferation (Ki67) were analyzed in the proliferative organs. The results were reported in three completely new Figures: Fig 6, Fig EV5 and Appendix Fig S5.

Minor points:

Figure S2: The effect of miR-182-3p on DDR does not appear to be strong, judged from the WB images. Please provide the quantification of pATM and gamma-H2AX protein levels in S2A, S2B, S2G, and S2H.

In the revised version of the manuscript we provided the quantification for pATM, γH2AX and TRF2 protein levels (Fig EV2A,C and EV2G,H).

Referee#2

Dinami et al. identifies a novel and potentially interesting strategy how to suppress the growth of tumor cells in vitro and in vivo using miR-182-3p overexpression. They nicely document that this miR directly binds to the 3'UTR of TRF2 and inhibits its expression in vitro and in vivo. They make a proof of concept that it is possible to shrink the tumor size in mouse xenographts by administrating miR-182-3p intravenously. Authors claim that this treatment does not cause a systemic toxicity.

My main concern is that it has been already shown that TRF2 inhibition triggers apoptosis and reduces tumorigenicity of human melanoma cells (Biroccio, 2006) and therefore the novelty of this study is limitted to the discovery that TRF2 levels can also be modulated using a miR, an approach that may be of a potential therapeutic interest. While this study provides promising in vivo data about the feasibility of such an approach it fails to addresses properly the systemic toxicity. Authors shall provide data for proliferation, apoptosis, DNA damage and TRF2 levels for all main organs (currently only one unspecified organ was evaluated).

Another main concern, which authors themselves address briefly in discussion, is that this miR has other known targets that regulate tumor progression including a transcription factor EBF. With current evidence it is impossible to evaluate to what extend the proposed therapy acts through TRF2. According to the authors this question is beyond the scope, however it can be experimentally addresed by evaluating the growth of cancer cells mutated in 3'UTR- TRF2 in xenografts recieving miR-182-3p injections.

Answer to Referee #2:

We thank the Referee for considering our study of potential therapeutic interest and for all useful suggestions about data presentation and interpretation to improve the quality of our work.

Systemic toxicity was addressed by a more extensive analysis of proliferative and non-proliferative organs as described in the reply to point by point remarks (see below).

Regarding the second concern about the possibility that miR-182-3p may have other targets that regulate tumor progression, we first tested EBF, the only target already published, in our experimental condition, but we did not observe any effect of miR-182-3p on its expression in HeLa and U2-OS cells (Figure provided for Referee). Moreover, a prediction analysis, identifying other putative targets of miR-182-3p, has been introduced in Table EV2 and discussed in the Discussion Section. Validation of the new predicted targets would require a great experimental effort which will be addressed in a future study. On the other hand, the role of TRF2 as key target of miR-182-3p in cell growth inhibition and apoptosis has been specifically addressed by new experiments showed in Fig. 3I,J.

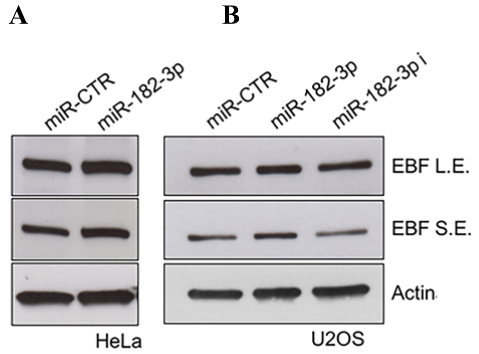

Figure for Referee: Western blotting analysis of EBF expression in Hela (A) and U2-OS (B) upon transfection with the indicated miRNAs

General remarks:

Scale bars for histograms in figures 4 and 5 are cut. Please avoid this practice and rather choose the log scale. It is particularly disturbing for Fig4B where the graph is cut twice, once inbetween data points and by consequence the error bar is gone as well.

As suggested by the Referee, in the revised version of the manuscript, we modified the graph presentation of old Fig 4B,D, Fig 5A,C and Fig S6A with the new Fig. 4C,D, Fig. 5A,B and Fig EV4A using the log scale.

Statistical analysis needs to be provided for all data in a particular graph and for all graphs. Currently there is no statistics applied to data with visually smaller differences.
In the new version of the manuscript, we showed the statistical analysis for all the presented data.

Legends need to be more concise (lots of repetition within each legend). When several images are shown under one Image letter they needs to be unambigously annotated (I imaging legends state conditions from left to right).
As required by Referee, in the revised manuscript we reformulated the legends and, changing the position of some graphs (new Fig. 4C,D, Fig. 5A,B), we indicated the figures from left to right.

For immunofluorescence images, please provide representative images with fleshes pointing to the colocalization events, consistently accompanied with enlarged sections (sections of nuclei rather than one chosen focus) and scale bars. For quantifications I strongly recommend showing the actual number of colocalization events for each nucleus or at least the average number of colocalisation events for each experimental condition. Current quantifications are based on the observer defined cutoff values for identifying cells with DNA damage which are poorly justified and are not the norm in the field today.
In the new version of the manuscript we provided fleshes pointing to the co-localization events and the scale bars for all immunofluorescence experiments (Fig 1I; Figure 2B,F; Fig EV2E,G; Appendix Fig S1C). Moreover, for TIFs experiments we introduced enlarged sections of the nuclei in the representative images (Fig 2B,F; Fig EV2E; Appendix Fig S1C).
For quantifications of TRF2-TEL co-localizations (Fig 1I) and all TIFs data (Fig 2A,E, Fig EV2D, Appendix Fig S1B), new graphs show the average number of co-localization events. For PIFs data (Figure 2C,G; Fig EV2F), we used the quantification method reported in Mendez-Bermudez A. et al Mol Cell 2018.

Point by point remarks:
51 - Please specify in the abstract that the high-throughput screen was done with a set of candidate miRs. As written it suggests that all known miRs were screened.
We added this information in the Abstract Section.

74 - Human telomeres are composed BY à replace with OF
We replaced "by" with "of".

164 - TRF1 displays high sequence homology with TRF2 à sequence homology has little importance when it comes to targeting by miRs. Either the target sequence is there or it is not. The paragraph 162-170 has to be rephrased and I strongly recommend to shorten it.
As suggested by the Referee, we rephrased and shortened the indicated paragraph.

175 - in Fig S1C only mRNA expression data for Telomerase+ cell lines are shown. Please do not make general conclusions. Either rephrase to specify that TRF2 mRNA levels are not affected in telomerase-positive cell lines or provide data for ALT.
In the new version of the paper, we provided data on TRF2 mRNA levels in ALT U2-OS cells reported in Fig EV1C.

176 - correcte syntaxe : impacts something or an impact on something
This sentence was rephrased.

177 - The conclusion is beyond data provided. Only TRF2 staining is shown therefore no conclusions about TRF2 abundance at telomeres can be made. This figure needs to be replaced by a combined anti-TRF2 telomeric FISH approach.
To address Referee's request, we performed some new experiments. As suggested by the Referee, we combined TRF2 staining with a telomeric DNA FISH (Fig 1I). Moreover, we performed telomeric ChIP

assay using the anti-TRF2 antibody in both telomerase positive MDA-MB-231 cells and ALT positive U2-OS cells (Fig EV1D,E).

198 - reproducing the effect induced by siTRF2. Reference is missing. If Mendez-Bermudez is referred to, please note that data in this particual paper is analysed and presented differently.
This sentence was not referred to Mendez-Bermudez 2018 but to our data reported in the same panel (Fig. 2A,C).

229 - authors suggest that miR-182-3p induces a drastic change of cell morphology without specifying what this change might be. Indeed in S31-B figure we can observe that cells are rounded up and at least some look apoptotic, a phenotype that authors address elsewhere in the manuscript. Precisely because of this change in morphology, quantifying proliferation using a confluency approach may be problematic. Growth curves with population doublings need to be shown.
We agree with the Referee that morphological change induced by miR-182-3p could introduce a bias in the analysis. For this reason we provided new data showing cell number counted at the end of the experiment, (when the effect is more evident), in TNBC cells (new Fig 3C,D) and U2-OS cell line (Fig. EV3B). Moreover, a short description of the observed morphological change was introduced in the Results Section.

231-240: The paragraph about the cell cycle analysis is written in an ambiguous way without real conclusions. The opening sentence claims that they wanted to evaluate whether there is an inhibition of cell proliferation and/or apoptosis. Authors suggest that there is a reduced percentage of cells in the proliferative compartment but this phrase is misleading since it may be understood that there is a problem with replication. Authors do not mention that there is a reduction in G1 as well, therefore the only conclusion is that cells are dying. The paragraph should be shortened to one sentence.
As suggested by the Referee, in the new version of the work we reformulated and shortened the relative paragraph of the Results Section.

Figure 1B: Justify why only 6 out of 7 nucleotides recognized by miR in 3'UTR of TRF2 were deleted.
The deletion of a specific number of nucleotides in the 3'UTR of TRF2 basically depends on technical reasons such as primer design and mutagenesis kit used. However, many studies in the field of miRNAs have shown that even deletions of a smaller number of nucleotides in the miRNA target site are sufficient to prevent the targeting (Vinchure OS. et al 2021; Li T. et al 2020; Chipman LB. et al 2019;Chen G. et al 2019).

Figure 1C-E. Specify what miR-Control corresponds to. Provide statistics for all images. Is the difference in E statistically significant?
MiR-Control corresponds to a random sequence miRNA mimic molecule that has been extensively tested in human cell lines as indicated by the company (ThermoFisher Scientific). Further details on miR-Control have been added in the Materials and Methods Section.
We provided statistical analysis also for new Fig 1D (old Fig 1E), where the difference was not statistically significant.

Figure 1H: Image needs to be replaced with an immune-FISH. TRF2 signals colocalizing with telomeres need to be quantified and such quantification needs to be limited to G1 cells since telomeric content and therefore TRF2 signal at telomeres varies with the cell cycle. Imaging only 60 nuclei is not sufficient if cell phases are not discriminated.
To address this specific remark, we introduced the following data:

1) A new experiment of immune-FISH presented in Fig. 1I as suggested by the Referee.
2) New experiments of telomeric ChIP performed in Telomerase- and ALT-positive cells presented in Fig EV1 D,E.
3) Number of cells analyzed in Fig 1H was increased to 90 nuclei per condition.

Figure 2A: My recommendations are in the general remarks.

From representative images the suppression of TIFs by TRF2 overexpression is not evident. No enlarged sections shown.

In the new version of the manuscript, we replaced representative images of pBabe miR-182 and, as indicated in the general remark, we provided the enlargement section of the nuclei (Fig.2F).

Figure 3:

Please provide data for population doublings rather than confluence considering that you consider there is an effect or mir-182-3p on cell morphology. I recommend putting all FACS scatter plots into Supplemental data. Rather than labeling +/-Dox label with the gene that is modulated.

As described above (Point by point remarks), we provided new data of cell number in addition to the confluence analysis in the new Fig. 3C,D and Fig. EV3B.

Moreover, we moved FACS data of shBRCA2 MDA-MB-231 cells (previous Fig 3I,J) into the supplementary data (Appendix Fig S2G,H of the new version) and we changed the labeling adding the name of the gene.

Figure 4:

Consider putting histograms on log scale rather than cutting the axe.

As indicate above (General remarks), in the new version of the work we modified the graph presentation using the log scale (Fig. 4C,D, Fig. 5A,B and Fig EV4A).

Figure 5:

« Area of each PDTCs was measures » - not clear what this refers to, spheres? If so, you need to perform the proper assay to measure sphere size.

To address the point of the Referee, we changed the name “spheres” with the more general “3D cells” in the Figure legend 5. Moreover, to evaluate relative growth of PDTCs we measured their area in um^2 as generally used in various studies involving organoids and 3D cell cultures (Yoshida T. et al 2018; Cai T. et. Al 2018; Dinami et al 2021).

Figure S3:

Profiles for BrdU FACS are represented in an unconventional. It looks like most G1 cells were excluded and some G1 may be excluded as well.

We would like to apologize with the Referee for the misinterpretation. Indeed, FACS analysis reported in Appendix Fig S2I-L does not show the cells in the different phases of cell cycle. It represents, instead, the monoparametric analysis of BrdU positivity. To avoid confusion of the readers we included the negative control and we re-wrote the Figure Legend.

Figure S4C - legend does not specify for which organ the analysis was done. All organs sholud have been analyzed.

In agreement with the Referee, in the Figure legend EV4B of the new version of the manuscript, we specified that images of IHC referred to tumor tissue sections from mice bearing MDA-MB-436 human breast cancer xenografts.

The issue of the Referee was specifically addressed by performing H&E analysis in proliferative (bone marrow, skin, and intestine) and non-proliferative organs. Moreover, general markers of DNA damage (γHAX), apoptosis (Caspase 3), senescence (p21/p16/β-Gal) and proliferation (Ki67) were analyzed in proliferative organs. Systemic toxicity results were reported in three completely new Figures (Fig 6, Fig EV5, Appendix Fig S5).

Referee #3 (Comments on Novelty/Model System for Author):

Mouse is an appropriate model system to study efficacy of cancer therapies

Referee #3 (Remarks for Author):

Inhibition of telomerase and/or inducing telomere dysfunction remains a potentially powerful anti-cancer therapy. Indeed, strategies to target shelterin components such as TRF1 have shown promising results in in suppressing cancer growth. In the study by Dinami et al. the authors conduct a screen to identify microRNAs that can suppress the expression of TRF2, another component of shelterin and a factor that is critical for maintaining telomere function. They identify miR-182-3p as an efficient post-transcriptional regulator of TRF2 levels. Overexpression of miR-182-3p caused telomere dysfunction, double stranded DNA breaks at pericentromeric sites and consequently apoptosis in a number of human cancer cell lines. Delivery of miR-182-3p in liquid nanoparticles (LNPs) also caused telomere dysfunction in cancer cells transplanted into animals leading to impaired tumor growth, in addition to preventing growth of Patient-Derived Tumor Xenografts. This is an interesting and potentially important study as it reveals a potentially new anti-cancer therapy. However, a number of controls and experiments are missing that would make this study stronger. Major and minor comments are listed below.

Answer to Referee #3:
We thank the Referee for considering our study important for the development of a potentially new anti-cancer therapy. In the new version of the manuscript, we introduced new data to satisfy all the Referee's requests.

Major comments:
1) It is surprising that miR-179 182-3p does not affect viability of normal cells as animal experiments suggest. One would expect that TRF2 downregulation leads to cellular senescence or apoptosis in normal human somatic cells, just like TRF2 knockdown does, as demonstrated in previous studies. Data should be shown that reveal the effects of miR-182-3p on normal somatic human cells (cell proliferation curves, SAbGal, TUNEL, gH2AX, etc) and on normal cells in mouse tissue (see below).
According to the Referee's request, we have evaluated the effects of miR-182-3p on MCF10A (normal epithelial cell line from mammary gland) and normal mouse tissues (see point 5). Results on MCF10A cells were reported in Fig. EV3E-J.

2) Fig 1H shows that miR-179 182-3p decreases telomere signal intensities, while a miR-179 182-3p inhibitor enhances TRF2 signal intensity in an ALT cell line, U2-OS cells. It is unclear why is this relevant for this study that primarily uses MDA-MB-231 and MDA-MB-436 cells? Along these lines, based on Figure 2B and F, telomere signal intensities do not appear to be affected by miR-179 182-3p in MDA 231 cancer cells (and in HeLa cells shown in Fig S2D), which is unlike U2OS cells (Fig 1H). Why is this? Telomere signal intensity reduction, following miR-179 182-3p treatment, and enhancement, following miR-179 182-3p inhibitor treatment, should be shown for at least one of these telomerase expressing cancer cells in Figure 1H.
Data presented in Fig 1H shows TRF2 spots intensity analysis in U2-OS cells (performed with anti-TRF2 antibody) and not telomere signals. In particular, among telomerase- and ALT-positive cell lines of Fig.1F,G, we chose U2-OS model for technical reasons as they show higher spots intensity of TRF2 and a lower background compared to breast cancer cell lines, allowing a more accurate analysis. Moreover, new data regarding the effect of miR-182-3p in U2-OS have been introduced in the new version of the manuscript (Fig EV3A-D, Appendix Fig. S1, see point 3).

However, as required from the Referee, telomere length analysis was performed by telomeric DNA FISH in MDA-MB-231 cells. The results are presented in the new Fig EV2B.

3) If data on U2OS cells will remain in the study, more data on these cells should be shown. For example, does miR-179 182-3p also cause TIF, PIF, and apoptosis in ALT cells, such as U2OS cells? These data should be shown in Figure 2, or in the supplemental Figures, as it would be highly relevant when considering treating ALT cancers

As required by the Referee, in the revised manuscript we provided more data on U2-OS. In particular, we show:

1) TRF2 mRNA levels upon transfection with miR-182-3p (Fig EV1C).
2) Analysis of TRF2 abundance at telomeres by TRF2 immunofluorescence combined with telomeric DNA FISH (Fig 1I).
3) Analysis of TRF2 abundance at telomeres by telomeric ChIP assay (Fig EV1E).
4) Global and telomeric DNA damage analysis (Appendix Fig S1).
5) Cell growth and apoptosis data (Fig. EV3A-D).

4) Data should be shown that would reveal why miR-179 182-3p causes apoptotic cell death. For example, is the miR-179 182-3p-induced apoptotic cell death response due to telomeric bfb cycles? Evidence for or against this this should be provided.

According to the request of Referee, since BFB cycle is a mechanism of genomic instability occurring as a result of telomere loss, we performed telomeric FISH on metaphase spreads to evaluate the presence of telomeric aberrations such as telomere free ends, sister chromatid fusions and telomere fragility. Moreover, the ability of miRNA to induce micronuclei, a general marker of genomic instability was also evaluated at day 3 and 6 post-transfection. The results were reported in Appendix Fig S3.

5) More data should be shown on the absence/presence of side effects of administering LNP containing miR-179 182-3p on non-tumor tissue in animal experiments. Markers of senescence (SA-bGal, p16, p21, gH2AX, ...) or apoptosis (TUNEL) should be analyzed, in addition to morphological changes in normal tissue. Also, levels of TRF2 should be compared in normal tissue, similar to what is shown for the tumors.

To address the Referee's require, we performed H&E analysis in proliferative (bone marrow, skin, and intestine) and non-proliferative organs. Moreover, general markers of DNA damage (γHAX), apoptosis (Caspase 3 activated), senescence (p21/p16/β-Gal) and proliferation (Ki67), as well as TRF2, were analyzed in proliferative organs. Systemic toxicity results were reported in three completely new Figures (Fig 6, Fig EV5, Appendix Fig S5).

Minor comments:

- It should be discussed whether TRF2 inhibition also affects non-telomeric functions of TRF2. This seems unlikely given that the primary response to miR-182-3p is apoptosis induced by telomere dysfunction.

Although primary response to miR-182-3p consists in apoptosis induced by telomere-dysfunction, IHC analysis of tumor tissues of mice treated with LNPs-miR-182-3p showed a reduction of CD31 (marker of blood vessels). This is in line with the well demonstrated non-telomeric function of TRF2 in angiogenesis and suggests a possible contribution of TRF2-mediated cell-extrinsic mechanisms to the anti-tumoral activity of miR-182-3p *in vivo*. We introduced a sentence in the Discussion Section.

- The role of TRF2 at pericentromeric sites, and how it suppresses a DDR there, should be mentioned.

We introduced a sentence about the role of TRF2 at pericentromeric sites in the Introduction section.

- It should be discussed why BRCA1 compromised cancers are more sensitive to TRF2 inhibition.

We introduced a brief paragraph on this point in the Discussion section.

10th Aug 2022

Dear Dr. Biroccio,

Thank you for the submission of your revised manuscript to EMBO Molecular Medicine, and please accept my apologies for the delay in getting back to you as I was hoping to receive the report from referee #2. However, as we have not heard from this referee yet, and in order not to delay the process further, we prefer to make a decision on your manuscript with the two reports at hand.

As you will see from the reports below, while referee #1 is satisfied with the revised manuscript, referee #3 raises a number of important concerns that should be addressed before further consideration.
I further cross-commented with referee #1 who stated:
"I agree with the reviewer 3' comments about the impact of miR-182-3p on normal somatic human cells. Additional experiments proposed by this reviewer help strengthen the manuscript. Although the authors concluded that miR-182-3p treatment resulted only in a slight increase of senescent cells compared to cancer cells, it is important to realize that even few senescent cells may contribute to aging through SASP. Thus, it may be helpful to determine SASP concentration in miR-182-3p-treated normal somatic human cells, along with the culture medium."

Therefore, we would like you to revise the manuscript further and address all the points raised by the referees. As EMBO Press usually encourages a single round of revisions, please be aware that this will be the last chance for you to address these points.

Additionally, please also address the following editorial issues:
- Please add a legend and title for Table EV2.
- Please provide The Paper Explained section. EMBO Molecular Medicine articles are accompanied by a summary of the articles to emphasize the major findings in the paper and their medical implications for the non-specialist reader. Please provide a draft summary of your article highlighting
- the medical issue you are addressing,
- the results obtained and
- their clinical impact.
This may be edited to ensure that readers understand the significance and context of the research. Please refer to any of our published articles for an example.
- Please remove the red text (including in the Appendix).
- Your manuscript contains error bars based on n=2 (Fig. EV1A). Please use scatter blots showing the individual datapoints in these cases. The use of statistical tests needs to be justified.
- As part of the EMBO Publications transparent editorial process initiative (see our Editorial at http://embomolmed.embopress.org/content/2/9/329), EMBO Molecular Medicine will publish online a Review Process File (RPF) to accompany accepted manuscripts.
This file will be published in conjunction with your paper and will include the anonymous referee reports, your point-by-point response and all pertinent correspondence relating to the manuscript. Let us know whether you agree with the publication of the RPF and as here, if you want to remove or not any figures from it prior to publication.
Please note that the Authors checklist will be published at the end of the RPF.

I look forward to receiving your revised manuscript.

With kind regards,

Lise Roth

Lise Roth, PhD
Senior Editor
EMBO Molecular Medicine

***** Reviewer's comments *****

Referee #1 (Remarks for Author):

The authors have addressed all my comments. I have no further questions

Referee #3 (Remarks for Author):

Major comments:
In previous submission, this reviewer raised concerns about the possibility that the potential therapeutic molecule miR-182-3p also negatively affects normal cells and requested that the authors test whether and to what degree miR-182-3p causes senescence, apoptosis, and other defects also in normal somatic human cells. The new experiments conducted using the non-invasive breast epithelial cell line MCF10A, however, are not ideal as this cell line not only expresses telomerase, which protects cells from telomere dysfunction, but also is defective in at least one senescence pathway: p16 mediated senescence.

In addition, based on the new data conducted using MCF10A cells and now included in the study (Fig EV3G-J), the author state that miR-182-3p did not have significant impact on DNA damage, proliferation was only transiently inhibited, resulted only in a slight increase of apoptotic and senescent cells compared to cancer cells. While all this appears to be accurate, the data are not described or presented in the same rigorous and unbiased manner as data using cancer cells. For example, similar to MDA-MB-231 (Fig 3B), MCF10A cells regain some proliferation 4 days after transfection of miR-182-3p (FigEV3F). This is highlighted in the text to describe the transient growth arrest of "normal cells" but it is not mentioned when describing the effects of the miR-182-3p on MDA-MB-321 cancer cells (Fig 3B). Also, the y axis of the bar graph depicting increases in SA-bGal expressing MCF10 cells treated with miR-182-3p is shown at a scale of 100%, which obviously minimizes the visual impact of the statistically significant increases, while minimal differences in cancer cells treated with miR-182-3p are shown at scales that maximizes visual impact (see all other bar graphs). To be balanced and unbiased, authors should adjust the axes of all graphs consistently.

Cancer cells were consistently transfected twice with miR-182-3p. Were these same conditions used also for MCF10 cells? This should be clearly described in the text. If MCF10A cells were transfected only once, authors should repeat transfections using identical procedures used for cancer cells.

The authors state that miR-182-3p had no significance impact of DNA damage, however only a western blot for gH2AX is shown. Authors should support this conclusion by showing representative images of gH2AX foci, as well as a quantification of these foci/cell in control and miR-182-3p transfected MCF10A cells. TIF should also be analyzed and quantified in these cells, just like the authors did for cancer cells.

As requested in the previous review, the authors should analyze the effects (senescence, apoptosis, gH2AX foci, TIF, proliferation) of miR-182-3p on normal somatic human cells treated in an identical manner as cancer cells. Appropriate cells to use would be, for example, primary human fibroblasts or primary human epithelial cells that display functional senescence responses and absence of telomerase activity.

The following statement in line 453 "thus allowing us to exclude important side effects of administering LNP-miR-182-3p in vivo" should be removed as this is a very strong statement that is not supported by the data. In order to exclude side effects, a much more detailed study would have to be conducted.

If new data demonstrate that normal somatic human cells indeed are less sensitive to miR-182-3p compared to cancer cells, the authors should include a paragraph in the Discussion that discusses these surprising results. For example, why does miR-182-3p cause a reduction in TRF2 protein levels in both cancer cells and normal cells (at similar levels), but only causes significant proliferative defects and apoptosis/senescence in cancer cells? It would be important to know what the potential reasons for these differences are, since this is not apparent.

Minor comments:
What exactly are the miR-controls used in cell culture experiments and animal experiments? This information should be included in the manuscript.

REPLY TO REFEREE' COMMENTS

Dinami et al. 2022

Referee #1 (Remarks for Author):
"I agree with the reviewer 3' comments about the impact of miR-182-3p on normal somatic human cells. Additional experiments proposed by this reviewer help strengthen the manuscript. Although the authors concluded that miR-182-3p treatment resulted only in a slight increase of senescent cells compared to cancer cells, it is important to realize that even few senescent cells may contribute to aging through SASP. Thus, it may be helpful to determine SASP concentration in miR-182-3p-treated normal somatic human cells, along with the culture medium."
As required also from the Referee #3, we performed new experiments to study the effects of miR-182-3p on primary human fibroblast (BJ). In addition, we analyzed Senescence-Associated Secretory Phenotype (SASP) in the cell culture medium as indicated by this Referee. All the data performed with BJ cells are presented in the new Figure 4.

Referee #3 (Remarks for Author):

Major comments:

In previous submission, this reviewer raised concerns about the possibility that the potential therapeutic molecule miR-182-3p also negatively affects normal cells and requested that the authors test whether and to what degree miR-182-3p causes senescence, apoptosis, and other defects also in normal somatic human cells. The new experiments conducted using the non-invasive breast epithelial cell line MCF10A, however, are not ideal as this cell line not only expresses telomerase, which protects cells from telomere dysfunction, but also is defective in at least one senescence pathway: p16 mediated senescence.
We thank the Referee for this interesting observation. Thus, to satisfy Referee request, we performed new experiments to investigate the effects of miR-182-3p on primary human skin fibroblasts (BJ) which do not express telomerase and are not defective for p16 pathway (see below).

In addition, based on the new data conducted using MCF10A cells and now included in the study (Fig EV3G-J), the author state that miR-182-3p did not have significant impact on DNA damage, proliferation was only transiently inhibited, resulted only in a slight increase of apoptotic and senescent cells compared to cancer cells. While all this appears to be accurate, the data are not described or presented in the same rigorous and unbiased manner as data using cancer cells. For example, similar to MDA-MB-231 (Fig 3B), MCF10A cells regain some proliferation 4 days after transfection of miR-182-3p (FigEV3F). This is highlighted in the text to describe the transient growth arrest of "normal cells" but it is not mentioned when describing the effects of the miR-182-3p on MDA-MB-321 cancer cells (Fig 3B).
In the new version of the manuscript, we now included data about the effect of miR-182-3p on primary human fibroblast (BJ), the normal cell model required from the Referee. The results have been added in a new figure (Fig 4),while the data on MCF10A have been moved in figure EV3. Finally, the effect of miR-182-3p on normal versus cancer cells has been discussed.

Also, the y axis of the bar graph depicting increases in SA-bGal expressing MCF10 cells treated with miR-182-3p is shown at a scale of 100%, which obviously minimizes the visual impact of the

statistically significant increases, while minimal differences in cancer cells treated with miR-182-3p are shown at scales that maximizes visual impact (see all other bar graphs). To be balanced and unbiased, authors should adjust the axes of all graphs consistently.
We modified the axes of senescence graph of MCF10A according to the observation of the Referee.

Cancer cells were consistently transfected twice with miR-182-3p. Were these same conditions used also for MCF10 cells? This should be clearly described in the text. If MCF10A cells were transfected only once, authors should repeat transfections using identical procedures used for cancer cells.
For proliferation/senescence/apoptosis experiments, normal cells (MCF10A and BJ) were transfected twice with miR-182-3p as we did for cancer cells. We provided this information in the relative figure legend of the new version of the manuscript.

The authors state that miR-182-3p had no significance impact of DNA damage, however only a western blot for gH2AX is shown. Authors should support this conclusion by showing representative images of gH2AX foci, as well as a quantification of these foci/cell in control and miR-182-3p transfected MCF10A cells. TIF should also be analyzed and quantified in these cells, just like the authors did for cancer cells.
As required by Referee, we added new experiments on DNA damage caused by miR-182-3p in MCF10A cells. In particular, we provided the quantification of gH2AX foci/cell and the analysis of TIFs, and we included the representative images.

As requested in the previous review, the authors should analyze the effects (senescence, apoptosis, gH2AX foci, TIF, proliferation) of miR-182-3p on normal somatic human cells treated in an identical manner as cancer cells. Appropriate cells to use would be, for example, primary human fibroblasts or primary human epithelial cells that display functional senescence responses and absence of telomerase activity.
As indicated above, we performed new experiments to study the effects of miR-182-3p on primary human fibroblast (BJ). In addition, we also analyzed Senescence-Associated Secretory Phenotype (SASP) in the cell culture medium as indicated from Referee #1. All the data performed with BJ cells are presented in the new Figure 4.

The following statement in line 453 "thus allowing us to exclude important side effects of administering LNP-miR-182-3p in vivo" should be removed as this is a very strong statement that is not supported by the data. In order to exclude side effects, a much more detailed study would have to be conducted.
According to the right observation of the Referee, we have changed the sentence indicated in line 453.

If new data demonstrate that normal somatic human cells indeed are less sensitive to miR-182-3p compared to cancer cells, the authors should include a paragraph in the Discussion that discusses these surprising results. For example, why does miR-182-3p cause a reduction in TRF2 protein levels in both cancer cells and normal cells (at similar levels), but only causes significant proliferative defects and apoptosis/senescence in cancer cells? It would be important to know what the potential reasons for these differences are, since this is not apparent.
The effect of miR-182-3p on normal versus cancer cells has been added in the Discussion section.

Minor comments:
What exactly are the miR-controls used in cell culture experiments and animal experiments? This information should be included in the manuscript.
This information has been added in the new version of the manuscript (Materials and Methods).

14th Oct 2022

Dear Dr. Biroccio,

Thank you for the submission of your revised manuscript to EMBO Molecular Medicine. I have now received the report from referee #3. While most concerns have been addressed, this referee raises a valid point regarding the blot in the new Figure 4A. We would therefore like you to please provide quantification for this blot, as well as the corresponding Source Data (annotated uncropped blots with molecular weights). Please also indicate how many times the experiment was performed (technical and biological replicates).

Kindly also let us know whether you agree with the publication of the Review Process File (This file will be published in conjunction with your paper and will include the anonymous referee reports, your point-by-point response and all pertinent correspondence relating to the manuscript. Let us know whether you agree with the publication of the RPF and as here, if you want to remove or not any figures from it prior to publication. Please note that the Authors checklist will be published at the end of the RPF.)

I look forward to receiving your revised manuscript.

With kind regards,

Lise Roth

Lise Roth, PhD
Senior Editor
EMBO Molecular Medicine

***** Reviewer's comments *****

Referee #3 (Remarks for Author):

The authors have addressed my concerns, yet data that was newly included is problematic for the following reason. In previous submission, this reviewer requested that the authors analyze the effects of miR-182-3p on normal somatic human cells. The authors tested the primary human fibroblasts BJ cells, an adequate cell strain. While the cells responded to miR-182-3p transfection as expected (telomere dysfunction and rapid activation of senescence), the immunoblot in Figure 4A does not demonstrate that miR-182-3p reduces TRF2 expression, as the authors claim in lines 448-449. In contrast, the immunoblot shows additional bands migrating at higher molecular-weight compared to the band in control transfected BJ cells. This needs to be resolved.

REPLY TO REFEREE' COMMENTS

Dinami et al. 2022

Referee #3 (Remarks for Author):

The authors have addressed my concerns, yet data that was newly included is problematic for the following reason. In previous submission, this reviewer requested that the authors analyze the effects of miR-182-3p on normal somatic human cells. The authors tested the primary human fibroblasts BJ cells, an adequate cell strain. While the cells responded to miR-182-3p transfection as expected (telomere dysfunction and rapid activation of senescence), the immunoblot in Figure 4A does not demonstrate that miR-182-3p reduces TRF2 expression, as the authors claim in lines 448-449. In contrast, the immunoblot shows additional bands migrating at higher molecular-weight compared to the band in control transfected BJ cells. This needs to be resolved.

We thank Referee #3 for this observation. To resolve this point, we have now included quantification of three independent Western Blotting experiments. Moreover, based on the quantification, we replaced WB in Figure 4A with another biological replicate more representative of the mean value and we indicated with asterisk (*) the unspecific bands.
Finally, we provided Source Data (uncropped blot) for new blot in Figure 4A, showing that TRF2 specific band corresponds to the lower band (about 66kDa) under the 75kDa marker.

27th Oct 2022

Dear Dr. Biroccio,

Thank you for addressing the last concern from referee #3. I am pleased to inform you that we will be able to accept the manuscript once the following editorial concerns will be addressed:

1/ Manuscript text:
- Please address the queries from our data editor in the Data edited ms file. Please indicate in track changes mode any new modification included in the manuscript.
- We note that there are three first authors on the manuscript. Do you confirm equal contribution of these 3 authors?
- Material sand Methods:
o Please indicate whether the cells were authenticated and tested for mycoplasma contamination.
o Please indicate the dilutions used for the antibodies.
- Data Availability Section: Please note that this section is limited to large datasets produced in this study. The link you provided seems to give access to the raw data underlying your figures, i.e. the Source Data. If this is the case, please note that these files should be uploaded as Source Data files (1 file per figure). If you have no data that requires deposition in a public database (such as transcriptomics, sequencing, etc), please state so in this section: "This study includes no data deposited in external repositories."
- Author contributions: CRediT has replaced the traditional author contributions section because it offers a systematic machine readable author contributions format that allows for more effective research assessment. Please remove the Authors Contributions from the manuscript and use the free text boxes beneath each contributing author's name in our system to add specific details on the author's contribution. More information is available in our guide to authors.
- Please remove "Supporting information" (between the COI and References).
- Thank you for providing The paper explained. Please include it in the main manuscript text file.

2/ Figures:
- Please indicate in the legend of Figure 4A that the graph represents the quantification of 3 independent experiments and
- Fig. EV1A contains error bars based on n=2. The use of statistical tests needs to be justified, and we encourage you to remove the error bars.

3/ Checklist:
- Please fill in your name and manuscript number (top left corner)
- Please indicate whether the cells were authenticated and tested for mycoplasma contamination ("Cell materials")
- Please indicate whether data describe technical or biological replicates.
- Please check whether the Data availability section is properly filled.

4/ Synopsis:
I slightly edited your text, please let me know if you agree with the following, or amend as you see fit:
A miRNA-based strategy to inhibit the telomeric protein TRF2 was developed, which led to efficient decrease of triple negative breast cancer growth.

- miR-182-3p was identified as an efficient regulator of TRF2 expression in human cancer through high-throughput miRNA luciferase screening.
- TRF2 inhibition by miR-182-3p induced DNA damage at telomeric and pericentromeric sites and consequent genomic instability
- miR-182-3p limited the growth of Triple Negative Breast Cancer (TNBC) models by activating apoptosis
- Lipid nanoparticles (LNPs) containing miR-182-3p reduced tumor volume in vivo in various TNBC models, including Olaparib-resistant patient-derived tumor xenografts
- LNPs-miR-182-3p crossed the blood brain barrier, showing therapeutic potential against brain metastasis

Thank you for providing a nice synopsis picture. Please resize it as a PNG file 550 px wide x 300-600 px high, and make sure the text remains legible.

5/ As part of the EMBO Publications transparent editorial process initiative (see our Editorial at http://embomolmed.embopress.org/content/2/9/329), EMBO Molecular Medicine will publish online a Review Process File (RPF) to accompany accepted manuscripts.
This file will be published in conjunction with your paper and will include the anonymous referee reports, your point-by-point response and all pertinent correspondence relating to the manuscript. Let us know whether you agree with the publication of the RPF and as here, if you want to remove or not any figures from it prior to publication.
Please note that the Authors checklist will be published at the end of the RPF.

I look forward to receiving your revised manuscript.

Yours sincerely,

Lise Roth

Lise Roth, PhD
Senior Editor
EMBO Molecular Medicine

The authors addressed the minor editorial issues.

5th Nov 2022

Dear Dr. Biroccio,

Thank you for submitting your revised files. I am pleased to inform you that your manuscript is now accepted for publication in EMBO Molecular Medicine!

There is one last editorial point that needs however to be addressed: I could not find your modifications in response to the data editor queries (in Data Edited ms file).
In case you did not find this file, I copy their queries here:
- Fig. 1I: define the arrowheads
- Fig. 2B, D, F, H: define the arrowheads
- Fig. 3F, H, J: define the number and the nature, i.e. biological or technical, of the replicates.
- Fig. 4F (now 5F): There are more than 3 data points per group plotted in the graphs, please clarify.
- Fig. 4H (now 5H): Data in C, D and H are presented as boxplots. Please define the central band, boxes and whiskers of the boxplot.
- Fig. 5A, B, C, D (now 6A, B, C, D): Please define the number and the nature, i.e. biological or technical, of the replicates.
- Fig. 5G (now 6G): Please define the size of the scale bar.
- Fig. 5H (now 6H): There are more than 3 data points per group plotted in the graphs, please clarify.
- Fig. 6A, D, G (now 7A, D, G): Please define the size of the scale bar.
- Fig. 6 (now 7), Data information: There are more than 3 data points per group plotted in the graphs, please clarify.
- Fig EV1A: Please define the number and the nature, i.e. biological or technical, of the replicates.
- Fig. EV4: There are more than 3 data points per group plotted in some of the graphs, please clarify.

Please address their concerns, accept all changes, and send us the final manuscript via email. We will then send the manuscript files to our publisher to be published in the next available issue.

Thank you for bearing with these last minor issues, and congratulations on your interesting work!

With kind regards,

Lise Roth

Lise Roth, Ph.D
Senior Editor
EMBO Molecular Medicine

Follow us on Twitter @EmboMolMed
Sign up for eTOCs at embopress.org/alertsfeeds

EMBO Press Author Checklist

Corresponding Author Name: Annamaria Biroccio	
Journal Submitted to: EMBO Molecular Medicine	
Manuscript Number: EMM-2022-16033	

USEFUL LINKS FOR COMPLETING THIS FORM
The EMBO Journal - Author Guidelines
EMBO Reports - Author Guidelines
Molecular Systems Biology - Author Guidelines
EMBO Molecular Medicine - Author Guidelines

Reporting Checklist for Life Science Articles (updated January 2022)
This checklist is adapted from Materials Design Analysis Reporting (MDAR) Checklist for Authors. MDAR establishes a minimum set of requirements in transparent reporting in the life sciences (see Statement of Task: 10.31222/osf.io/9sm4x). Please follow the journal's guidelines in preparing your manuscript.
Please note that a copy of this checklist will be published alongside your article.

Abridged guidelines for figures
1. Data
The data shown in figures should satisfy the following conditions:
- the data were obtained and processed according to the field's best practice and are presented to reflect the results of the experiments in an accurate and unbiased manner.
- ideally, figure panels should include only measurements that are directly comparable to each other and obtained with the same assay.
- plots include clearly labeled error bars for independent experiments and sample sizes. Unless justified, error bars should not be shown for technical replicates.
- if n<5, the individual data points from each experiment should be plotted. Any statistical test employed should be justified.
- Source Data should be included to report the data underlying figures according to the guidelines set out in the authorship guidelines on Data Presentation.

2. Captions
Each figure caption should contain the following information, for each panel where they are relevant:
- a specification of the experimental system investigated (eg cell line, species name).
- the assay(s) and method(s) used to carry out the reported observations and measurements.
- an explicit mention of the biological and chemical entity(ies) that are being measured.
- an explicit mention of the biological and chemical entity(ies) that are altered/varied/perturbed in a controlled manner.
- the exact sample size (n) for each experimental group/condition, given as a number, not a range;
- a description of the sample collection allowing the reader to understand whether the samples represent technical or biological replicates (including how many animals, litters, cultures, etc.).
- a statement of how many times the experiment shown was independently replicated in the laboratory.
- definitions of statistical methods and measures:
  - common tests, such as t-test (please specify whether paired vs. unpaired), simple χ2 tests, Wilcoxon and Mann-Whitney tests, can be unambiguously identified by name only, but more complex techniques should be described in the methods section;
  - are tests one-sided or two-sided?
  - are there adjustments for multiple comparisons?
  - exact statistical test results, e.g., P values = x but not P values < x;
  - definition of 'center values' as median or average;
  - definition of error bars as s.d. or s.e.m.

Please complete ALL of the questions below.
Select "Not Applicable" only when the requested information is not relevant for your study.

Materials

Newly Created Materials	Information included in the manuscript?	In which section is the information available? (Reagents and Tools Table, Materials and Methods, Figures, Data Availability Section)
New materials and reagents need to be available; do any restrictions apply?	Yes	Materials and Methods

Antibodies	Information included in the manuscript?	In which section is the information available? (Reagents and Tools Table, Materials and Methods, Figures, Data Availability Section)
For **antibodies** provide the following information:<br>- Commercial antibodies: RRID (if possible) or supplier name, catalogue number and or/clone number<br>- Non-commercial: RRID or citation	Yes	Materials and Methods

DNA and RNA sequences	Information included in the manuscript?	In which section is the information available? (Reagents and Tools Table, Materials and Methods, Figures, Data Availability Section)
**Short novel DNA or RNA including primers, probes**: provide the sequences.	Yes	Materials and Methods

Cell materials	Information included in the manuscript?	In which section is the information available? (Reagents and Tools Table, Materials and Methods, Figures, Data Availability Section)
**Cell lines:** Provide species information, strain. Provide accession number in repository **OR** supplier name, catalog number, clone number, and/**OR** RRID.	Yes	Materials and Methods
**Primary cultures:** Provide species, strain, sex of origin, genetic modification status.	Not Applicable	
Report if the cell lines were recently **authenticated** (e.g., by STR profiling) and tested for mycoplasma contamination.	Yes	Materials and Methods

Experimental animals	Information included in the manuscript?	In which section is the information available? (Reagents and Tools Table, Materials and Methods, Figures, Data Availability Section)
**Laboratory animals or Model organisms:** Provide species, strain, sex, age, genetic modification status. Provide accession number in repository **OR** supplier name, catalog number, clone number, **OR** RRID.	Yes	Materials and Methods
**Animal observed in or captured from the field:** Provide species, sex, and age where possible.	Not Applicable	
Please detail **housing and husbandry conditions**.	Yes	Materials and Methods

Plants and microbes	Information included in the manuscript?	In which section is the information available? (Reagents and Tools Table, Materials and Methods, Figures, Data Availability Section)
**Plants:** provide species and strain, ecotype and cultivar where relevant, unique accession number if available, and source (including location for collected wild specimens).	Not Applicable	
**Microbes:** provide species and strain, unique accession number if available, and source.	Not Applicable	

Human research participants	Information included in the manuscript?	In which section is the information available? (Reagents and Tools Table, Materials and Methods, Figures, Data Availability Section)
If collected and within the bounds of privacy constraints report on age, sex and gender or ethnicity for all study participants.	Not Applicable	

Core facilities	Information included in the manuscript?	In which section is the information available? (Reagents and Tools Table, Materials and Methods, Figures, Data Availability Section)
If your work benefited from core facilities, was their service mentioned in the acknowledgments section?	Not Applicable	

Design

Study protocol	Information included in the manuscript?	In which section is the information available? (Reagents and Tools Table, Materials and Methods, Figures, Data Availability Section)
If study protocol has been **pre-registered, provide DOI in the manuscript**. For clinical trials, provide the trial registration number **OR** cite DOI.	Not Applicable	
Report the **clinical trial registration number** (at ClinicalTrials.gov or equivalent), where applicable.	Not Applicable	

Laboratory protocol	Information included in the manuscript?	In which section is the information available? (Reagents and Tools Table, Materials and Methods, Figures, Data Availability Section)
Provide DOI OR other citation details if **external detailed step-by-step protocols** are available.	Not Applicable	

Experimental study design and statistics	Information included in the manuscript?	In which section is the information available? (Reagents and Tools Table, Materials and Methods, Figures, Data Availability Section)
Include a statement about **sample size** estimate even if no statistical methods were used.	Yes	Figures
Were any steps taken to minimize the effects of subjective bias when allocating animals/samples to treatment (e.g. **randomization procedure**)? If yes, have they been described?	Yes	Materials and Methods
Include a statement about **blinding** even if no blinding was done.	Yes	investigators were blinded when quantifying microscopy and immunofluorescence images.
Describe **inclusion/exclusion criteria** if samples or animals were excluded from the analysis. Were the criteria pre-established?<br><br>If sample or data points were omitted from analysis, report if this was due to attrition or intentional exclusion and provide justification.	Not Applicable	
For every figure, are **statistical tests** justified as appropriate? Do the data meet the assumptions of the tests (e.g., normal distribution)? Describe any methods used to assess it. Is there an estimate of variation within each group of data? Is the variance similar between the groups that are being statistically compared?	Yes	Figure legends

Sample definition and in-laboratory replication	Information included in the manuscript?	In which section is the information available? (Reagents and Tools Table, Materials and Methods, Figures, Data Availability Section)
In the figure legends: state number of times the experiment was **replicated** in laboratory.	Yes	Figure legends
In the figure legends: define whether data describe **technical or biological replicates**.	Yes	Figure legends

Ethics

Ethics	Information included in the manuscript?	In which section is the information available? (Reagents and Tools Table, Materials and Methods, Figures, Data Availability Section)
Studies involving **human participants**: State details of **authority granting ethics approval** (IRB or equivalent committee(s), provide reference number for approval.	Not Applicable	
Studies involving **human participants**: Include a statement confirming that **informed consent** was obtained from all subjects and that the experiments conformed to the principles set out in the WMA Declaration of Helsinki and the Department of Health and Human Services Belmont Report.	Not Applicable	
Studies involving **human participants:** For publication of **patient photos**, include a statement confirming that consent to publish was obtained.	Not Applicable	
Studies involving experimental **animals**: State details of **authority granting ethics approval** (IRB or equivalent committee(s), provide reference number for approval. Include a statement of compliance with ethical regulations.	Yes	Materials and Methods
Studies involving **specimen and field samples:** State if relevant **permits** obtained, provide details of authority approving study; if none were required, explain why.	Not Applicable	

Dual Use Research of Concern (DURC)	Information included in the manuscript?	In which section is the information available? (Reagents and Tools Table, Materials and Methods, Figures, Data Availability Section)
Could your study fall under dual use research restrictions? Please check biosecurity documents and list of **select agents and toxins** (CDC): https://www.selectagents.gov/sat/list.htm	Not Applicable	
If you used a select agent, is the security level of the lab appropriate and reported in the manuscript?	Not Applicable	
If a study is subject to dual use research of concern regulations, is the name of the **authority granting approval and reference number** for the regulatory approval provided in the manuscript?	Not Applicable	

Reporting

The MDAR framework recommends adoption of discipline-specific guidelines, established and endorsed through community initiatives. Journals have their own policy about requiring specific guidelines and recommendations to complement MDAR.

Adherence to community standards	Information included in the manuscript?	In which section is the information available? (Reagents and Tools Table, Materials and Methods, Figures, Data Availability Section)
State if relevant guidelines or checklists (e.g., **ICMJE, MIBBI, ARRIVE, PRISMA**) have been followed or provided.	Yes	Materials and Methods
For **tumor marker prognostic studies**, we recommend that you follow the **REMARK** reporting guidelines (see link list at top right). See author guidelines, under 'Reporting Guidelines'. Please confirm you have followed these guidelines.	Not Applicable	
For **phase II and III randomized controlled trials**, please refer to the **CONSORT** flow diagram (see link list at top right) and submit the CONSORT checklist (see link list at top right) with your submission. See author guidelines, under 'Reporting Guidelines'. Please confirm you have submitted this list.	Not Applicable	

Data Availability

Data availability	Information included in the manuscript?	In which section is the information available? (Reagents and Tools Table, Materials and Methods, Figures, Data Availability Section)
Have **primary datasets** been deposited according to the journal's guidelines (see 'Data Deposition' section) and the respective accession numbers provided in the Data Availability Section?	Yes	Data Avaibility Section
Were **human clinical and genomic datasets** deposited in a public access-controlled repository in accordance to ethical obligations to the patients and to the applicable consent agreement?	Not Applicable	
Are **computational models** that are central and integral to a study available without restrictions in a machine-readable form? Were the relevant accession numbers or links provided?	Not Applicable	
If publicly available data were reused, provide the respective **data citations in the reference list**.	Not Applicable	
